# Landscape of retron diversity across the SPIRE microbial metagenome resource reveals candidate novel type XI-like lineages

**DOI:** 10.1128/msystems.00732-26

**Published:** 2026-09-11

**Authors:** Nicolás Toro

**Affiliations:** 1 Department of Soil and Plant Microbiology, Estación Experimental del Zaidín, Consejo Superior de Investigaciones Científicas, Microbiome Engineering, Reverse-Transcriptases, and regulatory RNA (MIRTA) 73025, Granada, Spain; University of Birmingham, Birmingham, United Kingdom

**Keywords:** retrons, reverse transcriptases, non coding RNA, metagenomes, effectors

## Abstract

Retrons are bacterial genetic elements encoding a specialized reverse transcriptase (RT) that synthesizes multicopy single-stranded DNA and are increasingly recognized as components of bacterial anti-phage defense systems. However, their diversity and ecological distribution across large-scale genomic resources remain poorly characterized. Here, we surveyed retron RTs across the SPIRE representative metagenome collection, a non-redundant, species-level data set spanning diverse microbial habitats. Using a curated panel of type-specific hidden Markov models, we identified retrons representing all canonical types together with additional divergent lineages. Retron distribution showed strong taxonomic and ecological structuring, with some groups restricted to specific bacterial phyla, whereas others were broadly distributed across environmental categories. Systematic novelty assessment identified two candidate type XI-like lineages, TXI_C2like and TXI_noncan_h, characterized by protease-independent architectures and distinct accessory modules associated with WYL- and DnaB_C-containing proteins, respectively. *De novo* covariance-based analyses further identified candidate msr/msd-like non-coding RNA structures in both lineages, supporting conservation of the canonical RT–ncRNA organizational framework despite extensive sequence divergence. Together, these findings expand the known diversity of retron systems and identify type XI-like retrons as a dynamic and previously underexplored evolutionary group.

**IMPORTANCE:**

Retrons are bacterial genetic elements that are increasingly exploited as programmable tools for genome editing, molecular recording, and biosensing in addition to their natural role in anti-phage defense. Despite this growing biotechnological interest, the true diversity of retrons across the bacterial world has remained largely unmapped. By mining a resource of over 100,000 processed microbial metagenomes, we uncovered thousands of retron sequences spanning known types as well as previously unrecognized lineages and found that their distribution is strongly shaped by both bacterial taxonomy and ecological niche. Among these, we identified two candidate new lineages related to type XI retrons that lack the protease domain typical of this group but instead carry distinct accessory proteins, expanding the known architectural diversity of these systems. These findings broaden the catalog of retron diversity available for functional characterization and biotechnological engineering and provide a framework for prioritizing candidate lineages for future experimental validation.

## INTRODUCTION

Retrons are widespread bacterial genetic elements that encode a specialized reverse transcriptase (RT) capable of producing multicopy single-stranded DNA (msDNA), a distinctive RNA–DNA hybrid molecule synthesized from a dedicated non-coding RNA (ncRNA) containing the msr and msd elements (1, 2). Originally discovered as producers of unusual DNA in myxobacteria and enteric bacteria, retrons were long regarded as molecular curiosities with unknown biological functions. This view changed dramatically with the discovery that many retrons function as components of bacterial anti-phage defense systems, acting as sensors that trigger abortive infection or toxin activation upon phage challenge (3–5). Researchers have recently co-opted retrons as programmable tools for genome editing and biosensing applications, further underscoring their biological and biotechnological relevance (6, 7).

The systematic classification of retron diversity was significantly advanced by Mestre et al. (8), who defined 13 canonical retron types (types I–XIII) based on RT phylogeny together with the architecture of associated ncRNA elements and accessory proteins, using a curated data set of 1,928 reference sequences spanning diverse bacterial lineages. This framework established that distinct retron types are characterized by specific msr/msd structures, recurrent accessory effector domains, and non-random taxonomic distributions, indicating that retron diversity has been shaped by both vertical inheritance and horizontal gene transfer across the microbial tree of life. Subsequent studies have expanded the catalog of retron-associated defense mechanisms, revealing unexpectedly rich functional diversity within this element family (3, 5, 9–11).

Despite these advances, our understanding of retron diversity remains heavily biased toward cultivated isolates and well-characterized organisms. Much of the microbial diversity present in environmental and host-associated microbiomes remains underrepresented in reference databases, limiting the discovery of divergent retron lineages and obscuring the full ecological breadth of these elements. Metagenomic approaches offer a powerful complement to culture-dependent surveys; however, the systematic application of retron detection pipelines to large-scale genome-resolved metagenomic resources has not yet been reported. Key questions, therefore, remain unresolved: how widespread are retron systems across the microbial biosphere? How are canonical retron types distributed across taxonomic groups and environments? To what extent does unexplored microbial diversity harbor previously unrecognized retron lineages?

The Searchable planetary-scale microbiome REsource (SPIRE) database provides an unprecedented resource for addressing these questions. SPIRE comprises 107,078 non-redundant representative genomes, including both metagenome-assembled genomes (MAGs) and isolate representatives, spanning aquatic, terrestrial, host-associated, and anthropogenic environments (12). By integrating genome-resolved assemblies with standardized taxonomic and ecological annotations, SPIRE enables systematic exploration of genetic element diversity across the global microbiome.

Here, we present a systematic survey of retron RT diversity across the SPIRE resource using a panel of type-specific hidden Markov model (HMM) profiles benchmarked against all canonical retron groups defined by Mestre et al. (8). We identified 1,286 retron candidates spanning all 13 canonical types, characterized their taxonomic and ecological distributions, and implemented a novelty prioritization framework to identify lineages with divergent architectures. This analysis revealed two candidate type XI-like lineages, TXI_C2like and TXI_noncan_h, within retron RT clade 8, distinguished by protease-independent architectures and distinct lineage-specific accessory modules. The *de novo* computational identification of candidate msr/msd-like ncRNA elements in both lineages further indicates that these highly divergent systems retain the core organizational framework of canonical retrons.

## MATERIALS AND METHODS

### Genome data set and protein prediction

A total of 107,078 representative microbial genomes from the SPIRE v01 data set (12) were analyzed, comprising one genome per species-level cluster defined at approximately 95% average nucleotide identity. Of these, 92,134 were metagenome-assembled genomes (MAGs), whereas the remaining 14,944 were isolate representatives derived from linked ProGenomes clusters. Protein-coding sequences were predicted *de novo* from all assemblies using Prodigal v2.6.3 (13) in metagenomic mode (-p meta), yielding approximately 272 million predicted proteins.

Taxonomic lineages (phylum to species) were assigned by mapping genome identifiers to their corresponding SPIRE species-level clusters, including both ProGenomes-linked specI_v4_* clusters and *de novo* 95% ANI clusters (spire_v1_095_*), using the SPIRE genome metadata file (spire_v1_genome_metadata.tsv). Full lineage strings were then reconstructed using taxonkit v0.14 (14) against the NCBI taxonomy database.

Ecological metadata were assigned by linking each candidate contig to its originating sample through the derived_from_sample field and querying the SPIRE microntology (spire_v1_microntology.tsv), a controlled vocabulary that provides structured, multi-tag environmental annotations for each sequenced sample. When multiple annotations were available for a given sample, the most specific descriptor was retained. Sample-level descriptors were subsequently collapsed into seven high-level environmental categories derived from the SPIRE microntology: host-associated, aquatic, terrestrial, anthropogenic, temperature-defined, salinity-defined, and pH-defined environments.

### HMM construction, candidate detection, and classification

A panel of 28 type-specific hidden Markov model (HMM) profiles was constructed to cover all 13 known retron types (types I–XIII and their recognized subgroups), as well as the clade 11 and clade2_Ec107like lineages. For each group, reference sequences from Mestre et al. (8) were aligned using MAFFT v7 (15), and profile HMMs were generated using hmmbuild from HMMER v3.3 (16).

Profiles were empirically cross-calibrated to define group-specific classification thresholds. For each HMM, two score distributions were calculated: (i) self-hit scores, obtained by searching the profile against its own reference sequences and recording the minimum score (pos_min) and (ii) cross-hit scores, obtained by searching the profile against all reference sequences from the remaining groups and recording the maximum score (neg_max). The classification threshold for each group was defined as the midpoint between these values, calculated as (pos_min + neg_max)/2. Thresholds ranged from 177.2 bits for type I-B2 to 317.5 bits for clade11-A (Table S1).

The approximately 272 million predicted proteins were first screened using hmmsearch (HMMER v3.3) against a general retron RT HMM constructed from the 1,928 reference RT sequences reported by Mestre et al. (8). Searches were performed with an *E*-value cutoff of 1 × 10^−10^, yielding 31,280 initial hits. Sequences with bit scores ≥ 160 bits were retained as candidate retron RTs (*n* = 1,319).

The 160-bit cutoff was selected empirically based on the score distribution of the 1,928 reference RTs against the general HMM. The lowest-scoring reference sequence yielded 114.5 bits, corresponding to a highly divergent type II RT. At the 160-bit threshold, 195 reference sequences (10.1%) fell below the cutoff, predominantly from type VI (*n* = 91), type I-B2 (*n* = 36), type I-B1 (*n* = 34), and type IV (*n* = 20). This threshold, therefore, represents a conservative trade-off between sensitivity and specificity and likely results in underrepresentation of the most divergent retron lineages.

Among the 1,319 candidates, 33 sequences carried contig identifiers in k* format that lacked assembly sequences, gene annotations, and associated metadata; these were excluded from downstream biological analyses but were retained for phylogenetic placement to preserve overall tree context.

The remaining 1,286 SPIRE-native candidates were classified by scoring each sequence against the 28 type-specific HMMs using hmmsearch. Each candidate was assigned to the group producing the highest bit score. The overall computational workflow used for retron identification, classification, and novelty assessment is summarized in Fig. S1.

Confidence tiers were assigned based on three independent criteria: (i) HMM detection strength, assessed relative to the group-specific classification threshold; (ii) RT domain completeness based on conservation of the canonical RT1–RT7 motif core; and (iii) consistency of phylogenetic placement with established retron clades using EPA-ng v0.3.8 (17). High-confidence candidates exceeded the group-specific threshold, displayed near-complete RT1–RT7 architecture, and showed coherent phylogenetic placement within or immediately adjacent to established retron clades. Low-confidence candidates showed detectable but weaker support (bit score ≥160 bits but below the group-specific threshold) and/or partial RT domain coverage, with broadly compatible but less well-supported phylogenetic placement. Borderline candidates had scores near the overlap zone between self-hit and cross-hit distributions. These were retained for completeness and included in genomic neighborhood analyses but excluded from high-confidence biological interpretations.

### Phylogenetic reference tree and placement of SPIRE candidates

A maximum-likelihood reference tree was inferred from the 1,928 retron RT sequences compiled by Mestre et al. (8). Reference sequences were aligned using MAFFT v7 (15), and the resulting alignment was trimmed to the 203 positions corresponding to the conserved RT1–RT7 motif core using a custom Python script that removes columns containing more than 50% gaps. The trimmed alignment was used to infer a maximum-likelihood tree with IQ-TREE 2 v2.3.2 (18). The best-fitting substitution model was selected using ModelFinder and corresponded to Q.pfam + F + I + G4. Branch support was assessed with 1,000 ultrafast bootstrap replicates.

SPIRE candidate RTs were incorporated into the reference alignment using MAFFT with the --add option (19), preserving the original alignment coordinates. Phylogenetic placement was performed using EPA-ng v0.3.8 (17). For each candidate, the placement with the highest likelihood weight ratio (LWR) was retained. Placements were then grafted onto the reference topology using Gappa (20), and the resulting combined tree was visualized and annotated in Interactive Tree Of Life (iTOL) v6 (21).

### Covariance model searches for msr/msd detection

The presence of msr/msd non-coding RNA elements in the genomic neighborhood of each RT candidate was assessed using cmsearch from Infernal v1.1 (22) with a panel of 21 covariance models derived from msr/msd transcripts representing retron types for which structural information is available (8). Searches were performed against genomic regions extending 10 kb upstream and downstream of each RT locus extracted from the SPIRE genome assemblies.

Covariance model searches were applied to all high-confidence candidates (*n* = 934), of which 540 (57.8%) yielded a confident msr/msd assignment. The most frequently detected models corresponded to Ec107_like, clade11-A, TypeIC1_IC2, TypeIA_IIAI, and clade11-B. Retron types lacking experimentally validated msr/msd structures, including types VI, VII, VIII, X, XI, XII, III-A4, and III-A5, were not represented in the covariance model panel and therefore could not be annotated by this analysis. Accordingly, the absence of a covariance model hit in these groups should not be interpreted as evidence for the absence of an associated ncRNA, but rather as a consequence of the lack of a validated covariance model for these more divergent, less-studied lineages (msr/msd detection: 73.0% in groups with a validated model vs 26.3% in groups lacking one). For the clade 11 lineage, two distinct msr/msd structural families, designated A and B by Mestre et al. (8), were used to subdivide candidates into clade11-A (model A detected; *n* = 71), clade11-B (model B detected; *n* = 39), and a residual clade11 (no confident hit to either model; *n* = 9).

### *De novo* identification of candidates ncRNA structures

For *de novo* discovery of candidate retron ncRNAs, the 600 bp region immediately upstream of the RT start codon was extracted from all TXI_noncan_h (*n* = 28) and TXI_C2like (*n* = 120) loci using custom Python scripts. Conserved RNA secondary structures were initially identified using CMfinder 0.4 (23) with the parameters -f 0.5 -s1 2 -s2 3 -minspan1 80 -maxspan1 300 -minspan2 80 -maxspan2 300 -combine.

Covariance models were constructed using cmbuild and calibrated with cmcalibrate from Infernal v1.1.5 (22). Positional validation was performed by searching the resulting models against genomic regions extending 10 kb upstream and downstream of each RT locus using cmsearch with an *E*-value threshold of 0.01.

Structure-guided multiple sequence alignments were generated from the 300 bp immediately upstream of the RT start codon using mLocARNA 2.0.1 (24) with the parameters --plfold-span 400 --min-prob 0.03 --indel −4 --indel-open −400 --max-diff 100 --max-diff-am −60 --alifold-cons.

Covariation significance above phylogenetic expectation was assessed using R-scape 2.0.4 (25) with an *E*-value threshold of 0.05.

### Genomic neighborhood analysis

Coding sequences neighboring each RT candidate were identified from the Prodigal gene annotations (GFF format) associated with each SPIRE genome. For each RT locus, genes located on the same contig were ordered according to genomic coordinates, and neighboring coding sequences were extracted relative to the RT position using custom Python scripts.

A broad ±5 gene window was initially used to capture the local genomic context and provided complete coverage for all 1,286 SPIRE-native candidates. For accessory protein analyses, a more stringent ±2 gene window, excluding the RT itself, was used to define tight genomic neighborhoods. This approach retained complete neighborhood information for 1,284 of 1,286 loci while minimizing the inclusion of unrelated genes in gene-dense regions.

Protein sequences from these tight neighborhoods were clustered by sequence similarity using MMseqs2 (26) with thresholds of at least 30% amino acid identity and 80% alignment coverage. Cluster representatives were functionally annotated using hmmscan from HMMER v3.3 against Pfam-A v35.0 (27) with an *E*-value cutoff of 1 × 10^−5^.

In parallel, cluster representatives were screened against a curated collection of HMMs representing known retron-associated effector families (8) using the same significance threshold. This complementary analysis enabled the detection of functionally relevant accessory modules that were not always fully captured by general Pfam annotations.

For clusters lacking confident Pfam or effector assignments, structural homology searches were performed using HHsearch (28) against the PDB70 database. Candidate matches were evaluated based on HHsearch probability scores and alignment quality. For a subset of accessory protein clusters, three-dimensional structures were predicted using AlphaFold2 (29) as implemented in ColabFold (30), and the resulting models were compared against the PDB100 and AlphaFold/UniProt (AF-SwissProt) structural databases using Foldseek (31; easy-search mode, default parameters).

### Taxonomic annotation, ecological classification, and statistical analyses

Non-random associations between retron groups and bacterial phyla, and between retron groups and ecological biomes, were assessed using Pearson’s chi-squared test of independence applied to contingency tables of group × phylum and group × biome, respectively, retaining only groups with *n* ≥ 10 and phyla or biomes with *n* ≥ 5. Chi-squared statistics and *P*-values were computed using chi2_contingency (SciPy v1.11 [32]). Pearson standardized residuals were calculated as (*O* − *E*)/√*E*; |residuals| exceeding 2 were considered indicative of notable enrichment or depletion. Taxonomic breadth of each retron group was quantified using the Gini–Simpson diversity index (1 − *D*), where *D* = Σ*pᵢ*² and *pᵢ* is the proportion of candidates from phylum *i*; values of 0 and 1 indicate complete restriction to a single phylum and maximum evenness across phyla, respectively. To further resolve within-phylum patterns, an analogous chi-squared framework was applied to a group × taxonomic order contingency table (order *n* ≥ 5, group *n* ≥ 10), yielding standardized Pearson residuals at the order rank.

Statistical associations between accessory protein clusters and retron groups were quantified using two-sided Fisher’s exact test, comparing cluster frequency within each target group against all remaining groups. Odds ratios were computed as (*a* × *d*)/(*b* × *c*); clusters exclusively present in one group received OR = ∞. Multiple testing correction used the Benjamini–Hochberg false discovery rate procedure (33), implemented via SciPy v1.11 (32). Clusters with FDR <0.05 and OR >2 were considered significantly enriched. To rule out general phylum-level abundance as an explanation, WYL and DnaB_C frequencies were additionally compared against a phylum-matched genomic background (300 randomly sampled Bacteroidota genomes). All statistical analyses were implemented in Python v3.11 using NumPy v1.24, SciPy v1.11, and pandas v2.0.

## RESULTS

### Global survey of retron diversity across the SPIRE genome resource

To systematically identify retron reverse transcriptases (RTs) across the SPIRE genome resource, we screened the predicted proteomes of 107,078 representative genomes using a general retron RT HMM and classified all hits with a panel of 28 cross-calibrated type-specific HMM profiles. This approach yielded 31,280 initial RT-like hits, of which 1,319 exceeded the general score threshold. After excluding 33 candidates lacking associated genomic context, a total of 1,286 SPIRE-native candidates were retained for downstream analyses. These candidates were distributed across 1,187 unique genomes, indicating that retrons are detectable in approximately 1.1% of the species-level representatives included in SPIRE (Table S2).

**Fig 1:**
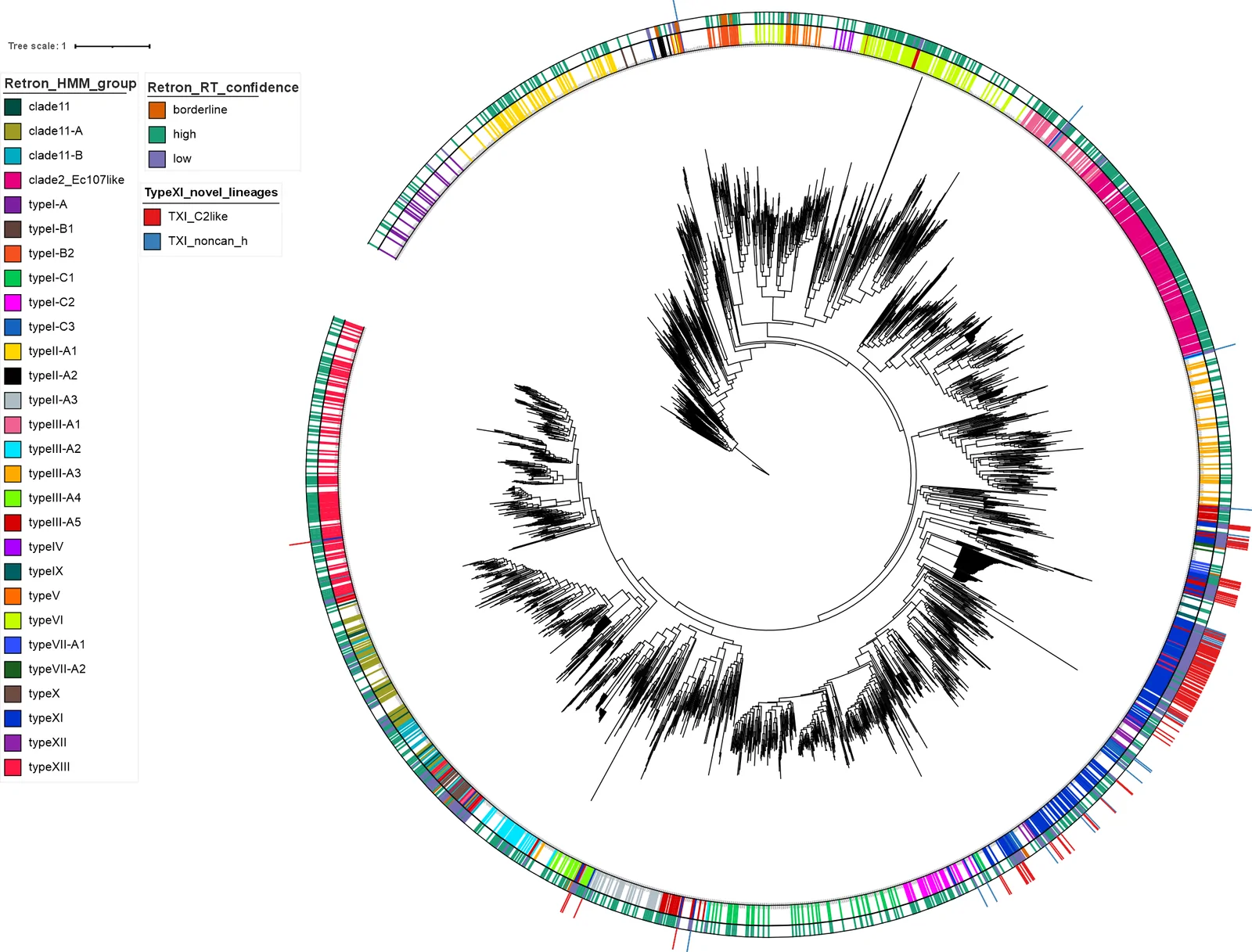
Phylogenetic placement of SPIRE-derived retron RTs in the global retron landscape. Circular maximum-likelihood phylogeny of retron reverse transcriptases (RTs) inferred from a curated reference data set of 1,928 sequences compiled by Mestre et al. (8). RT candidates identified in the SPIRE genome resource were incorporated into this framework by phylogenetic placement using EPA-ng v0.3.8 (17). The tree is shown using the original rooting of the reference phylogeny. The complete phylogenetic tree is provided as Data S1. Three concentric annotation rings summarize the main attributes of SPIRE-derived candidates. The innermost ring (Retron_HMM_group) indicates the HMM-based classification of each sequence into established retron groups and subgroups. The middle ring (Retron_RT_confidence) denotes the confidence tier assigned to each candidate (high, borderline, or low) based on HMM support, RT motif conservation, and phylogenetic consistency. The outermost ring (TypeXI_novel_lineages) highlights sequences belonging to the two noncanonical type XI-like lineages identified in this study, designated TXI_C2like and TXI_noncan_h, which are analyzed in detail in Figs. 4 and 5. SPIRE-derived RTs are distributed across the full breadth of the reference retron phylogeny, substantially expanding previously defined clades. Most candidates assigned to the novel Type XI-like lineages map within the reference Type XI region, although a small number of outlying placements are observed, likely reflecting uncertainty in the placement of highly divergent RT core sequences. Overall, the distribution of SPIRE candidates reveals extensive hidden retron diversity across known groups and highlights two previously unrecognized Type XI-associated radiations.

Candidates were assigned to confidence tiers based on HMM support, RT domain completeness, and phylogenetic consistency. Of the 1,286 candidates, 934 were classified as high-confidence (72.6%), 25 as borderline (1.9%), and 327 as low-confidence (25.4%). Phylogenetic placement using EPA-ng v0.3.8 (17) within a maximum-likelihood reference tree built from 1,928 curated retron RTs confirmed that all candidates fell within established retron phylogenetic diversity (Fig. 1).

**Fig 2:**
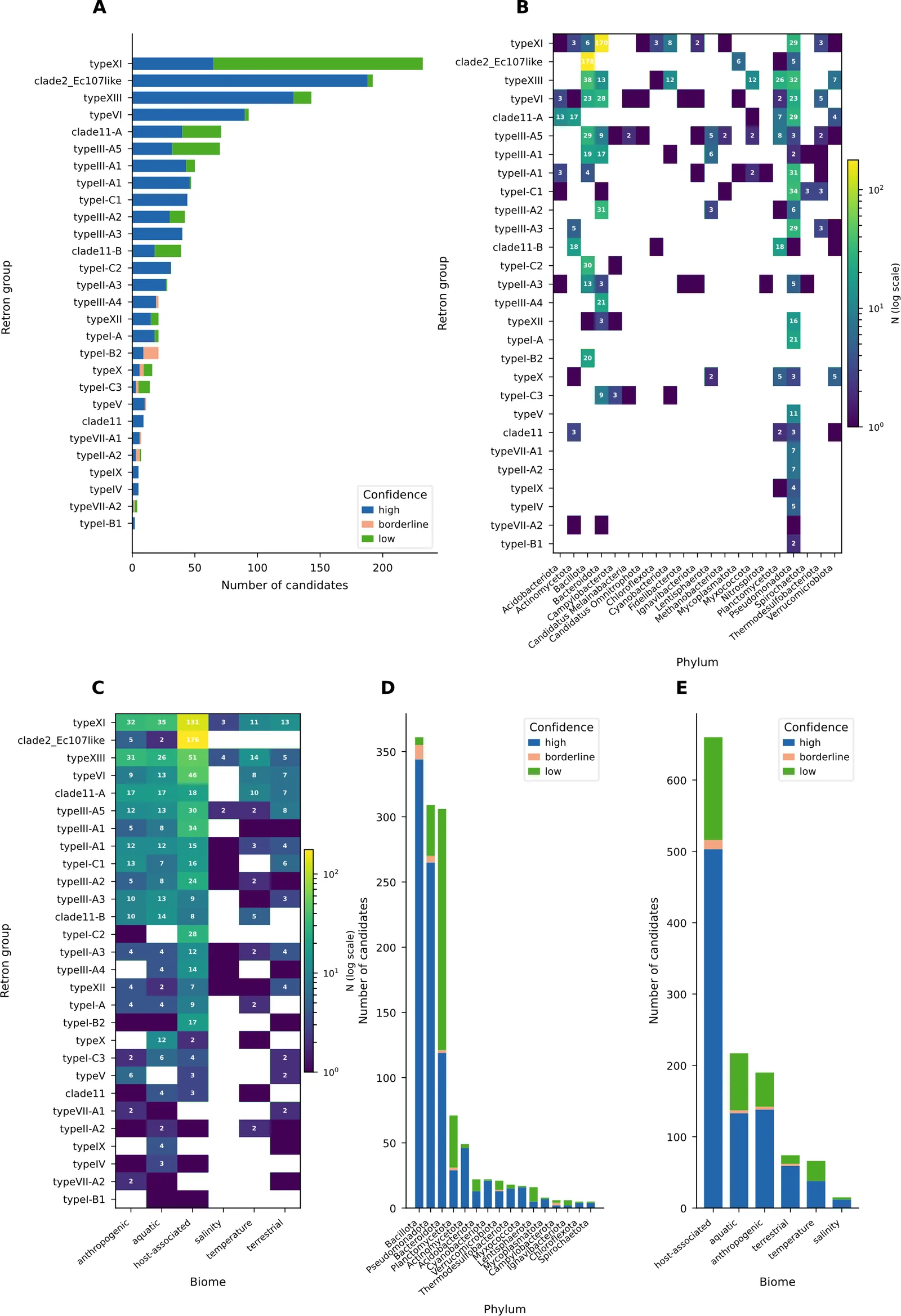
Global distribution of SPIRE-derived retron candidates across retron groups, bacterial phyla, and ecological biomes. (**A**) Distribution of retron candidates by classification group and hidden Markov model (HMM) confidence tier. Type XI-like retrons (*n* = 232) were the most abundant group but were dominated by low-confidence candidates (*n* = 167, 72%), in contrast to clade2_Ec107like (*n* = 192), which was almost entirely composed of high-confidence candidates (*n* = 188, 98%). Type XIII (*n* = 143), type VI (*n* = 93), and clade11-A (*n* = 71) were the next most abundant groups. Groups with notable borderline fractions included type I-B2 and type III-A4. (**B**) Distribution of retron groups across bacterial phyla (color scale indicates the log-transformed number of candidates). Type XI-like retrons were concentrated in Bacteroidota (170 of 232 candidates), whereas clade2_Ec107like was predominantly associated with Bacillota (178 of 192 members). Type XIII showed the broadest phylogenetic distribution, spanning Bacillota (*n* = 38), Pseudomonadota (*n* = 32), Planctomycetota (*n* = 26), and Bacteroidota (*n* = 13). Type VI was distributed across Bacteroidota (*n* = 28), Bacillota (*n* = 23), and Pseudomonadota (*n* = 23). Clade11-A was enriched in Pseudomonadota (*n* = 29), with additional representation in Actinomycetota (*n* = 17) and Acidobacteriota (*n* = 13). (**C**) Distribution of retron groups across high-level environmental categories (color scale indicates the log-transformed number of candidates). clade2_Ec107like showed the strongest association with host-associated environments (176 of 192 candidates, 91%). Type XI-like retrons were also predominantly assigned to host-associated environments (131 of 232 candidates, 56%) but were additionally detected in aquatic (*n* = 35) and anthropogenic (*n* = 32) categories. Type XIII displayed a broad environmental distribution, whereas type X was predominantly associated with aquatic environments (12 of 16 candidates). (**D**) Global phylum distribution stratified by HMM confidence tier. Bacillota and Pseudomonadota were dominated by high-confidence candidates (95% and 86%, respectively), consistent with the prevalence of well-characterized retron groups in these phyla. In contrast, Bacteroidota showed the lowest proportion of high-confidence candidates (39%), driven by the large contribution of low-confidence type XI-like retrons. Planctomycetota also exhibited an elevated low-confidence fraction (56%). (**E**) Global distribution of retron candidates across high-level environmental categories stratified by HMM confidence tier. Host-associated environments contained the largest number of retron candidates (*n* = 660, 51.3%), of which 76% were classified as high confidence. Aquatic and temperature-defined categories showed elevated low-confidence fractions (39% and 42%, respectively), consistent with the increased representation of type XI-like retrons in these categories.

All 13 canonical retron types described by Mestre et al. (8) were detected, together with the clade11 and clade2_Ec107like lineages, yielding 28 classification groups (Fig. 2A through E, Table 1; Table S1). The most abundant group was type XI-like (*n* = 232, 18.0%) although 72% of these candidates were assigned to the low-confidence tier, well above the dataset-wide average of 25.4%, indicating substantial sequence divergence within this lineage. The second most abundant group was clade2_Ec107like (*n* = 192, 14.9%), of which 98% were high-confidence, enabling clear separation from its sister lineage, type III-A1. Type XIII (*n* = 143, 11.1%) and type VI (*n* = 93, 7.2%) were the third and fourth most abundant groups, respectively, and were predominantly composed of high-confidence candidates. Each of the remaining 24 groups accounted for less than 6% of the total candidate set.

**TABLE 1:** Summary of retron candidates identified across the SPIRE microbial resource^*a*^

Retron group	*N* total	*N* high	*N* borderline	*N* low	% high-conf	Dominant phylum	Dominant biome	Top accessory module	msr/msd model	Review classification	Novelty divergence
typeXI	232	65	0	167	28%	Bacteroidota (73%)	Host-associated (56%)	WYL (*n* = 26, TXI_C2like)/DnaB_C (*n* = 16, TXI_noncan_h)	–	Two candidate novel lineages (TXI_C2like, TXI_noncan_h)	**HIGH**
clade2_Ec107like	192	188	0	4	98%	Bacillota (93%)	Host-associated (92%)	PDH_N (*n* = 8)	Ec107_like	Known-like/minimal expansion	*LOW*
typeXIII	143	129	0	14	90%	Bacillota (27%)	Host-associated (36%)	SWIM (*n* = 50)	TypeXIIIA_firmi/B_Ne144 /C_Mx162/Mx65	Known-type expansion	**MEDIUM**
typeVI	93	90	0	3	97%	Bacteroidota (30%)	Host-associated (49%)	HTH_3 (*n* = 21)	–	Known-type expansion	NONE
clade11-A	71	40	0	31	56%	Pseudomonadota (41%)	Host-associated (25%)	–	OutgroupA	Heterogeneous/low priority	NONE
typeIII-A5	70	32	0	38	46%	Bacillota (41%)	Host-associated (43%)	PRTase-CE_4 (*n* = 10)	–	Known-type expansion	*LOW*
typeIII-A1	50	43	0	7	86%	Bacillota (38%)	Host-associated (68%)	PRTase-CE (*n* = 18)	TypeIIIA1	Known-type expansion	NONE
typeII-A1	47	46	0	1	98%	Pseudomonadota (66%)	Host-associated (32%)	STM3845_2 (*n* = 12)	–	Known-type expansion	NONE
typeI-C1	44	44	0	0	100%	Pseudomonadota (77%)	Host-associated (36%)	Resolvase (*n* = 9)	TypeIC1_IC2	Known-type expansion	NONE
typeIII-A2	42	30	0	12	71%	Bacteroidota (74%)	Host-associated (57%)	DUF5686 (*n* = 10)	TypeIIIA2	Known-type expansion	NONE
typeIII-A3	40	40	0	0	100%	Pseudomonadota (72%)	Aquatic (32%)	PRTase-CE_3 (*n* = 12)	TypeIIIA3	Known-type expansion	NONE
clade11-B	39	18	0	21	46%	Actinomycetota (46%)	Aquatic (36%)	–	OutgroupB	Heterogeneous/low priority	NONE
typeI-C2	31	31	0	0	100%	Bacillota (97%)	Host-associated (90%)	–	–	Known-type expansion	NONE
typeII-A3	28	27	0	1	96%	Bacillota (46%)	Host-associated (43%)	STM3845 (*n* = 27)	TypeIIA3_firmi/proteo	Known-type expansion	NONE
typeIII-A4	21	19	2	0	90%	Bacteroidota (100%)	Host-associated (67%)	PRTase-CE_2 (*n* = 12)	–	Known-type expansion	NONE
typeXII	21	15	0	6	71%	Pseudomonadota (76%)	Host-associated (33%)	–	–	Known-type expansion	NONE
typeI-A	21	18	0	3	86%	Pseudomonadota (100%)	Host-associated (43%)	SMC_N (*n* = 14)	TypeIA_IIAI	Known-type expansion	NONE
typeI-B2	21	9	12	0	43%	Bacillota (95%)	Host-associated (81%)	AAA_21 (*n* = 14)	TypeIB2_firmi/proteo	Divergent subgroup	**MEDIUM**
typeX	16	6	3	7	38%	Planctomycetota (31%)	Aquatic (75%)	–	–	Low-count known group	NONE
typeI-C3	14	3	2	9	21%	Bacteroidota (64%)	Aquatic (43%)	–	–	Low-count known group	NONE
typeV	11	10	1	0	91%	Pseudomonadota (100%)	Anthropogenic (55%)	CSD (*n* = 8)	TypeV	Low-count known group	NONE
clade11	9	9	0	0	100%	Actinomycetota (33%)	Aquatic (44%)	–	–	Known-type expansion	NONE
typeVII-A1	7	6	1	0	86%	Pseudomonadota (100%)	Anthropogenic (29%)	–	–	Known-type expansion	NONE
typeII-A2	7	3	3	1	43%	Pseudomonadota (100%)	Aquatic (29%)	–	TypeIIA2	Known-type expansion	NONE
typeIX	5	5	0	0	100%	Pseudomonadota (80%)	Aquatic (80%)	–	TypeIX	Known-type expansion	NONE
typeIV	5	5	0	0	100%	Pseudomonadota (100%)	Aquatic (60%)	–	TypeIV	Known-type expansion	NONE
typeVII-A2	4	1	1	2	25%	Pseudomonadota (25%)	Anthropogenic (50%)	–	–	Known-type expansion	NONE
typeI-B1	2	2	0	0	100%	Pseudomonadota (100%)	Aquatic (50%)	–	TypeIB1	Known-type expansion	NONE
Total	1,286	934	25	327							

^*a*^ Top accessory module, largest recurrent accessory cluster within ±2-gene window (MMseqs2 ≥ 30% identity). Novelty divergence: NONE, known expansion (plain text); LOW, minimal divergence (italics); MEDIUM, divergent subgroup (bold); HIGH, candidate novel lineage (bold, underlined). Notes: msr/msd detection performed on high-confidence candidates using 21 covariance models (Infernal v1.1) (8). TypeXI msr/msd not detected with published models; candidate ncRNA structures identified by *de novo* discovery in this study (see Fig. 6). firmi/proteo, phylum-specific msr/msd covariance model variants, calibrated separately on Firmicutes (Bacillota) and Proteobacteria (Pseudomonadota) representatives, following the msr/msd structural families defined by Mestre et al. (8).

Within clade11, covariance model searches using the two known msr/msd structural families defined by Mestre et al. (8) resolved three subgroups: clade11-A (*n* = 71), clade11-B (*n* = 39), and a residual clade11 (*n* = 9), in which neither covariance model yielded a confident hit.

Retrons were detected across 13 bacterial phyla (Fig. 2B). Retron type–phylum associations were highly significant (*χ*² = 2,764.8, df = 375, *P* < 10⁻³⁰⁰), and Pearson standardized residuals highlighted strong lineage-specific enrichments and depletions across phyla (Fig. S2A). The most represented phyla were Bacillota (*n* = 361), Pseudomonadota (*n* = 309), and Bacteroidota (*n* = 306), which together accounted for 75.9% of all candidates. However, confidence distributions differed markedly among these phyla. Bacillota and Pseudomonadota were dominated by high-confidence candidates (95% and 86%, respectively), reflecting the prevalence of well-characterized retron groups. By contrast, Bacteroidota showed a substantially lower high-confidence fraction (39%), driven by the predominance of type XI-like candidates. Indeed, 170 of the 232 type XI-like retrons were identified in Bacteroidota, where they represented the dominant retron lineage.

To determine what underlies these confidence differences, we examined bitscore distributions and phylum composition for the four groups with the largest low-confidence fractions: type XI-like, clade11-A, clade11-B, and type III-A5. Motif-level analysis of the RT1–RT7 core showed that most low-confidence type XI-like candidates remained motif-compatible retron RTs (143 of 167, 85.6%), indicating that reduced confidence in this group reflects sequence divergence rather than the absence of retron identity. Low-confidence type XI-like candidates were overwhelmingly concentrated within Bacteroidota (156 of 167, 93.4%), consistent with the reduced high-confidence fraction observed for this phylum (Fig. 2D, 39%). However, this pattern was group-specific rather than general: low-confidence candidates were concentrated in Pseudomonadota for clade11-A (52%) and in Planctomycetota for clade11-B (86%), whereas type III-A5 candidates were more evenly distributed across phyla. Reduced classification confidence in each group, therefore, reflects sequence divergence within specific, typically underrepresented phyla, rather than a uniform Bacteroidota effect.

Quantitative analysis of taxonomic specificity using the Gini-Simpson diversity index (1 − *D*) revealed a continuum ranging from highly restricted to broadly distributed lineages (Fig. S2B). The most taxonomically restricted groups were clade2_Ec107like (1 − *D* = 0.139, 93% Bacillota), type I-C2 (1 − *D* = 0.062, 97% Bacillota), and type III-A4 (1 − *D* = 0, exclusively Bacteroidota). In contrast, type XIII (1 − *D* = 0.821), type VI (1 − *D* = 0.782), and type III-A5 (1 − *D* = 0.788) exhibited the broadest taxonomic distributions.

To resolve these phylum-level associations at finer taxonomic resolution, we extended the analysis to the order rank (order *n* ≥ 5, group *n* ≥ 10; 898 of 1,286 candidates, 21 groups × 35 orders). A chi-squared test of independence confirmed a highly significant non-random association between retron group and taxonomic order (*χ*² = 3,438.3, df = 680, *P* < 10⁻³⁰⁰), and standardized Pearson residuals identified the specific orders underlying each group’s phylum-level association (Fig. S3; Table S3). Type XI-like retrons were most strongly enriched within the order Bacteroidales (113 of 232 candidates; residual = 13.2), refining the phylum-level Bacteroidota association reported above to a specific bacterial order. clade2_Ec107like was enriched within Eubacteriales (83 of 192; residual = 10.8) and Lachnospirales (63 of 192; residual = 8.6), both orders within Bacillota. Among the less abundant groups, clade11-B showed the strongest order-level enrichment observed in the data set, within Mycobacteriales (18 of 39; residual = 21.8), and clade11-A was enriched within Micrococcales and Bryobacterales.

Retron-containing genomes were most frequently assigned to the host-associated category (*n* = 660, 51.3%), followed by aquatic (*n* = 217, 16.9%) and anthropogenic (*n* = 190, 14.8%) environments (Fig. 2C and E). This pattern was driven primarily by clade2_Ec107like, of which 91% of candidates were assigned to the host-associated category, and by type XI-like retrons, for which 56% of candidates received the same annotation. Together, these two groups accounted for nearly half of all host-associated candidates. In contrast, type X was most frequently assigned to aquatic environments (75%), whereas type V was most commonly associated with anthropogenic environments (55%). Retron type–biome associations were also highly significant (*χ*² = 453.3, df = 125, *P* = 9.2 × 10^−39^; Fig. 2C), demonstrating that ecological specialization is a pervasive feature of retron diversity.

Host-associated environments accounted for the largest single category of retron-containing genomes. To resolve this host association further, we examined the finer-resolution SPIRE biome ontology available for each host-associated candidate (*n* = 660). These partitioned into animal digestive tract (unspecified/non-human, 45.3%), explicitly human host (33.3%), rumen/likely livestock host (10.0%), and plant-associated environments, mainly rhizosphere (5.6%) (Table S4). Human-host candidates were dominated by clade2_Ec107like (*n* = 65), type XI-like (*n* = 33), and type VI (*n* = 20) and were most frequent in Bacillota (*n* = 124) and Bacteroidota (*n* = 53). Notably, type XI-like retrons’ broad digestive-tract association split almost evenly between human (*n* = 33) and rumen (*n* = 36) hosts, consistent with a broad mammalian gut association rather than a human-specific one. For Bacillota specifically, retron-containing genomes were concentrated in the orders Eubacteriales (*n* = 151) and Lachnospirales (*n* = 125), with the leading genera being *Ruminococcus*, *Clostridium*, *Oscillibacter*, *Eubacterium*, and *Blautia,* well-established commensal genera of the class Clostridia that are common members of the mammalian and human gut microbiota.

Together, these results establish that retrons are broadly distributed across the bacterial domain but exhibit pronounced lineage-specific patterns of taxonomic and ecological specialization. In particular, the concentration of type XI-like retrons in Bacteroidota and the strong host association of clade2_Ec107like suggest that both evolutionary history and environmental context have played major roles in shaping retron diversification.

### Most SPIRE retrons represent expansions of known lineages

To distinguish expanded diversity from genuine biological novelty, we systematically evaluated all 28 retron classification groups using five complementary criteria. (i) RT phylogenetic coherence assessed whether candidates assigned to a given group formed a discrete and well-supported cluster in both the EPA-ng v0.3.8 placement tree (Fig. 1) and an independent maximum-likelihood reconstruction. (ii) Recurrence and specificity of accessory protein architecture evaluated whether one or more non-RT modules were consistently associated with the group and statistically enriched relative to other retron groups. (iii) Confidence class distribution reflected the proportion of high-, borderline-, and low-confidence candidates, providing a measure of how closely each group conformed to the calibrated HMM profiles. (iv) Taxonomic coherence assessed the extent to which a group was restricted to specific bacterial lineages. (v) Ecological coherence assessed whether candidates showed non-random enrichment in particular biomes beyond that expected from host taxonomy alone. Each criterion was scored as absent (−), weak (+), moderate (++), or strong (+++), and the combined evidence was used to assign an overall novelty divergence score of none, low, medium, or high (Table S5).

**Fig 3:**
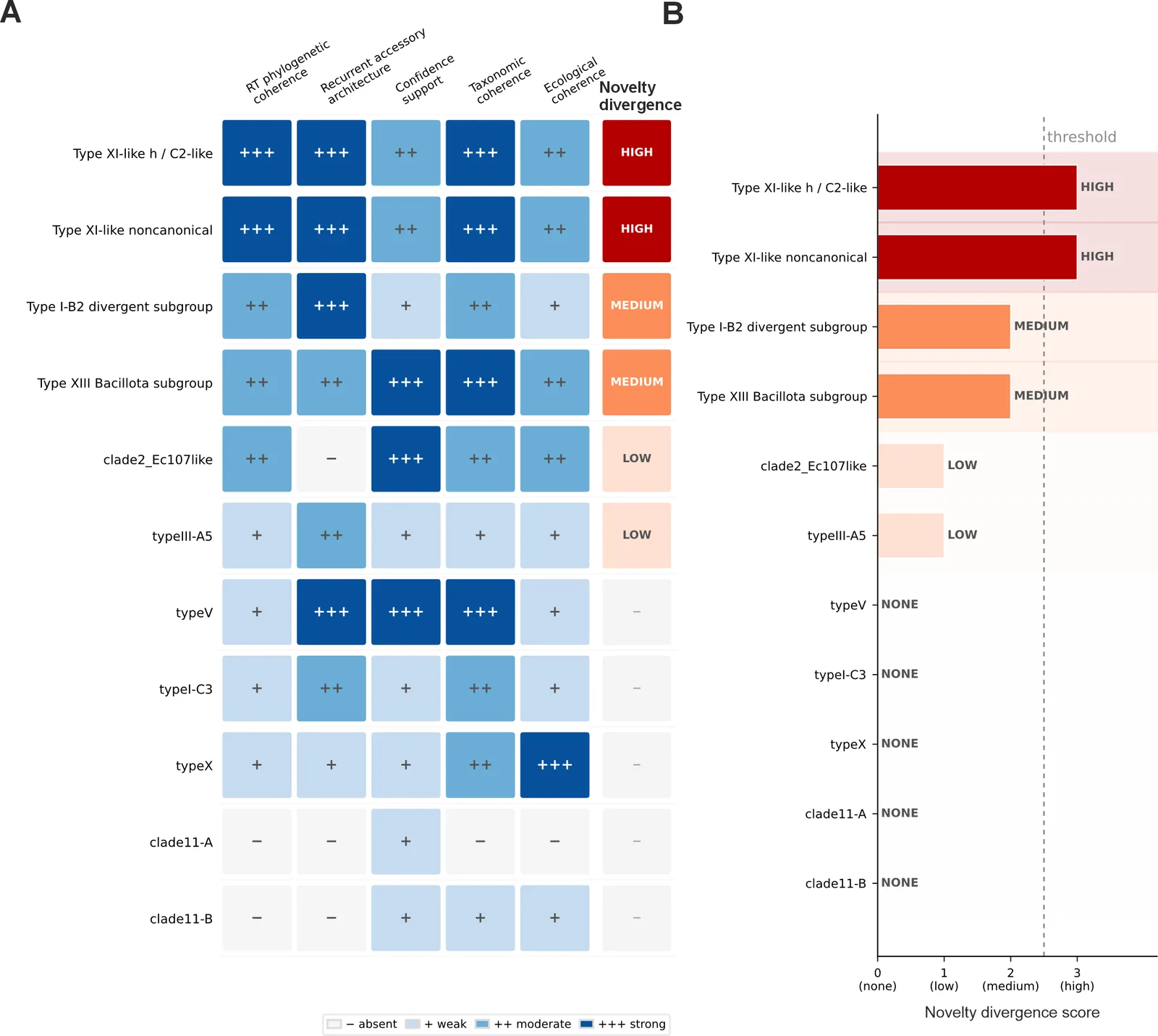
Systematic evaluation of SPIRE retron groups and novelty divergence scoring. (**A**) Curated evidence matrix summarizing the five criteria used to evaluate representative SPIRE retron lineages. Columns correspond to RT phylogenetic coherence, recurrence and specificity of accessory protein architecture, confidence class distribution, taxonomic coherence, and ecological coherence. Each criterion was scored on a four-point scale: absent (−), weak (+), moderate (++), or strong (+++). The composite novelty divergence score (none/low/medium/high) is shown in the rightmost column. TXI_C2like and TXI_noncan_h were the only lineages assigned strong (+++) scores for RT phylogenetic coherence, recurrent accessory architecture, and taxonomic coherence simultaneously and were prioritized as the strongest candidates for previously unrecognized retron lineages. In contrast, clade2_Ec107like showed strong (+++) confidence support and moderate (++) taxonomic coherence but was scored absent (−) for recurrent accessory architecture, reflecting the lack of non-RT accessory modules in this lineage. (**B**) Composite novelty divergence scores assigned to evaluated lineages after integration of all five criteria. TXI_C2like and TXI_noncan_h were the only lineages assigned a high novelty divergence score. The type XIII *Bacillota* subgroup and type I-B2 divergent subgroup reached a medium novelty divergence score. All remaining lineages received a low or no novelty score.

The results are summarized in a curated evidence matrix highlighting representative lineages that span the full spectrum of observed patterns, from canonical expansions and divergent subgroups to candidate novel lineages (Fig. 3A and B). The analysis was performed at the level of curated biological lineages rather than the original HMM classification groups. Groups scoring uniformly canonical (absent or weak divergence) across all five criteria were omitted from the matrix, as they provided limited discriminatory value for distinguishing degrees of novelty among groups.

Overall, 21 of the 28 retron classification groups were interpreted as expansions of previously described retron lineages. Three additional groups (types X, I-C3, and V) were classified as low-count representatives of known retron types. Clade2_Ec107like was considered a minimally divergent expansion of a previously described lineage, whereas clade11-A and clade11-B were analyzed separately but assigned low novelty divergence score because they lacked consistent distinguishing features. Only the type XI-like classification group, which belongs to RT Clade 8, yielded lineages with strong evidence of genuine biological novelty. Detailed analysis of its internal phylogenetic and architectural structure identified two candidate novel sublineages, TXI_C2like and TXI_noncan_h (Fig. 3A and B).

Several contrasting examples illustrate the framework’s discriminatory power. Type V showed strong accessory architecture (+++) and high confidence support (+++), yet was assigned no novelty divergence score because its organization fully conformed to the canonical cold-shock DNA-binding domain (CSD)-associated type V architecture, demonstrating that strong individual signals do not necessarily indicate biological novelty. Type I-B2 and the Bacillota-specific type XIII subgroup were assigned a medium novelty divergence score as divergent subgroups within the established retron types. Type I-B2 combined moderate phylogenetic divergence with a distinctive ATPase–TOPRIM accessory module, whereas the Bacillota type XIII subgroup exhibited restricted taxonomic distribution and recurrent SWIM_2/DUF6849 remodeling consistent with clade-level diversification.

In contrast, clade11-A, clade11-B, type III-A5, type X, and type I-C3 displayed weak or inconsistent evidence and were assigned low or no novelty divergence score. Notably, clade2_Ec107like received low novelty divergence score despite its abundance (*n* = 192), consistent with its canonical phylogenetic placement and the absence of recurrent non-RT accessory modules.

Collectively, this systematic evaluation identified TXI_C2like and TXI_noncan_h as the strongest candidates for previously unrecognized retron biology, motivating their detailed characterization in the following sections.

### Type XI non-canonical lineages encode two distinct stand-alone accessory architectures

**Fig 4:**
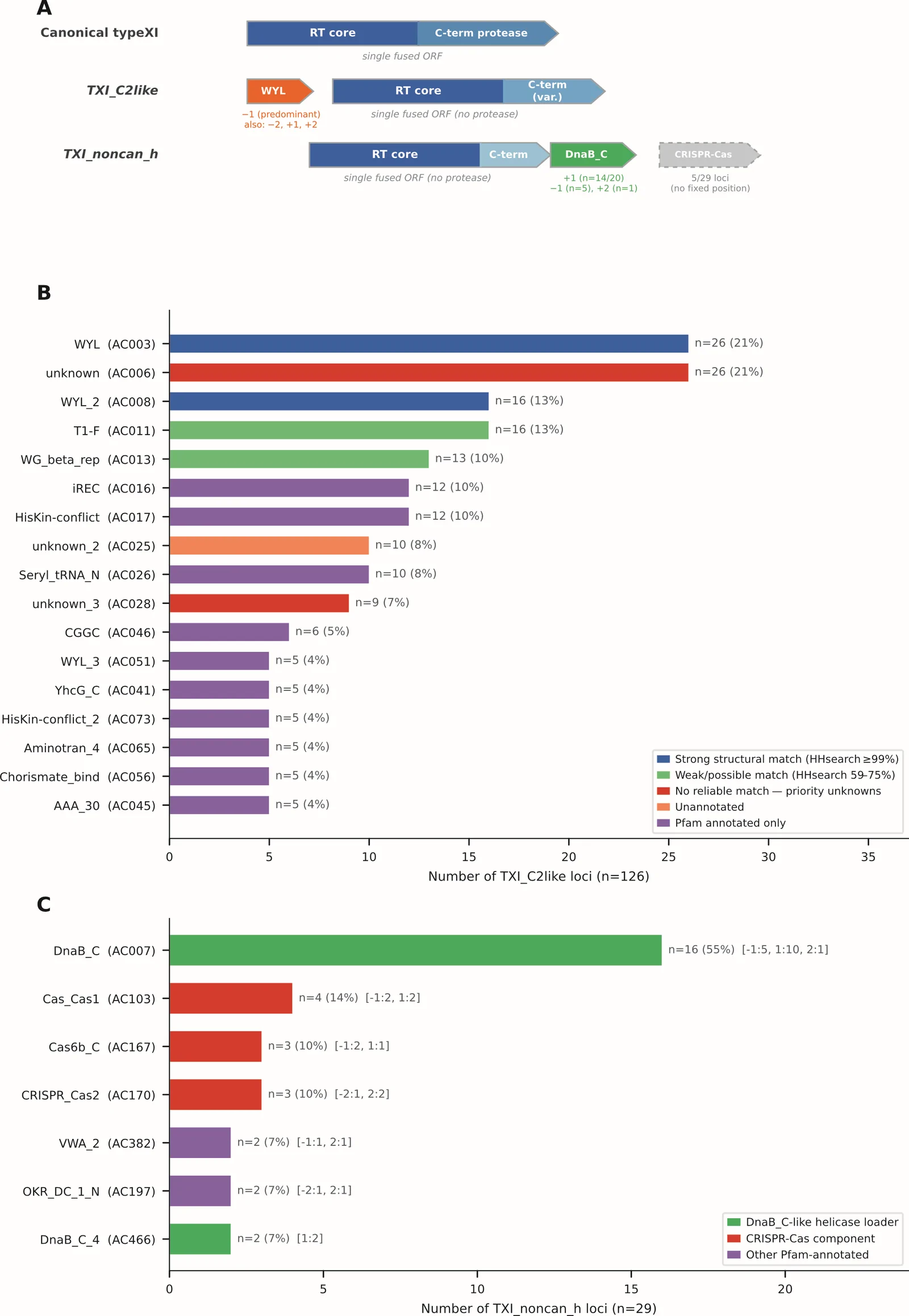
Genomic architecture and accessory protein repertoires of two noncanonical type XI-like lineages. (**A**) Schematic representation of the genomic organization of canonical type XI retrons and the two non-canonical lineages identified in this study. Canonical type XI retrons encode a single open reading frame in which the reverse transcriptase (RT) domain is fused to a C-terminal trypsin-like serine protease. In TXI_C2like, the RT lacks the protease domain and is encoded as a single ORF with a variable C-terminal extension, whereas accessory WYL-domain proteins are encoded as separate ORFs positioned upstream of the RT (offset −1, predominant) or at alternative positions within the ± 2 gene neighborhood (offsets −2, + 1, and + 2). TXI_noncan_h similarly lacks protease fusion and is associated with DnaB_C-containing proteins encoded as separate ORFs, most frequently at the immediate downstream position (offset + 1; 14 of 20 loci) although upstream positions also occur (offset −1; 5 of 20 loci). A subset of TXI_noncan_h loci was additionally associated with nearby CRISPR-Cas genes (5 of 29 loci, 17.2%), shown as a dashed arrow indicating absence of a fixed genomic position. Solid arrows indicate the predominant genomic organization. (**B**) Recurrent accessory protein clusters associated with TXI_C2like (*n* = 126). Bars indicate the number of loci in which each cluster was detected. Colors denote annotation confidence: blue, strong sequence-profile homology by HHsearch (probability ≥ 99%); green, tentative sequence-profile homology (probability 59%–75%); red, no reliable HHsearch match (priority novel unknowns); purple, Pfam-based annotation only. The dominant clusters corresponded to WYL-family proteins (AC003, AC008, and AC051; collectively detected in 47 loci, 37.3%) and the uncharacterized cluster AC006 (*n* = 26 loci). The dominant clusters corresponded to WYL-family proteins (AC003, AC008, and AC051; collectively detected in 47 loci, 37.3%) and the uncharacterized cluster AC006 (*n* = 26 loci). AC003, AC008, AC051, and AC006 were further evaluated by AlphaFold2 structure prediction and Foldseek structural comparison (see Results). (**C**) Recurrent accessory protein clusters associated with TXI_noncan_h (*n* = 29 curated loci). DnaB_C-containing proteins were detected in 20 loci (69.0%), a 368-fold enrichment over a phylum-matched genomic background (*P* = 2.9 × 10^−48^), and were dominated by AC007, with AC466 representing a secondary recurrent cluster. AC007 was further evaluated by AlphaFold2 structure prediction and Foldseek structural comparison (see Results). Two additional singleton clusters (AC377 and AC1199), each detected in a single locus, contributed to the overall total but are not shown. Positional offsets relative to the RT are indicated in brackets for each displayed cluster. CRISPR-Cas-associated clusters shown in the panel include AC103, AC167, and AC170; together with additional singleton CRISPR-Cas-associated clusters not shown, these occurred in 5 loci (17.2%). In several loci, multiple Cas components co-occurred, consistent with the presence of partial or complete CRISPR-Cas systems adjacent to the retron RT.

Canonical type XI retrons encode a single open reading frame in which the reverse transcriptase (RT) domain is fused to a C-terminal trypsin-like serine protease domain (8). Analysis of the 232 type XI-like candidates identified in SPIRE revealed that 207 loci (89.2%) lacked detectable C-terminal protease domains. Of these, 184 (88.9%) retained the conserved region X (NAxxH) and region Y (VTG) motifs characteristic of retron RTs and were distributed across 114 taxa. Systematic analysis of their genomic architectures and phylogenetic placement identified two recurrent non-canonical lineages, TXI_C2like (*n* = 126) and TXI_noncan_h (*n* = 29), in which the canonical protease fusion was replaced by distinct stand-alone accessory proteins encoded as separate open reading frames within the ±2 gene neighborhood of the RT (Fig. 4A).

TXI_C2like loci (*n* = 126; 11 high-confidence and 115 low-confidence candidates; 112 motif-compatible) were characterized by a recurrent association with WYL-domain proteins. WYL-associated modules were detected in 47 loci (37.3%), a 189-fold enrichment over a phylum-matched genomic background (*P* = 6.5 × 10^−93^), and grouped into three enriched accessory clusters: AC003 (WYL; *n* = 26 loci), AC008 (WYL_2; *n* = 16), and AC051 (WYL_3; *n* = 5). These proteins were found both upstream and downstream of the RT (offsets −2, −1, +1, and +2), indicating a flexible local organization rather than a fixed operon structure (Fig. 4A). HHsearch against the pdb70 database confirmed strong sequence similarity for the two dominant clusters: AC003 matched 7P5X_AX (probability = 99.6%, *E* = 5.1 × 10^−19^) and AC008 matched 7P5X_AY (probability = 99.7%, *E* = 4.2 × 10^−22^), both corresponding to the WYL superfamily fold, a nucleic acid-binding regulatory domain frequently associated with bacterial defense (34, 35). To complement this sequence-based inference with direct structural evidence, we performed AlphaFold2 structure prediction (ColabFold) followed by Foldseek structural comparison for AC003, AC008, AC051, and AC006. AC003, AC008, and AC051 all matched the WYL-domain activator DriD from *Caulobacter crescentus* (PDB8tp8/7u02; *E* ≤ 9.6 × 10^−11^), directly confirming the WYL-fold assignment for all three clusters, whereas AC006 returned no significant structural match (best *E* ≈ 1.0), consistent with its designation as a genuinely novel accessory module.

In addition to WYL proteins, TXI_C2like displayed a broader accessory repertoire comprising eight significantly enriched protein clusters relative to all type XI-like loci (Fisher’s exact test, FDR < 3.6 × 10^−8^; Table 2). Among these, AC006 (*n* = 26 loci; OR = ∞) encoded a protein lacking detectable Pfam domains, making it the most abundant uncharacterized accessory module associated with this lineage and a priority target for functional investigation (Fig. 4B; Table 2).

**TABLE 2:** Accessory protein clusters significantly associated with TXI_C2like and TXI_noncan_h retron sublineages^*a*^

AC	Label	Pfam annotation	Sublineage	*N* loci	OR	FDR
AC003	WYL	WYL	TXI_C2like	26	∞	1.7 × 10^−21^
AC006^*b*^	Unknown	–	TXI_C2like	26	∞	4.8 × 10^−20^
AC008	WYL_2	WYL	TXI_C2like	16	∞	5.1 × 10^−17^
AC011	T1-F	T1-F	TXI_C2like	16	∞	7.8 × 10^−15^
AC013	WG_beta_rep	WG_beta_rep	TXI_C2like	13	∞	7.5 × 10^−12^
AC016	iREC	iREC	TXI_C2like	12	62.4	2.5 × 10^−9^
AC017	HisKin-conflict	HisKin-conflict	TXI_C2like	12	62.4	2.5 × 10^−9^
AC026	Seryl_tRNA_N	Seryl_tRNA_N	TXI_C2like	10	∞	3.6 × 10^−8^
AC007	DnaB_C	DnaB_C	*TXI_noncan_h*	16	14.7	1.6 × 10^−9^
AC103^*c*^	Cas_Cas1	Cas1 (CRISPR)	*TXI_noncan_h*	4	∞	7.0 × 10^−4^
AC167^*c*^	Cas6b_C	Cas6b (CRISPR)	*TXI_noncan_h*	3	∞	2.0 × 10^−3^
AC170^*c*^	CRISPR_Cas2	Cas2 (CRISPR)	*TXI_noncan_h*	3	∞	2.0 × 10^−3^

^*a*^ OR, odds ratio from Fisher’s exact test with Benjamini-Hochberg FDR correction. ∞, cluster exclusive to sublineage. *N* loci, number of typeXI RT loci carrying the accessory cluster within a ±2-gene window. Sublineage: TXI_C2like shown in roman; TXI_noncan_h shown in italics.

^*b*^ AC006 encodes a protein of unknown function with no detectable Pfam domain or structural homology; priority novel module for functional characterization.

^*c*^ AC103 (Cas1), AC167 (Cas6b), and AC170 (Cas2) co-occur in overlapping sets of TXI_noncan_h loci, consistent with components of complete CRISPR-Cas systems. Their association suggests functional coupling between TXI_noncan_h retrons and adaptive immunity systems.

TXI_noncan_h loci (*n* = 29 curated loci; all low-confidence; 22 motif-compatible; *n* = 24 included in phylogenetic analysis) were dominated by DnaB_C-like helicase-domain proteins encoded as separate ORFs adjacent to the RT, which were detected in 20 loci (69.0%). These proteins showed a strong positional bias toward the immediate downstream gene (offset +1; 14 loci) although upstream occurrences were also observed (offset −1; 5 loci; offset +2; 1 locus), suggesting a conserved functional coupling between RT and the associated helicase-related protein. A subset of loci (5 of 29; 17.2%) was located in proximity to CRISPR-Cas genes without a consistent positional pattern, consistent with their presence within broader defense islands rather than in a conserved operon arrangement. No WYL-domain proteins were detected in TXI_noncan_h, clearly distinguishing this lineage from TXI_C2like (Fig. 4A and C). Statistical enrichment analysis identified AC007 (DnaB_C; *n* = 16 loci; OR = 14.7; FDR = 1.6 × 10⁻⁹) as the dominant accessory cluster in the study population. AlphaFold2 structure prediction and Foldseek comparison of the AC007 representative returned a highly significant match to *Escherichia coli* DnaB helicase in complex with DnaG primase (PDB7t22; *E* = 2.0 × 10⁻²⁴), providing direct structural confirmation of the DnaB_C assignment.

Three additional clusters corresponding to CRISPR-Cas components, AC103 (Cas1; *n* = 4), AC167 (Cas6b; *n* = 3), and AC170 (Cas2; *n* = 3) were detected exclusively in TXI_noncan_h loci (OR = ∞; FDR < 2 × 10⁻³). The co-occurrence of Cas1, Cas2, and Cas6b in overlapping loci suggests the presence of complete CRISPR-Cas systems adjacent to the retron rather than the isolated recruitment of individual components (Table 2).

Together, TXI_C2like and TXI_noncan_h are defined by two distinct patterns of accessory module recruitment around protease-less type XI-like RTs: a WYL-enriched lineage with a diverse and flexible accessory repertoire (TXI_C2like) and a DnaB_C-dominated lineage characterized by preferential downstream positioning and occasional association with CRISPR-Cas systems (TXI_noncan_h).

**Fig 5:**
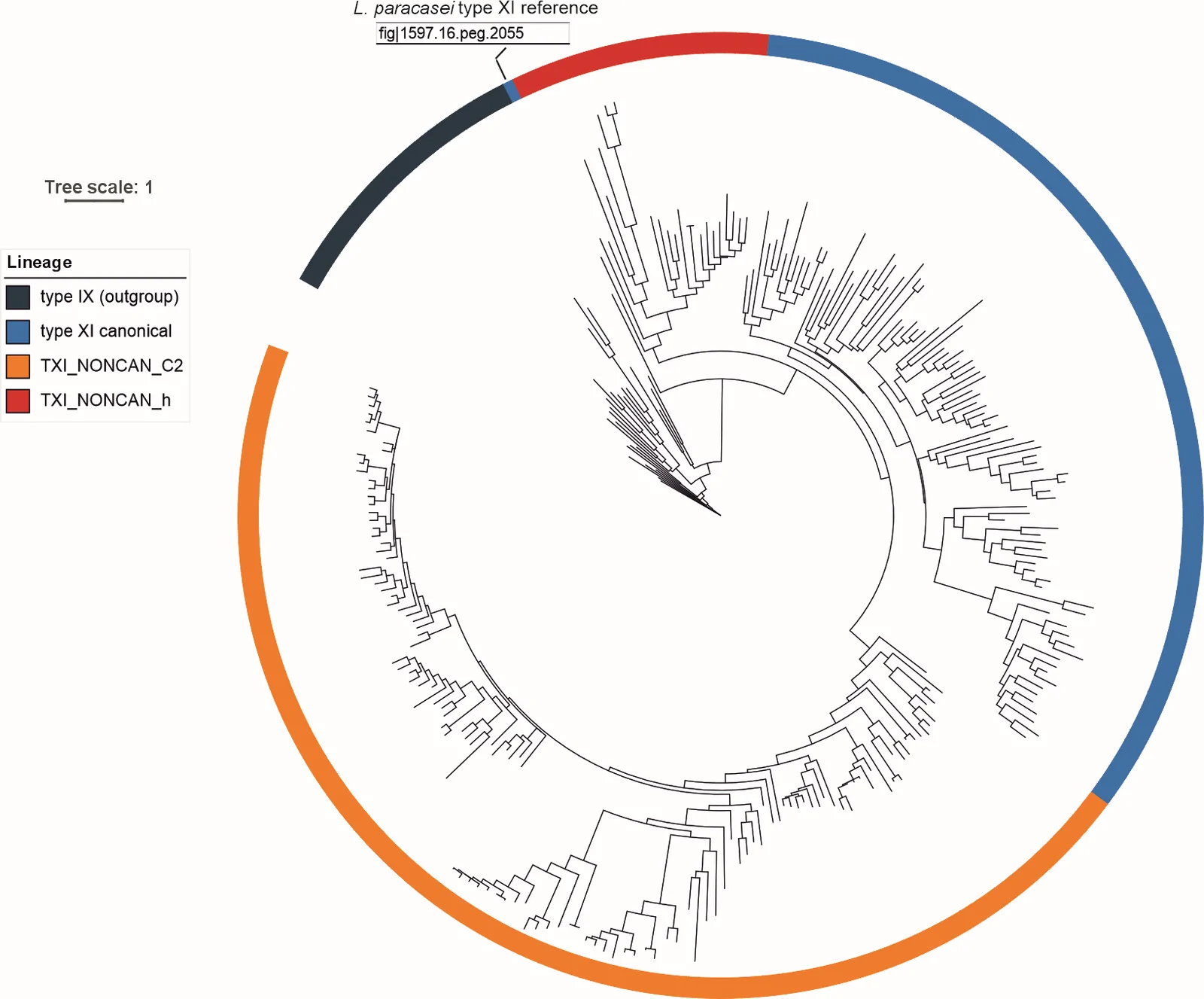
Phylogenetic structure and evolutionary diversification of noncanonical type XI-like retrons. Maximum-likelihood phylogeny of canonical and non-canonical type XI-like retron reverse transcriptases (RTs) inferred with IQ-TREE2 using the LG + I + G4 substitution model and 1,000 ultrafast bootstrap replicates from an alignment of the conserved RT domain. Type IX retron RTs (black) were included as outgroups to root the tree. TXI_C2like (*n* = 126; orange) and TXI_noncan_h (*n* = 24; red) formed two distinct monophyletic radiations within the broader type XI clade (blue), rather than representing sporadic architectural variants dispersed among canonical type XI retrons. TXI_C2like was composed exclusively of Bacteroidota sequences, whereas TXI_noncan_h comprised both Bacillota and Bacteroidota members. The position of the protease-fused type XI reference sequence from *L. paracasei* (fig1597.16_peg.2055 [8]) is indicated in the tree. This sequence branches basally to the canonical and non-canonical type XI-like lineages with strong bootstrap support (93%); see Results for discussion of the evolutionary implications of this topology. The complete phylogenetic tree is provided as Data S2.

Phylogenetic analysis showed that TXI_C2like and TXI_noncan_h do not represent sporadic architectural variants scattered throughout the canonical type XI tree but instead form two distinct monophyletic radiations within a broader type XI-like clade (Fig. 5). TXI_C2like formed a well-supported branch composed exclusively of Bacteroidota sequences, whereas TXI_noncan_h formed a separate lineage comprising both Bacillota and Bacteroidota members. A protease-fused type XI RT from *Lactobacillus paracasei* branched basally to both non-canonical lineages with strong bootstrap support (93%). This basal position suggests that protease fusion may represent the ancestral state of this clade though the lack of detectable sequence-level homology between the canonical protease domain and the non-canonical C-terminal extensions (mean pairwise identity 3.9%–7.9%) means we cannot exclude alternative scenarios, including independent modular acquisitions in divergent lineages.

### Identification of candidate msr/msd-like ncRNA structures in TXI_noncan_h and TXI_C2like retrons

Type XI retrons were previously recognized as one of the few retron lineages for which no msr/msd-like ncRNA structure could be identified computationally (8). To determine whether the expanded TXI_noncan_h and TXI_C2like data sets recovered from SPIRE provided sufficient sequence diversity for ncRNA discovery, we performed *de novo* searches for conserved RNA secondary structures in the genomic regions upstream of RT genes.

**Fig 6:**
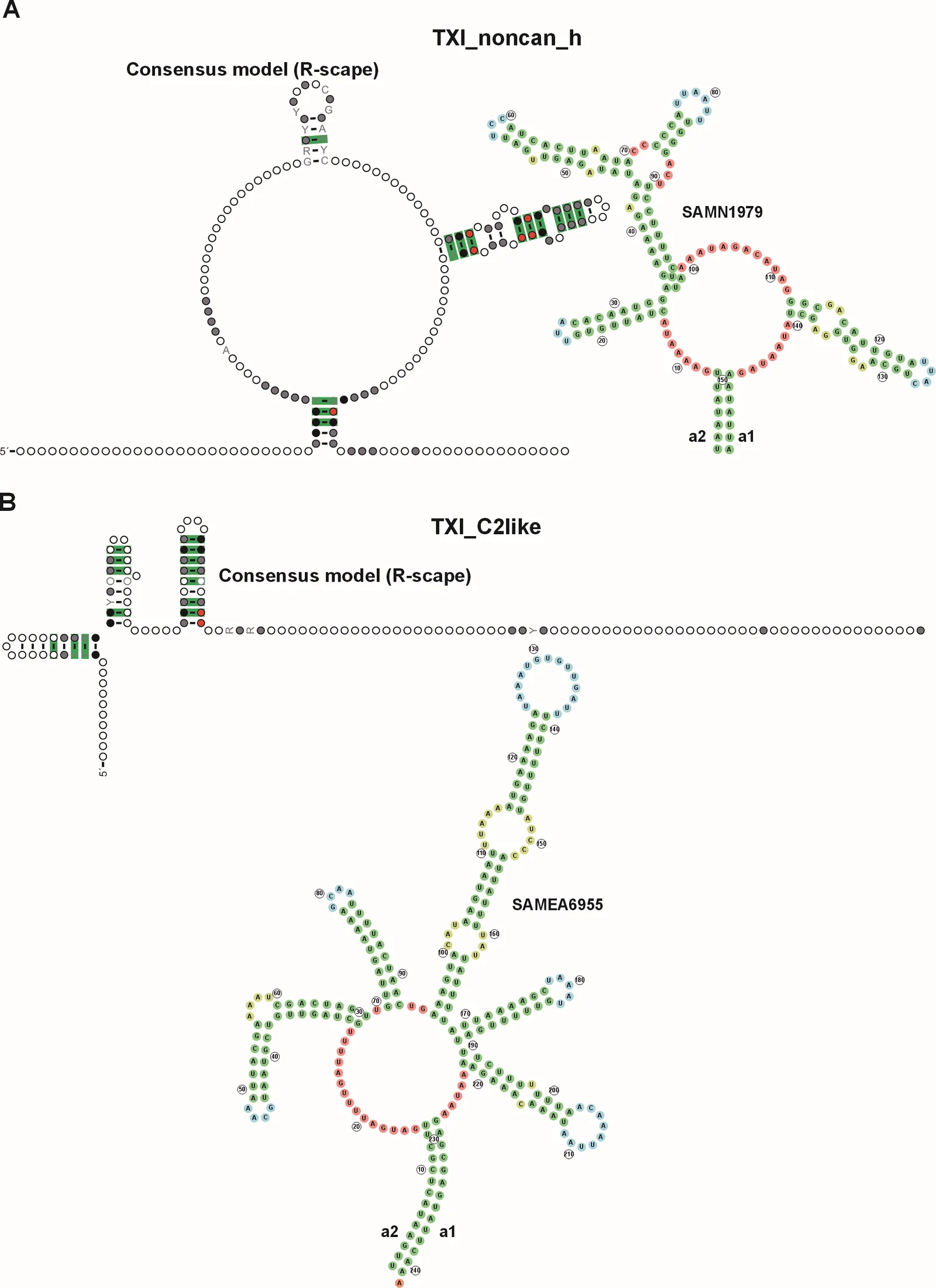
Candidate msr/msd-like ncRNA structures identified in non-canonical type XI-like retrons. (**A**) TXI_noncan_h. Consensus secondary structure of a conserved ncRNA element identified in 20 loci using the 300 bp immediately upstream of the RT start codon. The consensus model was derived from a structure-guided multiple sequence alignment generated using mLocARNA 2.0.1 and evaluated using R-scape 2.0.4. A representative RNAfold/FORNA structure from an individual locus is shown alongside the consensus model and closely recapitulates the predicted double-stem-loop architecture. (**B**) TXI_C2like. Consensus secondary structure of a conserved ncRNA element identified in 37 loci, using the 300 bp immediately upstream of the reverse transcriptase (RT) start codon. The consensus model was generated and evaluated as described for panel A. A representative RNAfold/FORNA structure from an individual locus is shown alongside the consensus model and recapitulates the predicted three-stem-loop architecture. For both panels, the circle size indicates positional occupancy (the proportion of sequences containing a nucleotide at each alignment position), and the circle color indicates nucleotide conservation (red, ≥97%; dark gray, ≥90%; light gray, ≥75%; white, ≥50%). Base pairs showing statistically significant covariation above the phylogenetic expectation (R-scape *E* < 0.05) are highlighted in green. Conserved inverted repeat elements corresponding to the canonical a1 and a2 regions are indicated, and the 5′ end of each structure is marked.

For TXI_noncan_h, CMfinder identified a recurrent candidate motif in 20 of the 28 loci. A covariance model built from this motif detected significant matches exclusively upstream of the RT start codon at distances of 22–104 bp. Structure-guided alignment of the 300 bp immediately upstream of the RT start codon (27 sequences), followed by R-scape analysis, yielded a consensus structure comprising 23 base pairs, 13 of which showed statistically significant covariation (observed/expected ratio = 1.23; *E* < 0.05, individual *E*-values ranging from 4.70 × 10⁻⁷ to 0.0243). CaCoFold, R-scape’s covariation-guided *de novo* structure refinement algorithm, recovered all 13 significant pairs and identified four additional supported pairs, confirming the robustness of the consensus structure. The predicted RNA adopts a compact double stem-loop architecture containing a conserved sequence motif consistent with the a1/a2 inverted-repeat organization, characteristic of canonical retron msr elements (Fig. 6A).

For TXI_C2like, CMfinder identified a recurrent upstream motif in 73 of the 120 loci. Covariance model searches confirmed significant matches in 37 loci, all located 38–388 bp upstream of the RT start codon. Structure-guided alignment and R-scape analyses yielded a consensus structure comprising 25 predicted base pairs, 14 of which showed statistically significant covariation (observed/expected ratio = 1.16; *E* < 0.05, individual *E*-values ranging from 5.85 × 10⁻¹⁰ to 0.0178). The resulting model displayed a three-stem-loop architecture flanked by inverted-repeat elements consistent with the a1/a2 regions characteristic of canonical retron msr/msd transcripts (Fig. 6B). Comparable structures were recovered, irrespective of whether the associated WYL-domain protein was encoded upstream or downstream of the RT, indicating that the ncRNA is functionally linked to the RT rather than to the accessory module.

Representative RNAfold structures from individual loci closely matched the corresponding consensus models, supporting the structural conservation of both ncRNA families (Fig. 6A and B).

Together, these analyses provide the first computational evidence for candidate msr/msd-like ncRNA elements in type XI retrons, addressing a gap highlighted in previous surveys of retron ncRNA diversity (8). Both TXI_noncan_h and TXI_C2like encode distinct conserved RNA structures upstream of their RT genes, indicating that these highly divergent lineages retain the core architectural organization of canonical retrons despite their non-canonical protein architectures.

## DISCUSSION

This study provides the first systematic survey of retron diversity across the SPIRE microbial genome resource, encompassing 107,078 representative genomes and identifying 1,286 retron candidates spanning all 13 canonical retron types, together with additional divergent lineages. In addition to confirming the broad taxonomic and ecological distribution of known retrons, our analysis uncovered two candidate novel type XI-like lineages: TXI_C2like and TXI_noncan_h. These findings demonstrate that large-scale genome-resolved surveys continue to reveal substantial unexplored diversity in retron systems.

Host-associated environments accounted for half of all retron candidates, and type XI-like retrons were strongly enriched in Bacteroidota. The pronounced host association of clade2_Ec107like further suggests that Bacillota-dominated gut communities represent a productive niche for diversification of this lineage. Quantitative analysis of taxonomic breadth using the Gini-Simpson diversity index revealed a continuum ranging from highly specialized groups, such as clade2_Ec107like and type I-C2, to broadly distributed lineages, including types VI and XIII. These contrasting patterns likely reflect distinct evolutionary strategies: specialized lineages may have co-evolved with particular bacterial hosts and their associated phage communities, whereas more cosmopolitan retrons may spread more readily through horizontal gene transfer across diverse bacterial taxa.

The subdivision of the type XI-like clade underscores the importance of resolving biologically meaningful sublineages. The apparent Bacteroidota specificity of the overall type XI-like group was driven primarily by TXI_C2like, which was detected exclusively in Bacteroidota, whereas canonical type XI retrons showed a broader taxonomic distribution, and TXI_noncan_h was recovered from both Bacteroidota and Bacillota. The ecological enrichment of type X in aquatic environments and type V in anthropogenic habitats further indicates that retron distribution is shaped not only by phylogenetic history but also by environmental context, potentially reflecting adaptation to habitat-specific phage communities. Although these patterns likely reflect genuine ecological constraints, they may also be influenced by uneven sampling across environments, emphasizing the need to explore under-represented habitats such as soils, deep marine ecosystems, and extreme environments.

The designation of TXI_C2like and TXI_noncan_h as candidate novel retron lineages is supported by five independent lines of evidence: distinct RT phylogeny, non-canonical domain architectures, lineage-specific accessory modules, computational identification of candidate msr/msd-like ncRNA elements, and AlphaFold2/Foldseek structural homology of the RT domain to characterized retron reverse transcriptases (including the retron Ec86 effector complex, PDB 9jm0). Previous surveys were unable to detect ncRNA structures associated with type XI retrons (8), highlighting the unusual divergence of this lineage and the limitations of the sequence data available at the time. By leveraging the expanded SPIRE data set together with structure-guided alignment using mLocARNA and covariance analysis with R-scape, we identified conserved RNA structures with statistically supported covariation in both lineages. The three stem-loop architecture of TXI_C2like and the compact a1/a2-flanked structure of TXI_noncan_h are consistent with the general organizational principles of canonical retron ncRNAs, indicating that these highly divergent systems likely retain the same core functional framework.

The accessory modules associated with these lineages suggest distinct biological roles. TXI_C2like is recurrently associated with WYL-domain proteins, a class of regulatory factors implicated in nucleic acid sensing and frequently linked to bacterial defense systems (34), an assignment further corroborated by AlphaFold2/Foldseek structural comparison. In contrast, TXI_noncan_h is associated with DnaB_C-containing proteins, similarly confirmed by structural comparison, and, in a subset of loci, with nearby CRISPR-Cas genes, raising the possibility of functional coupling between retrons and other bacterial immune systems, consistent with the modular organization of defense islands in microbial genomes (10, 35). The significance of the DnaB_C association remains unclear, but it may indicate a replication-related function or structural role within the retron ribonucleoprotein complex.

Several limitations should be considered. The conservative HMM threshold used for candidate identification prioritized specificity at the expense of sensitivity and likely underestimated the abundance of highly divergent retrons, particularly within types VI, I-B1, I-B2, and IV retrons. Searches using relaxed thresholds recovered additional candidates, but their classification was less reliable because of reduced HMM specificity in this score range. The ncRNA structures described here remain computational predictions and require further experimental validation. In addition, the predominance of metagenome-assembled genomes in SPIRE introduces potential assembly fragmentation biases that may affect genomic context analysis.

The strong ecological specialization of TXI_C2like and TXI_noncan_h to host-associated Bacteroidota and Bacillota environments has practical implications for retron-based biotechnology. Retron systems are increasingly explored as programmable gene-editing tools and biosensors in bacterial and human cell contexts (6, 7). Lineages adapted to gut-associated environments may offer enhanced activity in relevant host-associated organisms, as the native cellular context—GC content, codon usage, protein folding chaperones—is more closely matched to the intended application host. The TXI_C2like and TXI_noncan_h systems, with their distinct accessory architectures (WYL-domain and DnaB_C-domain proteins, respectively), represent unexplored functional diversity with potential for engineering novel retron-based tools operating through mechanisms distinct from canonical msDNA synthesis.

Overall, the discovery of TXI_C2like and TXI_noncan_h expands the known diversity of retron systems and highlights type XI-like retrons as a particularly dynamic evolutionary group. Experimental characterization of the associated WYL-RT and DnaB_C-RT modules, together with validation of the predicted ncRNA structures, will be essential to determine whether these systems synthesize msDNA and to clarify their biological roles. Their strong association with gut-associated Bacteroidota and Bacillota suggests that suitable cultured representatives may be available for future studies.

## Data Availability

The computational pipeline scripts used in this study, the phylogenetic trees corresponding to Fig. 1 and 5, and the 29 type-specific retron RT HMM profiles (together with the global reference profile and their calibration thresholds) are deposited at Zenodo (https://doi.org/10.5281/zenodo.20409741) under MIT license. The SPIRE microbial metagenome resource is publicly available at https://spire.embl.de (12). The retron reference sequence data set of 1,928 RT sequences used for HMM construction and phylogenetic placement is available from Mestre et al. (8). Covariance models generated in this study are available from the corresponding author upon reasonable request.

## SUPPLEMENTAL MATERIAL

Data file S1 msystems.00732-26-s0001.txt Retron reference phylogeny.

Data file S2 msystems.00732-26-s0002.txt Type XI local tree.

Supplemental material msystems.00732-26-s0003.pdf Fig. S1 to S3 and captions for supplemental tables and data files.

Table S1 msystems.00732-26-s0004.pdf HMM calibration thresholds for the 29 type-specific retron RT profiles used in SPIRE candidate classification.

Table S2 msystems.00732-26-s0005.pdf Complete list of 1,286 SPIRE-native retron RT candidates.

Table S3 msystems.00732-26-s0006.pdf Retron group enrichment by taxonomic order.

Table S4 msystems.00732-26-s0007.pdf Detailed host-organism and biome association of host-associated SPIRE retron candidates.

Table S5 msystems.00732-26-s0008.pdf Systematic evidence review of all 28 SPIRE retron classification groups.

ASM does not own the copyrights to Supplemental Material that may be linked to, or accessed through, an article. The authors have granted ASM a non-exclusive, world-wide license to publish the Supplemental Material files. Please contact the corresponding author directly for reuse.
