# Supplementary material for: Landscape of retron diversity across the SPIRE microbial metagenome resource reveals candidate novel type XI-like lineages
Source: mSystems. 2026 Sep 11;11(10):e00732-26. doi: 10.1128/msystems.00732-26 (PMC13712802; doi:10.1128/msystems.00732-26)
Supplement: Supplementary file 1 [file msystems.00732-26-s0003.pdf]

Supplementary Materials

Supplementary Figures

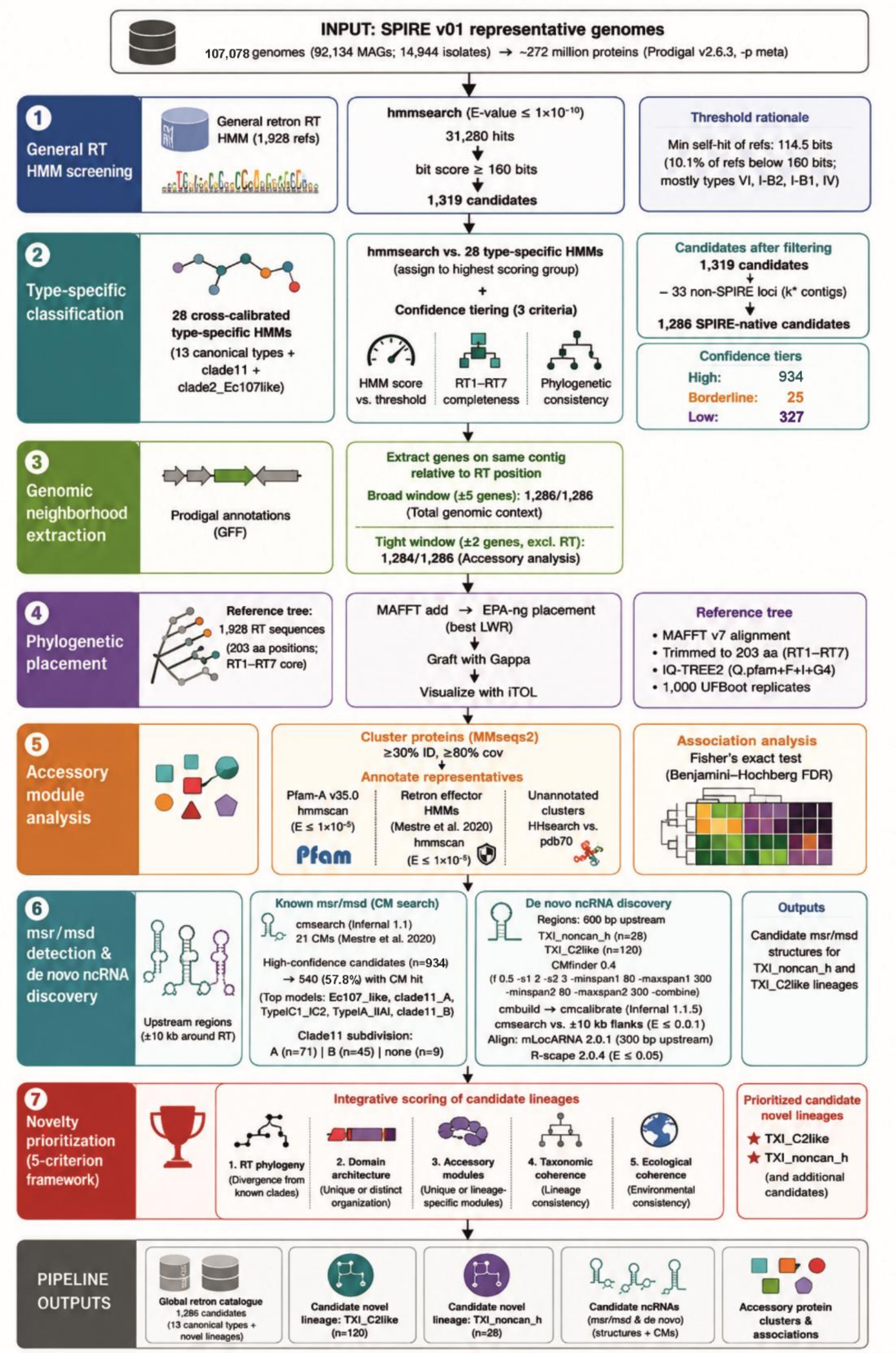

Supplementary Figure S1. Overview of the computational pipeline used for retron discovery and classification in the SPIRE representative genome collection**.** Protein-coding genes predicted from 107,078 representative genomes were screened using a general retron RT HMM, followed by classification with 28 cross-calibrated type-specific HMMs. Candidates were further evaluated through genomic neighbourhood analysis, phylogenetic placement, accessory module annotation, covariance model searches for known msr/msd elements, and de novo ncRNA structure discovery. A five-criterion novelty prioritization framework integrating RT phylogeny, domain architecture, accessory modules, taxonomic coherence, and ecological coherence was used to identify candidate novel retron lineages.

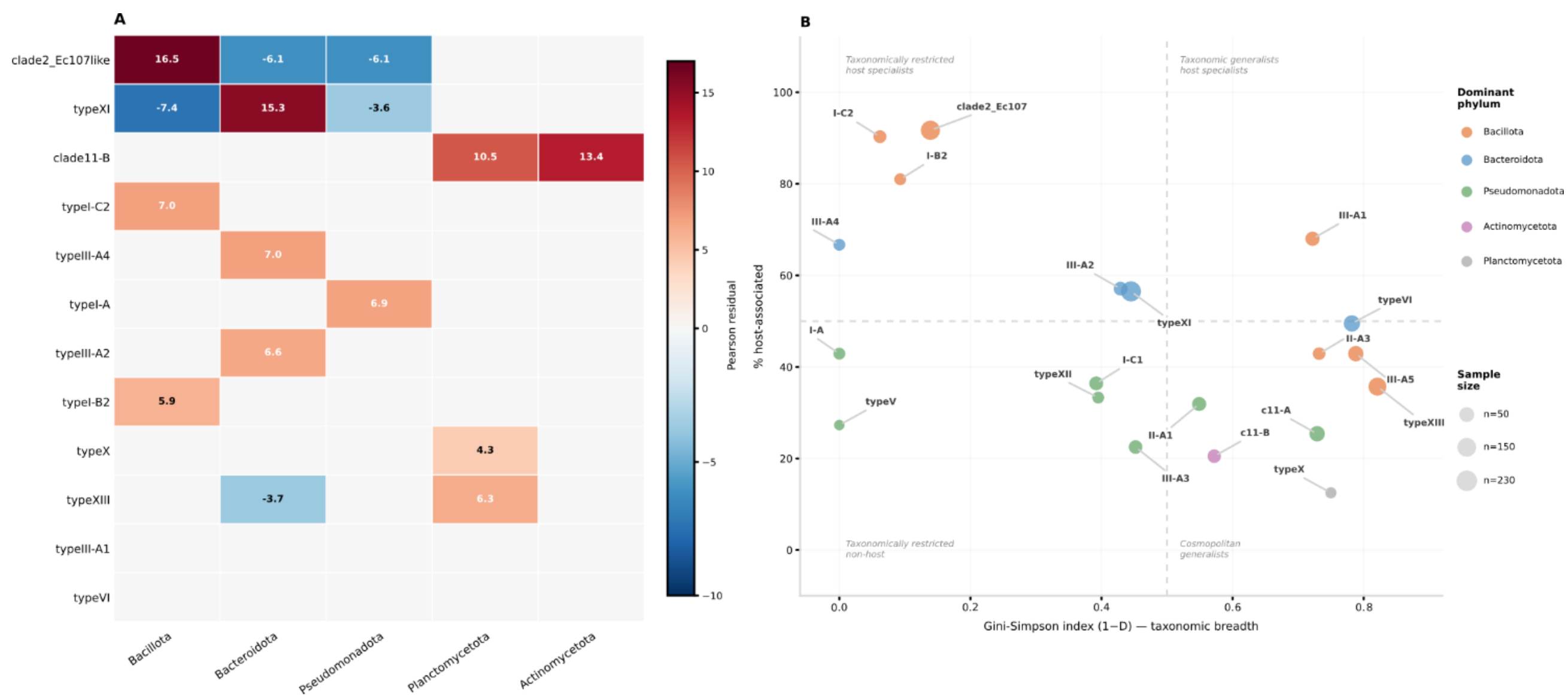

Supplementary Figure S2. Statistical associations between retron types, phyla, and ecological specialization. (**A**) Heatmap of Pearson standardized residuals from Chi² contingency analysis of retron group × dominant phylum (Chi²=2,764.8, df=375, $p<10^{-300}$). Red cells indicate significant enrichment, blue cells significant depletion relative to the expected frequency under independence. Only groups with n≥10 and phyla with n≥5 are shown. (**B**) Scatter plot of taxonomic breadth (Gini–Simpson diversity index, 1 − D; x-axis) versus ecological specialization (% host-associated, y-axis)

for each retron group. Bubble size is proportional to the total number of candidates, and colors indicate the dominant phylum. Groups in the lower-left quadrant (low $1 - D$, high host%) are taxonomically restricted and ecologically specialized; groups in the lower-right quadrant (high $1 - D$, low host%) are broadly distributed ecological generalists.

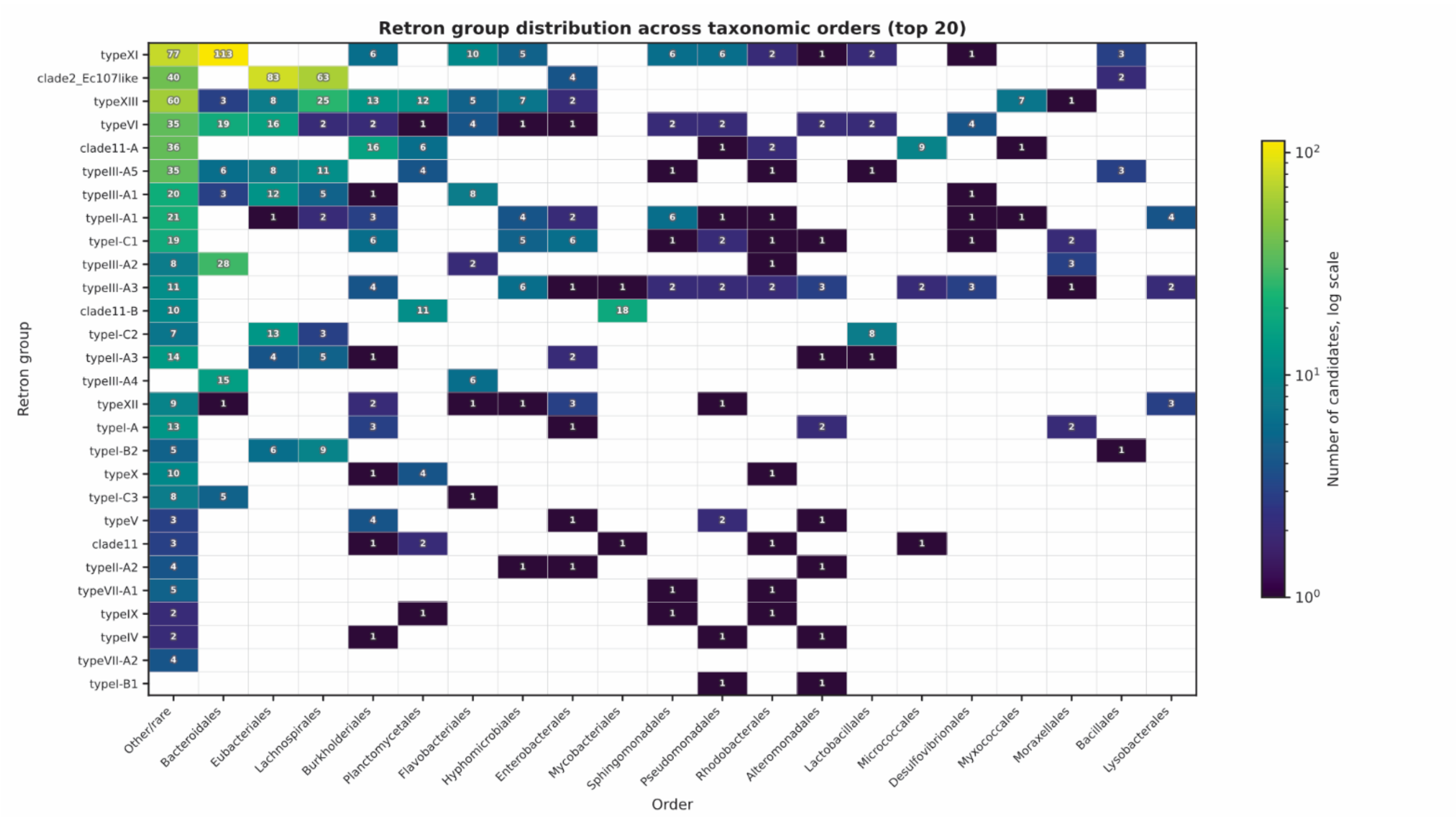

Supplementary Figure S3. Retron group distribution across taxonomic orders.Heatmap of retron candidate counts (color scale, log-transformed) for each retron group across the top 20 most represented taxonomic orders; orders outside the top 20 are aggregated as "Other/rare". Cell values indicate the number of candidates in each group–order combination; white cells indicate zero candidates. Underlying data derive from a chi-squared test of independence on the full group × order contingency table (order $n \geq 5$, group $n \geq 10$; 898 of 1,286 candidates, 21 groups × 35 orders; $\chi^2 = 3{,}438.3$, $df = 680$, $p < 10^{-300}$). Standardized Pearson residuals identifying the specific group–order associations driving this result are reported in **Supplementary Table S3.**

Supplementary Tables

Table S1. HMM calibration thresholds for the 29 type-specific retron RT profiles used in SPIRE candidate classification

Table S2. Complete list of 1,286 SPIRE-native retron RT candidates

Table S3. Retron group enrichment by taxonomic order

Table S4. Detailed host-organism and biome association of host-associated SPIRE retron candidates

Table S5. Systematic evidence review of all 28 SPIRE retron classification groups

Supplementary Data Files

Data File 1: retron_reference_phylogeny_EPAng.newick

Data File 2: typeXI_local_tree.contree
