# Supplementary material for: Landscape of retron diversity across the SPIRE microbial metagenome resource reveals candidate novel type XI-like lineages
Source: mSystems. 2026 Sep 11;11(10):e00732-26. doi: 10.1128/msystems.00732-26 (PMC13712802; doi:10.1128/msystems.00732-26)
Supplement: Supplementary file 2 [file msystems.00732-26-s0004.pdf]

Supplementary Table S1. HMM calibration thresholds for the 29 type-specific retron RT profiles used in SPIRE candidate classification.

For each HMM, pos_min = minimum self-hit score (HMM vs its own reference sequences); neg_max = maximum cross-hit score (HMM vs all other groups' reference sequences); threshold = (pos_min + neg_max) / 2. Overlap zone: neg_max > pos_min (classification inherently ambiguous). Clean separation: neg_max < pos_min (groups well-separated). High-confidence: score ≥ threshold. Low-confidence: score ≥ 160 but < threshold. Borderline: score within the overlap zone between distributions.

Retron type	pos_min (bits)	neg_max (bits)	threshold (bits)	Separation	Notes	n_ref_seqs_below_160
**clade11**	250,2	345,7	297,9	Overlap	Overlap zone; borderline assignments expected	—
**clade11-A**	290,3	344,6	317,4	Overlap	Overlap zone; OutgroupA ncRNA sub-clade	—
**clade11-B**	276,7	326,2	301,4	Overlap	Overlap zone; OutgroupB ncRNA sub-clade	—
**clade2_Ec107like**	230,5	258,9	244,7	Overlap	Overlap zone; new HMM in pipeline v2	—
**typeI-A**	276,7	301,3	289,0	Overlap	Overlap zone	—
**typeI-B1**	236,6	144,4	190,5	Clean	Clean separation; 34 refs below screening threshold	**34**
**typeI-B2**	216,2	138,2	177,2	Clean	Clean separation; 36 refs below screening threshold	**36**
**typeI-C1**	249,7	311,2	280,4	Overlap	Overlap zone	—
**typeI-C2**	232,8	295,3	264,1	Overlap	Overlap zone	—
**typeI-C3**	310,3	285,8	298,1	Clean	Clean separation	—
**typeII-A1**	192,8	277,2	235,0	Overlap	Overlap zone; 2 refs below screening threshold	**2**
**typeII-A2**	292,7	216,7	254,7	Clean	Clean separation	—
**typeII-A3**	245,0	267,9	256,4	Overlap	Overlap zone	—
**typeIII-A1**	210,6	256,5	233,6	Overlap	Overlap zone	—
**typeIII-A2**	239,7	252,8	246,2	Overlap	Overlap zone	—
**typeIII-A3**	200,9	230,5	215,7	Overlap	Overlap zone	—
**typeIII-A4**	274,3	250,5	262,4	Clean	Clean separation	—
**typeIII-A5**	235,3	264,9	250,1	Overlap	Overlap zone	—
**typeIV**	263,0	200,5	231,8	Clean	Clean separation; 20 refs below screening threshold	**20**
**typeIX**	253,0	187,8	220,4	Clean	Clean separation	—
**typeV**	279,8	218,9	249,3	Clean	Clean separation; 10 refs below screening threshold	**10**
**typeVI**	162,9	207,8	185,3	Overlap	Overlap zone; 91 refs below screening threshold — most affected	**91**
**typeVII-A1**	303,6	177,9	240,8	Clean	Clean separation	—
**typeVII-A2**	292,7	172,3	232,5	Clean	Clean separation	—
**typeVIII**	360,6	157,0	258,8	Clean	Clean separation; 2 refs below screening threshold	**2**
**typeX**	306,2	211,2	258,7	Clean	Clean separation	—
**typeXI**	233,3	294,2	263,8	Overlap	Overlap zone; large low-confidence fraction in SPIRE	—
**typeXII**	263,5	302,4	282,9	Overlap	Overlap zone	—
**typeXIII**	226,3	255,0	240,7	Overlap	Overlap zone	—
**TOTALS / RANGE (n=29 HMMs)**				**Overlap: 16 | Clean: 13**	**Threshold range: 177.2 – 317.5 bits**	**Total: 195 refs**

Legend:
■ Yellow (Overlap): neg_max > pos_min — classification ambiguous; higher borderline/low fraction expected. ■ Green (Clean): neg_max < pos_min — groups well-separated; high-confidence classification reliable. ■ Red values in column G: number of reference sequences with self-hit score < 160 bits (below the general screening threshold; these would not be recovered in SPIRE).
