# Supplementary material for: Landscape of retron diversity across the SPIRE microbial metagenome resource reveals candidate novel type XI-like lineages
Source: mSystems. 2026 Sep 11;11(10):e00732-26. doi: 10.1128/msystems.00732-26 (PMC13712802; doi:10.1128/msystems.00732-26)
Supplement: Supplementary file 3 [file msystems.00732-26-s0005.pdf]

Supplementary Table S2. Complete list of 1,286 SPIRE-native retron RT candidates identified in this study. Candidates are sorted by retron group and HMM score. Confidence class: high = score ≥ group-specific calibrated threshold; borderline = score within the overlap zone; low = score ≥ 160 bits but below threshold.

Protein ID	Retron group	HMM score	Confidence class	Phylum	Class	Order	Family	Genus	Species	Sample ID	Biome	N neighbors (±5 genes)	Has tight neighbor (±2 genes)	N accessory clusters	Top Pfam annotation (neighbor)	Top effector annotation (neighbor)
1736354.SAMN04151744.LMQA01000(	clade11	340,6	high	Actinomycetota	Actinomycetes	Geodermatophilales	Geodermatophilaceae	Geodermatophilus	Geodermatophilus sp. Leaf369	SAMN04151744	host-associated	11	Yes	4	ABC_tran;Xan_ur_permease;DUF1	—
1443900.SAMN02569999.JMER01000(	clade11	328,1	high	Actinomycetota	Actinomycetes	Mycobacteriales	Nocardiaceae	Rhodococcoides	Rhodococcoides fascians	SAMN02569999	aquatic	11	Yes	4	DeoRC;YrhK;DUF3558;LEDGF	—
2020130.SAMN07346923.CP022462_2	clade11	322,5	high	Actinomycetota	Actinomycetes	Micrococcales	Micrococcaceae	Arthrobacter	Arthrobacter sp. 7749	SAMN07346923	host-associated	11	Yes	4	HD_assoc;Dus;Bac_luciferase;Acet	—
2026779.SAMN13893644.JAAYAY0100	clade11	299,3	high	Planctomycetota	Planctomycetia	Planctomycetales	Planctomycetaceae	—	Planctomycetaceae bacterium	SAMN13893644	temperature	8	Yes	4	FAD_oxidored;ThuA;PD40;HZS_al	—
1191521.SAMEA6955893.CAIRTJ0100(	clade11	294,4	high	Verrucomicrobiota	Pedosphaerae	Pedosphaerales	Pedosphaeraceae	Pedosphaera	uncultured Pedosphaera sp.	SAMEA6955893	aquatic	11	Yes	4	Beta_helix;UnbV_ASPIC;DabA;Pro	—
904984.SAMEA6956427.CAIMFH01000	clade11	288,8	high	Planctomycetota	Planctomycetia	Planctomycetales	Planctomycetaceae	Schlesneria	uncultured Schlesneria sp.	SAMEA6956427	aquatic	11	Yes	4	Dus;PMT_2;Response_reg;FHA	—
406425.SAMN02598404.CP000960_57	clade11	280,6	high	Pseudomonadota	Betaproteobacteria	Burkholderiales	Burkholderiaceae	Burkholderia	Burkholderia orbicola	SAMN02598404	host-associated	11	Yes	4	MFS_1;Histone_HNS;PFIN;UTRA	—
1062.SAMN16425547.JADJAB0100000	clade11	279,1	high	Pseudomonadota	Alphaproteobacteria	Rhodobacterales	—	Rhodobacter	Rhodobacter sp.	SAMN16425547	anthropogenic	11	Yes	4	Peptidase_S13;CTP_transf_like;AB	—
153809.SAMEA6947388.CAISGC0100(	clade11	264,6	high	Pseudomonadota	—	—	—	—	uncultured Pseudomonadota bacterium	SAMEA6947388	aquatic	11	Yes	4	FGE-sulfatase;NYN_YacP;ACR_tra	—
479431.SAMN02598467.CP001737_31	clade11-A	349,8	high	Actinomycetota	Actinomycetes	Nakamurellales	Nakamurellaceae	Nakamurella	Nakamurella multipartita	SAMN02598467	anthropogenic	11	Yes	4	CBM_48;Zn_peptidase;LysE	—
1736303.SAMN04151693.LMLU010000	clade11-A	341	high	Actinomycetota	Actinomycetes	Micrococcales	Micrococcaceae	Arthrobacter	Arthrobacter sp. Leaf234	SAMN04151693	anthropogenic	11	Yes	4	BPD_transp_1;DUF5996;ABC_tran	—
1032480.SAMD00061117.AP012204_2	clade11-A	337	high	Actinomycetota	Actinomycetes	Propionibacteriales	Propionibacteriaceae	Microlunatus	Microlunatus phosphovorus	SAMD00061117	anthropogenic	11	Yes	4	DUF2252;tRNA-synt_His;ROK;CS	—
2769423.SAMD00245656.BNCN010000	clade11-A	333,1	high	Pseudomonadota	Betaproteobacteria	Burkholderiales	Comamonadaceae	Comamonas	Comamonas sp. KCTC 72670	SAMD00245656	host-associated	11	Yes	4	HSP70;Phage_holin_3_6;DUF659	—
2026658.SAMN14167782.JAAKZI0100(	clade11-A	332,3	high	Actinomycetota	Actinomycetes	Micrococcales	Micrococcaceae	Arthrobacter	Arthrobacter silviterrae	SAMN14167782	host-associated	11	Yes	4	DSBA;Guanylate_cyc	—
1898745.SAMN05736493.MJGS010000	clade11-A	331,7	high	Actinomycetota	Actinomycetes	Micrococcales	Microbacteriaceae	Frigoribacterium	Frigoribacterium sp. MCBA15_019	SAMN05736493	anthropogenic	11	Yes	4	FlgD;Flg_bbr_C;DUF4190;YitT_me	—
1522176.SAMN12025139.JAAOYV0100	clade11-A	329,3	high	Actinomycetota	Actinomycetes	Micrococcales	Microbacteriaceae	Frigoribacterium	Frigoribacterium endophyticum	SAMN12025139	host-associated	11	Yes	4	YitT_membrane;Mannosyl_trans2;F	—
2175619.SAMN09009058.QKSS010000	clade11-A	329,1	high	Actinomycetota	Actinomycetes	Micrococcales	Microbacteriaceae	Curtobacterium	Curtobacterium sp. MCJR17_020	SAMN09009058	anthropogenic	11	Yes	4	Bac_luciferase;Acyl-CoA_dh_M;Gly	—
1279028.SAMN05660766.OCMI010000	clade11-A	329	high	Actinomycetota	Actinomycetes	Micrococcales	Microbacteriaceae	Curtobacterium	Curtobacterium sp. 314Chir4.1	SAMN05660766	host-associated	11	Yes	4	Bac_luciferase;Acyl-CoA_dh_M;Mg	—
2017486.SAMN07299025.CP022296_4	clade11-A	328,7	high	Actinomycetota	Actinomycetes	Propionibacteriales	Nocardioidaceae	Nocardioides	Nocardioides sp. S5	SAMN07299025	aquatic	11	Yes	4	Acyltransferase;MerR-DNA-bind;Dn	—
1905847.SAMN05854879.CP017580_3	clade11-A	328,3	high	Actinomycetota	Actinomycetes	Micrococcales	Microbacteriaceae	Curtobacterium	Curtobacterium sp. BH-2-1-1	SAMN05854879	—	11	Yes	0	—	—
2485192.SAMN10361296.RKIE0100000	clade11-A	328,1	high	Actinomycetota	Actinomycetes	Micrococcales	Microbacteriaceae	Frigoribacterium	Frigoribacterium sp. PhB160	SAMN10361296	host-associated	7	Yes	3	Apolipoprotein;YitT_membrane;FlbD	—
1909295.SAMN08179254.PLES010001	clade11-A	327,1	high	Actinomycetota	Thermoleophilia	Solirubrobacterales	—	—	Solirubrobacterales bacterium	SAMN08179254	temperature	11	Yes	4	Cyt_bd_oxida_II;AHSA1;HTH_20	—
351215.SAMN14517837.JABEMA0100(	clade11-A	326,3	high	Actinomycetota	Actinomycetes	Kineosporiales	Kineosporiaceae	Pseudokineococcus	Pseudokineococcus marinus	SAMN14517837	anthropogenic	11	Yes	4	Glyoxalase;Glyoxalase_6;MST;MDI	—
2487580.SAMN16425975.JADJPO0100	clade11-A	323,3	high	Actinomycetota	Actinomycetes	Kineosporiales	Kineosporiaceae	—	Kineosporiaceae bacterium	SAMN16425975	anthropogenic	11	Yes	4	Cation_efflux;ROK;AP_endonuc_2	—
1735682.SAMN04151684.LMKK010000	clade11-A	320,6	high	Actinomycetota	Actinomycetes	Micrococcales	Microbacteriaceae	Agreia	Agreia sp. Leaf210	SAMN04151684	host-associated	11	Yes	4	TMEM260-like;AAA_2	—
477641.SAMEA2272685.FO203431_10	clade11-A	309,9	high	Actinomycetota	Actinomycetes	Geodermatophilales	Geodermatophilaceae	Modestobacter	Modestobacter marinus	SAMEA2272685	anthropogenic	11	Yes	4	Glyco_trans_1_2;LOR;Glyco_hydro	—
100233.SAMEA6946666.CAIVBC01000	clade11-A	306,1	high	Planctomycetota	Planctomycetia	Planctomycetales	Planctomycetaceae	—	uncultured Planctomycetaceae bacteri	SAMEA6946666	aquatic	3	Yes	2	WHD_3rd_SelB;ORF6C	—
100233.SAMEA6956128.CAIXVO01000	clade11-A	302,3	high	Planctomycetota	Planctomycetia	Planctomycetales	Planctomycetaceae	—	uncultured Planctomycetaceae bacteri	SAMEA6956128	aquatic	8	Yes	4	META;Thil_fer;GBD-like_ZMIZ1_Z	—
1218077.SAMD00000366.BAYC010000	clade11-A	300,9	high	Pseudomonadota	Betaproteobacteria	Burkholderiales	Burkholderiaceae	Paraburkholderia	Paraburkholderia fungorum	SAMD00000366	aquatic	11	Yes	4	Phage_GPD;GyrI-like;AA_permease	—
1884385.SAMN05518669.FNOL010000	clade11-A	300,3	high	Pseudomonadota	Betaproteobacteria	Burkholderiales	Comamonadaceae	Variovorax	Variovorax sp. YR634	SAMN05518669	host-associated	11	Yes	4	FAD_binding_3;SMI1_KNR4;Trp_d	—
2026791.SAMN08179553.PMKM01000	clade11-A	300,3	high	Acidobacteriota	Terriglobia	Bryobacterales	—	—	Bryobacterales bacterium	SAMN08179553	temperature	11	Yes	4	CxxCxxCC;ParE_like;SIS_2	—
2052164.SAMN08179277.PLDW01000(	clade11-A	299,5	high	Planctomycetota	Planctomycetia	Gemmatales	Gemmataceae	—	Gemmataceae bacterium	SAMN08179277	temperature	11	Yes	4	PGM_PMM_I	—
1191521.SAMEA6956431.CAIYZC0100	clade11-A	299,1	high	Verrucomicrobiota	Pedosphaerae	Pedosphaerales	Pedosphaeraceae	Pedosphaera	uncultured Pedosphaera sp.	SAMEA6956431	aquatic	3	Yes	2	Aconitase_C;Glyco_hydro_20b	—
2654182.SAMN17140642.JAFAZI01000	clade11-A	298,1	high	Actinomycetota	Actinomycetes	Frankiales	Frankiaceae	—	Frankiaceae bacterium	SAMN17140642	host-associated	11	Yes	4	Response_reg;Peptidase_S11;DUF	—
1913988.SAMN17181029.JAEUML010(	clade11-A	298	high	Pseudomonadota	Alphaproteobacteria	—	—	—	Alphaproteobacteria bacterium	SAMN17181029	anthropogenic	10	Yes	4	SET;Peptidase_M3;DUF308;Questi	—
1797564.SAMN04316052.MESG01000(	clade11-A	297,8	high	Pseudomonadota	Betaproteobacteria	Burkholderiales	—	—	Burkholderiales bacterium RIFCSPLO'	SAMN04316052	terrestrial	11	Yes	4	BPD_transp_1;PhyH;Aldedh;Amino	—
2026779.SAMEA104651807.OMIV0100	clade11-A	297	high	Planctomycetota	Planctomycetia	Planctomycetales	Planctomycetaceae	—	Planctomycetaceae bacterium	SAMEA104651807	aquatic	7	Yes	3	PGM_PMM_I;GFO_IDH_MocA_C	—
60549.SAMN05444165.FSRU01000002	clade11-A	296,3	high	Pseudomonadota	Betaproteobacteria	Burkholderiales	Burkholderiaceae	Paraburkholderia	Paraburkholderia phenazinium	SAMN05444165	host-associated	11	Yes	4	AtuA_ferredoxin;HpsJ;Polysacc_de	—
1882827.SAMN05443579.FOWG01000	clade11-A	295,8	high	Pseudomonadota	Betaproteobacteria	Burkholderiales	Comamonadaceae	Variovorax	Variovorax sp. PDC80	SAMN05443579	anthropogenic	11	Yes	4	FAD_binding_3;SMI1_KNR4;Trp_d	—
1781067.SAMN04320694.LRHV010000	clade11-A	295,8	high	Pseudomonadota	Betaproteobacteria	Burkholderiales	Oxalobacteraceae	Duganella	Duganella sp. HH105	SAMN04320694	host-associated	11	Yes	4	DUF885;Ricin_B_lectin;DUF945;Ep	—
1978231.SAMN08912219.QIAH010004	clade11-A	295,8	high	Acidobacteriota	—	—	—	—	Acidobacteriota bacterium	SAMN08912219	terrestrial	10	Yes	4	Cytochrom_C552;CMD;OMP_b-brl_	—
2026791.SAMN08178866.PMRN01000:	clade11-A	295,4	high	Acidobacteriota	Terriglobia	Bryobacterales	—	—	Bryobacterales bacterium	SAMN08178866	temperature	8	Yes	4	MacB_PCD;FitA-like_RHH	—
2026748.SAMN16635730.JADMJM010(	clade11-A	294,5	high	Pseudomonadota	Alphaproteobacteria	Hyphomonadales	Hyphomonadaceae	—	Hyphomonadaceae bacterium	SAMN16635730	terrestrial	11	Yes	4	HD;DAP_epimerase;zf-HC2	—
2502223.SAMN08712290.SCNV010000(	clade11-A	294,3	high	Pseudomonadota	Betaproteobacteria	Burkholderiales	Burkholderiaceae	Burkholderia	Burkholderia sp. 4M9327F10	SAMN08712290	aquatic	6	Yes	2	AtuA_ferredoxin;AtuA	—
2026791.SAMN08180001.PLRJ010000(	clade11-A	294,2	high	Acidobacteriota	Terriglobia	Bryobacterales	—	—	Bryobacterales bacterium	SAMN08180001	temperature	11	Yes	4	DUF433;Acetyltransf_1;DUF4890;E	—
2854789.SAMN19994155.JAHUWW01(	clade11-A	291,4	high	Pseudomonadota	Betaproteobacteria	Burkholderiales	Comamonadaceae	Acidovorax	Acidovorax sp. sif0632	SAMN19994155	terrestrial	11	Yes	4	Ni_hydr_CYTB;Acetyltransf_1;Redc	—
1121035.SAMN02441190.AUCH010000(	clade11-A	291	high	Pseudomonadota	Betaproteobacteria	Rhodocyclales	Rhodocyclaceae	Azovibrio	Azovibrio restrictus	SAMN02441190	host-associated	11	Yes	4	DXP_synthase_N;GCHY-1;Exo_en	—
2052484.SAMN07200918.NIOE010000(	clade11-A	290,7	high	Pseudomonadota	Betaproteobacteria	Burkholderiales	Sphaerotilaceae	Roseateles	Roseateles noduli	SAMN07200918	host-associated	11	Yes	4	Response_reg;2CSK_N;LysR_subs	—
48.SAMN07426514.QFQP01000062_1!	clade11-A	290,7	high	Myxococcota	Myxococcia	Myxococcales	Archangiaceae	Archangium	Archangium gephyra	SAMN07426514	anthropogenic	6	Yes	2	Pentapeptide_4	—
904984.SAMEA6955890.CAIOKO01000	clade11-A	289,3	low	Planctomycetota	Planctomycetia	Planctomycetales	Planctomycetaceae	Schlesneria	uncultured Schlesneria sp.	SAMEA6955890	aquatic	10	Yes	4	GFO_IDH_MocA;NIPSNAP;RtcB;H	—
946333.SAMN04621830.CP015118_73	clade11-A	288,8	low	Pseudomonadota	Betaproteobacteria	Burkholderiales	Sphaerotilaceae	Piscinibacter	Piscinibacter gummiphilus	SAMN04621830	host-associated	10	Yes	4	TauE;Hrs_helical;DUF4194	—
1801619.SAMN04515619.FOLC010000	clade11-A	288,3	low	Pseudomonadota	Betaproteobacteria	Burkholderiales	Oxalobacteraceae	Collimonas	Collimonas sp. OK412	SAMN04515619	host-associated	10	Yes	4	GTP_EFTU;GTP_cyclohydro2;PqiA	—
171953.SAMEA6946981.CAITVS01000	clade11-A	288,2	low	Acidobacteriota	—	—	—	—	uncultured Acidobacteriota bacterium	SAMEA6946981	aquatic	9	Yes	4	OMP_b-brl_4;Ank_5;tRNA-synt_1_	—
2682146.SAMN16425753.JADJHQ0100	clade11-A	287,8	low	Pseudomonadota	Betaproteobacteria	Nitrosomonadales	Sterolibacteriaceae	—	Sterolibacteriaceae bacterium	SAMN16425753	anthropogenic	10	Yes	4	DUF4372;KH_NucS_shadow;EAL	—
2004485.SAMN07156299.NOIG010000	clade11-A	284,8	low	Pseudomonadota	Betaproteobacteria	Burkholderiales	Comamonadaceae	Acidovorax	Acidovorax kalamii	SAMN07156299	aquatic	10	Yes	4	Ni_hydr_CYTB;Acetyltransf_1;Redc	—
2052142.SAMN08179306.PLCU010001	clade11-A	284,7	low	Acidobacteriota	Terriglobia	Terriglobales	Acidobacteriaceae	—	Acidobacteriaceae bacterium	SAMN08179306	temperature	10	Yes	4	Lactamase_B;Dus;Ribosomal_L31	—
2026791.SAMN08178945.PMUN01000:	clade11-A	284,7	low	Acidobacteriota	Terriglobia	Bryobacterales	—	—	Bryobacterales bacterium	SAMN08178945	temperature	10	Yes	4	PMSR;Aminotran_3;TehB	—
2587122.SAMN12024204.JACHYS0100	clade11-A	284,2	low	Pseudomonadota	Betaproteobacteria	Burkholderiales	Sphaerotilaceae	Roseateles	Mitsuaria sp. BK037	SAMN12024204	host-associated	10	Yes	4	DUF488-N3a;LysR_substrate;Abhy	—
2838466.SAMN15816783.DXIO010001	clade11-A	284,2	low	Pseudomonadota	Betaproteobacteria	Burkholderiales	—	Aquabacterium	Candidatus Aquabacterium excrementi	SAMN15816783	host-associated	10	Yes	4	DUF5691;SWIM;HTH_18;Isochoris	—
156588.SAMEA6944372.CAIMXY01000	clade11-A	282,8	low	Verrucomicrobiota	—	—	—	—	uncultured Verrucomicrobiota bacteriu	SAMEA6944372	aquatic	9	Yes	4	Zn_ribbon_4;Sigma70_r2;FecR	—
259537.SAMN17348697.JAFIHA01000(	clade11-A	282,3	low	Pseudomonadota	Betaproteobacteria	Rhodocyclales	Azonexaceae	Dechloromonas	Dechloromonas aromatica (nom. nud.)	SAMN17348697	host-associated	6	Yes	3	TerC;tRNA-synt_1b;ThermoDBP-R	—
1871071.SAMN18061362.JAFKGX0100	clade11-A	281,5	low	Pseudomonadota	Betaproteobacteria	Burkholderiales	Comamonadaceae	—	Comamonadaceae bacterium	SAMN18061362	host-associated	10	Yes	4	Ribosomal_S17;Redoxin;Acetyltrans	—
2026791.SAMN08178902.PMSX010000(	clade11-A	281,5	low	Acidobacteriota	Terriglobia	Bryobacterales	—	—	Bryobacterales bacterium	SAMN08178902	temperature	10	Yes	4	Beta-prop_THOC3;PSD4	—
2026779.SAMN11369404.SYJV010002	clade11-A	281,5	low	Planctomycetota	Planctomycetia	Planctomycetales	Planctomycetaceae	—	Planctomycetaceae bacterium	SAMN11369404	aquatic	6	Yes	3	Sulfatase;DPBB_PEX6;SGSH_C	—
2026791.SAMN08178783.PMOK01000(	clade11-A	280,6	low	Acidobacteriota	Terriglobia	Bryobacterales	—	—	Bryobacterales bacterium	SAMN08178783	—	10	Yes	0	—	—
1978231.SAMN14214674.JABCZI0100(	clade11-A	277,7	low	Acidobacteriota	—	—	—	—	Acidobacteriota bacterium	SAMN14214674	terrestrial	10	Yes	4	Dus;AIM24;Lactamase_B;AAA_2	—
2026799.SAMN14913714.JABGQM010	clade11-A	277	low	Verrucomicrobiota	—	—	—	—	Verrucomicrobiota bacterium	SAMN14913714	aquatic	10	Yes	3	ABC2_membrane_3;SBP_bac_10;	—
2053492.SAMN18059897.JAFLDO0100	clade11-A	272,3	low	Pseudomonadota	Betaproteobacteria	—	—	Candidatus Accumulib	Accumulibacter sp.	SAMN18059897	anthropogenic	10	Yes	4	PacC_helical;Response_reg;HATP	—
1913988.SAMN18118915.JAGNKE010(	clade11-A	271,6	low	Pseudomonadota	Alphaproteobacteria	—	—	—	Alphaproteobacteria bacterium	SAMN18118915	anthropogenic	10	Yes	4	GtfA_EBD;Pro_CA;MASE5;UDG	—
1978231.SAMN14214676.JABCZK0100	clade11-A	271,4	low	Acidobacteriota	—	—	—	—	Acidobacteriota bacterium	SAMN14214676	terrestrial	10	Yes	4	HTH_8;SAICAR_synt;SMC_N	—
2026791.SAMN08178828.PMQB01000	clade11-A	269,7	low	Acidobacteriota	Terriglobia	Bryobacterales	—	—	Bryobacterales bacterium	SAMN08178828	temperature	5	Yes	3	Lipase_GDSL_2;HHH_3;Beta-prop_	—

86027.SAMEA6956982.CAIMXF01000	**clade11-A**	269,1	low	Pseudomonadota	Betaproteobacteria	—	—	—	uncultured beta proteobacterium	SAMEA6956982	aquatic	10	Yes	3	MFS_1;Phenol_MetA_deg	—
1632864.SAMN03417854.CP011270_3	**clade11-A**	268,8	low	Planctomycetota	Planctomycetia	Planctomycetales	Planctomycetaceae	Planctomyces	Planctomyces sp. SH-PL14	SAMN03417854	anthropogenic	10	Yes	4	CENP-F_leu_zip;SIS;Reprolysin_3;	—
2072747.SAMN08366045.RCHM01000	**clade11-A**	268,2	low	Pseudomonadota	Gammaproteobacteria	Pseudomonadales	Ketobacteraceae	Ketobacter	Ketobacter sp. GenoA1	SAMN08366045	aquatic	10	Yes	4	Acyl-CoA_dh_1;ChaC;DUF4234;Al	—
373675.SAMN04488045.FNUZ0100000	**clade11-A**	265,1	low	Pseudomonadota	Alphaproteobacteria	Rhodobacterales	Roseobacteraceae	Thalassococcus	Thalassococcus halodurans	SAMN04488045	aquatic	10	Yes	4	Pyr_redox_2;Dioxygenase_C;Sacch	—
2591109.SAMN12116251.CP041352_3	**clade11-A**	263,6	low	Pseudomonadota	Betaproteobacteria	—	Casimicrobiaceae	Casimicrobium	Casimicrobium huifangae	SAMN12116251	anthropogenic	10	Yes	4	HMGL-like;2-Hacid_dh_C;CPSase_	—
100234.SAMEA9694258.CAJXQE0100	**clade11-A**	260	low	Verrucomicrobiota	Verrucomicrobiia	Verrucomicrobiales	—	—	uncultured Verrucomicrobiales bacteriu	SAMEA9694258	temperature	7	Yes	4	SASA;Sulfatase;LRR_14;Beta-prop	—
2595004.SAMN12273953.VKKX010000	**clade11-A**	253,7	low	Pseudomonadota	Alphaproteobacteria	Rhodobacterales	Paracoccaceae	Gemmobacter	Gemmobacter caeruleus	SAMN12273953	anthropogenic	10	Yes	4	Acetyltransf_1;GFA;ABC_tran;MipZ	—
1891241.SAMN11380348.VBCQ01000	**clade11-A**	233,9	low	Pseudomonadota	Betaproteobacteria	—	—	—	Betaproteobacteria bacterium	SAMN11380348	terrestrial	7	Yes	4	GTP_EFTU;UbiA;ELFV_dehydrog	—
1978231.SAMN10966252.VFZW01000	**clade11-A**	214,2	low	Acidobacteriota	—	—	—	—	Acidobacteriota bacterium	SAMN10966252	aquatic	5	Yes	2	PQQ_2;Vgb_lyase	—
1314686.SAMN06349878.MVKV01000	**clade11-B**	336,2	high	Actinomycetota	Actinomycetes	Mycobacteriales	Gordoniaceae	Gordonia	Gordonia sp. IITR100	SAMN06349878	host-associated	9	Yes	4	ABC_tran;Acetyltransf_1;Globin;GT	—
1136179.SAMN02603403.CP003761_5	**clade11-B**	319	high	Actinomycetota	Actinomycetes	Mycobacteriales	Nocardiaceae	Rhodococcus	Rhodococcus erythropolis	SAMN02603403	—	11	Yes	0	—	—
1112204.SAMN02603285.CP003119_4	**clade11-B**	319	high	Actinomycetota	Actinomycetes	Mycobacteriales	Gordoniaceae	Gordonia	Gordonia polyisoprenivorans	SAMN02603285	aquatic	11	Yes	4	Acyl_transf_3;CorA;HNH;Sulfotrans	—
1219016.SAMD00047211.BCWX01000	**clade11-B**	318,8	high	Actinomycetota	Actinomycetes	Mycobacteriales	Nocardiaceae	Rhodococcus	Rhodococcus globerulus	SAMD00047211	anthropogenic	11	Yes	4	Aldolase_II;NMT1;TetR_N;ECH_1	—
2739471.SAMN15016254.JABUDE010	**clade11-B**	318,2	high	Actinomycetota	Actinomycetes	Mycobacteriales	Nocardiaceae	Rhodococcus	Rhodococcus sp. BP-369	SAMN15016254	aquatic	11	Yes	4	Flavin_Reduct;VanW;Thiolase_N;a	—
1051662.SAMN02569993.JMEX010000	**clade11-B**	315,3	high	Actinomycetota	Actinomycetes	Mycobacteriales	Nocardiaceae	Rhodococcoides	Rhodococcoides fascians	SAMN02569993	host-associated	11	Yes	4	Beta-lactamase;FAD-oxidase_C;adh	—
1108044.SAMD00041778.BAFB010000	**clade11-B**	314,1	high	Actinomycetota	Actinomycetes	Mycobacteriales	Gordoniaceae	Gordonia	Gordonia otitidis	SAMD00041778	anthropogenic	11	Yes	4	EryCIII-like_C;DUF3046;RecA_N;R	—
1219362.SAMD00046772.BDAP01000	**clade11-B**	314	high	Actinomycetota	Actinomycetes	Mycobacteriales	Nocardiaceae	Williamsia	Williamsia muralis	SAMD00046772	anthropogenic	11	Yes	4	Ala_racemase_N;DNA_pol3_alpha;	—
2806442.SAMN17526040.CP069242_2	**clade11-B**	313,4	high	Actinomycetota	Actinomycetes	Mycobacteriales	Nocardiaceae	Rhodococcus	Rhodococcus sp. USK13	SAMN17526040	host-associated	11	Yes	4	Polyketide_cyc2;PLDc_N;TetR_N;F	—
498198.SAMN06265174.FXTG0100001	**clade11-B**	312,7	high	Actinomycetota	Actinomycetes	Mycobacteriales	Dietziaceae	Dietzia	Dietzia kunjamensis	SAMN06265174	anthropogenic	6	Yes	2	DUF4131;DUF4077	—
194249.SAMEA9694981.CAJXRT0100	**clade11-B**	311,9	high	Actinomycetota	Actinomycetes	Mycobacteriales	Nocardiaceae	Rhodococcus	uncultured Rhodococcus sp.	SAMEA9694981	aquatic	11	Yes	4	GyrI-like;HTH_3;AzlC	—
2838538.SAMN15816639.DWWP0100	**clade11-B**	311,4	high	Actinomycetota	Actinomycetes	Mycobacteriales	Dietziaceae	Dietzia	Candidatus Dietzia intestinipullorum	SAMN15816639	host-associated	7	Yes	3	DUF2339;Guanylate_cyc	—
632772.SAMD00060964.AP011115_53	**clade11-B**	310,7	high	Actinomycetota	Actinomycetes	Mycobacteriales	Nocardiaceae	Rhodococcus	Rhodococcus opacus	SAMD00060964	host-associated	11	Yes	4	Nitroreductase;adh_short_C2;DUF3	—
1089453.SAMD00041776.BAFC010000	**clade11-B**	309,2	high	Actinomycetota	Actinomycetes	Mycobacteriales	Gordoniaceae	Gordonia	Gordonia sputi	SAMD00041776	anthropogenic	11	Yes	4	EryCIII-like_C;DUF3046;RecA_N;R	—
1220583.SAMD00041806.BANR01000	**clade11-B**	308,7	high	Actinomycetota	Actinomycetes	Mycobacteriales	Gordoniaceae	Gordonia	Gordonia aichiensis	SAMD00041806	anthropogenic	11	Yes	4	EryCIII-like_C;DUF3046;RecA_N;R	—
2838537.SAMN15816635.DWWG0100	**clade11-B**	304,2	high	Actinomycetota	Actinomycetes	Mycobacteriales	Dietziaceae	Dietzia	Candidatus Dietzia intestinigallinarum	SAMN15816635	host-associated	11	Yes	4	EamA;FUSC_2;PadR;HAAS	—
57704.SAMN04457857.LSRG0100001	**clade11-B**	296,5	high	Actinomycetota	Actinomycetes	Mycobacteriales	Tsukamurellaceae	Tsukamurella	Tsukamurella tyrosinosolvens	SAMN04457857	host-associated	11	Yes	4	Isochorismatase;HTH_18;HAD_2;H	—
47312.SAMN04457854.LSRH0100004	**clade11-B**	291,9	high	Actinomycetota	Actinomycetes	Mycobacteriales	Tsukamurellaceae	Tsukamurella	Tsukamurella pulmonis	SAMN04457854	host-associated	11	Yes	4	HAD_2;DUF2157;Acetyltransf_1	—
100233.SAMEA6952416.CAIOTJ01000	**clade11-B**	276,6	low	Planctomycetota	Planctomycetia	Planctomycetales	Planctomycetaceae	—	uncultured Planctomycetaceae bacteri	SAMEA6952416	aquatic	10	Yes	4	Response_reg;Sacchrp_dh_NADP;	—
100234.SAMEA9695074.CAJXVQ0100	**clade11-B**	273,5	low	Verrucomicrobiota	Verrucomicrobiia	Verrucomicrobiales	—	—	uncultured Verrucomicrobiales bacteriu	SAMEA9695074	temperature	10	Yes	4	2Fe-2S_thioredx;DNA_pol3_delta;P	—
2026780.SAMN15870312.JACTNB010	**clade11-B**	265,4	low	Planctomycetota	—	—	—	—	Planctomycetota bacterium	SAMN15870312	anthropogenic	10	Yes	4	WCX;Abhydrolase_6;PFK;Hydrolas	—
756272.SAMN00103628.CP002546_23	**clade11-B**	257,4	low	Planctomycetota	Planctomycetia	Planctomycetales	Planctomycetaceae	Rubinisphaera	Rubinisphaera brasiliensis	SAMN00103628	aquatic	10	Yes	4	IMPDH;RrnaAD;PFK;Hydrolase	—
904984.SAMEA6954088.CAIWEX0100	**clade11-B**	255,9	low	Planctomycetota	Planctomycetia	Planctomycetales	Planctomycetaceae	Schlesneria	uncultured Schlesneria sp.	SAMEA6954088	temperature	2	Yes	2	DUF1501	—
904984.SAMEA6951781.CAIKKW0100	**clade11-B**	255,5	low	Planctomycetota	Planctomycetia	Planctomycetales	Planctomycetaceae	Schlesneria	uncultured Schlesneria sp.	SAMEA6951781	temperature	5	Yes	2	Glycos_transf_3;Abhydrolase_2	—
904984.SAMEA6956427.CAIMFH0100	**clade11-B**	251,9	low	Planctomycetota	Planctomycetia	Planctomycetales	Planctomycetaceae	Schlesneria	uncultured Schlesneria sp.	SAMEA6956427	aquatic	10	Yes	4	MSMEG_6518_N;Peptidase_M50;I	—
904984.SAMEA6954856.CAIQIR01000	**clade11-B**	251,4	low	Planctomycetota	Planctomycetia	Planctomycetales	Planctomycetaceae	Schlesneria	uncultured Schlesneria sp.	SAMEA6954856	aquatic	10	Yes	4	PseudoU_synth_2;Vma12;RsfS	—
2052181.SAMD00166097.BJHP010000	**clade11-B**	248,7	low	Planctomycetota	Planctomycetia	—	—	—	Planctomycetia bacterium	SAMD00166097	aquatic	10	Yes	4	HPPK;Peptidase_M16_C;ATP_binc	—
2026780.SAMN15013952.JABTUM010	**clade11-B**	248,6	low	Planctomycetota	—	—	—	—	Planctomycetota bacterium	SAMN15013952	temperature	10	Yes	3	TPR_19;Inositol_P;SGL	—
2026780.SAMN10966916.VGZH01000	**clade11-B**	248,5	low	Planctomycetota	—	—	—	—	Planctomycetota bacterium	SAMN10966916	aquatic	10	Yes	4	DUF1501;ATP_bind_3;Peptidase_I	—
2026779.SAMN14915429.JABJDJ0100	**clade11-B**	245,3	low	Planctomycetota	Planctomycetia	Planctomycetales	Planctomycetaceae	—	Planctomycetaceae bacterium	SAMN14915429	aquatic	10	Yes	4	GFO_IDH_MocA;AP_endonuc_2;S	—
904984.SAMEA6955702.CAIULL01000	**clade11-B**	242,9	low	Planctomycetota	Planctomycetia	Planctomycetales	Planctomycetaceae	Schlesneria	uncultured Schlesneria sp.	SAMEA6955702	—	10	Yes	0	—	—
904984.SAMEA6955890.CAIOKO0100	**clade11-B**	242,6	low	Planctomycetota	Planctomycetia	Planctomycetales	Planctomycetaceae	Schlesneria	uncultured Schlesneria sp.	SAMEA6955890	aquatic	10	Yes	0	—	—
904984.SAMEA6943704.CAIVEI010000	**clade11-B**	240,1	low	Planctomycetota	Planctomycetia	Planctomycetales	Planctomycetaceae	Schlesneria	uncultured Schlesneria sp.	SAMEA6943704	aquatic	6	Yes	3	MJ0013;CHAT;SBP_bac_10	—
2026778.SAMN13893722.JAAYDY0100	**clade11-B**	227,4	low	Planctomycetota	Phycisphaerae	—	—	—	Phycisphaerae bacterium	SAMN13893722	anthropogenic	6	Yes	3	Phage_tail_beta;Beta-prop_IFT122_	—
2026780.SAMN18118927.JAGNKM010	**clade11-B**	215,1	low	Planctomycetota	—	—	—	—	Planctomycetota bacterium	SAMN18118927	anthropogenic	10	Yes	4	LRR_14;Beta-prop_IFT122_1st;FG	—
2026778.SAMD00166093.BJHL010000	**clade11-B**	188,1	low	Planctomycetota	Phycisphaerae	—	—	—	Phycisphaerae bacterium	SAMD00166093	aquatic	10	Yes	4	GT87;RNase_HII;Thymidylate_kin;E	—
153809.SAMEA6949946.CAIMSU0100	**clade11-B**	179,7	low	Pseudomonadota	—	—	—	—	uncultured Pseudomonadota bacterium	SAMEA6949946	aquatic	6	Yes	3	—	—
2026724.SAMN20307496.JAIOAS0100	**clade11-B**	174,6	low	Chloroflexota	—	—	—	—	Chloroflexota bacterium	SAMN20307496	temperature	9	Yes	4	Band_7;Peripla_BP_3;Response_re	—
1895807.SAMN05660562.MKTT010000	**clade11-B**	158,3	low	Planctomycetota	Planctomycetia	Planctomycetales	—	—	Planctomycetales bacterium 71-10	SAMN05660562	anthropogenic	10	Yes	4	MMPL;Band_7;DUF6298	—
2044587.SAMN01731007.KB822441_6	**clade2_Ec107like**	322,5	high	Bacillota	Clostridia	Lachnospirales	Lachnospiraceae	Schaedlerella	Schaedlerella arabinosiphila	SAMN01731007	host-associated	11	Yes	4	ECF-ribofla_trS;tRNA-synt_1b;BcrA	—
297314.SAMEA8805696.CAJULL0100	**clade2_Ec107like**	321,7	high	Bacillota	Clostridia	Lachnospirales	Lachnospiraceae	—	uncultured Lachnospiraceae bacterium	SAMEA8805696	host-associated	11	Yes	4	DUF3267;tRNA-synt_1b;Glyco_hyc	—
1121114.SAMN02441708.KB892637_1	**clade2_Ec107like**	321	high	Bacillota	Clostridia	Lachnospirales	Lachnospiraceae	Blautia	Blautia producta	SAMN02441708	host-associated	11	Yes	4	F420_ligase;Metallophos_2;Ham1p_	—
2025493.SAMN07460455.NQOF01000	**clade2_Ec107like**	319,1	high	Bacillota	Clostridia	Lachnospirales	Lachnospiraceae	Blautia	Blautia hominis	SAMN07460455	host-associated	11	Yes	4	Response_reg;Lipocalin_4;DUF740	—
1898203.SAMEA8801244.CAJSZT010	**clade2_Ec107like**	319	high	Bacillota	Clostridia	Lachnospirales	Lachnospiraceae	—	Lachnospiraceae bacterium	SAMEA8801244	host-associated	11	Yes	4	ECF-ribofla_trS;tRNA-synt_1b;FtsX	—
1745713.SAMEA3959520.LT574838_1	**clade2_Ec107like**	319	high	Bacillota	Clostridia	Lachnospirales	Lachnospiraceae	Bariatricus	Bariatricus massiliensis	SAMEA3959520	host-associated	11	Yes	4	Glycos_transf_2;MazG;adh_short;A	—
1410651.SAMN02743875.JHWJ010000	**clade2_Ec107like**	318,8	high	Bacillota	Clostridia	Lachnospirales	Lachnospiraceae	Lacrimispora	Lacrimispora aerotolerans	SAMN02743875	host-associated	11	Yes	4	PDH_N;DAHP_synth_1;Choline_bi	—
1946596.SAMN06450376.DGRE01000	**clade2_Ec107like**	318,6	high	Bacillota	Clostridia	Lachnospirales	Lachnospiraceae	Hungatella	Hungatella sp. UBA4396	SAMN06450376	anthropogenic	11	Yes	4	PDH_N;DAHP_synth_1;Choline_bi	—
94868.SAMN05504968.MCIA01000032	**clade2_Ec107like**	317,5	high	Bacillota	Clostridia	Lachnospirales	Lachnospiraceae	Lacrimispora	Lacrimispora algidixylanolytica	SAMN05504968	anthropogenic	11	Yes	4	PDH_N;DAHP_synth_1;Choline_bi	—
1898203.SAMN09901047.QXWY01000	**clade2_Ec107like**	317,4	high	Bacillota	Clostridia	Lachnospirales	Lachnospiraceae	—	Lachnospiraceae bacterium	SAMN09901047	host-associated	11	Yes	4	adh_short;tRNA-synt_1b;ECF-ribofl	—
1111728.SAMN02440598.ATYS010000	**clade2_Ec107like**	317,3	high	Pseudomonadota	Gammaproteobacteria	Enterobacterales	Budviciaceae	Budvicia	Budvicia aquatica	SAMN02440598	host-associated	11	Yes	4	HD;Diacid_rec;GD_AH_C;MFS_1	—
1965569.SAMN06473635.NFKT010000	**clade2_Ec107like**	315,7	high	Bacillota	Clostridia	Lachnospirales	Lachnospiraceae	Lachnoclostridium	Lachnoclostridium sp. An169	SAMN06473635	host-associated	11	Yes	4	DegV;DHDPS;FAD_binding_5;Fer2	—
904190.SAMEA5278550.CAAEFH0100	**clade2_Ec107like**	315,6	high	Bacillota	Clostridia	Lachnospirales	Lachnospiraceae	Robinsoniella	uncultured Robinsoniella sp.	SAMEA5278550	host-associated	11	Yes	4	Glyco_hydro_77;Acyltransferase;Fts	—
537007.SAMN07340700.CP022413_28	**clade2_Ec107like**	315,3	high	Bacillota	Clostridia	Lachnospirales	Lachnospiraceae	Blautia	Blautia hansenii	SAMN07340700	host-associated	11	Yes	4	CBM_48;TetR_N;TatD_DNase;AI-2	—
297314.SAMEA7848117.CAJKQE0100	**clade2_Ec107like**	315,3	high	Bacillota	Clostridia	Lachnospirales	Lachnospiraceae	—	uncultured Lachnospiraceae bacterium	SAMEA7848117	host-associated	11	Yes	0	—	—
1946599.SAMN06457310.DIJQ010000	**clade2_Ec107like**	314,5	high	Bacillota	Clostridia	Lachnospirales	Lachnospiraceae	Hungatella	Hungatella sp. UBA5640	SAMN06457310	anthropogenic	11	Yes	4	His_kinase;Response_reg;DAHP_s	—
904190.SAMEA5849789.CABPVW010	**clade2_Ec107like**	314,4	high	Bacillota	Clostridia	Lachnospirales	Lachnospiraceae	Robinsoniella	uncultured Robinsoniella sp.	SAMEA5849789	host-associated	11	Yes	4	Glyco_hydro_77;Acyltransferase;Fts	—
1232440.SAMD00008809.BAHR02000	**clade2_Ec107like**	314	high	Bacillota	Clostridia	Lachnospirales	Lachnospiraceae	Hungatella	Hungatella hathewayi	SAMD00008809	host-associated	11	Yes	4	2TM_P5A-ATPase;DUF2871;EPSP	—
33035.SAMN02903323.JPJF01000011_	**clade2_Ec107like**	314	high	Bacillota	Clostridia	Lachnospirales	Lachnospiraceae	Blautia	Blautia producta	SAMN02903323	host-associated	11	Yes	4	DinB_2;Hydrolase_4;Condensation;	—
742740.SAMN02463857.GL834320_36	**clade2_Ec107like**	313,9	high	Bacillota	Clostridia	Lachnospirales	Lachnospiraceae	Otoolea	[Clostridium] symbiosum	SAMN02463857	host-associated	11	Yes	4	GFO_IDH_MocA;LysR_substrate;N	—
904190.SAMEA4890949.UQGT010000	**clade2_Ec107like**	313,3	high	Bacillota	Clostridia	Lachnospirales	Lachnospiraceae	Robinsoniella	uncultured Robinsoniella sp.	SAMEA4890949	host-associated	10	Yes	4	ABC_tran;FtsX;Acyltransferase;Gly	—
297314.SAMEA7202538.CAJFUP0100	**clade2_Ec107like**	312,1	high	Bacillota	Clostridia	Lachnospirales	Lachnospiraceae	—	uncultured Lachnospiraceae bacterium	SAMEA7202538	host-associated	9	Yes	4	PTS-HPr;DUF6264;Adenylsucc_sy	—
2559709.SAMN10863240.SPHP010000	**clade2_Ec107like**	312	high	Bacillota	Clostridia	Eubacteriales	Clostridiaceae	Clostridium	Clostridium sp. 1001175st1 A10	SAMN10863240	host-associated	11	Yes	4	PDH_N;EPSP_synthase;GDE_C;L	—
297314.SAMEA8805883.CAJUTU0100	**clade2_Ec107like**	311,7	high	Bacillota	Clostridia	Lachnospirales	Lachnospiraceae	—	uncultured Lachnospiraceae bacterium	SAMEA8805883	host-associated	11	Yes	4	RraA-like;GFO_IDH_MocA;DUF60	—
1304881.SAMN02597187.AZUI0100000	**clade2_Ec107like**	311,4	high	Bacillota	Clostridia	Lachnospirales	Lachnospiraceae	Lacrimispora	Lacrimispora indolis	SAMN02597187	host-associated	11	Yes	4	His_kinase;Response_reg;DAHP_s	—
1286362.SAMN02745763.PGET010000	**clade2_Ec107like**	311	high	Bacillota	Clostridia	Lachnospirales	Lachnospiraceae	Lacrimispora	Lacrimispora celerecrescens	SAMN02745763	host-associated	11	Yes	0	—	—
765821.SAMEA7848047.CAJJVI01000	**clade2_Ec107like**	311	high	Bacillota	Clostridia	Lachnospirales	Lachnospiraceae	Blautia	uncultured Blautia sp.	SAMEA7848047	host-associated	7	Yes	3	GWT1;Alpha-amylase;ToxN_toxin	—
500632.SAMN00000730.DS995353_83	**clade2_Ec107like**	310,7	high	Bacillota	Clostridia	Lachnospirales	Lachnospiraceae	Faecalimonas	Faecalimonas nexilis	SAMN00000730	host-associated	11	Yes	4	Topoisom_bac;BcrAD_BadFG;Pseu	—
1720294.SAMN09736885.QVLV010000	**clade2_Ec107like**	309,8	high	Bacillota	Clostridia	Lachnospirales	Lachnospiraceae	Eisenbergiella	Eisenbergiella massiliensis	SAMN09736885	host-associated	11	Yes	4	Glyoxalase;Metallophos;Flg_new;D	—
1291051.SAMD00000437.BBAB010000	**clade2_Ec107like**	309,6	high	Bacillota	Clostridia	Lachnospirales	Lachnospiraceae	Mediterraneibacter	Mediterraneibacter glycyrrhizinilyticus	SAMD00000437	host-associated	11	Yes	4	Glyco_hydro_3;Glyco_hydro_79n	—
1898203.SAMEA7848093.CAJKQB010	**clade2_Ec107like**	309,4	high	Bacillota	Clostridia	Lachnospirales	Lachnospiraceae	—	Lachnospiraceae bacterium	SAMEA7848093	host-associated	11	Yes	4	HATPase_c;Response_reg;Metallo	—

59620.SAMEA4890773.UPZT01000002	**clade2_Ec107like**	309	high	Bacillota	Clostridia	Eubacteriales	Clostridiaceae	Clostridium	uncultured Clostridium sp.	SAMEA4890773	host-associated	11	Yes	4	Hpt;HD_5;DAPDH_C;tRNA_edit	—
2763667.SAMN15805280.CP060635_1	**clade2_Ec107like**	308,9	high	Bacillota	Clostridia	Lachnospirales	Lachnospiraceae	Wansuia	Wansuia hejianensis	SAMN15805280	host-associated	11	Yes	4	HTH_18;adh_short_C2;GFO_IDH_	—
1232458.SAMD00008692.BAIL0200001	**clade2_Ec107like**	307,8	high	Bacillota	Clostridia	Eubacteriales	—	—	Clostridiales bacterium VE202-29	SAMD00008692	host-associated	11	Yes	4	DAPDH_C;Flg_new;SMAGP;tRNA_	—
77133.SAMEA7852881.CAJMPJ01000(	**clade2_Ec107like**	307,7	high	—	—	—	—	—	uncultured bacterium	SAMEA7852881	—	11	Yes	4	PDH_N;DAHP_synth_1;Choline_bi	—
457421.SAMN02463678.DS990263_33	**clade2_Ec107like**	306	high	Bacillota	Clostridia	Eubacteriales	—	—	Clostridiales bacterium 1_7_47FAA	SAMN02463678	host-associated	11	Yes	0	—	—
358742.SAMN09734406.QVEX0100000	**clade2_Ec107like**	305,8	high	Bacillota	Clostridia	Lachnospirales	Lachnospiraceae	Enterocloster	Enterocloster aldenensis	SAMN09734406	host-associated	11	Yes	4	TetR_N;adh_short;AIM24;Cons_hy	—
297314.SAMEA8805860.CAJUTQ0100(	**clade2_Ec107like**	305,8	high	Bacillota	Clostridia	Lachnospirales	Lachnospiraceae	—	uncultured Lachnospiraceae bacterium	SAMEA8805860	host-associated	9	Yes	3	Acyl_transf_3;TatD_DNase;RmaAD	—
1123075.SAMN02441541.AUDP01000(	**clade2_Ec107like**	305,7	high	Bacillota	Clostridia	Eubacteriales	Oscillospiraceae	Ruminococcus	Ruminococcus gauvreauii	SAMN02441541	host-associated	11	Yes	4	GMP_synt_C;AbiTii;MTTB;AP_end	—
2838502.SAMN15816793.DWUX01000	**clade2_Ec107like**	305,3	high	Bacillota	Clostridia	Lachnospirales	Lachnospiraceae	Blautia	Candidatus Blautia stercoripullorum	SAMN15816793	host-associated	11	Yes	4	AI-2E_transport;TatD_DNase;Alpha	—
742733.SAMN02463853.JH376424_86	**clade2_Ec107like**	304,2	high	Bacillota	Clostridia	Lachnospirales	Lachnospiraceae	Enterocloster	Enterocloster citroniae	SAMN02463853	host-associated	11	Yes	4	TetR_N;adh_short;AIM24;Cons_hy	—
1796618.SAMN10864764.SGXF010000	**clade2_Ec107like**	303,9	high	Bacillota	Clostridia	Lachnospirales	Lachnospiraceae	Cuneatibacter	Cuneatibacter caecimuris	SAMN10864764	host-associated	11	Yes	4	Transketolase_N;MerR_1;APH;DHI	—
411902.SAMN00627070.DS480695_87	**clade2_Ec107like**	303,4	high	Bacillota	Clostridia	Lachnospirales	Lachnospiraceae	Enterocloster	Enterocloster bolteae	SAMN00627070	host-associated	11	Yes	4	Flavoprotein;YoaP;BPD_transp_1	—
2559708.SAMN10863263.SPHO01000(	**clade2_Ec107like**	303,3	high	Bacillota	Clostridia	Eubacteriales	Clostridiaceae	Clostridium	Clostridium sp. 1001271st1 H5	SAMN10863263	host-associated	11	Yes	4	BPD_transp_1;Flavoprotein;DpaA_I	—
1912897.SAMN09487132.CP030280_3	**clade2_Ec107like**	302,9	high	Bacillota	Clostridia	Lachnospirales	Lachnospiraceae	Blautia	Blautia argi	SAMN09487132	host-associated	11	Yes	4	Glyco_hydro_77;Zn_peptidase_2;C	—
1898203.SAMN17800811.JAGZGI0100	**clade2_Ec107like**	302,9	high	Bacillota	Clostridia	Lachnospirales	Lachnospiraceae	—	Lachnospiraceae bacterium	SAMN17800811	host-associated	11	Yes	4	TetR_N;SpoIIID;Glyco_hydro_4	—
1122155.SAMN02745158.FQVI0100001	**clade2_Ec107like**	301,4	high	Bacillota	Clostridia	Eubacteriales	Clostridiaceae	Lactonifactor	Lactonifactor longoviformis	SAMN02745158	host-associated	11	Yes	4	Ldh_1_N;Malic_M;FAA_hydrolase;I	—
562.SAMN14342434.CP050195_3503	**clade2_Ec107like**	301,3	high	Pseudomonadota	Gammaproteobacteria	Enterobacterales	Enterobacteriaceae	Escherichia	Escherichia coli	SAMN14342434	host-associated	11	Yes	4	dUTPase;SlmA-like_C;Pribosyltran;	—
165186.SAMEA7202183.CAJFQJ01000(	**clade2_Ec107like**	300,7	high	Bacillota	Clostridia	Eubacteriales	Oscillospiraceae	Ruminococcus	uncultured Ruminococcus sp.	SAMEA7202183	host-associated	6	Yes	2	Fer2_2;FAD_binding_5	—
297314.SAMEA8805492.CAJUFN01000(	**clade2_Ec107like**	300,7	high	Bacillota	Clostridia	Lachnospirales	Lachnospiraceae	—	uncultured Lachnospiraceae bacterium	SAMEA8805492	host-associated	11	Yes	4	Zn_ribbon_2;GyrI-like;GIY-YIG;SseI	—
297314.SAMEA7202275.CAJFIC01000(	**clade2_Ec107like**	300,5	high	Bacillota	Clostridia	Lachnospirales	Lachnospiraceae	—	uncultured Lachnospiraceae bacterium	SAMEA7202275	host-associated	11	Yes	4	PTS-HPr;DUF6264;Adenylsucc_sy	—
1879010.SAMN17800882.JAGZJB0100	**clade2_Ec107like**	300,2	high	Bacillota	—	—	—	—	Bacillota bacterium	SAMN17800882	host-associated	11	Yes	4	Peptidase_M17;X25_BaPul_like;Fo	—
297314.SAMEA7202512.CAJFNX0100(	**clade2_Ec107like**	299,6	high	Bacillota	Clostridia	Lachnospirales	Lachnospiraceae	—	uncultured Lachnospiraceae bacterium	SAMEA7202512	host-associated	7	Yes	3	AAA-ATPase_like;Trypsin_2;UTRA	—
411463.SAMN00627091.DS264270_16	**clade2_Ec107like**	298	high	Bacillota	Clostridia	Eubacteriales	Eubacteriaceae	Eubacterium	Eubacterium ventriosum	SAMN00627091	host-associated	11	Yes	4	DUF2201_N;GmrSD_N;FOXO-TAD	—
1658109.SAMEA3442603.LN852692_3	**clade2_Ec107like**	298	high	Bacillota	Erysipelotrichia	Erysipelotrichales	Erysipelotrichaceae	Candidatus Stoquefich	Candidatus Stoquefichus sp. SB1	SAMEA3442603	host-associated	11	Yes	4	DUF3784;PucR;VanY;RBFA	—
59620.SAMEA7847086.CAJLJW01000(	**clade2_Ec107like**	297,8	high	Bacillota	Clostridia	Eubacteriales	Clostridiaceae	Clostridium	uncultured Clostridium sp.	SAMEA7847086	host-associated	11	Yes	4	SbcD_C;Cupin_2;Peptidase_M20;A	—
610130.SAMEA7202544.CAJFOY0100(	**clade2_Ec107like**	297	high	Bacillota	Clostridia	Lachnospirales	Lachnospiraceae	Lacrimispora	Lacrimispora saccharolytica	SAMEA7202544	host-associated	11	Yes	4	Big_3;Cupin_2;PRD	—
224209.SAMEA8804963.CAJTLN0100(	**clade2_Ec107like**	296,7	high	Bacillota	Bacilli	—	—	—	uncultured Bacilli bacterium	SAMEA8804963	host-associated	11	Yes	4	HTH_5;Hydrolase;APH;DUF1653	—
297314.SAMEA8805827.CAJURT0100(	**clade2_Ec107like**	296,5	high	Bacillota	Clostridia	Lachnospirales	Lachnospiraceae	—	uncultured Lachnospiraceae bacterium	SAMEA8805827	host-associated	11	Yes	4	CAP;PfkB;BPD_transp_1	—
2044939.SAMEA5849627.CABJAP010(	**clade2_Ec107like**	296,4	high	Bacillota	Clostridia	—	—	—	Clostridia bacterium	SAMEA5849627	host-associated	11	Yes	4	Peptidase_S8;Acetyltransf_1;DUF1	—
224209.SAMEA8805129.CAJTRV0100(	**clade2_Ec107like**	296	high	Bacillota	Bacilli	—	—	—	uncultured Bacilli bacterium	SAMEA8805129	host-associated	9	Yes	4	Ribosomal_L44;Acetyltransf_1;PHP	—
172733.SAMEA7847762.CAJKVZ01000(	**clade2_Ec107like**	294,9	high	Bacillota	Clostridia	Eubacteriales	—	—	uncultured Eubacteriales bacterium	SAMEA7847762	host-associated	6	Yes	2	FMN_dh;Branch_AA_trans	—
2485925.SAMEA6150724.CACZGD010	**clade2_Ec107like**	294,9	high	Bacillota	Clostridia	Eubacteriales	Oscillospiraceae	—	Oscillospiraceae bacterium	SAMEA6150724	host-associated	8	Yes	4	HTH_28;ThiG	—
2485925.SAMN11294983.SVTP010000	**clade2_Ec107like**	293,9	high	Bacillota	Clostridia	Eubacteriales	Oscillospiraceae	—	Oscillospiraceae bacterium	SAMN11294983	host-associated	8	Yes	4	RHS_repeat;MerR_1;Glyco_hydro_	—
2485925.SAMN16345939.JAFVXA0100	**clade2_Ec107like**	293,8	high	Bacillota	Clostridia	Eubacteriales	Oscillospiraceae	—	Oscillospiraceae bacterium	SAMN16345939	host-associated	11	Yes	4	Pan_kinase;ECF_trnsprt;DUF7575;	—
172733.SAMEA8805310.CAJUBH0100(	**clade2_Ec107like**	293,5	high	Bacillota	Clostridia	Eubacteriales	—	—	uncultured Eubacteriales bacterium	SAMEA8805310	host-associated	11	Yes	4	Peptidase_Prp;Ribosomal_L27;HD_	—
2838555.SAMN15816711.DXDD010000	**clade2_Ec107like**	293,3	high	Bacillota	Clostridia	Lachnospirales	Lachnospiraceae	Eisenbergiella	Candidatus Eisenbergiella pullistercoris	SAMN15816711	host-associated	11	Yes	4	DUF3592;Cellulase;DUF2268;DAP	—
1898204.SAMEA8801320.CAJSZV010(	**clade2_Ec107like**	293,1	high	Bacillota	Clostridia	Eubacteriales	Clostridiaceae	—	Clostridiaceae bacterium	SAMEA8801320	host-associated	11	Yes	4	zf-IS66;Acyl_transf_3;SDF	—
2044939.SAMN16346710.JAFXAN010(	**clade2_Ec107like**	293	high	Bacillota	Clostridia	—	—	—	Clostridia bacterium	SAMN16346710	host-associated	10	Yes	4	DNA_ligase_aden;WHD_RNase_R	—
224209.SAMEA7202252.CAJFHJ01000	**clade2_Ec107like**	292,8	high	Bacillota	Bacilli	—	—	—	uncultured Bacilli bacterium	SAMEA7202252	host-associated	8	Yes	4	PIK3CG_ABD;DUF1292;CTP_synt	—
1750510.SAMEA7202319.CAJFVE010(	**clade2_Ec107like**	292,8	high	Bacillota	Clostridia	Lachnospirales	Lachnospiraceae	Eisenbergiella	uncultured Eisenbergiella sp.	SAMEA7202319	host-associated	11	Yes	4	DAPDH_C;Cellulase;MFS_1	—
2049044.SAMN16346208.JAFWHJ010(	**clade2_Ec107like**	292,7	high	Bacillota	Erysipelotrichia	Erysipelotrichales	Erysipelotrichaceae	—	Erysipelotrichaceae bacterium	SAMN16346208	host-associated	11	Yes	4	DUF4230;Hydrolase_3;Hydrolase;R	—
2485925.SAMN11294755.SVKV010000	**clade2_Ec107like**	292,5	high	Bacillota	Clostridia	Eubacteriales	Oscillospiraceae	—	Oscillospiraceae bacterium	SAMN11294755	host-associated	6	Yes	2	TatD_DNase;UDPGT	—
1849603.SAMEA5278299.CAAFVQ010	**clade2_Ec107like**	292,3	high	Pseudomonadota	Gammaproteobacteria	Enterobacterales	Enterobacteriaceae	—	Enterobacteriaceae bacterium	SAMEA5278299	host-associated	11	Yes	4	RNase_PH;Pribosyltran;SlmA-like_(	—
2485925.SAMN11294929.SVRN010000	**clade2_Ec107like**	292,3	high	Bacillota	Clostridia	Eubacteriales	Oscillospiraceae	—	Oscillospiraceae bacterium	SAMN11294929	host-associated	10	Yes	4	NADAR-DarT1;TetR_C_33;ABC_tr	—
2838556.SAMN15816624.DXHY010000	**clade2_Ec107like**	292,2	high	Bacillota	Clostridia	Lachnospirales	Lachnospiraceae	Eisenbergiella	Candidatus Eisenbergiella stercoraviur	SAMN15816624	host-associated	11	Yes	4	Target_ACGX;DUF3592;Cellulase;I	—
2763047.SAMN15808687.JACOPN010(	**clade2_Ec107like**	292,1	high	Bacillota	Clostridia	Eubacteriales	—	Flintibacter	Flintibacter faecis	SAMN15808687	host-associated	11	Yes	4	CobA_CobO_BtuR;PEP-utilizers_C	—
224209.SAMEA8805448.CAJUBJ01000	**clade2_Ec107like**	291,7	high	Bacillota	Bacilli	—	—	—	uncultured Bacilli bacterium	SAMEA8805448	host-associated	10	Yes	4	ADP_ribosyl_GH;HH_RND_rel;APH	—
297314.SAMEA4891325.UQVK010001:	**clade2_Ec107like**	291,5	high	Bacillota	Clostridia	Lachnospirales	Lachnospiraceae	—	uncultured Lachnospiraceae bacterium	SAMEA4891325	host-associated	7	Yes	3	DAPDH_C;DUF3592;MFS_1	—
297314.SAMEA8805098.CAJTPQ0100(	**clade2_Ec107like**	291,5	high	Bacillota	Clostridia	Lachnospirales	Lachnospiraceae	—	uncultured Lachnospiraceae bacterium	SAMEA8805098	host-associated	11	Yes	4	HTH_18;Glyco_hydro_2_C;DUF53	—
157472.SAMEA7846648.CAJJRY01000	**clade2_Ec107like**	291,5	high	Bacillota	Bacilli	Bacillales	—	—	uncultured Bacillales bacterium	SAMEA7846648	host-associated	7	Yes	3	Adenine_glyco;FapA;Epimerase	—
2720819.SAMEA7202238.CAJFGS010(	**clade2_Ec107like**	290,7	high	Bacillota	Clostridia	—	—	Candidatus Avimonas	Candidatus Avimonas merdigallinarum	SAMEA7202238	host-associated	11	Yes	4	PAD_porph;DUF7620;DUF2201_N	—
172733.SAMEA5850774.CABKLX01000	**clade2_Ec107like**	290,7	high	Bacillota	Clostridia	Eubacteriales	—	—	uncultured Eubacteriales bacterium	SAMEA5850774	host-associated	7	Yes	3	HTH_3;La;GFO_IDH_MocA	—
59620.SAMEA7202323.CAJFJI0100000	**clade2_Ec107like**	290,6	high	Bacillota	Clostridia	Eubacteriales	Clostridiaceae	Clostridium	uncultured Clostridium sp.	SAMEA7202323	host-associated	11	Yes	4	Radical_SAM;DUF4130;C_GCAxx(	—
1924109.SAMN18359857.JAGHID0100	**clade2_Ec107like**	290,5	high	Bacillota	Clostridia	Lachnospirales	Lachnospiraceae	Eisenbergiella	Eisenbergiella sp.	SAMN18359857	host-associated	5	Yes	3	PfkB;AbiEii;DUF6088	—
59620.SAMEA7847304.CAJJPT010000	**clade2_Ec107like**	290,3	high	Bacillota	Clostridia	Eubacteriales	Clostridiaceae	Clostridium	uncultured Clostridium sp.	SAMEA7847304	host-associated	7	Yes	3	SMC_N;Fzo_mitofusin	—
297314.SAMEA8805487.CAJUHR0100(	**clade2_Ec107like**	290,2	high	Bacillota	Clostridia	Lachnospirales	Lachnospiraceae	—	uncultured Lachnospiraceae bacterium	SAMEA8805487	host-associated	11	Yes	4	BPD_transp_1;Peptidase_M20;Hep	—
1879010.SAMN11294487.SVAM010000	**clade2_Ec107like**	290,1	high	Bacillota	—	—	—	—	Bacillota bacterium	SAMN11294487	host-associated	11	Yes	4	DUF951;ABG_transport;YchF-GTP	—
2840815.SAMN15817011.DVGI010000	**clade2_Ec107like**	290	high	Bacillota	Clostridia	—	—	Candidatus Fimenecus	Candidatus Fimenecus excrementaviu	SAMN15817011	—	7	Yes	0	—	—
2005360.SAMN18359906.JAGHKA010(	**clade2_Ec107like**	289,8	high	Bacillota	Clostridia	Lachnospirales	Lachnospiraceae	Faecalicatena	Faecalicatena sp.	SAMN18359906	host-associated	11	Yes	4	DegV;DHDPS;HTH_11;PP-binding	—
2044939.SAMN16349910.JAGBSO010(	**clade2_Ec107like**	289,8	high	Bacillota	Clostridia	—	—	—	Clostridia bacterium	SAMN16349910	host-associated	11	Yes	4	Aldolase_II;SCP2;LbH_EIF2B;Pept	—
165185.SAMEA4891232.UQRM010000	**clade2_Ec107like**	289,3	high	Bacillota	Clostridia	Eubacteriales	Eubacteriaceae	Eubacterium	uncultured Eubacterium sp.	SAMEA4891232	host-associated	8	Yes	3	HTH_3;PIN_8;Thioredoxin_3	—
1262908.PRJEB1041.HF993109_93	**clade2_Ec107like**	289,3	high	Mycoplasmatota	Mollicutes	Mycoplasmatales	Mycoplasmataceae	Mycoplasma	Mycoplasma sp. CAG:956	PRJEB1041	host-associated	11	Yes	4	LRR_At5g56370;Transglut_core;HT	—
2485925.SAMN11294845.SVOH010000	**clade2_Ec107like**	288,6	high	Bacillota	Clostridia	Eubacteriales	Oscillospiraceae	—	Oscillospiraceae bacterium	SAMN11294845	host-associated	11	Yes	4	His_Phos_1;RuvX;Acetyltransf_1;G	—
658143.SAMEA4891343.UQVP010000	**clade2_Ec107like**	288,5	high	Mycoplasmatota	—	—	—	—	uncultured Mycoplasmatota bacterium	SAMEA4891343	host-associated	8	Yes	4	DUF4006;Flg_new;HD_3	—
1903720.SAMEA5278229.CAAGCO01(	**clade2_Ec107like**	288,4	high	Bacillota	Bacilli	—	—	—	Bacilli bacterium	SAMEA5278229	host-associated	8	Yes	4	B3_4;H-kinase_dim;NlpE	—
707003.SAMEA7846686.CAJJLK01000	**clade2_Ec107like**	287,9	high	Bacillota	Clostridia	Eubacteriales	Oscillospiraceae	—	uncultured Oscillospiraceae bacterium	SAMEA7846686	host-associated	6	Yes	2	GGDEF	—
2320105.SAMN10024802.RAYY010000	**clade2_Ec107like**	287,9	high	—	—	—	—	—	bacterium D16-56	SAMN10024802	host-associated	11	Yes	4	AraC_binding;DUF2809;Peptidase_	—
1903720.SAMEA5278509.CAAEXI0100	**clade2_Ec107like**	287,8	high	Bacillota	Bacilli	—	—	—	Bacilli bacterium	SAMEA5278509	host-associated	11	Yes	4	Mur_ligase_M;DNA_binding_1;SLT	—
1965578.SAMN06473650.NFKG01000(	**clade2_Ec107like**	287,4	high	Bacillota	Clostridia	Eubacteriales	Oscillospiraceae	Pseudoflavonifractor	Pseudoflavonifractor sp. An187	SAMN06473650	host-associated	11	Yes	4	PAD_porph;Pur_DNA_glyco;Sel1	—
1898207.SAMN19224892.JAHZBC010(	**clade2_Ec107like**	286,6	high	Bacillota	Clostridia	Eubacteriales	—	—	Clostridiales bacterium	SAMN19224892	host-associated	11	Yes	4	GntP_permease;PAS_6;2-Hacid_dh	—
297314.SAMEA8805573.CAJUGD0100	**clade2_Ec107like**	286,6	high	Bacillota	Clostridia	Lachnospirales	Lachnospiraceae	—	uncultured Lachnospiraceae bacterium	SAMEA8805573	host-associated	11	Yes	4	Cas12f1-like_TNB;DUF2809;Peptic	—
1751881.SAMEA8805622.CAJUJV010(	**clade2_Ec107like**	286,5	high	Bacillota	Clostridia	Lachnospirales	Lachnospiraceae	Hungatella	uncultured Hungatella sp.	SAMEA8805622	host-associated	11	Yes	4	Flagellin_C;DUF6510;CyanoTRADI	—
41978.SAMN16345046.JAFUKO010000	**clade2_Ec107like**	286	high	Bacillota	Clostridia	Eubacteriales	Oscillospiraceae	Ruminococcus	Ruminococcus sp.	SAMN16345046	host-associated	11	Yes	4	Dockerin_1;PepSY;FIK;Y1_Tnp	—
28121.SAMN16349382.JAGAYD010000	**clade2_Ec107like**	286	high	Bacillota	Clostridia	Lachnospirales	Lachnospiraceae	Butyrivibrio	Butyrivibrio sp.	SAMN16349382	host-associated	11	Yes	4	GtfA_EBD;MS_TIM-barrel;PPR_3	—
157472.SAMEA7847028.CAJLIR01000(	**clade2_Ec107like**	285,9	high	Bacillota	Bacilli	Bacillales	—	—	uncultured Bacillales bacterium	SAMEA7847028	host-associated	11	Yes	4	Sporozoite_P67;ETRAMP;SesA	—
2840820.SAMN15817229.DVML010000	**clade2_Ec107like**	285,6	high	Mycoplasmatota	—	—	—	Candidatus Fimihabitar	Candidatus Fimihabitans intestinipullor	SAMN15817229	host-associated	11	Yes	4	SmpB;RNB;NDK	—
658143.SAMEA4891688.URIY0100001(	**clade2_Ec107like**	285,6	high	Mycoplasmatota	—	—	—	—	uncultured Mycoplasmatota bacterium	SAMEA4891688	host-associated	6	Yes	2	LytR_cpsA_psr;DDE_Tnp_ISL3	—
59620.SAMEA7202342.CAJFSI010000(	**clade2_Ec107like**	285,4	high	Bacillota	Clostridia	Eubacteriales	Clostridiaceae	Clostridium	uncultured Clostridium sp.	SAMEA7202342	host-associated	11	Yes	4	FA_synthesis;C_GCAxxG_C_C;Lys	—
172733.SAMEA4891262.UQSP0100000(	**clade2_Ec107like**	285,3	high	Bacillota	Clostridia	Eubacteriales	—	—	uncultured Eubacteriales bacterium	SAMEA4891262	host-associated	11	Yes	4	O-antigen_lig;HTH_19;RNA_pol_I_	—

2044939.SAMN16342067.JAFQCR010(	**clade2_Ec107like**	285	high	Bacillota	Clostridia	—	—	—	Clostridia bacterium	SAMN16342067	host-associated	11	Yes	4	TPP_enzyme_C;POR;GtaA_6_Hair	—
331630.SAMEA6149063.CACWUS010(	**clade2_Ec107like**	284,8	high	Bacillota	Erysipelotrichia	Erysipelotrichales	Erysipelotrichaceae	—	uncultured Erysipelotrichaceae bacteri	SAMEA6149063	host-associated	11	Yes	4	PedC-like;KTI12;Peptidase_M19	—
2840670.SAMN15817049.DVMT01000(	**clade2_Ec107like**	284,7	high	Bacillota	Bacilli	—	—	Candidatus Aphodocol	Candidatus Aphodocola excrementigal	SAMN15817049	host-associated	11	Yes	4	SLT;HMGL-like;Cyclophil_like2;Pep	—
297314.SAMEA8805848.CAJUSJ01000	**clade2_Ec107like**	284,4	high	Bacillota	Clostridia	Lachnospirales	Lachnospiraceae	—	uncultured Lachnospiraceae bacterium	SAMEA8805848	host-associated	11	Yes	4	YoeB_toxin;Hydrolase_3;HemY_N	—
2044939.SAMEA5278674.CAAFVV010(	**clade2_Ec107like**	284,3	high	Bacillota	Clostridia	—	—	—	Clostridia bacterium	SAMEA5278674	host-associated	9	Yes	4	PMI_typeI_cat;SpoU_methylase;Na	—
297314.SAMEA8805495.CAJUDH0100(	**clade2_Ec107like**	283,7	high	Bacillota	Clostridia	Lachnospirales	Lachnospiraceae	—	uncultured Lachnospiraceae bacterium	SAMEA8805495	host-associated	11	Yes	4	PfkB;ROK;MobB;MoeA_N	—
1263032.PRJEB1017.HF990861_60	**clade2_Ec107like**	283,3	high	Bacillota	—	—	—	—	Firmicutes bacterium CAG:822	PRJEB1017	host-associated	11	Yes	4	Aldo_ket_red;HSP20;tRNA-synt_1c	—
1265.SAMEA6148936.CACWPR01000(	**clade2_Ec107like**	283,1	high	Bacillota	Clostridia	Eubacteriales	Oscillospiraceae	Ruminococcus	Ruminococcus flavefaciens	SAMEA6148936	host-associated	7	Yes	3	SdpI;DinB_2;PGK	—
297314.SAMEA7202416.CAJFLQ01000	**clade2_Ec107like**	282,9	high	Bacillota	Clostridia	Lachnospirales	Lachnospiraceae	—	uncultured Lachnospiraceae bacterium	SAMEA7202416	host-associated	11	Yes	4	Fer4_22;HTH_Crp_2;Form_Nir_trai	—
59620.SAMEA7846780.CAJJLQ010000	**clade2_Ec107like**	282,7	high	Bacillota	Clostridia	Eubacteriales	Clostridiaceae	Clostridium	uncultured Clostridium sp.	SAMEA7846780	host-associated	11	Yes	4	Methyltransf_4;tRNA-synt_1b;DUF!	—
2485925.SAMN11294846.SVOI0100000	**clade2_Ec107like**	282,4	high	Bacillota	Clostridia	Eubacteriales	Oscillospiraceae	—	Oscillospiraceae bacterium	SAMN11294846	host-associated	11	Yes	4	Helicase_dom;DUF2201_N;AAA_5	—
2485925.SAMEA7847870.CAJKFU010(	**clade2_Ec107like**	282,1	high	Bacillota	Clostridia	Eubacteriales	Oscillospiraceae	—	Oscillospiraceae bacterium	SAMEA7847870	host-associated	11	Yes	4	Helicase_C_2;ClpB_D2-small;ABC_	—
41978.SAMN16340732.JAFOED010000	**clade2_Ec107like**	281,8	high	Bacillota	Clostridia	Eubacteriales	Oscillospiraceae	Ruminococcus	Ruminococcus sp.	SAMN16340732	host-associated	8	Yes	4	DUF8255;PepSY;Acyl_transf_3	—
297314.SAMEA7202269.CAJFIE010000	**clade2_Ec107like**	281,7	high	Bacillota	Clostridia	Lachnospirales	Lachnospiraceae	—	uncultured Lachnospiraceae bacterium	SAMEA7202269	host-associated	11	Yes	4	MarR_2;Form_Nir_trans;HTH_Crp_	—
658143.SAMEA4891289.UQTP0100000	**clade2_Ec107like**	281,4	high	Mycoplasmatota	—	—	—	—	uncultured Mycoplasmatota bacterium	SAMEA4891289	host-associated	11	Yes	4	CofD;Peptidase_S55;Epimerase	—
2485925.SAMN16927241.JAEDFW010	**clade2_Ec107like**	280,9	high	Bacillota	Clostridia	Eubacteriales	Oscillospiraceae	—	Oscillospiraceae bacterium	SAMN16927241	host-associated	3	Yes	0	—	—
2721134.SAMEA7202493.CAJFNQ010(	**clade2_Ec107like**	280,9	high	Bacillota	Clostridia	—	—	Candidatus Metarumin	Candidatus Metaruminococcus galliste	SAMEA7202493	host-associated	11	Yes	4	AcrF2;Flg_new_2;Fe-ADH;HrcA	—
2060878.SAMN16344967.JAFUHN010(	**clade2_Ec107like**	280,8	high	Bacillota	Erysipelotrichia	Erysipelotrichales	Erysipelotrichaceae	Solobacterium	Solobacterium sp.	SAMN16344967	host-associated	11	Yes	4	MerR_1;Imp-YgjV;Gate;Lactamase_	—
1265.SAMN04487832.FPJT01000001_	**clade2_Ec107like**	280,3	high	Bacillota	Clostridia	Eubacteriales	Oscillospiraceae	Ruminococcus	Ruminococcus flavefaciens	SAMN04487832	anthropogenic	11	Yes	4	HipA_C;Resolvase;GATase_6;Form	—
1898207.SAMN10183766.SFHJ010000	**clade2_Ec107like**	280,1	high	Bacillota	Clostridia	Eubacteriales	—	—	Clostridiales bacterium	SAMN10183766	host-associated	11	Yes	4	DUF4015;DUF554;Iso_dh;Aconitas	—
331630.SAMEA6150187.CACYLN0100(	**clade2_Ec107like**	280,1	high	Bacillota	Erysipelotrichia	Erysipelotrichales	Erysipelotrichaceae	—	uncultured Erysipelotrichaceae bacteri	SAMEA6150187	host-associated	11	Yes	4	Glyco_hydro_1;MFS_1_like;TelA;D.	—
297314.SAMEA8805230.CAJTTU01000	**clade2_Ec107like**	279,9	high	Bacillota	Clostridia	Lachnospirales	Lachnospiraceae	—	uncultured Lachnospiraceae bacterium	SAMEA8805230	host-associated	11	Yes	4	AAA_2;ClpS;Wzy_C	—
1751881.SAMEA8805086.CAJTOT010(	**clade2_Ec107like**	279,8	high	Bacillota	Clostridia	Lachnospirales	Lachnospiraceae	Hungatella	uncultured Hungatella sp.	SAMEA8805086	host-associated	11	Yes	4	LysR_substrate;Aldo_ket_red;MreB	—
658143.SAMEA4892001.URUT0100002	**clade2_Ec107like**	279,7	high	Mycoplasmatota	—	—	—	—	uncultured Mycoplasmatota bacterium	SAMEA4892001	host-associated	11	Yes	4	Peptidase_C39_2;dUTPase_2;3HC	—
1898203.SAMN17800722.JAGZCX010(	**clade2_Ec107like**	279,4	high	Bacillota	Clostridia	Lachnospirales	Lachnospiraceae	—	Lachnospiraceae bacterium	SAMN17800722	host-associated	11	Yes	4	Mg_chelatase;C_GCAxxG_C_C;DL	—
1898204.SAMN09901061.QXWR01000	**clade2_Ec107like**	279,3	high	Bacillota	Clostridia	Eubacteriales	Clostridiaceae	—	Clostridiaceae bacterium	SAMN09901061	host-associated	11	Yes	4	Lactamase_B;Radical_SAM;HATPa	—
707003.SAMEA8805751.CAJUOA0100(	**clade2_Ec107like**	278,6	high	Bacillota	Clostridia	Eubacteriales	Oscillospiraceae	—	uncultured Oscillospiraceae bacterium	SAMEA8805751	host-associated	6	Yes	2	PAPS_reduct;DUF6110	—
2044939.SAMN16344038.JAFSXU010(	**clade2_Ec107like**	277,9	high	Bacillota	Clostridia	—	—	—	Clostridia bacterium	SAMN16344038	host-associated	11	Yes	4	Y1_Tnp;PrsW-protease;GATase;His	—
172733.SAMEA7846782.CAJLHB01000	**clade2_Ec107like**	277,8	high	Bacillota	Clostridia	Eubacteriales	—	—	uncultured Eubacteriales bacterium	SAMEA7846782	host-associated	7	Yes	3	IU_nuc_hydro;HTH_18;Transpeptid	—
707003.SAMEA5278737.CAAGBT0100	**clade2_Ec107like**	277,7	high	Bacillota	Clostridia	Eubacteriales	Oscillospiraceae	—	uncultured Oscillospiraceae bacterium	SAMEA5278737	host-associated	9	Yes	4	PDDEXK_4;DUF2203;GTP_EFTU;	—
1898207.SAMN19225273.DXYE010001	**clade2_Ec107like**	277,3	high	Bacillota	Clostridia	Eubacteriales	—	—	Clostridiales bacterium	SAMN19225273	—	4	Yes	0	—	—
707003.SAMEA5278732.CAAFCU0100	**clade2_Ec107like**	276,9	high	Bacillota	Clostridia	Eubacteriales	Oscillospiraceae	—	uncultured Oscillospiraceae bacterium	SAMEA5278732	—	11	Yes	0	—	—
1965629.SAMN06473716.NFIQ010000	**clade2_Ec107like**	276,7	high	Bacillota	Clostridia	Eubacteriales	Oscillospiraceae	Flavonifractor	Flavonifractor sp. An306	SAMN06473716	host-associated	11	Yes	4	TP_0679;zf-like;DUF4367	—
165186.SAMEA4891392.UQXS010001	**clade2_Ec107like**	276,3	high	Bacillota	Clostridia	Eubacteriales	Oscillospiraceae	Ruminococcus	uncultured Ruminococcus sp.	SAMEA4891392	host-associated	7	Yes	4	Form-deh_trans;GATase_6;Phage_	—
224209.SAMEA8805494.CAJUCD0100(	**clade2_Ec107like**	276,1	high	Bacillota	Bacilli	—	—	—	uncultured Bacilli bacterium	SAMEA8805494	host-associated	11	Yes	4	FXR_C3;Acetyltransf_7;YchF-GTPa	—
172733.SAMEA4891435.UQZJ0100003	**clade2_Ec107like**	275,4	high	Bacillota	Clostridia	Eubacteriales	—	—	uncultured Eubacteriales bacterium	SAMEA4891435	host-associated	10	Yes	4	PAD_porph;DSPc;DUF1722	—
1193532.SAMEA7848332.CAJKYR010(	**clade2_Ec107like**	275,2	high	Bacillota	Clostridia	Eubacteriales	Butyricicoccaceae	Butyricicoccus	uncultured Butyricicoccus sp.	SAMEA7848332	host-associated	11	Yes	4	HTH_3;Pyrophosphatase;MFS_2;B	—
2044939.SAMN16345817.JAFVSI01000	**clade2_Ec107like**	275,2	high	Bacillota	Clostridia	—	—	—	Clostridia bacterium	SAMN16345817	host-associated	7	Yes	3	TMEM164;SASA;HTH_18	—
707003.SAMEA7847196.CAJLTB01000	**clade2_Ec107like**	275,1	high	Bacillota	Clostridia	Eubacteriales	Oscillospiraceae	—	uncultured Oscillospiraceae bacterium	SAMEA7847196	—	11	Yes	0	—	—
1506.SAMN17801316.JAGZZT0100000	**clade2_Ec107like**	275,1	high	Bacillota	Clostridia	Eubacteriales	Clostridiaceae	Clostridium	Clostridium sp.	SAMN17801316	host-associated	11	Yes	4	SbcD_C;Cupin_2;Peptidase_M20;A	—
41978.SAMN16346810.JAFXEJ010000	**clade2_Ec107like**	274,7	high	Bacillota	Clostridia	Eubacteriales	Oscillospiraceae	Ruminococcus	Ruminococcus sp.	SAMN16346810	host-associated	7	Yes	3	AGA-YXIM_GBD	—
165186.SAMEA8805374.CAJTWZ0100	**clade2_Ec107like**	274,6	high	Bacillota	Clostridia	Eubacteriales	Oscillospiraceae	Ruminococcus	uncultured Ruminococcus sp.	SAMEA8805374	host-associated	11	Yes	4	NikA-like;Relaxase;SH3_3;DUF417	—
297314.SAMEA6152050.CADBFE0100(	**clade2_Ec107like**	274,5	high	Bacillota	Clostridia	Lachnospirales	Lachnospiraceae	—	uncultured Lachnospiraceae bacterium	SAMEA6152050	host-associated	11	Yes	4	AAA_5;DUF2201_N;Dpy19;Zn_Rib	—
1898207.SAMN10183782.SFIY0100002	**clade2_Ec107like**	274,4	high	Bacillota	Clostridia	Eubacteriales	—	—	Clostridiales bacterium	SAMN10183782	—	11	Yes	0	—	—
1410672.SAMN02744011.JHXI0100001	**clade2_Ec107like**	274,2	high	Bacillota	Clostridia	Eubacteriales	Oscillospiraceae	Ruminococcus	Ruminococcus flavefaciens	SAMN02744011	host-associated	11	Yes	4	Sigma70_r4_2;Hydrolase_4;Pro_CA	—
707003.SAMEA5851660.CABLZZ01000	**clade2_Ec107like**	274,2	high	Bacillota	Clostridia	Eubacteriales	Oscillospiraceae	—	uncultured Oscillospiraceae bacterium	SAMEA5851660	—	11	Yes	0	—	—
297314.SAMEA8805828.CAJUPT0100(	**clade2_Ec107like**	273,9	high	Bacillota	Clostridia	Lachnospirales	Lachnospiraceae	—	uncultured Lachnospiraceae bacterium	SAMEA8805828	host-associated	11	Yes	4	AAA_2;ClpS;VanZ;Wzy_C	—
876091.SAMEA8805491.CAJUDV0100(	**clade2_Ec107like**	273,8	high	Bacillota	Clostridia	Eubacteriales	Oscillospiraceae	Oscillibacter	uncultured Oscillibacter sp.	SAMEA8805491	host-associated	10	Yes	4	PCRF;SLH;Sigma70_r2;zf-HC2	—
1262910.PRJEB727.FR890955_127	**clade2_Ec107like**	273,8	high	Bacillota	Clostridia	Eubacteriales	Oscillospiraceae	Oscillibacter	Oscillibacter sp. CAG:155	PRJEB727	host-associated	8	Yes	4	Rhomboid;S-AdoMet_synt_C;CSD;:	—
1265.SAMEA6150373.CACYSQ010000	**clade2_Ec107like**	273,6	high	Bacillota	Clostridia	Eubacteriales	Oscillospiraceae	Ruminococcus	Ruminococcus flavefaciens	SAMEA6150373	host-associated	11	Yes	4	Spore_permease;GerA;Phage_integ	—
142586.SAMN18359832.JAGHHE0100(	**clade2_Ec107like**	273,3	high	Bacillota	Clostridia	Eubacteriales	Eubacteriaceae	Eubacterium	Eubacterium sp.	SAMN18359832	host-associated	7	Yes	3	Acetyltransf_7;AAA_30;Methyltrans	—
1689270.SAMEA5138309.LR130816_6	**clade2_Ec107like**	272,8	high	Bacillota	Clostridia	Eubacteriales	—	Intestinimonas	Intestinimonas timonensis	SAMEA5138309	host-associated	11	Yes	4	Lyx_isomer;PfkB;MarR;ABC_memb	—
876091.SAMEA4891362.UWSF010000	**clade2_Ec107like**	272,7	high	Bacillota	Clostridia	Eubacteriales	Oscillospiraceae	Oscillibacter	uncultured Oscillibacter sp.	SAMEA4891362	—	11	Yes	0	—	—
59620.SAMEA7847350.CAJLVU010000	**clade2_Ec107like**	272,7	high	Bacillota	Clostridia	Eubacteriales	Clostridiaceae	Clostridium	uncultured Clostridium sp.	SAMEA7847350	host-associated	11	Yes	4	AMP-binding;3HCDH_N;FerA;Pepti	—
707003.SAMEA4890827.UQBX010000(	**clade2_Ec107like**	272,5	high	Bacillota	Clostridia	Eubacteriales	Oscillospiraceae	—	uncultured Oscillospiraceae bacterium	SAMEA4890827	host-associated	11	Yes	4	Cupin_2;Pectate_lyase_5;VCA004(	—
172733.SAMEA7847389.CAJKPX01000	**clade2_Ec107like**	272,4	high	Bacillota	Clostridia	Eubacteriales	—	—	uncultured Eubacteriales bacterium	SAMEA7847389	—	11	Yes	4	BNR_2;HTH_18;Cellulase	—
876091.SAMEA8805735.CAJULS01000	**clade2_Ec107like**	271,1	high	Bacillota	Clostridia	Eubacteriales	Oscillospiraceae	Oscillibacter	uncultured Oscillibacter sp.	SAMEA8805735	host-associated	8	Yes	4	RBR;SLH;Sigma70_r2;Ribosomal_!	—
2049044.SAMN11294514.SVBN010000	**clade2_Ec107like**	270,7	high	Bacillota	Erysipelotrichia	Erysipelotrichales	Erysipelotrichaceae	—	Erysipelotrichaceae bacterium	SAMN11294514	host-associated	10	Yes	4	Peptidase_C69;FMN_red;FMN_dh	—
707003.SAMEA7202338.CAJFJV01000	**clade2_Ec107like**	270	high	Bacillota	Clostridia	Eubacteriales	Oscillospiraceae	—	uncultured Oscillospiraceae bacterium	SAMEA7202338	host-associated	11	Yes	4	Asparaginase;BetaGal_ABD_1;BcrA	—
2044939.SAMN16342657.JAFQVO010(	**clade2_Ec107like**	269,1	high	Bacillota	Clostridia	—	—	—	Clostridia bacterium	SAMN16342657	host-associated	5	Yes	3	Pribosyltran;Transket_pyr;Transketc	—
876091.SAMEA8805797.CAJUQM0100	**clade2_Ec107like**	268,2	high	Bacillota	Clostridia	Eubacteriales	Oscillospiraceae	Oscillibacter	uncultured Oscillibacter sp.	SAMEA8805797	host-associated	10	Yes	4	PTPS_related;GDPD;SLH;PCRF	—
411475.SAMN00627089.JH417816_7	**clade2_Ec107like**	268	high	Bacillota	Clostridia	Eubacteriales	Oscillospiraceae	Flavonifractor	Flavonifractor plautii	SAMN00627089	host-associated	11	Yes	4	AIM24;AMP-binding;LuxC	—
2049044.SAMN16343168.JAFRQF010(	**clade2_Ec107like**	267,9	high	Bacillota	Erysipelotrichia	Erysipelotrichales	Erysipelotrichaceae	—	Erysipelotrichaceae bacterium	SAMN16343168	host-associated	10	Yes	4	AAA_14;DSPc;DUF4371;PerC	—
876091.SAMEA5279433.CAAGJQ0100(	**clade2_Ec107like**	267,1	high	Bacillota	Clostridia	Eubacteriales	Oscillospiraceae	Oscillibacter	uncultured Oscillibacter sp.	SAMEA5279433	host-associated	7	Yes	4	MatE;HerA_C;SLH;PCRF	—
2584680.SAMN11946324.WNAN01000	**clade2_Ec107like**	267,1	high	Bacillota	Clostridia	Eubacteriales	Oscillospiraceae	Pseudoflavonifractor	Pseudoflavonifractor sp. BIOML-A18	SAMN11946324	host-associated	11	Yes	4	Molybdopterin;FdhD-NarQ;CutC;Tra	—
2763673.SAMN15805297.JACRTB010(	**clade2_Ec107like**	266,7	high	Bacillota	Clostridia	Eubacteriales	Oscillospiraceae	Yanshouia	Yanshouia hominis	SAMN15805297	host-associated	11	Yes	4	ECF_trnsprt;ATP_bind_3;RNase_Y	—
1265.SAMEA6148735.CACWHY010000	**clade2_Ec107like**	263,8	high	Bacillota	Clostridia	Eubacteriales	Oscillospiraceae	Ruminococcus	Ruminococcus flavefaciens	SAMEA6148735	anthropogenic	11	Yes	4	DUF6198;DUF4832;HATPase_c;R	—
41978.SAMN16344534.JAFTQW01000	**clade2_Ec107like**	262,6	high	Bacillota	Clostridia	Eubacteriales	Oscillospiraceae	Ruminococcus	Ruminococcus sp.	SAMN16344534	host-associated	11	Yes	4	GATase_6;PlyB_C;Response_reg;I	—
1898207.SAMN19225266.DYCV010000	**clade2_Ec107like**	262,1	high	Bacillota	Clostridia	Eubacteriales	—	—	Clostridiales bacterium	SAMN19225266	host-associated	11	Yes	4	PDDEXK_4;Metallophos;AAA_lid_6	—
2049044.SAMN16343007.JAFRKA010(	**clade2_Ec107like**	261,7	high	Bacillota	Erysipelotrichia	Erysipelotrichales	Erysipelotrichaceae	—	Erysipelotrichaceae bacterium	SAMN16343007	host-associated	11	Yes	4	SepF;DSPc;DUF4379;MazG-like	—
165186.SAMEA6150165.CACYKN0100	**clade2_Ec107like**	258,4	high	Bacillota	Clostridia	Eubacteriales	Oscillospiraceae	Ruminococcus	uncultured Ruminococcus sp.	SAMEA6150165	host-associated	11	Yes	4	DUF6311;IU_nuc_hydro;DUF308	—
2838570.SAMN15816904.DXDN010000	**clade2_Ec107like**	253,1	high	Bacillota	Clostridia	Eubacteriales	Eubacteriaceae	Eubacterium	Candidatus Eubacterium faecigallinaru	SAMN15816904	host-associated	11	Yes	4	EamA;Zn_ribbon_NrdR;PsbP_2;Sig	—
165186.SAMEA6151767.CADAUE0100	**clade2_Ec107like**	250,3	high	Bacillota	Clostridia	Eubacteriales	Oscillospiraceae	Ruminococcus	uncultured Ruminococcus sp.	SAMEA6151767	host-associated	11	Yes	4	Nmad3;Nmad2;WYL;HI_0787_N	—
41978.SAMN16342822.JAFRBX010000	**clade2_Ec107like**	248	high	Bacillota	Clostridia	Eubacteriales	Oscillospiraceae	Ruminococcus	Ruminococcus sp.	SAMN16342822	host-associated	11	Yes	4	Virul_fac_BrkB;PDDEXK_4;WYL;P	—
165186.SAMEA6152540.CADBYC0100	**clade2_Ec107like**	248	high	Bacillota	Clostridia	Eubacteriales	Oscillospiraceae	Ruminococcus	uncultured Ruminococcus sp.	SAMEA6152540	host-associated	11	Yes	4	WYL;HI_0787_N	—
165186.SAMEA6149501.CACXLM0100	**clade2_Ec107like**	242,6	high	Bacillota	Clostridia	Eubacteriales	Oscillospiraceae	Ruminococcus	uncultured Ruminococcus sp.	SAMEA6149501	host-associated	7	Yes	3	Zn_dep_PLPC;RIFIN;Sigma70_r3	—
41978.SAMN16342855.JAFRDE010000	**clade2_Ec107like**	242,5	high	Bacillota	Clostridia	Eubacteriales	Oscillospiraceae	Ruminococcus	Ruminococcus sp.	SAMN16342855	host-associated	11	Yes	4	Peptidase_M15;S-AdoMet_lyase;Fe	—
1689265.SAMEA7202227.CAJFGQ010(	**clade2_Ec107like**	241,1	high	Bacillota	Clostridia	Eubacteriales	—	Intestinimonas	uncultured Intestinimonas sp.	SAMEA7202227	host-associated	11	Yes	4	Peptidase_M56;Penicillinase_R;bPI	—
1002368.SAMN02299454.JH417862_4	**clade2_Ec107like**	224,9	low	Pseudomonadota	Gammaproteobacteria	Enterobacterales	Enterobacteriaceae	Yokenella	Yokenella regensburgei	SAMN02299454	host-associated	10	Yes	4	FGGY_C;AraC_binding;VTT_dom	—

2485925.SAMEA6150216.CACYMP010	**clade2_Ec107like**	210,2	low	Bacillota	Clostridia	Eubacteriales	Oscillospiraceae	—	Oscillospiraceae bacterium	SAMEA6150216	host-associated	10	Yes	4	OGG_N;Abhydrolase_3;Acetyltrans	—
2026780.SAMN10222782.RFON01000(	**clade2_Ec107like**	192	low	Planctomycetota	—	—	—	—	Planctomycetota bacterium	SAMN10222782	aquatic	4	Yes	3	N6_N4_Mtase;TauE	—
296830.SAMEA6951688.CAIOSQ0100(	**clade2_Ec107like**	186,1	low	Pseudomonadota	Alphaproteobacteria	Rhodospirillales	—	—	uncultured Rhodospirillales bacterium	SAMEA6951688	aquatic	5	Yes	2	AAA_5	—
945550.SAMN02952919.AEVT0100002	**typeI-A**	341,6	high	Pseudomonadota	Gammaproteobacteria	Vibrionales	Vibrionaceae	Vibrio	Vibrio sinaloensis	SAMN02952919	aquatic	11	Yes	4	SMC_N;Ribosomal_L25p;Glycos_tr	—
1806667.SAMEA4029000.FLRA010000	**typeI-A**	338	high	Pseudomonadota	Gammaproteobacteria	Oceanospirillales	Oceanospirillaceae	Marinomonas	Marinomonas gallaica	SAMEA4029000	aquatic	11	Yes	4	SMC_N;TrbL;AF_1314_C	—
349965.SAMN00005354.AALF0200000	**typeI-A**	333,5	high	Pseudomonadota	Gammaproteobacteria	Enterobacterales	Yersiniaceae	Yersinia	Yersinia intermedia	SAMN00005354	anthropogenic	11	Yes	4	SF0329;SMC_N;Wzy_C;FaeA	—
1122212.SAMN02440684.AULO010000	**typeI-A**	330,9	high	Pseudomonadota	Gammaproteobacteria	Oceanospirillales	Oceanospirillaceae	Marinospirillum	Marinospirillum minutulum	SAMN02440684	anthropogenic	11	Yes	4	MmeI_Mtase;MmeI_N;SMC_N	—
1196094.SAMN03081540.CP007446_1	**typeI-A**	328,7	high	Pseudomonadota	Betaproteobacteria	Neisseriales	Neisseriaceae	Snodgrassella	Snodgrassella alvi	SAMN03081540	host-associated	11	Yes	4	SMC_N;FTHFS;TAL_FSA	—
181674.SAMEA6943824.CAIOMN0100(	**typeI-A**	325,7	high	Pseudomonadota	Gammaproteobacteria	Methylococcales	Methylococcaceae	—	uncultured Methylococcaceae bacteriu	SAMEA6943824	—	4	Yes	0	—	—
181674.SAMEA6955042.CAIMEB0100(	**typeI-A**	324,5	high	Pseudomonadota	Gammaproteobacteria	Methylococcales	Methylococcaceae	—	uncultured Methylococcaceae bacteriu	SAMEA6955042	aquatic	3	Yes	2	SMC_N;HNH_5	—
181674.SAMEA6949539.CAIPDT01000	**typeI-A**	320,8	high	Pseudomonadota	Gammaproteobacteria	Methylococcales	Methylococcaceae	—	uncultured Methylococcaceae bacteriu	SAMEA6949539	temperature	11	Yes	4	RepD-like_N;SMC_N;ESAG1;GmrS	—
154336.SAMEA9694770.CAJXQM0100	**typeI-A**	318,8	high	Pseudomonadota	Gammaproteobacteria	Alteromonadales	Colwelliaceae	Colwellia	uncultured Colwellia sp.	SAMEA9694770	temperature	7	Yes	3	Phage_GPA;HTH_29	—
326537.SAMN06626649.NBOE0100000(	**typeI-A**	318,8	high	Pseudomonadota	Gammaproteobacteria	Alteromonadales	Colwelliaceae	Colwellia	Colwellia polaris	SAMN06626649	—	11	Yes	0	—	—
2136183.SAMN08772518.PYIX0200000(	**typeI-A**	318	high	Pseudomonadota	Gammaproteobacteria	Moraxellales	Moraxellaceae	Acinetobacter	Acinetobacter sichuanensis	SAMN08772518	host-associated	11	Yes	4	RepD-like_N;SMC_N;NAD_kinase_	—
243277.SAMN02603969.AE003852_22	**typeI-A**	316,8	high	Pseudomonadota	Gammaproteobacteria	Vibrionales	Vibrionaceae	Vibrio	Vibrio cholerae	SAMN02603969	host-associated	11	Yes	4	LysR_substrate;TauE;SMC_N;HNH	—
1122619.SAMN02441372.KB892326_9	**typeI-A**	315,5	high	Pseudomonadota	Betaproteobacteria	Burkholderiales	Alcaligenaceae	Oligella	Oligella ureolytica	SAMN02441372	host-associated	10	Yes	4	SMC_N;Arm-DNA-bind_3;Phage_ir	—
412437.SAMN08324222.PNRD0100000(	**typeI-A**	313,4	high	Pseudomonadota	Gammaproteobacteria	Chromatiales	Chromatiaceae	Rheinheimera	Rheinheimera aquimaris	SAMN08324222	aquatic	11	Yes	4	RepD-like_N;SMC_N;TetR_C_13;A	—
659.SAMN07327695.PYMQ01000073_	**typeI-A**	311,9	high	Pseudomonadota	Gammaproteobacteria	Vibrionales	Vibrionaceae	Photobacterium	Photobacterium phosphoreum	SAMN07327695	host-associated	11	Yes	4	Cas_Csy4;PDDEXK_11;SMC_N;R	—
156424.SAMEA6595137.CADEDJ0100(	**typeI-A**	294,9	high	Pseudomonadota	Betaproteobacteria	Nitrosomonadales	Nitrosomonadaceae	Nitrosomonas	uncultured Nitrosomonas sp.	SAMEA6595137	anthropogenic	6	Yes	2	Csf1_N;STM3845	—
1095744.SAMN00761805.AJSW010000(	**typeI-A**	286	high	Pseudomonadota	Gammaproteobacteria	Pasteurellales	Pasteurellaceae	Haemophilus	Haemophilus parahaemolyticus	SAMN00761805	host-associated	3	Yes	2	RepD-like_N;AAA_15	—
707232.SAMN00113594.GG770443_1	**typeI-A**	282,8	high	Pseudomonadota	Gammaproteobacteria	Moraxellales	Moraxellaceae	Acinetobacter	Acinetobacter haemolyticus	SAMN00113594	anthropogenic	6	Yes	2	Csp	—
762966.SAMN00189152.GL883764_17	**typeI-A**	275,2	low	Pseudomonadota	Betaproteobacteria	Burkholderiales	Sutterellaceae	Parasutterella	Parasutterella excrementihominis	SAMN00189152	host-associated	10	Yes	4	AAA_21;AAA_19;SMC_N	—
1035188.SAMN00621708.AFUV010000	**typeI-A**	265,7	low	Pseudomonadota	Gammaproteobacteria	Pasteurellales	Pasteurellaceae	Haemophilus	Haemophilus pittmaniae	SAMN00621708	host-associated	10	Yes	4	Importin_rep;GDPD;DUF417;MIP	—
286133.SAMEA7846561.CAJJSA01000	**typeI-A**	259,3	low	Pseudomonadota	Betaproteobacteria	Burkholderiales	Sutterellaceae	Sutterella	uncultured Sutterella sp.	SAMEA7846561	host-associated	5	Yes	2	SMC_N	—
2796373.SAMN17054785.JAEKED0100	**typeI-B1**	282,8	high	Pseudomonadota	Gammaproteobacteria	Pseudomonadales	Pseudomonadaceae	Pseudomonas	Pseudomonas sp. TH07	SAMN17054785	host-associated	11	Yes	4	Mob_Pre;AAA_21;SHQ1;DUF7982	—
2760933.SAMN15642185.JACJDS0100	**typeI-B1**	280,4	high	Pseudomonadota	Gammaproteobacteria	Alteromonadales	Pseudoalteromonadaceae	Pseudoalteromonas	Pseudoalteromonas sp. SR44-8	SAMN15642185	aquatic	7	Yes	3	Peptidase_S66;Phage_integrase;A/	—
2778430.SAMN16816195.JADPIE0100(	**typeI-B2**	241	high	Bacillota	Clostridia	Halanaerobiales	Halarsenatibacteraceae	Halonatronomonas	Halonatronomonas betaini	SAMN16816195	aquatic	11	Yes	4	PglD_N;AAA_15;TolA_bind_tri;DEA	—
165186.SAMEA6149038.CACWTQ0100	**typeI-B2**	239,9	high	Bacillota	Clostridia	Eubacteriales	Oscillospiraceae	Ruminococcus	uncultured Ruminococcus sp.	SAMEA6149038	host-associated	11	Yes	4	YhcG_C;AAA_15;Eco57I;ResIII	—
1306154.SAMN17155236.JAEMBY0100	**typeI-B2**	236,2	high	Bacillota	Bacilli	Bacillales	Caryophanaceae	Rummeliibacillus	Rummeliibacillus suwonensis	SAMN17155236	anthropogenic	11	Yes	4	Phage_integrase;AAA_15;NLRC4_	—
56779.SAMN05421834.FTNC01000003	**typeI-B2**	234,3	high	Bacillota	Clostridia	Halanaerobiales	Halanaerobiaceae	Halanaerobium	Halanaerobium kushneri	SAMN05421834	terrestrial	11	Yes	4	DivIC;AAA_15;AAA	—
853.SAMN07350508.NMTY01000012_	**typeI-B2**	232,8	high	Bacillota	Clostridia	Eubacteriales	Oscillospiraceae	Faecalibacterium	Faecalibacterium prausnitzii	SAMN07350508	host-associated	8	Yes	4	RepA_N;AAA_21;DALR_1;DUF193	—
2841523.SAMN19374015.JAHLPV0100	**typeI-B2**	231,7	high	Bacillota	Clostridia	Acetivibrionales	Acetivibrionaceae	Acetivibrio	Acetivibrio sp. MSJd-27	SAMN19374015	host-associated	11	Yes	4	HTH_17;AAA_21;RE_LlaJI	—
2044939.SAMEA5278378.CAAGAI0100	**typeI-B2**	231,2	high	Bacillota	Clostridia	—	—	—	Clostridia bacterium	SAMEA5278378	host-associated	11	Yes	4	VWA_4;DUF6809;AAA_21;RRM_[	—
214851.SAMN10183615.SFKI01000027	**typeI-B2**	228,3	high	Bacillota	Clostridia	Eubacteriales	Oscillospiraceae	Subdoligranulum	Subdoligranulum variabile	SAMN10183615	host-associated	6	Yes	2	ALP_N	—
1798168.SAMN04487829.FOZB010000	**typeI-B2**	222,2	high	Bacillota	Clostridia	Lachnospirales	Lachnospiraceae	Pseudobutyrivibrio	Pseudobutyrivibrio sp. NOR37	SAMN04487829	host-associated	11	Yes	4	ORF6C;AAA_15;PSS;WD40_RLD	—
1898203.SAMEA8801389.CAJTEV0100	**typeI-B2**	215,1	borderline	Bacillota	Clostridia	Lachnospirales	Lachnospiraceae	—	Lachnospiraceae bacterium	SAMEA8801389	host-associated	10	Yes	4	RmlD_sub_bind;DUF6602;AAA_21	—
610130.SAMEA7202544.CAJFOY0100(	**typeI-B2**	214,5	borderline	Bacillota	Clostridia	Lachnospirales	Lachnospiraceae	Lacrimispora	Lacrimispora saccharolytica	SAMEA7202544	host-associated	7	Yes	4	ADH_Fe_C;AAA_21;DUF6602;Tsp	—
588581.SAMN00002727.ACXX0200000(	**typeI-B2**	213,4	borderline	Bacillota	Clostridia	Eubacteriales	Oscillospiraceae	Ruminiclostridium	Ruminiclostridium papyrosolvens	SAMN00002727	host-associated	10	Yes	4	Pentapeptide_4;AAA_21;tRNA-synt	—
195049.SAMEA7847077.CAJLTX01000	**typeI-B2**	210,9	borderline	Bacillota	Clostridia	Eubacteriales	Clostridiaceae	—	uncultured Clostridiaceae bacterium	SAMEA7847077	—	10	Yes	0	—	—
297314.SAMEA8805400.CAJUBW0100	**typeI-B2**	210	borderline	Bacillota	Clostridia	Lachnospirales	Lachnospiraceae	—	uncultured Lachnospiraceae bacterium	SAMEA8805400	host-associated	10	Yes	4	Na_Ala_symp;DUF6602;AAA_21;R	—
876091.SAMEA8805500.CAJUDW0100	**typeI-B2**	208	borderline	Bacillota	Clostridia	Eubacteriales	Oscillospiraceae	Oscillibacter	uncultured Oscillibacter sp.	SAMEA8805500	host-associated	10	Yes	4	HhH-GPD;AAA_21;AbrB	—
297314.SAMEA8805089.CAJTOV0100(	**typeI-B2**	206,3	borderline	Bacillota	Clostridia	Lachnospirales	Lachnospiraceae	—	uncultured Lachnospiraceae bacterium	SAMEA8805089	host-associated	6	Yes	3	DUF5630;AAA_21;DUF6602	—
77133.SAMEA7852836.CAJMQQ01000	**typeI-B2**	203,8	borderline	—	—	—	—	—	uncultured bacterium	SAMEA7852836	host-associated	7	Yes	4	AAA_21;DUF6602;HTH_3	—
2823316.SAMN18603874.CP073692_1	**typeI-B2**	200,7	borderline	Bacillota	Clostridia	Lachnospirales	Lachnospiraceae	Faecalicatena	Faecalicatena sp. Marseille-Q4148	SAMN18603874	host-associated	10	Yes	4	DUF6602;Lyase_8_N;AAA_21	—
297314.SAMEA8805250.CAJTUZ0100(	**typeI-B2**	198,2	borderline	Bacillota	Clostridia	Lachnospirales	Lachnospiraceae	—	uncultured Lachnospiraceae bacterium	SAMEA8805250	host-associated	10	Yes	4	Metallophos_2;AAA_21;DUF6602;[	—
1898203.SAMN10878285.SRYA010000	**typeI-B2**	194,1	borderline	Bacillota	Clostridia	Lachnospirales	Lachnospiraceae	—	Lachnospiraceae bacterium	SAMN10878285	host-associated	10	Yes	4	AAA_21;Fic;Uma2	—
297314.SAMEA8805667.CAJUQJ0100(	**typeI-B2**	181,5	borderline	Bacillota	Clostridia	Lachnospirales	Lachnospiraceae	—	uncultured Lachnospiraceae bacterium	SAMEA8805667	host-associated	7	Yes	4	Big_2;AAA_21;DUF6602;YchF-GTI	—
2013716.SAMN06767617.PHCH01000(	**typeI-C1**	349,7	high	Pseudomonadota	Betaproteobacteria	—	—	—	Betaproteobacteria bacterium HGW-B	SAMN06767617	terrestrial	11	Yes	4	NttA;DHOR;TOBE	—
1948756.SAMN06451412.DCUV01000(	**typeI-C1**	348	high	Spirochaetota	Spirochaetia	—	—	—	Spirochaetia bacterium UBA2205	SAMN06451412	anthropogenic	10	Yes	4	PD40;Na_Pi_cotrans	—
1619952.SAMN03340298.JYOD010000	**typeI-C1**	344,2	high	Pseudomonadota	Betaproteobacteria	Burkholderiales	Burkholderiaceae	—	Burkholderiaceae bacterium 16	SAMN03340298	terrestrial	11	Yes	4	Resolvase;Abi_C;DUF6320;UvrD-h	—
1951640.SAMN06454975.DKFJ010000	**typeI-C1**	343,8	high	Deferribacterota	Deferribacteres	Deferribacterales	Deferribacteraceae	—	Deferribacteraceae bacterium UBA679	SAMN06454975	anthropogenic	11	Yes	4	WYL;DLH;DAHP_synth_1;SIS_2	—
1743159.SAMN03400177.LOJI0100004	**typeI-C1**	342,2	high	Pseudomonadota	Betaproteobacteria	Burkholderiales	Burkholderiaceae	Polynucleobacter	Polynucleobacter yangtzensis	SAMN03400177	salinity	11	Yes	4	Asp_decarbox;dGTP_diPhyd_N	—
2052184.SAMN16056138.JAHDPY0100	**typeI-C1**	341,7	high	Thermodesulfobacteriota	Syntrophia	Syntrophales	Syntrophaceae	—	Syntrophaceae bacterium	SAMN16056138	host-associated	6	Yes	2	PglZ;BrxL_ATPase	—
1792290.SAMN17124794.JAEMUI0100	**typeI-C1**	340,8	high	Pseudomonadota	Gammaproteobacteria	Oceanospirillales	Oceanospirillaceae	Marinomonas	Marinomonas spartinae	SAMN17124794	aquatic	11	Yes	4	tRNA-synt_1;ABC_tran;KARI_N	—
2081523.SAMN20253999.JAIQFH0100(	**typeI-C1**	340,8	high	Acidobacteriota	Terriglobia	—	—	—	Terriglobia bacterium	SAMN20253999	aquatic	11	Yes	4	Big_3_5;Phage_integrase;HSDR_N	—
1315271.SAMD00169750.BJXY010000	**typeI-C1**	340,7	high	Pseudomonadota	Gammaproteobacteria	Alteromonadales	Pseudoalteromonadaceae	Pseudoalteromonas	Pseudoalteromonas tetraodonis	SAMD00169750	aquatic	11	Yes	4	Lipid_DES;SprT-like;HTH_26;Tn7_	—
1948417.SAMN06454857.DITJ0100004	**typeI-C1**	339,5	high	Pseudomonadota	Alphaproteobacteria	—	—	—	Alphaproteobacteria bacterium UBA61	SAMN06454857	anthropogenic	11	Yes	4	DarT;Macro;Response_reg;HTH_3	—
1977087.SAMN17574143.JAFEDJ0100	**typeI-C1**	336,5	high	Pseudomonadota	—	—	—	—	Pseudomonadota bacterium	SAMN17574143	host-associated	7	Yes	3	EAL;Acetyltransf_10	—
1898104.SAMN19298592.JAHJTN0100	**typeI-C1**	336,3	high	Bacteroidota	—	—	—	—	Bacteroidota bacterium	SAMN19298592	terrestrial	7	Yes	3	UvrD_C_2;DUF2813	—
208544.SAMEA6949913.CAIJIT010000	**typeI-C1**	336,2	high	Pseudomonadota	Betaproteobacteria	Burkholderiales	—	—	uncultured Burkholderiales bacterium	SAMEA6949913	aquatic	2	Yes	1	DDE_Tnp_ISAZ013	—
316.SAMN03352191.JYHV01000007_9	**typeI-C1**	336,2	high	Pseudomonadota	Gammaproteobacteria	Pseudomonadales	Pseudomonadaceae	Stutzerimonas	Stutzerimonas stutzeri	SAMN03352191	anthropogenic	11	Yes	4	MFS_1;Gas_vesicle;Reovirus_L2_7	—
1265490.SAMN02952889.JHVY010000	**typeI-C1**	336	high	Pseudomonadota	Gammaproteobacteria	Pseudomonadales	Pseudomonadaceae	Pseudomonas	Pseudomonas sp. URMO17WK12:I8	SAMN02952889	anthropogenic	11	Yes	4	RES;Reovirus_L2_7th;AsnC_trans_	—
129578.SAMN06648058.NQJF0100000	**typeI-C1**	336	high	Pseudomonadota	Gammaproteobacteria	Aeromonadales	Aeromonadaceae	Oceanimonas	Oceanimonas baumannii	SAMN06648058	anthropogenic	9	Yes	4	adh_short;HTH_23	—
562.SAMN14342453.CP050214_1061	**typeI-C1**	331,6	high	Pseudomonadota	Gammaproteobacteria	Enterobacterales	Enterobacteriaceae	Escherichia	Escherichia coli	SAMN14342453	host-associated	11	Yes	4	Sigma54_activat;CTD6	—
562.SAMN14342455.CP050216_4924	**typeI-C1**	331,6	high	Pseudomonadota	Gammaproteobacteria	Enterobacterales	Enterobacteriaceae	Escherichia	Escherichia coli	SAMN14342455	—	11	Yes	0	—	—
883078.SAMN02596753.KB375285_44	**typeI-C1**	331,1	high	Pseudomonadota	Alphaproteobacteria	Hyphomicrobiales	Nitrobacteraceae	Afipia	Afipia broomeae	SAMN02596753	host-associated	11	Yes	4	AAA_23;UvrD-helicase;Metallophos	—
91915.SAMN20927460.CP081941_236	**typeI-C1**	329,9	high	Pseudomonadota	Alphaproteobacteria	Acetobacterales	Acetobacteraceae	Asaia	Asaia bogorensis	SAMN20927460	anthropogenic	11	Yes	4	MC2;CTD6;Resolvase	—
2065379.SAMN08211554.CP025583_1	**typeI-C1**	329,3	high	Pseudomonadota	Alphaproteobacteria	Rhodobacterales	Paracoccaceae	Paracoccus	Paracoccus jeotgali	SAMN08211554	anthropogenic	11	Yes	4	PSK_trans_fac;DAO;TrbL	—
562.SAMN04563602.CP017061_4253	**typeI-C1**	327,4	high	Pseudomonadota	Gammaproteobacteria	Enterobacterales	Enterobacteriaceae	Escherichia	Escherichia coli	SAMN04563602	host-associated	11	Yes	4	DinI;DUF8087;Phage_portal;Termin	—
192812.SAMN18440232.JAGHQS0100(	**typeI-C1**	326,3	high	Pseudomonadota	Alphaproteobacteria	Sphingomonadales	Erythrobacteraceae	Qipengyuania	Qipengyuania flava	SAMN18440232	aquatic	11	Yes	4	Peptidase_S9;ThiC_Rad_SAM;Maz	—
2779529.SAMN16427092.JADCNH0100	**typeI-C1**	325,8	high	Pseudomonadota	Betaproteobacteria	Burkholderiales	Alcaligenaceae	Verticiella	Verticiella alkaliphila	SAMN16427092	host-associated	11	Yes	4	N6_Mtase;RadC;Phage_integrase	—
1124991.SAMN02603113.CP004345_2	**typeI-C1**	324,7	high	Pseudomonadota	Gammaproteobacteria	Enterobacterales	Morganellaceae	Morganella	Morganella morganii	SAMN02603113	host-associated	11	Yes	4	Peptidase_S24;Herpes_IE1;Phage_	—
2099680.SAMN19791121.JAIBBO0100(	**typeI-C1**	324,7	high	Methanobacteriota	Methanobacteria	Methanobacteriales	Methanobacteriaceae	—	Methanobacteriaceae archaeon	SAMN19791121	anthropogenic	2	Yes	1	NES_C_h	—
60549.SAMN05444165.FSRU01000001	**typeI-C1**	324,3	high	Pseudomonadota	Betaproteobacteria	Burkholderiales	Burkholderiaceae	Paraburkholderia	Paraburkholderia phenazinium	SAMN05444165	host-associated	11	Yes	4	DUF2268;Glyoxalase;TPR_5	—
1660129.SAMN03652473.MEEX01000(	**typeI-C1**	323	high	Pseudomonadota	Alphaproteobacteria	Caulobacterales	Caulobacteraceae	Phenylobacterium	Phenylobacterium sp. SCN 70-31	SAMN03652473	anthropogenic	8	Yes	4	PilM_2;Piwi;Sf6_terminase;PhyH	—
1381123.SAMN02299295.AYOD010000	**typeI-C1**	322,4	high	Pseudomonadota	Alphaproteobacteria	Hyphomicrobiales	Phyllobacteriaceae	Aliihoeflea	Aliihoeflea sp. 2WW	SAMN02299295	host-associated	11	Yes	4	EamA;QueF;PALP	—
1947379.SAMN06454808.DHXT010001	**typeI-C1**	320,9	high	Pseudomonadota	Betaproteobacteria	Burkholderiales	Comamonadaceae	Rhodoferax	Rhodoferax sp. UBA5149	SAMN06454808	terrestrial	11	Yes	4	RHH_6;Phage_integrase;HK;TMP-1	—
2053307.SAMN16091041.JADQBJ0100	**typeI-C1**	320,9	high	Thermodesulfobacteriota	Desulfobulbia	Desulfobulbales	Desulfobulbaceae	—	Desulfobulbaceae bacterium	SAMN16091041	aquatic	11	Yes	4	Thy1;SNase;RHH_7	—
2060068.SAMN08166491.PJZH010000	**typeI-C1**	320,2	high	Pseudomonadota	Gammaproteobacteria	Enterobacterales	Yersiniaceae	Chimaeribacter	Chimaeribacter coloradensis	SAMN08166491	host-associated	11	Yes	4	Bac_export_2;DUF5076;Peripla_BF	—

153809.SAMEA6151226.CACZZK0100(	**typeI-C1**	318,3	high	Pseudomonadota	—	—	—	—	uncultured Pseudomonadota bacterium	SAMEA6151226	host-associated	11	Yes	4	Resolvase;CdiA_C_tRNase;Inositol	—
2053615.SAMEA5279326.CAAGBC010	**typeI-C1**	316,6	high	Spirochaetota	Spirochaetia	—	—	—	Spirochaetia bacterium	SAMEA5279326	host-associated	11	Yes	4	YtrI_C;JetD_C;Macro	—
29522.SAMN10696518.SAXU01000001	**typeI-C1**	315,1	high	Spirochaetota	Spirochaetia	Brachyspirales	Brachyspiraceae	Brachyspira	Brachyspira aalborgi	SAMN10696518	host-associated	11	Yes	4	DUF4160;DUF2442;Eco57I;DUF78	—
470934.SAMN03068907.CP011427_15	**typeI-C1**	307,7	high	Pseudomonadota	Gammaproteobacteria	Enterobacterales	Erwiniaceae	Pantoea	Pantoea vagans	SAMN03068907	aquatic	11	Yes	4	Terminase_6N;Phage_portal;PPR;F	—
1830124.SAMN04620766.LWHH01000(	**typeI-C1**	304,8	high	Pseudomonadota	Alphaproteobacteria	Hyphomicrobiales	Brucellaceae	Ochrobactrum	Ochrobactrum sp. 3-3	SAMN04620766	terrestrial	11	Yes	4	Alpha-amy_C_pro;TM2;GSH-S_AT	—
1981031.SAMEA8805184.CAJTRX010(	**typeI-C1**	297,1	high	Thermodesulfobacteriota	Desulfovibrionia	Desulfovibrionales	Desulfovibrionaceae	Mailhella	uncultured Mailhella sp.	SAMEA8805184	host-associated	11	Yes	4	ADP_ribosyl_GH;DUF3801;T1RH-I	—
1395944.SAMN11959953.VDMN010000	**typeI-C1**	296,9	high	Pseudomonadota	Alphaproteobacteria	Hyphomicrobiales	Rhizobiaceae	Aliirhizobium	Aliirhizobium smilacinae	SAMN11959953	anthropogenic	11	Yes	4	Resolvase;DUF7354;PAP2_3;Phag	—
1940281.SAMN19405844.JAHRFE010(	**typeI-C1**	292,7	high	Pseudomonadota	Alphaproteobacteria	Hyphomicrobiales	Rhizobiaceae	Hoeflea	Hoeflea sp.	SAMN19405844	terrestrial	6	Yes	2	Resolvase;DUF302	—
440079.SAMN07680236.NXBQ0100000	**typeI-C1**	285,9	high	Pseudomonadota	Gammaproteobacteria	Aeromonadales	Aeromonadaceae	Aeromonas	Aeromonas bivalvium	SAMN07680236	host-associated	11	Yes	4	CENP-U;CrtC;FtsX	—
2529835.SAMN10994878.SJOH010000	**typeI-C1**	267,4	high	Pseudomonadota	Gammaproteobacteria	Moraxellales	Moraxellaceae	Acinetobacter	Acinetobacter sp. ANC 3781	SAMN10994878	anthropogenic	11	Yes	4	HTH_3;Flk_N;UCP010056;Phage_	—
2529840.SAMN10994883.SJOC010000	**typeI-C1**	258,5	high	Pseudomonadota	Gammaproteobacteria	Moraxellales	Moraxellaceae	Acinetobacter	Acinetobacter sp. ANC 4216	SAMN10994883	anthropogenic	6	Yes	3	Thiolase_N;3HCDH_N	—
1648519.SAMEA8395269.CAJPST010(	**typeI-C1**	253,1	high	Pseudomonadota	Gammaproteobacteria	Pasteurellales	Pasteurellaceae	Mesocricetibacter	uncultured Mesocricetibacter sp.	SAMEA8395269	host-associated	6	Yes	2	PsiE;Y1_Tnp	—
2840708.SAMN15816948.DVFN010001	**typeI-C2**	355,8	high	Bacillota	Clostridia	Eubacteriales	Oscillospiraceae	Candidatus Avoscillosp	Candidatus Avoscillospira stercorigallir	SAMN15816948	host-associated	6	Yes	2	HSP90;Methyltransf_4	—
512298.SAMEA4891445.UQZO0100001	**typeI-C2**	353,5	high	Bacillota	Clostridia	Eubacteriales	Oscillospiraceae	Subdoligranulum	uncultured Subdoligranulum sp.	SAMEA4891445	host-associated	9	Yes	4	IstB_IS21;DUF3231;Zn_Ribbon_1;I	—
1586779.SAMEA4891222.UWSG01000(	**typeI-C2**	353,2	high	Bacillota	Clostridia	Lachnospirales	Lachnospiraceae	Lachnoclostridium	uncultured Lachnoclostridium sp.	SAMEA4891222	—	11	Yes	0	—	—
1673721.SAMEA3481237.LN869529_3	**typeI-C2**	352,8	high	Bacillota	Clostridia	Eubacteriales	—	Intestinimonas	Intestinimonas massiliensis (ex Afouda	SAMEA3481237	—	11	Yes	0	—	—
1898207.SAMN13894142.JAAYPT0100	**typeI-C2**	351	high	Bacillota	Clostridia	Eubacteriales	—	—	Clostridiales bacterium	SAMN13894142	host-associated	11	Yes	4	Hydrolase;HTH_3;WYL	—
1349765.SAMD00046312.BCQJ010000	**typeI-C2**	350,5	high	Bacillota	Bacilli	Lactobacillales	Enterococcaceae	Enterococcus	Enterococcus canis	SAMD00046312	host-associated	8	Yes	4	SieB;DUF6095;MamF_MmsF	—
1139219.SAMN02596962.ASWK01000(	**typeI-C2**	350,5	high	Bacillota	Bacilli	Lactobacillales	Enterococcaceae	Enterococcus	Enterococcus dispar	SAMN02596962	host-associated	11	Yes	0	—	—
297314.SAMEA8805450.CAJUDY0100(	**typeI-C2**	348,5	high	Bacillota	Clostridia	Lachnospirales	Lachnospiraceae	—	uncultured Lachnospiraceae bacterium	SAMEA8805450	host-associated	11	Yes	4	DUF6491;DUF3795;DUF4096	—
165185.SAMEA4891893.URQO010001	**typeI-C2**	347,6	high	Bacillota	Clostridia	Eubacteriales	Eubacteriaceae	Eubacterium	uncultured Eubacterium sp.	SAMEA4891893	host-associated	3	Yes	2	Fer4_5;TfoX_C	—
2292270.SAMN09736896.QUKQ01000(	**typeI-C2**	346,7	high	Bacillota	Clostridia	Eubacteriales	Oscillospiraceae	—	Ruminococcaceae bacterium TF06-43	SAMN09736896	host-associated	11	Yes	4	4HBT;DUF3783;Phage_integrase;F	—
1965575.SAMN06473646.NFKJ010000	**typeI-C2**	345,7	high	Bacillota	Clostridia	Lachnospirales	Lachnospiraceae	Lachnoclostridium	Lachnoclostridium sp. An181	SAMN06473646	host-associated	11	Yes	4	RelB;Fic;DnaB_C;YicC-like_C	—
904996.SAMEA4891869.URPY0100000	**typeI-C2**	342,1	high	Bacillota	Clostridia	Eubacteriales	Eubacteriaceae	Anaerofustis	uncultured Anaerofustis sp.	SAMEA4891869	host-associated	11	Yes	4	S-AdoMet_synt_C;EIIA-man;Dak2;I	—
344338.SAMEA7847122.CAJLJV01000	**typeI-C2**	341,6	high	Bacillota	—	—	—	—	uncultured Bacillota bacterium	SAMEA7847122	host-associated	11	Yes	4	Cadherin-like;DUF4430;Glyco_hydr	—
199.SAMEA5905261.CABPUP0100000	**typeI-C2**	336,6	high	Campylobacterota	Epsilonproteobacteria	Campylobacterales	Campylobacteraceae	Campylobacter	Campylobacter concisus	SAMEA5905261	host-associated	11	Yes	4	ACR_tran;CinA;tRNA-synt_1	—
1123489.SAMN02441545.KE386795_1	**typeI-C2**	334,4	high	Bacillota	Negativicutes	Veillonellales	Veillonellaceae	Veillonella	Veillonella magna	SAMN02441545	host-associated	11	Yes	4	UvrD-helicase;HTH_38;KAP_NTPa	—
1898207.SAMN17800681.JAGZBI0100	**typeI-C2**	333,4	high	Bacillota	Clostridia	Eubacteriales	—	—	Clostridiales bacterium	SAMN17800681	host-associated	11	Yes	4	ABC_membrane;DUF3810;DUF11;	—
707003.SAMEA7847144.CAJLUT0100(	**typeI-C2**	331,7	high	Bacillota	Clostridia	Eubacteriales	Oscillospiraceae	—	uncultured Oscillospiraceae bacterium	SAMEA7847144	host-associated	11	Yes	4	PadR;DUF4131;NLPC_P60;AAA_	—
876091.SAMEA8805797.CAJUQM0100	**typeI-C2**	331,6	high	Bacillota	Clostridia	Eubacteriales	Oscillospiraceae	Oscillibacter	uncultured Oscillibacter sp.	SAMEA8805797	host-associated	6	Yes	3	Phage_integrase;Zn_Ribbon_1;Pkir	—
2044939.SAMN16342949.JAFRHU010(	**typeI-C2**	328,8	high	Bacillota	Clostridia	—	—	—	Clostridia bacterium	SAMN16342949	host-associated	11	Yes	4	AAA_25;Sigma70_r4_2;Response_	—
2485925.SAMEA6152165.CADBJK010(	**typeI-C2**	328,6	high	Bacillota	Clostridia	Eubacteriales	Oscillospiraceae	—	Oscillospiraceae bacterium	SAMEA6152165	host-associated	11	Yes	4	DUF1979;Arg_tRNA_synt_N;DUF8	—
1161902.SAMN00829151.AZKM010000	**typeI-C2**	327,6	high	Bacillota	Clostridia	Peptostreptococcales	Anaerovoracaceae	—	[Eubacterium] nodatum	SAMN00829151	host-associated	9	Yes	4	HTH_28;MinE;HTH_29	—
1328.SAMEA7846664.CAJLPW010000	**typeI-C2**	327	high	Bacillota	Bacilli	Lactobacillales	Streptococcaceae	Streptococcus	Streptococcus anginosus	SAMEA7846664	host-associated	11	Yes	4	RrnaAD;DUF7822;adh_short;DUF4	—
888721.SAMN03897724.CP016202_58	**typeI-C2**	325,2	high	Bacillota	Clostridia	Peptostreptococcales	Anaerovoracaceae	—	[Eubacterium] minutum	SAMN03897724	host-associated	11	Yes	4	Methylase_S;N6_Mtase;HTH_21;D	—
172733.SAMEA7846885.CAJKUA0100(	**typeI-C2**	318,7	high	Bacillota	Clostridia	Eubacteriales	—	—	uncultured Eubacteriales bacterium	SAMEA7846885	host-associated	10	Yes	4	AAA_12;AAA_11	—
1123304.SAMN02441723.AQYA010000	**typeI-C2**	316,1	high	Bacillota	Bacilli	Lactobacillales	Streptococcaceae	Streptococcus	Streptococcus henryi	SAMN02441723	anthropogenic	11	Yes	4	Methyltransf_5;RrnaAD;Abi_C;DUF	—
1715105.SAMN04477515.KV797949_8	**typeI-C2**	314,8	high	Bacillota	Bacilli	Lactobacillales	Aerococcaceae	Aerococcus	Aerococcus sp. HMSC062A02	SAMN04477515	host-associated	11	Yes	4	SWI2_SNF2;EF_EFCAB10_C;ZinT	—
2044939.SAMEA5278349.CAAGAO010	**typeI-C2**	314,8	high	Bacillota	Clostridia	—	—	—	Clostridia bacterium	SAMEA5278349	host-associated	11	Yes	4	P-mevalo_kinase;DUF4467;Resolv	—
1965587.SAMN06473663.NFJU010000	**typeI-C2**	313,4	high	Bacillota	Clostridia	Eubacteriales	Acutalibacteraceae	Anaeromassilibacillus	Anaeromassilibacillus sp. An200	SAMN06473663	host-associated	7	Yes	3	MatE;Y1_Tnp	—
1123312.SAMN02256411.KB904582_1	**typeI-C2**	312,4	high	Bacillota	Bacilli	Lactobacillales	Streptococcaceae	Streptococcus	Streptococcus ovis	SAMN02256411	host-associated	11	Yes	4	Peptidase_MA_2;RrnaAD;Abi_C;HI	—
83427.SAMEA7848085.CAJMDY01000	**typeI-C2**	279,8	high	Bacillota	Bacilli	Lactobacillales	Streptococcaceae	Streptococcus	uncultured Streptococcus sp.	SAMEA7848085	host-associated	11	Yes	4	BPD_transp_1;CBM_11;tRNA-Thr_	—
331679.SAMN11653953.VBTH0100002	**typeI-C2**	266,2	high	Bacillota	Bacilli	Lactobacillales	Lactobacillaceae	Pediococcus	Pediococcus stilesii	SAMN11653953	host-associated	6	Yes	2	CoV_S2;VanZ	—
1965645.SAMN06473737.NFIA0100002	**typeI-C3**	357,5	high	Bacteroidota	Bacteroidia	Bacteroidales	Rikenellaceae	Alistipes	Alistipes sp. An54	SAMN06473737	host-associated	11	Yes	4	Metallophos;HTH_17	—
1872444.SAMN16346988.JAFXLG0100	**typeI-C3**	325,9	high	Bacteroidota	Bacteroidia	Bacteroidales	Rikenellaceae	Alistipes	Alistipes sp.	SAMN16346988	host-associated	11	Yes	4	tRNA_edit;NTP_transf_5;Peptidase	—
2840757.SAMN15817056.JADILV0100(	**typeI-C3**	318,1	high	Bacteroidota	Bacteroidia	Bacteroidales	—	Candidatus Cryptobact	Candidatus Cryptobacteroides avicola	SAMN15817056	host-associated	8	Yes	4	TatA_B_E;GFO_IDH_MocA;Amino	—
505249.SAMN07360937.NWVX010000	**typeI-C3**	308,9	borderline	Campylobacterota	Epsilonproteobacteria	Campylobacterales	Arcobacteraceae	Malaciobacter	Malaciobacter marinus	SAMN07360937	terrestrial	10	Yes	4	DUF808;AhpC-TSA;TRAP_alpha;M	—
1802259.SAMN04314634.MICF010000	**typeI-C3**	305,8	borderline	Campylobacterota	Epsilonproteobacteria	Campylobacterales	Sulfurimonadaceae	Sulfurimonas	Sulfurimonas sp. RIFOXYD12_FULL_	SAMN04314634	terrestrial	7	Yes	4	Putative_G5P;zf-CRD;ParE_toxin	—
2030927.SAMN11294446.SUYX010000	**typeI-C3**	293,1	low	Bacteroidota	Bacteroidia	Bacteroidales	—	—	Bacteroidales bacterium	SAMN11294446	host-associated	10	Yes	4	LPD3;UDPG_MGDP_dh_N;NTP_t	—
2590021.SAMN12085941.CP041166_5	**typeI-C3**	282,3	low	Campylobacterota	Epsilonproteobacteria	Campylobacterales	Sulfurimonadaceae	Sulfurimonas	Sulfurimonas xiamenensis	SAMN12085941	anthropogenic	10	Yes	4	Lipase_GDSL_2;Phage_integrase;F	—
315271.SAMN14567401.CP051206_10	**typeI-C3**	278	low	Cyanobacteriota	Cyanophyceae	Nostocales	Aphanizomenonaceae	Dolichospermum	Dolichospermum flos-aquae	SAMN14567401	aquatic	10	Yes	4	DNA_pol_B;PDDEXK_7;DNA_pol3	—
2052166.SAMN11164354.DUDU010000	**typeI-C3**	264,3	low	Candidatus Melainabacte	—	—	—	—	Candidatus Melainabacteria bacterium	SAMN11164354	aquatic	10	Yes	4	MinC_C;DivIVA;ABC2_membrane;	—
152509.SAMEA6946680.CAIMGI01000	**typeI-C3**	228,7	low	Bacteroidota	—	—	—	—	uncultured Bacteroidota bacterium	SAMEA6946680	aquatic	6	Yes	3	SdpI;DnaB_C	—
152509.SAMEA6951679.CAIKXD01000	**typeI-C3**	220,9	low	Bacteroidota	—	—	—	—	uncultured Bacteroidota bacterium	SAMEA6951679	aquatic	10	Yes	4	DnaB_C;EppA_BapA;Zeta_toxin	—
152509.SAMEA6953983.CAINDA0100(	**typeI-C3**	218,5	low	Bacteroidota	—	—	—	—	uncultured Bacteroidota bacterium	SAMEA6953983	aquatic	10	Yes	4	WCX;UDG;DnaB_C	—
194843.SAMEA6951804.CAIQUS0100(	**typeI-C3**	214,5	low	Bacteroidota	Bacteroidia	Bacteroidales	—	—	uncultured Bacteroidales bacterium	SAMEA6951804	aquatic	10	Yes	4	Metallophos;DnaB_C;Y_phosphatas	—
2026728.SAMN16425675.JADJEU0100(	**typeI-C3**	212	low	Bacteroidota	Flavobacteriia	Flavobacteriales	Crocinitomicaceae	—	Crocinitomicaceae bacterium	SAMN16425675	anthropogenic	10	Yes	4	DnaB_C;Ubiquitin_RHG40_C;Ocnu	—
1198309.SAMN04102813.LKEF010000	**typeII-A1**	291,5	high	Pseudomonadota	Gammaproteobacteria	Pseudomonadales	Pseudomonadaceae	Pseudomonas	Pseudomonas fluorescens	SAMN04102813	host-associated	11	Yes	4	AAA_13;STM3845	—
2789216.SAMN12641115.CP043489_8	**typeII-A1**	284,2	high	Pseudomonadota	Alphaproteobacteria	Hyphomicrobiales	Xanthobacteraceae	Labrys	Labrys sp. KNU-23	SAMN12641115	host-associated	11	Yes	4	UvrB_inter;STM3845;Polysacc_lya	—
2013740.SAMN06767641.PHBJ010000	**typeII-A1**	280,2	high	—	Deltaproteobacteria	—	—	—	Deltaproteobacteria bacterium HGW-D	SAMN06767641	terrestrial	7	Yes	3	Transposase_20;STM3845;DUF31	—
1969471.SAMN08180044.PLPS010000	**typeII-A1**	274,9	high	Acidobacteriota	Terriglobia	Terriglobales	Acidobacteriaceae	Granulicella	Granulicella sp.	SAMN08180044	temperature	7	Yes	3	STM3845	—
42354.SAMN05216300.FNOE0100003	**typeII-A1**	274,4	high	Pseudomonadota	Betaproteobacteria	Nitrosomonadales	Nitrosomonadaceae	Nitrosomonas	Nitrosomonas oligotropha	SAMN05216300	aquatic	11	Yes	4	DUF4179;DUF3653;STM3845	—
1737357.SAMN14908366.JACHEG010(	**typeII-A1**	272,9	high	Pseudomonadota	Alphaproteobacteria	Hyphomicrobiales	Rhizobiaceae	Rhizobium	Rhizobium wenxiniae	SAMN14908366	host-associated	11	Yes	4	Phage_integrase;STM3845	—
358.SAMN16786093.CP072309_21	**typeII-A1**	272,7	high	Pseudomonadota	Alphaproteobacteria	Hyphomicrobiales	Rhizobiaceae	Agrobacterium	Agrobacterium tumefaciens	SAMN16786093	anthropogenic	11	Yes	4	DUF8208;STM3845;Phage_integra	—
1954207.SAMN06299364.MVDH010000	**typeII-A1**	271,4	high	Pseudomonadota	Gammaproteobacteria	Cellvibrionales	Cellvibrionaceae	Cellvibrio	Cellvibrio sp. 79	SAMN06299364	host-associated	11	Yes	4	AAA_31;STM3845;DUF3037;HipA_	—
2026735.SAMN18119946.JAGOGO010	**typeII-A1**	271,4	high	Myxococcota	Myxococcia	—	—	—	Deltaproteobacteria bacterium	SAMN18119946	anthropogenic	11	Yes	4	PPV_E1_C;STM3845;DUF499	—
1173101.SAMN18839544.CP073754_2	**typeII-A1**	271,2	high	Pseudomonadota	Gammaproteobacteria	Methylococcales	Methylococcaceae	Methylomonas	Methylomonas paludis	SAMN18839544	aquatic	11	Yes	4	Trypco2;STM3845;Virulence_RhuM	—
2569093.SAMEA6954955.CAILKY0100(	**typeII-A1**	270,4	high	Acidobacteriota	Terriglobia	—	—	—	uncultured Acidobacteriia bacterium	SAMEA6954955	aquatic	11	Yes	4	AAA_15;STM3845;Pkinase	—
58169.SAMN10095182.RAHG0100000	**typeII-A1**	268,4	high	Pseudomonadota	Gammaproteobacteria	Enterobacterales	Yersiniaceae	Rahnella	Rahnella inusitata	SAMN10095182	host-associated	11	Yes	4	Ank;Glyco_transf_15;STM3845;Og	—
1978525.SAMN16426060.JADJSP0100	**typeII-A1**	267,6	high	Pseudomonadota	Alphaproteobacteria	Sphingomonadales	—	—	Sphingomonadales bacterium	SAMN16426060	anthropogenic	11	Yes	4	DUF4189;STM3845;Aida_N	—
2735259.SAMN14884351.JABJWH010(	**typeII-A1**	266,9	high	Pseudomonadota	Gammaproteobacteria	Oceanospirillales	Alcanivoracaceae	Alloalcanivorax	Alloalcanivorax profundimaris	SAMN14884351	aquatic	11	Yes	4	NERD;DUF7042;STM3845;ParD_	—
1895729.SAMN05660584.MKUP010000	**typeII-A1**	266,8	high	Pseudomonadota	Betaproteobacteria	Burkholderiales	—	—	Burkholderiales bacterium 67-32	SAMN05660584	anthropogenic	11	Yes	4	LysR_substrate;STM3845;Sigma54	—
2571749.SAMN11479196.CP039695_1	**typeII-A1**	266,2	high	Pseudomonadota	Alphaproteobacteria	Sphingomonadales	Sphingomonadaceae	Novosphingobium	Novosphingobium sp. EMRT-2	SAMN11479196	aquatic	11	Yes	4	NAD_binding_10;Phage_int_M;STN	—
57320.SAMEA104288281.LT907975_1	**typeII-A1**	265	high	Thermodesulfobacteriota	Desulfobulbia	Desulfobulbales	Desulfobulbaceae	Pseudodesulfovibrio	Pseudodesulfovibrio profundus	SAMEA104288281	aquatic	11	Yes	4	rve;STM3845;DDE_Tnp_ISL3	—
1292034.SAMN02470743.APMP010000	**typeII-A1**	264,2	high	Pseudomonadota	Alphaproteobacteria	Caulobacterales	Caulobacteraceae	Caulobacter	Caulobacter vibrioides	SAMN02470743	anthropogenic	11	Yes	4	AAA_33;STM3845;EcsC;DNA_gyr	—
34002.SAMN04489859.FODE0100002	**typeII-A1**	262,9	high	Pseudomonadota	Alphaproteobacteria	Rhodobacterales	Paracoccaceae	Paracoccus	Paracoccus alcaliphilus	SAMN04489859	host-associated	11	Yes	4	UDG;STM3845;Iso_dh;VirionAssen	—
1947551.SAMN06455167.DDVW01000	**typeII-A1**	257,6	high	Pseudomonadota	Gammaproteobacteria	Lysobacterales	Lysobacteraceae	Stenotrophomonas	Stenotrophomonas sp. UBA2302	SAMN06455167	host-associated	6	Yes	2	ANT;DDE_Tnp_ISL3	—
2485107.SAMN10361094.SMBO010000	**typeII-A1**	257,5	high	Pseudomonadota	Alphaproteobacteria	Sphingomonadales	Sphingomonadaceae	Novosphingobium	Novosphingobium sp. PhB57	SAMN10361094	terrestrial	7	Yes	3	STM3845;DUF3800;MmcB-like	—
1122188.SAMN02745674.FUXP010000	**typeII-A1**	256,8	high	Pseudomonadota	Gammaproteobacteria	Lysobacterales	Lysobacteraceae	Novilysobacter	Novilysobacter spongiicola	SAMN02745674	aquatic	11	Yes	4	PIN7;STM3845;PDH_E1_M	—

1909294.SAMEA4707559.UBPT010000	**typeII-A1**	255,3	high	Pseudomonadota	Alphaproteobacteria	Hyphomicrobiales	—	—	Hyphomicrobiales bacterium	SAMEA4707559	anthropogenic	6	Yes	2	Aminotran_5	—
2769289.SAMN15951386.JACVAP010	**typeII-A1**	253,3	high	Pseudomonadota	Gammaproteobacteria	Lysobacterales	Lysobacteraceae	Xanthomonas	Xanthomonas sp. XNM01	SAMN15951386	host-associated	11	Yes	4	MM_CoA_mutase;STM3845;C2c1_	—
1953413.SAMN06450843.DLMJ010000	**typeII-A1**	252,9	high	Candidatus Binatota	Candidatus Binatia	Candidatus Binatales	Candidatus Binataceae	Candidatus Binatus	Candidatus Binatus soli	SAMN06450843	salinity	11	Yes	4	DUF4140;STM3845;Methyltransf_2	—
2032568.SAMN07572155.NTJF010000	**typeII-A1**	252,8	high	Pseudomonadota	Gammaproteobacteria	Lysobacterales	Lysobacteraceae	—	Lysobacteraceae bacterium NML93-08	SAMN07572155	anthropogenic	11	Yes	4	PDH_E1_M;GvpG	—
1914330.SAMN07620071.PBYA010000	**typeII-A1**	252,5	high	Pseudomonadota	Gammaproteobacteria	Salinisphaerales	Salinisphaeraceae	Salinisphaera	Salinisphaera sp.	SAMN07620071	aquatic	11	Yes	4	Ku;STM3845;LigD_Prim-Pol;tRNA_	—
2268192.SAMN10966423.VGGK010000	**typeII-A1**	252,1	high	Chlorobiota	—	—	—	—	Chlorobiota bacterium	SAMN10966423	aquatic	11	Yes	4	BH4;M_Eco57I_C;STM3845;YfhO	—
1978525.SAMN18061488.JAFKLT0100	**typeII-A1**	251,8	high	Pseudomonadota	Alphaproteobacteria	Sphingomonadales	—	—	Sphingomonadales bacterium	SAMN18061488	anthropogenic	9	Yes	4	Aminotran_4;Phage_integrase;STM	—
1648404.SAMN03565637.CP011310_2	**typeII-A1**	247,5	high	Pseudomonadota	Alphaproteobacteria	Sphingomonadales	Erythrobacteraceae	Aurantiacibacter	Aurantiacibacter atlanticus	SAMN03565637	temperature	11	Yes	4	Phage_int_M;Nal1_C;HTH_Tnp_1	—
86027.SAMEA5364554.CAADGS01000	**typeII-A1**	244,4	high	Pseudomonadota	Betaproteobacteria	—	—	—	uncultured beta proteobacterium	SAMEA5364554	anthropogenic	8	Yes	4	Arm-DNA-bind_3;STM3845;Tag1_N	—
157176.SAMEA5954144.CABVRX0100	**typeII-A1**	241,5	high	Nitrospirota	Nitrospiria	Nitrospirales	Nitrospiraceae	Nitrospira	uncultured Nitrospira sp.	SAMEA5954144	anthropogenic	11	Yes	4	DUF7315;STM3845;GST_N_3;PilZ	—
1898112.SAMN11532965.VBWB010000	**typeII-A1**	239,2	high	Pseudomonadota	Alphaproteobacteria	Rhodospirillales	Rhodospirillaceae	—	Rhodospirillaceae bacterium	SAMN11532965	terrestrial	8	Yes	4	dnstrm_HI1420;STM3845;Phage_ir	—
2052142.SAMN08179164.PLIC0100000	**typeII-A1**	234	high	Acidobacteriota	Terriglobia	Terriglobales	Acidobacteriaceae	—	Acidobacteriaceae bacterium	SAMN08179164	temperature	11	Yes	4	Radical_SAM;STM3845;DEAD;Bac	—
2780381.SAMN16434398.JADCLP010	**typeII-A1**	232,7	high	Pseudomonadota	Alphaproteobacteria	Sphingomonadales	Erythrobacteraceae	—	Erythrobacteraceae bacterium E2-1 Ye	SAMN16434398	host-associated	11	Yes	4	STM3845;ABC_tran;Peptidase_M3	—
1946060.SAMN06455422.DFTV010000	**typeII-A1**	229	high	Pseudomonadota	Gammaproteobacteria	Chromatiales	Chromatiaceae	Arsukibacterium	Arsukibacterium sp. UBA4203	SAMN06455422	aquatic	11	Yes	4	DUF6500;DUF4440;STM3845;AAA	—
2026749.SAMN07619267.PAAX010000	**typeII-A1**	228,4	high	Ignavibacteriota	—	—	—	—	Ignavibacteriota bacterium	SAMN07619267	anthropogenic	11	Yes	4	Peptidase_M1;STM3845;PhdYeFM	—
1977087.SAMN19298580.JAHJTB0100	**typeII-A1**	225,3	high	Pseudomonadota	—	—	—	—	Pseudomonadota bacterium	SAMN19298580	terrestrial	11	Yes	4	MerR_1;Competence;Virul_fac_Brk	—
172733.SAMEA7846713.CAJJME0100	**typeII-A1**	211,1	high	Bacillota	Clostridia	Eubacteriales	—	—	uncultured Eubacteriales bacterium	SAMEA7846713	host-associated	4	Yes	3	NTP_transferase;Flg_new_2	—
2721131.SAMEA7202576.CAJFVX010	**typeII-A1**	209,4	high	Bacillota	Clostridia	—	—	Candidatus Howiella	Candidatus Howiella intestinavium	SAMEA7202576	host-associated	8	Yes	4	Mem_trans;HTH_3;STM3845;AAA_	—
1898203.SAMN11294595.SVEQ010000	**typeII-A1**	206,5	high	Bacillota	Clostridia	Lachnospirales	Lachnospiraceae	—	Lachnospiraceae bacterium	SAMN11294595	host-associated	11	Yes	4	PglD_N;Bac_transf;STM3845;DUF	—
253830.SAMEA6954216.CAIMLR01000	**typeII-A1**	205,6	high	Myxococcota	Myxococcia	Myxococcales	—	—	uncultured Myxococcales bacterium	SAMEA6954216	aquatic	11	Yes	4	Methylase_S;Phage_capsid_4;STM	—
1938870.SAMN06273570.OCMY010000	**typeII-A1**	200	high	Pseudomonadota	Gammaproteobacteria	Enterobacterales	Erwiniaceae	Pantoea	Candidatus Pantoea floridensis	SAMN06273570	host-associated	11	Yes	4	GPW_gp25;Phage_spike;STM3845	—
717962.SAMEA2272068.FP929038_45	**typeII-A1**	198,1	high	Bacillota	Clostridia	Lachnospirales	Lachnospiraceae	Pseudocoprococcus	Pseudocoprococcus catus	SAMEA2272068	host-associated	11	Yes	4	ThsA_Macro;STM3845	—
2315688.SAMN09976312.QXJC010000	**typeII-A1**	196,5	high	Pseudomonadota	Betaproteobacteria	Burkholderiales	Comamonadaceae	Simplicispira	Simplicispira hankyongi	SAMN09976312	host-associated	8	Yes	4	AAA_13;TPP_enzyme_N;STM3845	—
1218077.SAMD00000366.BAYC010000	**typeII-A1**	196,2	high	Pseudomonadota	Betaproteobacteria	Burkholderiales	Burkholderiaceae	Paraburkholderia	Paraburkholderia fungorum	SAMD00000366	aquatic	11	Yes	4	BPD_transp_1;STM3845;AAA_15	—
61635.SAMEA4532333.FO681348_167	**typeII-A1**	157,3	low	Mycoplasmatota	Mollicutes	Acholeplasmatales	Acholeplasmataceae	Acholeplasma	Acholeplasma brassicae	SAMEA4532333	anthropogenic	10	Yes	4	P5CR_dimer;UPF0158;STM3845;D	—
1173724.SAMN00990758.AAXWDZ010	**typeII-A2**	303,6	high	Pseudomonadota	Gammaproteobacteria	Enterobacterales	Enterobacteriaceae	Citrobacter	Citrobacter freundii	SAMN00990758	host-associated	11	Yes	4	Membra_charge;Psu;Ogr_Delta	—
1913989.SAMN07620381.PALV010000	**typeII-A2**	301,5	high	Pseudomonadota	Gammaproteobacteria	—	—	—	Gammaproteobacteria bacterium	SAMN07620381	aquatic	11	Yes	4	Peptidase_S8;AAA;STM3845;Meth	—
172194.SAMEA9694482.CAJXTU0100	**typeII-A2**	292,9	high	Pseudomonadota	Gammaproteobacteria	Thiotrichales	Piscirickettsiaceae	Cycloclasticus	uncultured Cycloclasticus sp.	SAMEA9694482	temperature	11	Yes	4	Methylase_S;STM3845	—
1768806.SAMN04299486.LSKJ010001	**typeII-A2**	289	borderline	Pseudomonadota	Alphaproteobacteria	Rhodospirillales	Rhodospirillaceae	—	Rhodospirillaceae bacterium CCH5-H1	SAMN04299486	aquatic	10	Yes	4	2-Hacid_dh_C;Arabinose_bd	—
331696.SAMN09742692.CP031417_26	**typeII-A2**	258,9	borderline	Pseudomonadota	Alphaproteobacteria	Hyphomicrobiales	Xanthobacteraceae	Pseudolabrys	Pseudolabrys taiwanensis	SAMN09742692	terrestrial	10	Yes	4	Avidin;PMSR;DUF2442;DUF4160	—
2282150.SAMN18059875.JAFLCS0100	**typeII-A2**	258,1	borderline	Pseudomonadota	Alphaproteobacteria	Caulobacterales	—	—	Caulobacterales bacterium	SAMN18059875	anthropogenic	10	Yes	4	ABC2_membrane_4;ThiC_Rad_SA	—
154336.SAMEA9694234.CAJXUT0100	**typeII-A2**	244,5	low	Pseudomonadota	Gammaproteobacteria	Alteromonadales	Colwelliaceae	Colwellia	uncultured Colwellia sp.	SAMEA9694234	temperature	4	Yes	3	Zn_ribbon_15;DUF6622;Pentapepti	—
1898207.SAMN13894018.JAAYKZ0100	**typeII-A3**	308,6	high	Bacillota	Clostridia	Eubacteriales	—	—	Clostridiales bacterium	SAMN13894018	temperature	2	Yes	1	STM3845	—
720554.SAMN02261428.CP003065_17	**typeII-A3**	307,2	high	Bacillota	Clostridia	Acetivibrionales	Acetivibrionaceae	Acetivibrio	Acetivibrio clariflavus	SAMN02261428	terrestrial	11	Yes	0	—	—
1125747.SAMD00036597.BAEK010000	**typeII-A3**	304,2	high	Pseudomonadota	Gammaproteobacteria	Alteromonadales	Alteromonadaceae	Paraglaciecola	Paraglaciecola chathamensis	SAMD00036597	aquatic	6	Yes	2	STM3845;Macro	—
400153.SAMN12559023.VRLR0100001	**typeII-A3**	301,7	high	Pseudomonadota	Gammaproteobacteria	Chromatiales	Chromatiaceae	Rheinheimera	Rheinheimera tangshanensis	SAMN12559023	anthropogenic	11	Yes	4	Zn_ribbon_6;RVT_1;STM3845;GD	—
2026887.SAMN11533002.WPIF010000	**typeII-A3**	300,6	high	Nitrospirota	—	—	—	—	Nitrospirota bacterium	SAMN11533002	terrestrial	7	Yes	3	HTH_3;HigB-like_toxin;STM3845	—
1879010.SAMEA7848008.CAJLNZ010	**typeII-A3**	297,7	high	Bacillota	—	—	—	—	Bacillota bacterium	SAMEA7848008	host-associated	11	Yes	4	DUF6594;STM3845;Transposase_	—
297314.SAMEA8805156.CAJTUB0100	**typeII-A3**	293,8	high	Bacillota	Clostridia	Lachnospirales	Lachnospiraceae	—	uncultured Lachnospiraceae bacterium	SAMEA8805156	host-associated	11	Yes	4	GyrI-like;STM3845;DUF2229;LysM	—
1286170.SAMN02603584.CP004142_2	**typeII-A3**	293,1	high	Pseudomonadota	Gammaproteobacteria	Enterobacterales	Enterobacteriaceae	Raoultella	Raoultella ornithinolytica	SAMN02603584	host-associated	11	Yes	4	Resolvase;STM3845;Adeno_E3_Cl	—
297314.SAMEA6151505.CADAKA0100	**typeII-A3**	290,9	high	Bacillota	Clostridia	Lachnospirales	Lachnospiraceae	—	uncultured Lachnospiraceae bacterium	SAMEA6151505	host-associated	8	Yes	4	BD-FAE;ABC_tran;STM3845;DUF	—
1898203.SAMN16342483.JAFQOW010	**typeII-A3**	288,2	high	Bacillota	Clostridia	Lachnospirales	Lachnospiraceae	—	Lachnospiraceae bacterium	SAMN16342483	host-associated	11	Yes	4	Acetyltransf_3;Acetyltransf_1;STM3	—
1129257.SAMEA6955832.CAIYPA0100	**typeII-A3**	286,9	high	Bacteroidota	Bacteroidia	—	—	—	uncultured Bacteroidia bacterium	SAMEA6955832	temperature	11	Yes	4	PIN_3;STM3845;CBP_BcsR	—
59620.SAMEA8805740.CAJUMP01000	**typeII-A3**	286,6	high	Bacillota	Clostridia	Eubacteriales	Clostridiaceae	Clostridium	uncultured Clostridium sp.	SAMEA8805740	—	7	Yes	0	—	—
59620.SAMEA8805516.CAJUHD01000	**typeII-A3**	286,3	high	Bacillota	Clostridia	Eubacteriales	Clostridiaceae	Clostridium	uncultured Clostridium sp.	SAMEA8805516	host-associated	11	Yes	4	DUF8318;Asn_synthase;STM3845	—
2800330.SAMN17189439.JAENGP010	**typeII-A3**	285,7	high	Pseudomonadota	Betaproteobacteria	Burkholderiales	Alcaligenaceae	Advenella	Advenella mandrilli	SAMN17189439	anthropogenic	11	Yes	4	GmrSD_N;STM3845;Type_ISP_C;	—
2024844.SAMN14914534.JABHWA010	**typeII-A3**	283,9	high	Nitrospinota	Nitrospinia	Nitrospinales	Nitrospinaceae	Nitrospina	Nitrospina sp.	SAMN14914534	aquatic	11	Yes	4	FlgM;Peptidase_M28;STM3845	—
1926307.SAMN18120172.JAGOOQ010	**typeII-A3**	283,4	high	Bacillota	Negativicutes	Veillonellales	Veillonellaceae	Veillonella	Veillonella sp.	SAMN18120172	anthropogenic	7	Yes	3	SLFN-g3_helicase;MazG-like;STM3	—
2026760.SAMN15049710.JACDNW010	**typeII-A3**	281,5	high	Fidelibacterota	—	—	—	—	Fidelibacterota bacterium	SAMN15049710	aquatic	11	Yes	4	STM3845;Tospov_NS-S_N	—
1849603.SAMEA5278299.CAAFVQ010	**typeII-A3**	281,1	high	Pseudomonadota	Gammaproteobacteria	Enterobacterales	Enterobacteriaceae	—	Enterobacteriaceae bacterium	SAMEA5278299	host-associated	11	Yes	4	Phage_int_M;Phage_integrase;STM	—
1797955.SAMN04314391.MGVA010000	**typeII-A3**	281	high	Elusimicrobiota	—	—	—	—	Elusimicrobia bacterium RIFOXYA12_	SAMN04314391	terrestrial	11	Yes	4	AAA_15;STM3845;Mal_decarbox_	—
2126740.SAMN08731216.PYLO010000	**typeII-A3**	280,2	high	Bacillota	Clostridia	Eubacteriales	Clostridiaceae	Clostridium	Clostridium fessum	SAMN08731216	host-associated	11	Yes	4	OST3_OST6;STM3845;KAP_NTPa	—
1898203.SAMEA7848093.CAJKQB010	**typeII-A3**	277,7	high	Bacillota	Clostridia	Lachnospirales	Lachnospiraceae	—	Lachnospiraceae bacterium	SAMEA7848093	host-associated	11	Yes	4	HicB_lk_antitox;HicA_toxin;STM38	—
1121132.SAMN02745247.FRDH010000	**typeII-A3**	277,3	high	Bacillota	Clostridia	Lachnospirales	Lachnospiraceae	Butyrivibrio	Butyrivibrio hungatei	SAMN02745247	host-associated	11	Yes	4	FRG;STM3845;ATLF;Glycos_trans	—
1898104.SAMN14914016.JABHCC010	**typeII-A3**	276,7	high	Bacteroidota	—	—	—	—	Bacteroidota bacterium	SAMN14914016	aquatic	11	Yes	4	HI_1054_N;STM3845;AAA_15;Pha	—
2013811.SAMN06767730.PGYR010000	**typeII-A3**	271,7	high	Ignavibacteriota	—	—	—	—	Ignavibacteriae bacterium HGW-Ignavi	SAMN06767730	anthropogenic	10	Yes	4	DUF3788;STM3845	—
1898104.SAMN19298609.JAHJUE0100	**typeII-A3**	264,6	high	Bacteroidota	—	—	—	—	Bacteroidota bacterium	SAMN19298609	terrestrial	11	Yes	4	Mrr_cat;STM3845;LiaF-TM;Adaptin	—
1953111.SAMN06455312.DLMI0100001	**typeII-A3**	264	high	Acidobacteriota	—	—	—	—	Acidobacteria bacterium UBA7540	SAMN06455312	salinity	7	Yes	3	NMT1;STM3845;Y1_Tnp	—
1274384.SAMD00244887.BMDT010000	**typeII-A3**	250	high	Bacillota	Bacilli	Lactobacillales	Enterococcaceae	Enterococcus	Enterococcus alcedinis	SAMD00244887	host-associated	11	Yes	4	AAA_15;STM3845;PH_alsin;SIR2_	—
221953.SAMEA5851657.CABLZX0100	**typeII-A3**	230,5	low	Spirochaetota	Spirochaetia	Brachyspirales	Brachyspiraceae	Brachyspira	uncultured Brachyspira sp.	SAMEA5851657	host-associated	10	Yes	4	Radical_SAM;SF-assemblin;STM38	—
1907659.SAMEA4555082.LT635469_63	**typeIII-A1**	308,4	high	Bacillota	Clostridia	Lachnospirales	Lachnospiraceae	Blautia	Blautia sp. Marseille-P3201T	SAMEA4555082	host-associated	11	Yes	4	PRTase-CE;DUF7761;DDE_Tnp_I	—
1953166.SAMN06454853.DITN0100007	**typeIII-A1**	307,4	high	Bacteroidota	—	—	—	—	Bacteroidetes bacterium UBA6183	SAMN06454853	aquatic	11	Yes	4	YjcN;HTH_45;PRTase-CE	—
1946545.SAMN06453218.DFXA010000	**typeIII-A1**	306,4	high	Bacteroidota	Flavobacteriia	Flavobacteriales	Flavobacteriaceae	Flavobacterium	Flavobacterium sp. UBA4120	SAMN06453218	terrestrial	11	Yes	4	PLDc_2;AhpC-TSA;DnaJ_C	—
2293108.SAMN09734653.QTVG010000	**typeIII-A1**	304,8	high	Bacillota	Clostridia	Eubacteriales	Eubacteriaceae	Eubacterium	Eubacterium sp. AF36-5BH	SAMN09734653	host-associated	11	Yes	4	TMEM62_C;DIP2311-like_C;DUF7	—
152509.SAMEA6951052.CAIMBK0100	**typeIII-A1**	303,5	high	Bacteroidota	—	—	—	—	uncultured Bacteroidota bacterium	SAMEA6951052	aquatic	8	Yes	4	Ribosomal_L35p;Ribosomal_L20;H	—
239.SAMN18120117.JAGOJA01000001	**typeIII-A1**	300,8	high	Bacteroidota	Flavobacteriia	Flavobacteriales	Flavobacteriaceae	Flavobacterium	Flavobacterium sp.	SAMN18120117	anthropogenic	11	Yes	4	Peptidase_M50;PLDc_2;YceI;DnaJ	—
1898203.SAMEA8801244.CAJSZT010	**typeIII-A1**	300,5	high	Bacillota	Clostridia	Lachnospirales	Lachnospiraceae	—	Lachnospiraceae bacterium	SAMEA8801244	host-associated	11	Yes	4	HTH_3;PNP_UDP_1;DUF7761;PR	—
2763671.SAMN15805278.JACRSZ010	**typeIII-A1**	298,1	high	Bacillota	Clostridia	Lachnospirales	Lachnospiraceae	Jingyaoa	Jingyaoa shaoxingensis	SAMN15805278	host-associated	10	Yes	4	PRTase-CE;DUF7761;LytR_cpsA_	—
190304.SAMN02603417.AE009951_72	**typeIII-A1**	298,1	high	Fusobacteriota	Fusobacteriia	Fusobacteriales	Fusobacteriaceae	Fusobacterium	Fusobacterium nucleatum	SAMN02603417	host-associated	11	Yes	4	PRTase-CE;DUF7761;MatE;S-met	—
213322.SAMEA9694813.CAJXTY0100	**typeIII-A1**	297,8	high	Bacteroidota	Flavobacteriia	Flavobacteriales	—	—	uncultured Flavobacteriales bacterium	SAMEA9694813	temperature	11	Yes	4	NLPC_P60;Thiolase_N;HTH_45;P	—
297314.SAMEA8805636.CAJUKA0100	**typeIII-A1**	296,6	high	Bacillota	Clostridia	Lachnospirales	Lachnospiraceae	—	uncultured Lachnospiraceae bacterium	SAMEA8805636	host-associated	6	Yes	2	DUF3173;Phage_int_SAM_3	—
1898204.SAMEA5849616.CABIZX0100	**typeIII-A1**	296,3	high	Bacillota	Clostridia	Eubacteriales	Clostridiaceae	—	Clostridiaceae bacterium	SAMEA5849616	host-associated	6	Yes	2	PRTase-CE;HTH_45	—
297314.SAMEA8805562.CAJUHN0100	**typeIII-A1**	296,3	high	Bacillota	Clostridia	Lachnospirales	Lachnospiraceae	—	uncultured Lachnospiraceae bacterium	SAMEA8805562	host-associated	4	Yes	0	—	—
2485925.SAMN16342043.JAFQBT010	**typeIII-A1**	296,1	high	Bacillota	Clostridia	Eubacteriales	Oscillospiraceae	—	Oscillospiraceae bacterium	SAMN16342043	host-associated	9	Yes	4	PRTase-CE;wHTH-PRTase_assc;	—
165185.SAMEA8805504.CAJUEE0100	**typeIII-A1**	294,7	high	Bacillota	Clostridia	Eubacteriales	Eubacteriaceae	Eubacterium	uncultured Eubacterium sp.	SAMEA8805504	host-associated	11	Yes	0	—	—
1977054.SAMN07619280.PAPI0100001	**typeIII-A1**	290,9	high	Bacteroidota	Flavobacteriia	Flavobacteriales	Flavobacteriaceae	Leeuwenhoekiella	Leeuwenhoekiella sp.	SAMN07619280	aquatic	9	Yes	4	AbiEii;DUF6922;HTH_45;PRTase-	—
1869212.SAMN16425552.JADJAG010	**typeIII-A1**	290,7	high	Bacteroidota	Chitinophagia	Chitinophagales	Chitinophagaceae	—	Chitinophagaceae bacterium	SAMN16425552	anthropogenic	11	Yes	4	DUF2188;HTH_45;PRTase-CE	—
192149.SAMN14492348.CP051244_14	**typeIII-A1**	289,6	high	Bacteroidota	Flavobacteriia	Flavobacteriales	Flavobacteriaceae	Allomuricauda	Allomuricauda sp.	SAMN14492348	aquatic	11	Yes	2	DUF2683	—
259315.SAMEA7847295.CAJMAQ0100	**typeIII-A1**	288,1	high	Bacillota	Clostridia	Eubacteriales	Oscillospiraceae	Faecalibacterium	uncultured Faecalibacterium sp.	SAMEA7847295	host-associated	7	Yes	3	Relaxase;PRTase-CE;DUF6061	—

558021.SAMN11512334.VFPD0100000	**typeIII-A1**	284,3	high	Bacteroidota	Flavobacteriia	Flavobacteriales	Weeksellaceae	Chryseobacterium	Chryseobacterium aquifrigidense	SAMN11512334	host-associated	11	Yes	4	TfoX_N;RlaP;DUF7225;PRTase-CE	—
152509.SAMEA6947882.CAIRES01000	**typeIII-A1**	284,3	high	Bacteroidota	—	—	—	—	uncultured Bacteroidota bacterium	SAMEA6947882	aquatic	11	Yes	4	Epimerase;RuvB_N;HTH_45;PRTa	—
370804.SAMEA6152641.CADCBZ0100	**typeIII-A1**	283,4	high	Bacteroidota	Bacteroidia	Bacteroidales	Prevotellaceae	—	uncultured Prevotellaceae bacterium	SAMEA6152641	host-associated	7	Yes	3	DUF4372;wHTH-PRTase_assc;PR	—
2766537.SAMN15783688.CP060696_2	**typeIII-A1**	277	high	Bacillota	Clostridia	Eubacteriales	Oscillospiraceae	Caproicibacterium	Caproicibacterium amylolyticum	SAMN15783688	host-associated	11	Yes	4	PRTase-CE;NADH_dhqG_C;Beta-p	—
1045867.SAMN18262562.JAGGDU010	**typeIII-A1**	274,5	high	Cyanobacteriota	Cyanophyceae	Oscillatoriales	Microcoleaceae	Trichodesmium	Trichodesmium erythraeum	SAMN18262562	aquatic	4	Yes	3	PRTase-CE;DUF2683	—
2044939.SAMN16347727.JAFYNR010	**typeIII-A1**	273,1	high	Bacillota	Clostridia	—	—	—	Clostridia bacterium	SAMN16347727	host-associated	9	Yes	4	PRTase-CE;wHTH-PRTase_assc;F	—
1898204.SAMN13893815.DUPM01000	**typeIII-A1**	272,6	high	Bacillota	Clostridia	Eubacteriales	Clostridiaceae	—	Clostridiaceae bacterium	SAMN13893815	host-associated	6	Yes	3	FtsX;HTH_45;Metallophos	—
194843.SAMEA6148840.CACWMD010	**typeIII-A1**	272	high	Bacteroidota	Bacteroidia	Bacteroidales	—	—	uncultured Bacteroidales bacterium	SAMEA6148840	host-associated	11	Yes	4	23S_rRNA_IVP;DegT_DnrJ_EryC1	—
1898104.SAMN16426528.JADKJP0100	**typeIII-A1**	268,1	high	Bacteroidota	—	—	—	—	Bacteroidota bacterium	SAMN16426528	anthropogenic	11	Yes	4	Transposase_mut;HTH_45;PRTase	—
2292300.SAMN09736784.QULN01000	**typeIII-A1**	265,2	high	Bacillota	Clostridia	Eubacteriales	Butyricicoccaceae	Butyricicoccus	Butyricicoccus sp. OM04-18BH	SAMN09736784	host-associated	11	Yes	4	Glycos_transf_2;NTP_transferase;v	—
2301481.SAMEA8805439.CAJUDL010	**typeIII-A1**	263,5	high	Bacteroidota	Bacteroidia	Bacteroidales	Muribaculaceae	—	uncultured Muribaculaceae bacterium	SAMEA8805439	host-associated	11	Yes	4	Glycos_transf_1;HTH_45;PRTase-(	—
1898206.SAMN16342349.JAFQNN010	**typeIII-A1**	261,9	high	Spirochaetota	Spirochaetia	Spirochaetales	Spirochaetaceae	—	Spirochaetaceae bacterium	SAMN16342349	host-associated	9	Yes	4	IFN-gamma;GmrSD_N;HTH_45;PR	—
2838877.SAMN19314831.CP075682_2	**typeIII-A1**	261,5	high	Bacteroidota	Flavobacteriia	Flavobacteriales	Weeksellaceae	Chryseobacterium	Chryseobacterium sp. ZHDP1	SAMN19314831	anthropogenic	11	Yes	4	Septknot;DUF2911;HTH_45;PRTas	—
1912772.SAMN13567726.JADMLB010	**typeIII-A1**	261,3	high	Thermodesulfobacteriota	Desulfovibrionia	Desulfovibrionales	Desulfovibrionaceae	Halodesulfovibrio	Halodesulfovibrio sp.	SAMN13567726	host-associated	11	Yes	4	PRTase-CE;HTH_45;EIF3E_C	—
2056868.SAMN08111093.QEHP01000	**typeIII-A1**	260,3	high	Bacteroidota	Flavobacteriia	Flavobacteriales	Weeksellaceae	Chryseobacterium	Chryseobacterium sp. HMWF035	SAMN08111093	anthropogenic	11	Yes	0	—	—
331630.SAMEA6149455.CACXJP0100	**typeIII-A1**	244,9	high	Bacillota	Erysipelotrichia	Erysipelotrichales	Erysipelotrichaceae	—	uncultured Erysipelotrichaceae bacteri	SAMEA6149455	host-associated	11	Yes	4	YidD;Ribonucleas_3_3	—
2203212.SAMN09090847.QGNY01000	**typeIII-A1**	242,3	high	Bacteroidota	Sphingobacteriia	Sphingobacteriales	Sphingobacteriaceae	Pedobacter	Pedobacter paludis	SAMN09090847	host-associated	11	Yes	4	DUF4365;Aminotran_1_2	—
2053569.SAMN16344304.JAFTIA0100	**typeIII-A1**	241,2	high	Lentisphaerota	Lentisphaeria	—	—	—	Lentisphaeria bacterium	SAMN16344304	host-associated	11	Yes	4	Pput2613-deam;Phage_holin_3_6	—
172901.SAMN08775280.QEKH0100002	**typeIII-A1**	238,4	high	Lentisphaerota	Lentisphaeria	Victivallales	Victivallaceae	Victivallis	Victivallis vadensis	SAMN08775280	host-associated	9	Yes	4	SmpB;Phage_holin_3_3;FAD_oxido	—
2053569.SAMN16344634.JAFTUS0100	**typeIII-A1**	237	high	Lentisphaerota	Lentisphaeria	—	—	—	Lentisphaeria bacterium	SAMN16344634	host-associated	11	Yes	4	DUF1232;Pput2613-deam;Prometh	—
1673717.SAMEA3750678.LN868536_1	**typeIII-A1**	233,6	high	Bacillota	Clostridia	Eubacteriales	Acutalibacteraceae	Anaeromassilibacillus	Anaeromassilibacillus senegalensis	SAMEA3750678	host-associated	11	Yes	4	Aminoglyc_resit;Glyco_hydro_25;Gl	—
2053569.SAMEA5278474.CAAGES010	**typeIII-A1**	223,1	high	Lentisphaerota	Lentisphaeria	—	—	—	Lentisphaeria bacterium	SAMEA5278474	host-associated	11	Yes	4	DUF6273;SQHop_cyclase_C;Histo	—
1948697.SAMN06452111.DHMO01000	**typeIII-A1**	222,6	high	Lentisphaerota	Lentisphaeria	—	—	—	Lentisphaeria bacterium UBA4640	SAMN06452111	—	11	Yes	0	—	—
1965604.SAMN06473682.NFJK010000	**typeIII-A1**	219,9	high	Bacillota	Clostridia	Eubacteriales	Acutalibacteraceae	Anaeromassilibacillus	Anaeromassilibacillus sp. An250	SAMN06473682	host-associated	11	Yes	4	FG-GAP;GH97_N;Glyco_hydro_25	—
1120746.SAMEA2602734.CCFG01000	**typeIII-A1**	208,6	low	—	—	—	—	—	bacterium MS4	SAMEA2602734	host-associated	10	Yes	4	FGGY_N;UTRA;DeoRC	—
707003.SAMEA7848199.CAJMFC0100	**typeIII-A1**	203	low	Bacillota	Clostridia	Eubacteriales	Oscillospiraceae	—	uncultured Oscillospiraceae bacterium	SAMEA7848199	host-associated	2	Yes	2	Glyco_hydro_25;GH97_N	—
905011.SAMEA7202205.CAJFQP0100	**typeIII-A1**	197,7	low	Bacillota	Clostridia	Eubacteriales	Oscillospiraceae	Anaerotruncus	uncultured Anaerotruncus sp.	SAMEA7202205	host-associated	7	Yes	4	DUF3089;tRNA_U5-meth_tr;YgaP-l	—
77133.SAMEA6946128.CAIOCH01000	**typeIII-A1**	189,2	low	—	—	—	—	—	uncultured bacterium	SAMEA6946128	aquatic	1	Yes	1	—	—
286133.SAMEA7846561.CAJJSA01000	**typeIII-A1**	170	low	Pseudomonadota	Betaproteobacteria	Burkholderiales	Sutterellaceae	Sutterella	uncultured Sutterella sp.	SAMEA7846561	host-associated	10	Yes	4	Peptidase_M20;DUF2201_N	—
1932692.SAMN11294377.SUWG01000	**typeIII-A1**	169	low	Lentisphaerota	—	—	—	—	Lentisphaerota bacterium	SAMN11294377	host-associated	10	Yes	4	DUF6530;zf-C2H2_ZNF462_11;HT	—
153809.SAMEA6947789.CAIKRR0100	**typeIII-A1**	168,4	low	Pseudomonadota	—	—	—	—	uncultured Pseudomonadota bacterium	SAMEA6947789	aquatic	8	Yes	4	DUF6036;MarR;N6_N4_Mtase	—
194843.SAMEA6954344.CAIRHD0100	**typeIII-A2**	310,2	high	Bacteroidota	Bacteroidia	Bacteroidales	—	—	uncultured Bacteroidales bacterium	SAMEA6954344	aquatic	10	Yes	4	Fic;HemN_C;PRTase-CE	—
595494.SAMN02598508.CP001616_71	**typeIII-A2**	309,3	high	Pseudomonadota	Gammaproteobacteria	Aeromonadales	Aeromonadaceae	Tolumonas	Tolumonas auensis	SAMN02598508	anthropogenic	11	Yes	4	rve;HTH_Tnp_1;DNApolII_N;PRTa	—
1262921.PRJEB697.HF992628_8	**typeIII-A2**	308,9	high	Bacteroidota	Bacteroidia	Bacteroidales	Prevotellaceae	Prevotella	Prevotella sp. CAG:1185	PRJEB697	host-associated	11	Yes	4	DNA_methylase;Phage_RepB;Hem	—
1946060.SAMN06455422.DFTV010000	**typeIII-A2**	303,9	high	Pseudomonadota	Gammaproteobacteria	Chromatiales	Chromatiaceae	Arsukibacterium	Arsukibacterium sp. UBA4203	SAMN06455422	aquatic	8	Yes	4	HTH_Tnp_1;rve;HTH_17;Dynactin_	—
1950669.SAMN06457402.DITE010000	**typeIII-A2**	302	high	Bacteroidota	Bacteroidia	Bacteroidales	—	—	Bacteroidales bacterium UBA6192	SAMN06457402	anthropogenic	8	Yes	4	PRTase-CE;HemN_C;Zn_ribbon_1	—
983548.SAMN00713609.CP002528_28	**typeIII-A2**	300,5	high	Bacteroidota	Flavobacteriia	Flavobacteriales	Flavobacteriaceae	Dokdonia	Dokdonia sp. 4H-3-7-5	SAMN00713609	temperature	11	Yes	4	PRTase-CE;HemN_C;DUF4369;Hi	—
162156.SAMEA7848044.CAJJYT01000	**typeIII-A2**	297,7	high	Bacteroidota	Bacteroidia	Bacteroidales	Bacteroidaceae	Bacteroides	uncultured Bacteroides sp.	SAMEA7848044	host-associated	11	Yes	4	HemN_C;RteC;DUF1062	—
1137281.SAMN01940371.ANLA010000	**typeIII-A2**	292	high	Bacteroidota	Flavobacteriia	Flavobacteriales	Flavobacteriaceae	Xanthomarina	Xanthomarina gelatinilytica	SAMN01940371	aquatic	6	Yes	2	Mga;PRTase-CE	—
159272.SAMEA8030120.CAJOCR0100	**typeIII-A2**	291,5	high	Bacteroidota	Bacteroidia	Bacteroidales	Prevotellaceae	Prevotella	uncultured Prevotella sp.	SAMEA8030120	host-associated	1	No	0	—	—
2136182.SAMN08772519.CP035934_8	**typeIII-A2**	276,8	high	Pseudomonadota	Gammaproteobacteria	Moraxellales	Moraxellaceae	Acinetobacter	Acinetobacter cumulans	SAMN08772519	aquatic	11	Yes	4	PRTase-CE;tRNA-synt_1;DUF648	—
2136182.SAMN08772519.CP035936_6	**typeIII-A2**	276,8	high	Pseudomonadota	Gammaproteobacteria	Moraxellales	Moraxellaceae	Acinetobacter	Acinetobacter cumulans	SAMN08772519	aquatic	11	Yes	0	—	—
2040292.SAMEA104414344.OEPV010	**typeIII-A2**	276,6	high	Bacteroidota	Bacteroidia	Bacteroidales	Dysgonomonadaceae	Pseudodysgonomonas	Pseudodysgonomonas massiliensis	SAMEA104414344	host-associated	11	Yes	4	PRTase-CE;Staph_reg_Sar_Rot;DU	—
1953167.SAMN06455210.DISB010000	**typeIII-A2**	269,9	high	Bacteroidota	—	—	—	—	Bacteroidetes bacterium UBA6221	SAMN06455210	anthropogenic	11	Yes	4	PRTase-CE;HTH_45;Bac_globin	—
2601176.SAMN12492949.VPDZ010000	**typeIII-A2**	269,3	high	Pseudomonadota	Gammaproteobacteria	Moraxellales	Moraxellaceae	Acinetobacter	Acinetobacter sp. YH12251	SAMN12492949	anthropogenic	11	Yes	4	PRTase-CE;tRNA-synt_1;DUF648	—
2040292.SAMEA104414344.OEPV010	**typeIII-A2**	252,6	high	Bacteroidota	Bacteroidia	Bacteroidales	Dysgonomonadaceae	Pseudodysgonomonas	Pseudodysgonomonas massiliensis	SAMEA104414344	host-associated	11	Yes	4	ASD1_dom;DUF4011;Lyase_8	—
162156.SAMEA7848044.CAJJYT01000	**typeIII-A2**	252	high	Bacteroidota	Bacteroidia	Bacteroidales	Bacteroidaceae	Bacteroides	uncultured Bacteroides sp.	SAMEA7848044	host-associated	11	Yes	4	SusD_RagB;CarbopepD_reg_2;HT	—
162156.SAMEA5279210.CAAFNQ0100	**typeIII-A2**	251,2	high	Bacteroidota	Bacteroidia	Bacteroidales	Bacteroidaceae	Bacteroides	uncultured Bacteroides sp.	SAMEA5279210	host-associated	11	Yes	4	HATPase_c_4;DUF5686;DUF4011	—
742766.SAMN02463862.GL891979_45	**typeIII-A2**	250,9	high	Bacteroidota	Bacteroidia	Bacteroidales	Dysgonomonadaceae	Dysgonomonas	Dysgonomonas gade	SAMN02463862	anthropogenic	11	Yes	4	ABC_tran;DUF4011;Sigma70_r2;A	—
77133.SAMEA6951857.CAIQON01000	**typeIII-A2**	249,3	high	—	—	—	—	—	uncultured bacterium	SAMEA6951857	aquatic	6	Yes	2	NHase_alpha;HD	—
1073388.SAMN02463954.JH815524_3	**typeIII-A2**	246,9	high	Bacteroidota	Bacteroidia	Bacteroidales	Bacteroidaceae	Bacteroides	Bacteroides fragilis	SAMN02463954	host-associated	11	Yes	4	DUF5686;S1;Anti-Pycsar_Apyc1	—
226186.SAMN02604314.AE015928_44	**typeIII-A2**	244,1	high	Bacteroidota	Bacteroidia	Bacteroidales	Bacteroidaceae	Bacteroides	Bacteroides thetaiotaomicron	SAMN02604314	host-associated	11	Yes	4	HEF_HK;DUF5686;MTES_1575;D	—
742727.SAMN02463851.JH992944_94	**typeIII-A2**	242,4	high	Bacteroidota	Bacteroidia	Bacteroidales	Bacteroidaceae	Bacteroides	Bacteroides oleiciplenus	SAMN02463851	host-associated	11	Yes	0	—	—
1236514.SAMD00010222.BAKL010000	**typeIII-A2**	242,2	high	Bacteroidota	Bacteroidia	Bacteroidales	Bacteroidaceae	Bacteroides	Bacteroides stercorirosoris	SAMD00010222	host-associated	11	Yes	0	—	—
411901.SAMN00627055.AAVM020000	**typeIII-A2**	241,9	high	Bacteroidota	Bacteroidia	Bacteroidales	Bacteroidaceae	Bacteroides	Bacteroides caccae	SAMN00627055	host-associated	11	Yes	4	GSIII_N;DUF5686;DUF4011;S1	—
338188.SAMEA5849530.CABIXA01000	**typeIII-A2**	241,4	high	Bacteroidota	Bacteroidia	Bacteroidales	Bacteroidaceae	Bacteroides	Bacteroides finegoldii	SAMEA5849530	host-associated	11	Yes	4	S1;DUF4011;DUF5686;GSIII_N	—
162156.SAMEA7847312.CAJLKP0100	**typeIII-A2**	241	high	Bacteroidota	Bacteroidia	Bacteroidales	Bacteroidaceae	Bacteroides	uncultured Bacteroides sp.	SAMEA7847312	—	6	Yes	2	Carb_kinase;SecDF_P1_head	—
29523.SAMN19224740.DXOY0100016	**typeIII-A2**	240,7	high	Bacteroidota	Bacteroidia	Bacteroidales	Bacteroidaceae	Bacteroides	Bacteroides sp.	SAMN19224740	host-associated	5	Yes	4	PDDEXK_2;DUF5686;DUF4011;S	—
295405.SAMD00061068.AP006841_98	**typeIII-A2**	240,3	high	Bacteroidota	Bacteroidia	Bacteroidales	Bacteroidaceae	Bacteroides	Bacteroides fragilis	SAMD00061068	host-associated	11	Yes	4	GSIII_N;DUF5686;S1;Anti-Pycsar_	—
1796613.SAMN04621613.CP015401_6	**typeIII-A2**	240,2	high	Bacteroidota	Bacteroidia	Bacteroidales	Bacteroidaceae	Bacteroides	Bacteroides caecimuris	SAMN04621613	host-associated	11	Yes	0	—	—
471870.SAMN00000015.ABJL0200000	**typeIII-A2**	240	high	Bacteroidota	Bacteroidia	Bacteroidales	Bacteroidaceae	Bacteroides	Bacteroides intestinalis	SAMN00000015	host-associated	11	Yes	4	Pep_deformylase;SecDF_P1_head;	—
702447.SAMN00007489.ADKP010000	**typeIII-A2**	239,5	low	Bacteroidota	Bacteroidia	Bacteroidales	Bacteroidaceae	Bacteroides	Bacteroides xylanisolvens	SAMN00007489	host-associated	8	Yes	0	—	—
512312.SAMEA8805346.CAJUAD0100	**typeIII-A2**	239,4	low	Bacteroidota	Bacteroidia	Bacteroidales	Tannerellaceae	Parabacteroides	uncultured Parabacteroides sp.	SAMEA8805346	host-associated	2	Yes	0	—	—
997874.SAMN02463917.JH724085_33	**typeIII-A2**	239,1	low	Bacteroidota	Bacteroidia	Bacteroidales	Bacteroidaceae	Bacteroides	Bacteroides cellulosilyticus	SAMN02463917	host-associated	10	Yes	0	—	—
2723058.SAMN14490823.JAAXGE010	**typeIII-A2**	238,3	low	Bacteroidota	Bacteroidia	Bacteroidales	Bacteroidaceae	Bacteroides	Bacteroides sp. GM023	SAMN14490823	host-associated	10	Yes	4	Selenoprotein_S;DUF5686;DUF737	—
1871006.SAMN15533020.JADMZD010	**typeIII-A2**	238,1	low	Bacteroidota	Bacteroidia	Bacteroidales	Bacteroidaceae	Bacteroides	Bacteroides congonensis	SAMN15533020	host-associated	10	Yes	4	LRR_5;DUF5686;DUF4011;S1	—
85831.SAMEA6831314.CAEUHO01000	**typeIII-A2**	237,2	low	Bacteroidota	Bacteroidia	Bacteroidales	Bacteroidaceae	Bacteroides	Bacteroides acidifaciens	SAMEA6831314	host-associated	10	Yes	4	S1;DUF4011;DUF5686;AAA-ATPa	—
712711.SAMN05761448.MIQB0100001	**typeIII-A2**	217,7	low	Bacteroidota	Bacteroidia	Bacteroidales	Tannerellaceae	Tannerella	Tannerella sp. oral taxon 808	SAMN05761448	host-associated	5	Yes	2	NAD_Gly3P_dh_C;tRNA-synt_2	—
1798573.SAMN04313869.MHBH01000	**typeIII-A2**	192,6	low	Lentisphaerota	—	—	—	—	Lentisphaerae bacterium GWF2_49_2	SAMN04313869	terrestrial	10	Yes	4	Prok-E2_D;SBP_bac_8;N_methyl	—
1506601.SAMEA6956220.CAIMFA010	**typeIII-A2**	189,2	low	Lentisphaerota	Lentisphaeria	—	—	—	uncultured Lentisphaeria bacterium	SAMEA6956220	aquatic	10	Yes	0	—	—
1506601.SAMEA6956826.CAIPAT0100	**typeIII-A2**	187,3	low	Lentisphaerota	Lentisphaeria	—	—	—	uncultured Lentisphaeria bacterium	SAMEA6956826	aquatic	4	Yes	4	DUF6908;DUF932	—
195913.SAMN04488004.FOTF0100001	**typeIII-A2**	175,4	low	Pseudomonadota	Alphaproteobacteria	Rhodobacterales	Roseobacteraceae	Loktanella	Loktanella salsilacus	SAMN04488004	salinity	5	Yes	2	STM3845;Toprim	—
2024855.SAMN13893731.JAAYEH010	**typeIII-A2**	170,4	low	Planctomycetota	Planctomycetia	Pirellulales	Pirellulaceae	Rhodopirellula	Rhodopirellula sp.	SAMN13893731	temperature	8	Yes	4	AAA_19;BD-FAE;Band_7;AP_endo	—
1192759.SAMN02470887.AKIB010000	**typeIII-A3**	320,2	high	Pseudomonadota	Alphaproteobacteria	Sphingomonadales	Sphingobiaceae	Sphingobium	Sphingobium xenophagum	SAMN02470887	terrestrial	7	Yes	3	PRTase-CE;SRAP;DUF7547	—
546367.SAMN08019857.DPJS0100004	**typeIII-A3**	319,6	high	Pseudomonadota	Gammaproteobacteria	Enterobacterales	Hafniaceae	Hafnia	Hafnia paralvei	SAMN08019857	anthropogenic	11	Yes	4	HTH_18;Ni_hydr_CYTB;PRTase-C	—
1219052.SAMD00046752.BCYZ010000	**typeIII-A3**	318,1	high	Pseudomonadota	Alphaproteobacteria	Sphingomonadales	Sphingomonadaceae	Sphingomonas	Sphingomonas pruni	SAMD00046752	host-associated	8	Yes	4	MobA_MobL;DUF6118;PRTase-CE	—
1946868.SAMN06455384.DCRO01000	**typeIII-A3**	314,7	high	Pseudomonadota	Gammaproteobacteria	Thiotrichales	Piscirickettsiaceae	Methylophaga	Methylophaga sp. UBA1490	SAMN06455384	aquatic	11	Yes	4	MOSC;PFF1_TM;Host_attach	—
1125979.SAMN02470738.AHZC010001	**typeIII-A3**	314,6	high	Pseudomonadota	Alphaproteobacteria	Hyphomicrobiales	Rhizobiaceae	Rhizobium	Rhizobium sp. PDO1-076	SAMN02470738	host-associated	3	Yes	2	Gamma_PGA_hydro;PRTase-CE	—
2163011.SAMN08912484.CP028901_2	**typeIII-A3**	310,8	high	Pseudomonadota	Betaproteobacteria	Burkholderiales	Alcaligenaceae	Orrella	Orrella marina	SAMN08912484	anthropogenic	11	Yes	4	Metallophos;PRTase-CE	—

1909294.SAMEA4707468.UBQB01000(	**typeIII-A3**	309,5	**high**	Pseudomonadota	Alphaproteobacteria	Hyphomicrobiales	—	—	Hyphomicrobiales bacterium	SAMEA4707468	host-associated	11	Yes	4	PRTase-CE;Penicillinase_R	—
1909294.SAMEA4707564.UBOO01000(	**typeIII-A3**	309,5	**high**	Pseudomonadota	Alphaproteobacteria	Hyphomicrobiales	—	—	Hyphomicrobiales bacterium	SAMEA4707564	—	11	Yes	0	—	—
2723087.SAMN14515540.JACHCP010(	**typeIII-A3**	308	**high**	Pseudomonadota	Gammaproteobacteria	Lysobacterales	Rhodanobacteraceae	Rhodanobacter	Rhodanobacter sp. A1T4	SAMN14515540	terrestrial	11	Yes	4	MM_CoA_mutase;PRTase-CE;RE_	—
216595.SAMEA2272316.AM181176_18	**typeIII-A3**	306,6	**high**	Pseudomonadota	Gammaproteobacteria	Pseudomonadales	Pseudomonadaceae	Pseudomonas	Pseudomonas fluorescens	SAMEA2272316	host-associated	11	Yes	4	TnpB_IS66;DDE_Tnp_IS66;PRTas	—
400153.SAMN12559023.VRLR0100002	**typeIII-A3**	305,8	**high**	Pseudomonadota	Gammaproteobacteria	Chromatiales	Chromatiaceae	Rheinheimera	Rheinheimera tangshanensis	SAMN12559023	anthropogenic	6	Yes	2	PRTase-CE;UvrD-helicase	—
1195246.SAMN02470206.AKKU010000	**typeIII-A3**	304,8	**high**	Pseudomonadota	Gammaproteobacteria	Alteromonadales	Alteromonadaceae	Alishewanella	Alishewanella agri	SAMN02470206	—	11	Yes	0	—	—
1417228.SAMN04498477.CP014579_7	**typeIII-A3**	304,7	**high**	Pseudomonadota	Betaproteobacteria	Burkholderiales	Burkholderiaceae	Paraburkholderia	Paraburkholderia phytofirmans	SAMN04498477	temperature	11	Yes	4	PRTase-CE;Glyoxalase_8;Metalloth	—
49181.SAMN16425591.JADJBR010000	**typeIII-A3**	303,4	**high**	Pseudomonadota	Betaproteobacteria	Rhodocyclales	Zoogloeaceae	Zoogloea	Zoogloea sp.	SAMN16425591	anthropogenic	11	Yes	4	HTH_3;NERD;PRTase-CE;Virulenc	—
1330531.SAMN02472181.KE166411_9	**typeIII-A3**	300,1	**high**	Pseudomonadota	Gammaproteobacteria	Pseudomonadales	Pseudomonadaceae	Pseudomonas	Pseudomonas plecoglossicida	SAMN02472181	host-associated	6	Yes	2	PRTase-CE	—
1400867.SAMN02641530.CP006768_2	**typeIII-A3**	299,7	**high**	Pseudomonadota	Gammaproteobacteria	Moraxellales	Moraxellaceae	Acinetobacter	Acinetobacter baumannii	SAMN02641530	anthropogenic	11	Yes	4	PRTase-CE;DUF6741;4HBT;Hexap	—
1946058.SAMN06451192.DEYP010000	**typeIII-A3**	298,8	**high**	Pseudomonadota	Gammaproteobacteria	Chromatiales	Chromatiaceae	Arsukibacterium	Arsukibacterium sp. UBA3155	SAMN06451192	aquatic	11	Yes	4	Phage_AlpA;PRTase-CE;WHD_Br	—
314271.SAMN12291826.VNHV0100001	**typeIII-A3**	298,2	**high**	Pseudomonadota	Alphaproteobacteria	Rhodobacterales	Roseobacteraceae	Maritimibacter	Maritimibacter alkaliphilus	SAMN12291826	aquatic	11	Yes	4	PRiA4_ORF3;PRTase-CE;AbiJ_NT	—
373675.SAMN04488045.FNUZ0100000	**typeIII-A3**	298,2	**high**	Pseudomonadota	Alphaproteobacteria	Rhodobacterales	Roseobacteraceae	Thalassococcus	Thalassococcus halodurans	SAMN04488045	aquatic	9	Yes	0	—	—
1848702.SAMN07581400.PCOM01000(	**typeIII-A3**	296	**high**	Pseudomonadota	Alphaproteobacteria	Hyphomicrobiales	Brucellaceae	Ochrobactrum	Ochrobactrum sp. MYb19	SAMN07581400	host-associated	7	Yes	3	YobI-ATPase;PRTase-CE	—
304208.SAMD00018367.DF820596_6	**typeIII-A3**	294,5	**high**	Pseudomonadota	Gammaproteobacteria	Alteromonadales	Pseudoalteromonadaceae	Pseudoalteromonas	Pseudoalteromonas sp. '520P1 No. 41	SAMD00018367	aquatic	11	Yes	4	DUF7662;PRTase-CE;YrhC;tSH3-E	—
1122616.SAMN02441143.KE383828_2	**typeIII-A3**	294,4	**high**	Pseudomonadota	Gammaproteobacteria	Oceanospirillales	Oceanospirillaceae	Oceanospirillum	Oceanospirillum beijerinckii	SAMN02441143	aquatic	11	Yes	4	HTH_3;PRTase-CE	—
314276.SAMN02436083.CH672406_16	**typeIII-A3**	293,4	**high**	Pseudomonadota	Gammaproteobacteria	Alteromonadales	Idiomarinaceae	Idiomarina	Idiomarina baltica	SAMN02436083	aquatic	11	Yes	4	Acetyltransf_7;PRTase-CE;Phage_	—
409.SAMN20165186.JAIETD01000002	**typeIII-A3**	293	**high**	Pseudomonadota	Alphaproteobacteria	Hyphomicrobiales	Methylobacteriaceae	Methylobacterium	Methylobacterium sp.	SAMN20165186	aquatic	6	Yes	4	Phage_tube_2;PRTase-CE;Phage_	—
2502232.SAMN08712299.SCOE01000(	**typeIII-A3**	288,9	**high**	Pseudomonadota	Betaproteobacteria	Burkholderiales	Comamonadaceae	—	Comamonadaceae bacterium ALPHA2	SAMN08712299	anthropogenic	11	Yes	4	Astacin;Peptidase_C14;PRTase-CE	—
2502219.SAMN08712286.SCNR010000	**typeIII-A3**	284,7	**high**	Pseudomonadota	Alphaproteobacteria	Caulobacterales	Caulobacteraceae	Caulobacter	Caulobacter sp. 3R27C2-B	SAMN08712286	aquatic	9	Yes	4	DUF6088;AbiEii;PRTase-CE;DUF3	—
2597224.SAMN10849879.SHMQ01000(	**typeIII-A3**	283,4	**high**	—	Deltaproteobacteria	Candidatus Acidulodesulf	—	Candidatus Acidulodes	Candidatus Acidulodesulfobacterium a	SAMN10849879	anthropogenic	11	Yes	4	BAR_4;Phage_integrase;PRTase-C	—
68895.SAMD00023459.BBQM0100000	**typeIII-A3**	283,1	**high**	Pseudomonadota	Betaproteobacteria	Burkholderiales	Burkholderiaceae	Cupriavidus	Cupriavidus basilensis	SAMD00023459	terrestrial	11	Yes	4	AimR;PRTase-CE;DUF4411	—
2033014.SAMN09639989.DTKD010000	**typeIII-A3**	281,5	**high**	Armatimonadota	—	—	—	—	Armatimonadota bacterium	SAMN09639989	aquatic	11	Yes	4	HTH_3;PRTase-CE;Phage_portal	—
1670.SAMN12638718.VTFV01000006_	**typeIII-A3**	278,5	**high**	Actinomycetota	Actinomycetes	Micrococcales	Micrococcaceae	Arthrobacter	Arthrobacter citreus	SAMN12638718	host-associated	11	Yes	4	AIPR;Cecropin;PRTase-CE;DUF33	—
57320.SAMEA104288281.LT907975_1(	**typeIII-A3**	277,4	**high**	Thermodesulfobacteriota	Desulfovibrionia	Desulfovibrionales	Desulfovibrionaceae	Pseudodesulfovibrio	Pseudodesulfovibrio profundus	SAMEA104288281	aquatic	11	Yes	4	HTH_Tnp_1;PRTase-CE;HTH_3;N	—
2052182.SAMN16426408.JADKFD010(	**typeIII-A3**	275,8	**high**	Pseudomonadota	Gammaproteobacteria	Lysobacterales	Rhodanobacteraceae	—	Rhodanobacteraceae bacterium	SAMN16426408	anthropogenic	11	Yes	4	dnstrm_HI1420;SLFN-g3_helicase;	—
1552759.SAMN12660232.CP043474_2	**typeIII-A3**	270,6	**high**	Actinomycetota	Actinomycetes	Mycobacteriales	Mycobacteriaceae	Mycolicibacterium	Mycolicibacterium grossiae	SAMN12660232	anthropogenic	11	Yes	4	HTH_5;Hydrolase;PRTase-CE;Gmr	—
2740298.SAMN15042369.CP054861_9	**typeIII-A3**	270,2	**high**	Pseudomonadota	Alphaproteobacteria	Hyphomicrobiales	Aurantimonadaceae	Martelella	Martelella soudanensis	SAMN15042369	aquatic	11	Yes	4	DDE_Tnp_1;DUF4096;RovC_DNA	—
2562705.SAMEA6944266.CAIQXC010(	**typeIII-A3**	267,9	**high**	Verrucomicrobiota	Verrucomicrobiia	Verrucomicrobiales	Akkermansiaceae	—	Akkermansiaceae bacterium	SAMEA6944266	aquatic	5	Yes	4	Methyltr_RsmB-F;PRTase-CE;SWI	—
184870.SAMN08193735.PKHX0100001	**typeIII-A3**	237,8	**high**	Actinomycetota	Actinomycetes	Actinomycetales	Actinomycetaceae	Varibaculum	Varibaculum cambriense	SAMN08193735	host-associated	11	Yes	4	ABC2_membrane_3;N6_Mtase;Met	—
1852372.SAMEA4521270.FNWI010000	**typeIII-A3**	236,6	**high**	Actinomycetota	Actinomycetes	Actinomycetales	Actinomycetaceae	Varibaculum	Varibaculum massiliense	SAMEA4521270	—	11	Yes	0	—	—
563192.SAMN02463824.KE150238_24	**typeIII-A3**	233,7	**high**	Thermodesulfobacteriota	Desulfovibrionia	Desulfovibrionales	Desulfovibrionaceae	Bilophila	Bilophila wadsworthia	SAMN02463824	host-associated	11	Yes	4	Methylase_S;PRTase-CE;Fic;Cupir	—
1981031.SAMEA7202140.CAJFUZ010(	**typeIII-A3**	233,4	**high**	Thermodesulfobacteriota	Desulfovibrionia	Desulfovibrionales	Desulfovibrionaceae	Mailhella	uncultured Mailhella sp.	SAMEA7202140	—	11	Yes	0	—	—
158897.SAMD00245121.BMKV0100000	**typeIII-A3**	229,5	**high**	Actinomycetota	Actinomycetes	Micrococcales	Micrococcaceae	Pseudarthrobacter	Pseudarthrobacter scleromae	SAMD00245121	anthropogenic	11	Yes	4	DNA_methylase;Helicase_C	—
1121890.SAMN02440631.AUDO01000(	**typeIII-A4**	348,7	**high**	Bacteroidota	Flavobacteriia	Flavobacteriales	Flavobacteriaceae	Flavobacterium	Flavobacterium frigidarium	SAMN02440631	salinity	9	Yes	4	DUF4172;Lipase_3;PRTase-CE;Ec	—
651561.SAMN06453202.DCFI0100000	**typeIII-A4**	345,8	**high**	Bacteroidota	Flavobacteriia	Flavobacteriales	Weeksellaceae	Chryseobacterium	Chryseobacterium arthrosphaerae	SAMN06453202	host-associated	11	Yes	4	TraG_N;GtfB_M;PRTase-CE;Acety	—
194843.SAMEA6945835.CAJAXK0100(	**typeIII-A4**	327,3	**high**	Bacteroidota	Bacteroidia	Bacteroidales	—	—	uncultured Bacteroidales bacterium	SAMEA6945835	aquatic	11	Yes	4	Ntox30;PRTase-CE;N6_Mtase;Met	—
1932669.SAMEA6565344.LR824569_2	**typeIII-A4**	327,3	**high**	Bacteroidota	Flavobacteriia	Flavobacteriales	Weeksellaceae	Chryseobacterium	Chryseobacterium sp. JV274	SAMEA6565344	host-associated	11	Yes	4	PRTase-CE;HTH_17	—
1871037.SAMN10967439.DTTW01000(	**typeIII-A4**	324,8	**high**	Bacteroidota	Flavobacteriia	Flavobacteriales	Flavobacteriaceae	—	Flavobacteriaceae bacterium	SAMN10967439	aquatic	11	Yes	4	HTH_17;WHD_lin-66;PRTase-CE;	—
2212467.SAMN11294424.SUYB01000(	**typeIII-A4**	318,6	**high**	Bacteroidota	Bacteroidia	Bacteroidales	Bacteroidaceae	—	Bacteroidaceae bacterium	SAMN11294424	host-associated	11	Yes	4	PRTase-CE;NttA;PSI_induc_2	—
194843.SAMEA6150768.CACZHT0100(	**typeIII-A4**	316,6	**high**	Bacteroidota	Bacteroidia	Bacteroidales	—	—	uncultured Bacteroidales bacterium	SAMEA6150768	host-associated	11	Yes	0	—	—
194843.SAMEA6150337.CACYRK0100	**typeIII-A4**	315,2	**high**	Bacteroidota	Bacteroidia	Bacteroidales	—	—	uncultured Bacteroidales bacterium	SAMEA6150337	host-associated	7	Yes	0	—	—
1965624.SAMN06473711.NFIV0100001	**typeIII-A4**	314,5	**high**	Bacteroidota	Bacteroidia	Bacteroidales	Muribaculaceae	Muribaculum	Muribaculum sp. An289	SAMN06473711	host-associated	7	Yes	3	Transposase_mut;PRTase-CE;Arm	—
2683263.SAMN13494107.JAAALB0100	**typeIII-A4**	307,5	**high**	Bacteroidota	Flavobacteriia	Flavobacteriales	Flavobacteriaceae	Cellulophaga	Cellulophaga sp. BC115SP	SAMN13494107	aquatic	9	Yes	4	SH3_3;PRTase-CE;S1;DUF7660	—
2249356.SAMN09487226.CP030261_2	**typeIII-A4**	305,5	**high**	Bacteroidota	Flavobacteriia	Flavobacteriales	Flavobacteriaceae	Flavobacterium	Flavobacterium fluviale	SAMN09487226	terrestrial	11	Yes	4	MerR_1;Abhydrolase_1;PRTase-CE	—
2748031.SAMN16350081.JAGBZD010(	**typeIII-A4**	304,7	**high**	Bacteroidota	Bacteroidia	Bacteroidales	Paludibacteraceae	—	Paludibacteraceae bacterium	SAMN16350081	host-associated	7	Yes	3	HTH_17;PRTase-CE	—
1236517.SAMN03704035.CP012074_1	**typeIII-A4**	300,6	**high**	Bacteroidota	Bacteroidia	Bacteroidales	Prevotellaceae	Prevotella	Prevotella fusca	SAMN03704035	—	11	Yes	0	—	—
702438.SAMN02463847.JH114217_20	**typeIII-A4**	300,1	**high**	Bacteroidota	Bacteroidia	Bacteroidales	Prevotellaceae	Segatella	Segatella oulorum	SAMN02463847	host-associated	11	Yes	4	PRTase-CE;Fijivirus_P9-2;DUF270	—
2030927.SAMN18472452.JAGHRT010(	**typeIII-A4**	299,4	**high**	Bacteroidota	Bacteroidia	Bacteroidales	—	—	Bacteroidales bacterium	SAMN18472452	host-associated	11	Yes	4	Bac_transf;PRTase-CE;Hypoth_Ym	—
370804.SAMEA6151246.CADAAG0100	**typeIII-A4**	298,1	**high**	Bacteroidota	Bacteroidia	Bacteroidales	Prevotellaceae	—	uncultured Prevotellaceae bacterium	SAMEA6151246	host-associated	5	Yes	0	—	—
370804.SAMEA6152641.CADCBZ0100	**typeIII-A4**	295,1	**high**	Bacteroidota	Bacteroidia	Bacteroidales	Prevotellaceae	—	uncultured Prevotellaceae bacterium	SAMEA6152641	host-associated	11	Yes	4	Med9;DEDD_Tnp_IS110;PRTase-(	—
2030927.SAMN14407244.JAAVFK0100	**typeIII-A4**	284,4	**high**	Bacteroidota	Bacteroidia	Bacteroidales	—	—	Bacteroidales bacterium	SAMN14407244	host-associated	8	Yes	4	AbiEii;Metallophos;PRTase-CE	—
194843.SAMEA6954344.CAIRHD0100(	**typeIII-A4**	282,2	**high**	Bacteroidota	Bacteroidia	Bacteroidales	—	—	uncultured Bacteroidales bacterium	SAMEA6954344	aquatic	11	Yes	4	PHB_acc;N6_Mtase;PRTase-CE;M	—
194843.SAMEA6149846.CACXYM0100	**typeIII-A4**	272,9	**borderline**	Bacteroidota	Bacteroidia	Bacteroidales	—	—	uncultured Bacteroidales bacterium	SAMEA6149846	host-associated	10	Yes	4	Pox_polyA_pol_N;KAP_NTPase;PF	—
194843.SAMEA6152515.CADBWX010(	**typeIII-A4**	272,4	**borderline**	Bacteroidota	Bacteroidia	Bacteroidales	—	—	uncultured Bacteroidales bacterium	SAMEA6152515	host-associated	7	Yes	4	PRTase-CE;KAP_NTPase	—
2293831.SAMN09736732.QUMM01000	**typeIII-A5**	298,8	**high**	Bacillota	Clostridia	Lachnospirales	Lachnospiraceae	—	Lachnospiraceae bacterium OF09-6	SAMN09736732	host-associated	7	Yes	3	PRTase-CE;HTH_45	—
1681184.SAMN04123205.KQ465162_8	**typeIII-A5**	294	**high**	Bacillota	Bacilli	Bacillales	Bacillaceae	Lysinibacillus	Lysinibacillus sp. ZYM-1	SAMN04123205	terrestrial	11	Yes	4	YhjD;PRTase-CE;IMS_C;YolD	—
1834198.SAMN04621621.CP015400_2	**typeIII-A5**	289,3	**high**	Bacillota	Clostridia	Eubacteriales	Oscillospiraceae	—	Hungateiclostridiaceae bacterium KB1	SAMN04621621	host-associated	11	Yes	4	DUF6537;GP57;DnaD_N;PRTase-	—
796937.SAMN00828788.ALNK0100002	**typeIII-A5**	288,9	**high**	Bacillota	Clostridia	Peptostreptococcales	Filifactoraceae	Peptoanaerobacter	Peptoanaerobacter stomatis	SAMN00828788	host-associated	11	Yes	4	PRTase-CE;wHTH-PRTase_assc;M	—
1898203.SAMEA5278693.CAAFYN010(	**typeIII-A5**	283,4	**high**	Bacillota	Clostridia	Lachnospirales	Lachnospiraceae	—	Lachnospiraceae bacterium	SAMEA5278693	host-associated	11	Yes	4	PRTase-CE;wHTH-PRTase_assc	—
1898207.SAMN19225207.DXWH01000	**typeIII-A5**	283	**high**	Bacillota	Clostridia	Eubacteriales	—	—	Clostridiales bacterium	SAMN19225207	host-associated	10	Yes	4	DUF6809;PRTase-CE	—
1280.SAMN05853508.CP017807_2376	**typeIII-A5**	282	**high**	Bacillota	Bacilli	Bacillales	Staphylococcaceae	Staphylococcus	Staphylococcus aureus	SAMN05853508	host-associated	11	Yes	4	TPP_enzyme_C;PRTase-CE;HNH	—
2028282.SAMN18359877.JAGHIX0100	**typeIII-A5**	281,3	**high**	Bacillota	Clostridia	Lachnospirales	Lachnospiraceae	Lachnoclostridium	Lachnoclostridium sp.	SAMN18359877	host-associated	4	Yes	2	wHTH-PRTase_assc;PRTase-CE	—
297314.SAMEA8805636.CAJUKA0100(	**typeIII-A5**	279,5	**high**	Bacillota	Clostridia	Lachnospirales	Lachnospiraceae	—	uncultured Lachnospiraceae bacterium	SAMEA8805636	host-associated	10	Yes	4	DUF5481;wHTH-PRTase_assc;PR	—
286138.SAMEA8805337.CAJUBG0100(	**typeIII-A5**	279,4	**high**	Bacillota	Clostridia	Lachnospirales	Lachnospiraceae	Dorea	uncultured Dorea sp.	SAMEA8805337	—	10	Yes	0	—	—
2044939.SAMN16345800.JAFVRR010(	**typeIII-A5**	275,3	**high**	Bacillota	Clostridia	—	—	—	Clostridia bacterium	SAMN16345800	host-associated	11	Yes	4	HNH;SLFN-g3_helicase;PRTase-C	—
1946771.SAMN06451782.DDLI0100001	**typeIII-A5**	274,8	**high**	Bacillota	Bacilli	Bacillales	Bacillaceae	Lysinibacillus	Lysinibacillus sp. UBA2576	SAMN06451782	anthropogenic	11	Yes	4	Flavin_Reduct;MerR_1;wHTH-PRT	—
1948087.SAMN06454066.DIWF010000	**typeIII-A5**	272,9	**high**	Bacillota	—	—	—	—	Firmicutes bacterium UBA6113	SAMN06454066	terrestrial	6	Yes	2	wHTH-PRTase_assc;PRTase-CE	—
1898207.SAMN19225191.DYCF010000	**typeIII-A5**	271,9	**high**	Bacillota	Clostridia	Eubacteriales	—	—	Clostridiales bacterium	SAMN19225191	host-associated	11	Yes	4	PNP_UDP_1;RVT_1;wHTH-PRTas	—
1950840.SAMN06452039.DDQW01000	**typeIII-A5**	271,8	**high**	Bacillota	Clostridia	Eubacteriales	—	—	Clostridiales bacterium UBA2432	SAMN06452039	terrestrial	8	Yes	4	CC4_CEP85;HTH_3;PRTase-CE;H	—
642492.SAMN00013959.CP002582_28	**typeIII-A5**	271,6	**high**	Bacillota	Clostridia	Lachnospirales	Cellulosilyticaceae	Cellulosilyticum	Cellulosilyticum lentocellum	SAMN00013959	host-associated	11	Yes	4	wHTH-PRTase_assc;PRTase-CE	—
1898207.SAMN14407303.JAAVHR010(	**typeIII-A5**	267,8	**high**	Bacillota	Clostridia	Eubacteriales	—	—	Clostridiales bacterium	SAMN14407303	host-associated	11	Yes	4	PRTase-CE;HTH_28;MCPsignal	—
1915574.SAMN06027062.DAKJ010000	**typeIII-A5**	267,8	**high**	Methanobacteriota	Methanomicrobia	Methanosarcinales	Methanosarcinaceae	Methanosarcina	Methanosarcina sp. UBA289	SAMN06027062	anthropogenic	11	Yes	4	YqaJ;Gly_zipper;RsgA_GTPase	—
1410670.SAMN02744016.JHXF010000	**typeIII-A5**	263,4	**high**	Bacillota	Clostridia	Eubacteriales	Oscillospiraceae	Ruminococcus	Ruminococcus flavefaciens	SAMN02744016	host-associated	8	Yes	4	HTH_Tnp_1;SH3_3;PRTase-CE	—
2250255.SAMEA8236582.CAJPFJ0100	**typeIII-A5**	261,2	**high**	Methanobacteriota	Methanomicrobia	Methanosarcinales	—	—	Methanosarcinales archaeon	SAMEA8236582	terrestrial	11	Yes	4	GIIM;tRNA-Thr_ED	—
297314.SAMEA8805271.CAJTZG0100(	**typeIII-A5**	261	**high**	Bacillota	Clostridia	Lachnospirales	Lachnospiraceae	—	uncultured Lachnospiraceae bacterium	SAMEA8805271	host-associated	11	Yes	4	NikA-like;HD_5;wHTH-PRTase_ass	—
1751881.SAMEA8805505.CAJUEJ010(	**typeIII-A5**	259,7	**high**	Bacillota	Clostridia	Lachnospirales	Lachnospiraceae	Hungatella	uncultured Hungatella sp.	SAMEA8805505	host-associated	11	Yes	4	His_kinase;HTH_34;PRTase-CE	—
2800128.SAMN17181733.CP067364_2	**typeIII-A5**	258,8	**high**	Bacillota	Bacilli	Lactobacillales	Carnobacteriaceae	Carnobacterium	Carnobacterium sp. CS13	SAMN17181733	host-associated	11	Yes	4	Band_7;PRTase-CE;Upx-like	—
2485925.SAMN11294765.SVLF010000	**typeIII-A5**	252	**high**	Bacillota	Clostridia	Eubacteriales	Oscillospiraceae	—	Oscillospiraceae bacterium	SAMN11294765	host-associated	10	Yes	4	SBP_bac_1;Agd3_C;PRTase-CE	—

297314.SAMEA8805790.CAJUQB0100(	**typeIII-A5**	251,6	high	Bacillota	Clostridia	Lachnospirales	Lachnospiraceae	—	uncultured Lachnospiraceae bacterium	SAMEA8805790	host-associated	10	Yes	4	Penicillinase_R;DUF4180;HTH_34;	—
2293093.SAMN09736788.QULM01000(	**typeIII-A5**	247,8	high	Bacillota	Clostridia	Lachnospirales	Lachnospiraceae	Coprococcus	Coprococcus sp. OM04-5BH	SAMN09736788	host-associated	11	Yes	4	Peptidase_S41;GMP_synt_C;PRTa	—
286138.SAMEA8805309.CAJUAM0100(	**typeIII-A5**	243,9	high	Bacillota	Clostridia	Lachnospirales	Lachnospiraceae	Dorea	uncultured Dorea sp.	SAMEA8805309	host-associated	11	Yes	4	HxlR;tRNA_edit;PRTase-CE;ROK	—
169435.SAMN15533212.JADNIE01000(	**typeIII-A5**	243,6	high	Bacillota	Clostridia	Eubacteriales	Oscillospiraceae	Anaerotruncus	Anaerotruncus colihominis	SAMN15533212	host-associated	9	Yes	4	PRTase-CE;HTH_AsnC-type;CstA;	—
1903720.SAMN18359853.JAGHHZ010(	**typeIII-A5**	242	high	Bacillota	Bacilli	—	—	—	Bacilli bacterium	SAMN18359853	host-associated	9	Yes	4	ZW10_C2;LysM;PRTase-CE;Resol	—
224209.SAMEA8804963.CAJTLN0100(	**typeIII-A5**	241,7	high	Bacillota	Bacilli	—	—	—	uncultured Bacilli bacterium	SAMEA8804963	host-associated	11	Yes	4	RE_LlaJI;PRTase-CE;Acetyltransf_	—
2026735.SAMN17179375.JAFDGY010(	**typeIII-A5**	241	high	Myxococcota	Myxococcia	—	—	—	Deltaproteobacteria bacterium	SAMN17179375	temperature	6	Yes	2	PNP_UDP_1;TPR_6	—
2049433.SAMN10607086.SKZT010000	**typeIII-A5**	236,4	high	Thermodesulfobacteriota	Desulfobacteria	Desulfobacterales	Desulfobacteraceae	—	Desulfobacteraceae bacterium	SAMN10607086	salinity	9	Yes	4	TPR_6;PNP_UDP_1;DUF983;DUF	—
2026735.SAMN18120041.JAGOOG010	**typeIII-A5**	233,9	low	Myxococcota	Myxococcia	—	—	—	Deltaproteobacteria bacterium	SAMN18120041	anthropogenic	10	Yes	4	DUF1848;N6_N4_Mtase;Peptidase	—
2137878.SAMN16056095.JAHDOI0100	**typeIII-A5**	232,8	low	Bacillota	Negativicutes	Selenomonadales	—	—	Selenomonadales bacterium	SAMN16056095	anthropogenic	10	Yes	4	HTH_17;HTH_Tnp_1_2;RH3_dom;	—
156588.SAMEA6955620.CAIMWS0100	**typeIII-A5**	227,8	low	Verrucomicrobiota	—	—	—	—	uncultured Verrucomicrobiota bacterium	SAMEA6955620	ph	7	Yes	4	HEAT_2;N6_N4_Mtase;DUF1848	—
282683.SAMN04488105.FNAV0100001	**typeIII-A5**	227,4	low	Pseudomonadota	Alphaproteobacteria	Rhodobacterales	Roseobacteraceae	Salipiger	Salipiger thiooxidans	SAMN04488105	aquatic	10	Yes	4	Mmel_Mtase;Mrr_N;Sigma70_r4;Pe	—
1895720.SAMN05660546.MKTD010000	**typeIII-A5**	226	low	Bacteroidota	Bacteroidia	—	—	—	Bacteroidia bacterium 43-41	SAMN05660546	anthropogenic	10	Yes	4	GMT-wHTH;Phage_integrase;PRTa	—
1798573.SAMN04313869.MHBH01000(	**typeIII-A5**	222,4	low	Lentisphaerota	—	—	—	—	Lentisphaerae bacterium GWF2_49_2	SAMN04313869	terrestrial	10	Yes	4	DUF983;ResIII;TPR_19;CBM_4_9	—
1798574.SAMN04313866.MHBI010000	**typeIII-A5**	221,2	low	Lentisphaerota	—	—	—	—	Lentisphaerae bacterium GWF2_50_9	SAMN04313866	terrestrial	10	Yes	4	BSH_RND;ACR_tran;ResIII;PUMA	—
2044936.SAMEA8395272.CAJPTC010(	**typeIII-A5**	219,7	low	Bacteroidota	Bacteroidia	—	—	—	Bacteroidia bacterium	SAMEA8395272	host-associated	10	Yes	4	PatX;PRTase-CE;Phage_int_SAM_	—
194843.SAMEA104666353.ONFO0100(	**typeIII-A5**	218,8	low	Bacteroidota	Bacteroidia	Bacteroidales	—	—	uncultured Bacteroidales bacterium	SAMEA104666353	host-associated	10	Yes	4	AAA_19;OLD-like_TOPRIM;PRTas	—
2024855.SAMN07619947.NZVD010000	**typeIII-A5**	218,5	low	Planctomycetota	Planctomycetia	Pirellulales	Pirellulaceae	Rhodopirellula	Rhodopirellula sp.	SAMN07619947	aquatic	10	Yes	4	SGL;Lum_binding;DUF6009	—
1349822.SAMN05444348.RBXN01000(	**typeIII-A5**	217,6	low	Bacteroidota	Bacteroidia	Bacteroidales	Barnesiellaceae	Coprobacter	Coprobacter fastidiosus	SAMN05444348	host-associated	10	Yes	4	HTH_17;Phage_int_SAM_5;PRTas	—
59823.SAMN11294431.SUYI01000044_	**typeIII-A5**	216,8	low	Bacteroidota	Bacteroidia	Bacteroidales	Prevotellaceae	Prevotella	Prevotella sp.	SAMN11294431	host-associated	10	Yes	4	UvrD-helicase;AAA_15;PRTase-CE	—
1129257.SAMEA6152838.CADCJI0100	**typeIII-A5**	216,7	low	Bacteroidota	Bacteroidia	—	—	—	uncultured Bacteroidia bacterium	SAMEA6152838	—	10	Yes	0	—	—
2026779.SAMN07619720.PAPJ010000	**typeIII-A5**	214,3	low	Planctomycetota	Planctomycetia	Planctomycetales	Planctomycetaceae	—	Planctomycetaceae bacterium	SAMN07619720	aquatic	10	Yes	4	SGL;Lum_binding;DUF6009	—
2005381.SAMN14533040.JABACA010(	**typeIII-A5**	213,9	low	Planctomycetota	Planctomycetia	Pirellulales	Pirellulaceae	Mariniblastus	Mariniblastus sp.	SAMN14533040	aquatic	6	Yes	3	PS_pyruv_trans;DUF5413	—
1952733.SAMN06454406.DEAW01000(	**typeIII-A5**	213,2	low	Planctomycetota	Planctomycetia	Planctomycetales	Planctomycetaceae	—	Planctomycetaceae bacterium UBA29	SAMN06454406	aquatic	5	Yes	2	SGL	—
2026780.SAMN10966917.VGZI0100011	**typeIII-A5**	208	low	Planctomycetota	—	—	—	—	Planctomycetota bacterium	SAMN10966917	aquatic	6	Yes	3	PAPS_reduct;AhpC-TSA;PDDEXK	—
2026779.SAMN14914450.JABHSU010(	**typeIII-A5**	206,3	low	Planctomycetota	Planctomycetia	Planctomycetales	Planctomycetaceae	—	Planctomycetaceae bacterium	SAMN14914450	aquatic	1	Yes	1	Lum_binding	—
1798574.SAMN04313866.MHBI010000	**typeIII-A5**	196,2	low	Lentisphaerota	—	—	—	—	Lentisphaerae bacterium GWF2_50_9	SAMN04313866	terrestrial	10	Yes	4	Prok-E2_D;DUF4942	—
2053306.SAMN18059852.JAFLBV0100	**typeIII-A5**	195,3	low	Ignavibacteriota	Ignavibacteria	—	—	—	Ignavibacteria bacterium	SAMN18059852	anthropogenic	10	Yes	4	Aminotran_5;MS_channel_2nd;Uvrl	—
2052166.SAMN15436385.JACRFZ010(	**typeIII-A5**	194,8	low	Candidatus Melainabacte	—	—	—	—	Candidatus Melainabacteria bacterium	SAMN15436385	anthropogenic	10	Yes	4	HigB_toxin;SMI1_KNR4	—
278095.SAMEA6946262.CAILFO01000	**typeIII-A5**	193,7	low	Lentisphaerota	—	—	—	—	uncultured Lentisphaerota bacterium	SAMEA6946262	aquatic	1	Yes	0	—	—
278095.SAMEA6946262.CAILFO01000	**typeIII-A5**	193,4	low	Lentisphaerota	—	—	—	—	uncultured Lentisphaerota bacterium	SAMEA6946262	aquatic	3	Yes	3	Cation_efflux	—
77133.SAMEA9695134.CAJXZK01000(	**typeIII-A5**	192,4	low	—	—	—	—	—	uncultured bacterium	SAMEA9695134	anthropogenic	5	Yes	2	ATLF;Thioredoxin_2	—
2364082.SAMN18076853.JAGVJU010(	**typeIII-A5**	191,1	low	Candidatus Melainabacte	—	Candidatus Obscuribacte	—	—	Candidatus Obscuribacterales bacteriu	SAMN18076853	aquatic	10	Yes	4	NLPC_P60;DUF4240;TPR_MalT	—
2026780.SAMN14533038.JABAAE010(	**typeIII-A5**	188,6	low	Planctomycetota	—	—	—	—	Planctomycetota bacterium	SAMN14533038	aquatic	10	Yes	3	DUF1579;Sulfatase	—
2035772.SAMN19297679.JAHIOU0100	**typeIII-A5**	187,4	low	Candidatus Omnitrophota	—	—	—	—	Candidatus Omnitrophota bacterium	SAMN19297679	terrestrial	5	Yes	4	IZUMO;PDDEXK_1;WDR62-MABF	—
1430440.SAMEA3138838.HG794546_3	**typeIII-A5**	185,2	low	Pseudomonadota	Alphaproteobacteria	Rhodospirillales	Rhodospirillaceae	Magnetospirillum	Magnetospirillum gryphiswaldense	SAMEA3138838	aquatic	10	Yes	4	SBP_bac_3;DDE_Tnp_IS66;DUF7	—
194843.SAMEA6151249.CADAAI01000	**typeIII-A5**	182	low	Bacteroidota	Bacteroidia	Bacteroidales	—	—	uncultured Bacteroidales bacterium	SAMEA6151249	host-associated	9	Yes	4	DUF6254;SH3_3;Flavin_Reduct	—
2597224.SAMN10849879.SHMQ01000(	**typeIII-A5**	179,7	low	—	Deltaproteobacteria	Candidatus Acidulodesulf	—	Candidatus Acidulodes	Candidatus Acidulodesulfobacterium a	SAMN10849879	anthropogenic	10	Yes	4	UBA_3;Phage_ORF5	—
1936112.SAMN14533044.JABABI01000	**typeIII-A5**	178,2	low	Planctomycetota	Planctomycetia	Planctomycetales	Planctomycetaceae	Fuerstiella	Fuerstiella sp.	SAMN14533044	aquatic	10	Yes	4	Flg_hook;DUF6702	—
2597224.SAMN10849879.SHMQ01000(	**typeIII-A5**	173,9	low	—	Deltaproteobacteria	Candidatus Acidulodesulf	—	Candidatus Acidulodes	Candidatus Acidulodesulfobacterium a	SAMN10849879	anthropogenic	10	Yes	4	UBA_3;Phage_ORF5;Ribosomal_L	—
370804.SAMEA104667047.ONJX0100(	**typeIII-A5**	173,6	low	Bacteroidota	Bacteroidia	Bacteroidales	Prevotellaceae	—	uncultured Prevotellaceae bacterium	SAMEA104667047	host-associated	10	Yes	4	MapZ_EC1;DUF6254;HepT-like	—
194843.SAMEA6151907.CADAZL0100(	**typeIII-A5**	170	low	Bacteroidota	Bacteroidia	Bacteroidales	—	—	uncultured Bacteroidales bacterium	SAMEA6151907	host-associated	10	Yes	4	MapZ_EC1;DUF6254;OMP_b-brl	—
2778646.SAMN16384127.JADMNM010	**typeIII-A5**	169,7	low	Pseudomonadota	Alphaproteobacteria	Sphingomonadales	Erythrobacteraceae	Qipengyuania	Qipengyuania sp. YIM B01966	SAMN16384127	anthropogenic	6	Yes	3	MarR_2;ABC1;Phage_int_SAM_5	—
1961416.SAMN06454634.DBZJ010002	**typeIII-A5**	160	low	Thermodesulfobacteriota	Syntrophia	Syntrophales	Syntrophaceae	—	Syntrophaceae bacterium UBA1163	SAMN06454634	salinity	10	Yes	4	DUF7683;HTH_3;AGTRAP	—
2268206.SAMN13894326.JAAYWD010	**typeIII-A5**	159,5	low	Thermotogota	Thermotogae	Thermotogales	Thermotogaceae	—	Thermotogaceae bacterium	SAMN13894326	anthropogenic	10	Yes	4	AAA_2;OrfB_IS605;TM2;DUF4350	—
2212472.SAMN12876714.WGDB01000(	**typeIII-A5**	131,6	low	Campylobacterota	Epsilonproteobacteria	Campylobacterales	Helicobacteraceae	—	Helicobacteraceae bacterium	SAMN12876714	temperature	10	Yes	4	Nitro_FeMo-Co;Rhodanese;Ham1p_	—
129578.SAMN06648058.NQJF0100000	**typeIV**	345,1	high	Pseudomonadota	Gammaproteobacteria	Aeromonadales	Aeromonadaceae	Oceanimonas	Oceanimonas baumannii	SAMN06648058	anthropogenic	6	Yes	2	ANC1_spectrin;Zn_ribbon_double	—
153809.SAMEA6953771.CAITHT01000	**typeIV**	337,2	high	Pseudomonadota	—	—	—	—	uncultured Pseudomonadota bacterium	SAMEA6953771	aquatic	11	Yes	4	DUF2730;Retron_Ec48_antiviral;Re	—
1236544.SAMD00046904.BCZT010001	**typeIV**	333,7	high	Pseudomonadota	Gammaproteobacteria	Alteromonadales	Shewanellaceae	Shewanella	Shewanella algae	SAMD00046904	host-associated	2	Yes	1	NERD	—
114715.SAMEA9694635.CAJXMR0100	**typeIV**	332,2	high	Pseudomonadota	Betaproteobacteria	Burkholderiales	Burkholderiaceae	Ralstonia	uncultured Ralstonia sp.	SAMEA9694635	aquatic	8	Yes	4	Mrr_cat;Retron_Ec48_antiviral	—
472181.SAMN09638568.DRHT0100000	**typeIV**	314,7	high	Pseudomonadota	Gammaproteobacteria	Pseudomonadales	Pseudomonadaceae	Halopseudomonas	Halopseudomonas sabulinigri	SAMN09638568	aquatic	7	Yes	3	EamA;Iso_dh;Retron_Ec48_antivira	—
1406866.SAMN02732283.AXZR010000	**typeIX**	292,3	high	Pseudomonadota	Alphaproteobacteria	Rhodobacterales	Roseobacteraceae	Sulfitobacter	Sulfitobacter pontiacus	SAMN02732283	aquatic	10	Yes	4	DUF6602;MarR;GIY-YIG	—
1898112.SAMN14915609.JABJKH0100	**typeIX**	289,5	high	Pseudomonadota	Alphaproteobacteria	Rhodospirillales	Rhodospirillaceae	—	Rhodospirillaceae bacterium	SAMN14915609	aquatic	9	Yes	4	DUF2779;ARMET_N	—
1977087.SAMN19298580.JAHJTB0100	**typeIX**	288,4	high	Pseudomonadota	—	—	—	—	Pseudomonadota bacterium	SAMN19298580	terrestrial	8	Yes	4	HDOD;HD_5;GGDEF_2;TrkA_N	—
383381.SAMN02737000.JMIV0100000	**typeIX**	281,3	high	Pseudomonadota	Alphaproteobacteria	Sphingomonadales	Erythrobacteraceae	Erythrobacter	Erythrobacter sp. JL475	SAMN02737000	aquatic	11	Yes	4	NMB0537_N;Phage_integrase;ARM	—
2026779.SAMN10607091.SKZY010003	**typeIX**	259,4	high	Planctomycetota	Planctomycetia	Planctomycetales	Planctomycetaceae	—	Planctomycetaceae bacterium	SAMN10607091	aquatic	7	Yes	3	GGDEF_2;MauE;PIN	—
998088.SAMN02603940.CP002607_39	**typeV**	335,4	high	Pseudomonadota	Gammaproteobacteria	Aeromonadales	Aeromonadaceae	Aeromonas	Aeromonas veronii	SAMN02603940	host-associated	11	Yes	4	CSD	—
1196835.SAMN02603608.CP003677_4	**typeV**	328,3	high	Pseudomonadota	Gammaproteobacteria	Pseudomonadales	Pseudomonadaceae	Stutzerimonas	Stutzerimonas stutzeri	SAMN02603608	terrestrial	11	Yes	4	GTP_EFTU;Tom5;CSD;VirionAsse	—
56192.SAMN07327741.PYLU01000035	**typeV**	321,7	high	Pseudomonadota	Gammaproteobacteria	Vibrionales	Vibrionaceae	Photobacterium	Photobacterium iliopiscarium	SAMN07327741	host-associated	8	Yes	4	Clathrin-link;CSD;CbiA	—
518738.SAMN13287348.JAACSM0100(	**typeV**	320,3	high	Pseudomonadota	Gammaproteobacteria	Alteromonadales	Shewanellaceae	Shewanella	Shewanella vesiculosa	SAMN13287348	terrestrial	11	Yes	4	DUF6037;CSD;Clathrin-link;ASA5	—
1283291.SAMN11792376.VEDF010000	**typeV**	319,6	high	Pseudomonadota	Gammaproteobacteria	Pseudomonadales	Pseudomonadaceae	Pseudomonas	Pseudomonas sp. URMO17WK12:I11	SAMN11792376	anthropogenic	11	Yes	4	LysR_substrate;CSD	—
1882827.SAMN05443579.FOWG01000	**typeV**	298,2	high	Pseudomonadota	Betaproteobacteria	Burkholderiales	Comamonadaceae	Variovorax	Variovorax sp. PDC80	SAMN05443579	anthropogenic	11	Yes	4	tRNA-synt_1c;Peptidase_M23;AstE	—
83655.SAMN13547356.WVWB0100000	**typeV**	296,2	high	Pseudomonadota	Gammaproteobacteria	Enterobacterales	Enterobacteriaceae	Leclercia	Leclercia adecarboxylata	SAMN13547356	anthropogenic	11	Yes	4	T6SS_HCP;SymE_toxin;DUF443;N	—
1566294.SAMN03159417.FOWV01000	**typeV**	294,9	high	Pseudomonadota	Betaproteobacteria	Burkholderiales	Burkholderiaceae	Ralstonia	Ralstonia sp. NFACC01	SAMN03159417	host-associated	11	Yes	4	Mrr_cat;CSD	—
180197.SAMN02982919.FOGD0100000	**typeV**	294,4	high	Pseudomonadota	Betaproteobacteria	Burkholderiales	Comamonadaceae	Giesbergeria	Giesbergeria anulus	SAMN02982919	anthropogenic	11	Yes	4	CBS;CSD;LYTB	—
2775292.SAMN16237464.JACYUQ010(	**typeV**	284,7	high	Pseudomonadota	Betaproteobacteria	Burkholderiales	Oxalobacteraceae	—	Oxalobacteraceae sp. CFBP 13730	SAMN16237464	anthropogenic	3	Yes	2	TGS	—
63.SAMN07313362.CP022423_1311	**typeV**	268	borderline	Pseudomonadota	Betaproteobacteria	Neisseriales	Neisseriaceae	Vitreoscilla	Vitreoscilla filiformis	SAMN07313362	anthropogenic	10	Yes	4	Transket_pyr;CSD;LysR_substrate	—
1004785.SAMN02604118.CP003845_2	**typeVI**	266,7	high	Pseudomonadota	Gammaproteobacteria	Alteromonadales	Alteromonadaceae	Alteromonas	Alteromonas macleodii	SAMN02604118	aquatic	11	Yes	4	LCAT;DUF7379;HTH_3	—
562.SAMN13545722.CP047609_747	**typeVI**	260,8	high	Pseudomonadota	Gammaproteobacteria	Enterobacterales	Enterobacteriaceae	Escherichia	Escherichia coli	SAMN13545722	host-associated	11	Yes	4	HTH_3;FRG;Phage_portal	—
748224.SAMN00189147.GL538343_33	**typeVI**	239,5	high	Bacillota	Clostridia	Eubacteriales	Oscillospiraceae	Faecalibacterium	Faecalibacterium prausnitzii	SAMN00189147	host-associated	11	Yes	4	HTH_3;DUF6050;Relaxase	—
862719.PRJEA50367.FQ311872_41	**typeVI**	236,5	high	Pseudomonadota	Alphaproteobacteria	Rhodospirillales	Azospirillaceae	Azospirillum	Azospirillum lipoferum	PRJEA50367	aquatic	11	Yes	4	UPF0020;Amanitin;Beta_elim_lyase	—
169435.SAMN15533212.JADNIE01000(	**typeVI**	236,3	high	Bacillota	Clostridia	Eubacteriales	Oscillospiraceae	Anaerotruncus	Anaerotruncus colihominis	SAMN15533212	host-associated	11	Yes	0	—	—
1979412.SAMN14944538.JABMOK010(	**typeVI**	235,6	high	Pseudomonadota	Gammaproteobacteria	Pseudomonadales	Pseudohongiellaceae	Pseudohongiella	Pseudohongiella sp.	SAMN14944538	temperature	11	Yes	4	HTH_3;DUF2951;Acetyltransf_10	—
2044939.SAMN16344672.JAFTWE010(	**typeVI**	235,3	high	Bacillota	Clostridia	—	—	—	Clostridia bacterium	SAMN16344672	host-associated	11	Yes	4	HTH_3;ADPrib_exo_Tox	—
2485925.SAMN16343178.JAFRQP010(	**typeVI**	235,1	high	Bacillota	Clostridia	Eubacteriales	Oscillospiraceae	—	Oscillospiraceae bacterium	SAMN16343178	host-associated	11	Yes	4	Mob_Pre;SlyX;Response_reg;HATF	—
556261.SAMN02463701.GL870812_31	**typeVI**	234,3	high	Bacillota	Clostridia	Eubacteriales	Clostridiaceae	Clostridium	Clostridium sp. D5	SAMN02463701	host-associated	11	Yes	4	HTH_3;KilA-N	—
153809.SAMEA6946215.CAIXHP01000	**typeVI**	232,9	high	Pseudomonadota	—	—	—	—	uncultured Pseudomonadota bacterium	SAMEA6946215	aquatic	11	Yes	4	DNA_methylase;RE_SacI;HTH_3	—
2109688.SAMD00113750.AP018533_2	**typeVI**	232,4	high	Bacillota	Clostridia	Eubacteriales	—	—	Clostridiales bacterium CCNA10	SAMD00113750	host-associated	11	Yes	4	Relaxase;DUF6050;HTH_3	—
1895845.SAMN05660629.MKWI010000(	**typeVI**	231,2	high	Pseudomonadota	Alphaproteobacteria	Sphingomonadales	Sphingobiaceae	Sphingobium	Sphingobium sp. 66-54	SAMN05660629	anthropogenic	11	Yes	4	ThsB_TIR;Resolvase;HTH_3;Dicer	—

1930536.SAMN17140639.JAFAZF0100(	typeVI	230,1	high	Pseudomonadota	Alphaproteobacteria	Sphingomonadales	Sphingomonadaceae	—	Sphingomonadaceae bacterium	SAMN17140639	host-associated	11	Yes	4	HTH_3;DUF2278;His_Phos_1	—
759821.SAMN09630405.QOHO010000	typeVI	228,7	high	Bacillota	Clostridia	Lachnospirales	Lachnospiraceae	Lacrimispora	Lacrimispora amygdalina	SAMN09630405	anthropogenic	6	Yes	2	HTH_3	—
1410652.SAMN02744012.JHXK010000	typeVI	227,2	high	Bacillota	Clostridia	Eubacteriales	Clostridiaceae	Clostridium	Clostridium beijerinckii	SAMN02744012	host-associated	9	Yes	4	Imp-YgjV;HTH_3;HSDR_N_2;TelK_	—
395358.SAMEA9694658.CAJXPX0100(	typeVI	226,8	high	Pseudomonadota	Alphaproteobacteria	Hyphomonadales	Hyphomonadaceae	—	uncultured Hyphomonadaceae bacteri	SAMEA9694658	temperature	10	Yes	4	HEPN_Toprim_N;HTH_3	—
1031594.SAMN09074687.QPJU010000	typeVI	225,5	high	Pseudomonadota	Betaproteobacteria	Burkholderiales	Comamonadaceae	Extensimonas	Extensimonas vulgaris	SAMN09074687	anthropogenic	11	Yes	4	QatB;KAP_NTPase;HTH_3;ARM_l	—
266264.SAMN02598450.CP000352_14	typeVI	225,5	high	Pseudomonadota	Betaproteobacteria	Burkholderiales	Burkholderiaceae	Cupriavidus	Cupriavidus metallidurans	SAMN02598450	—	11	Yes	0	—	—
1895859.SAMN05660633.MKWM0100(	typeVI	225,5	high	Pseudomonadota	Betaproteobacteria	Nitrosomonadales	Thiobacillaceae	Thiobacillus	Thiobacillus sp. 63-78	SAMN05660633	—	11	Yes	0	—	—
1940281.SAMN19405844.JAHRFE0100	typeVI	224,3	high	Pseudomonadota	Alphaproteobacteria	Hyphomicrobiales	Rhizobiaceae	Hoeflea	Hoeflea sp.	SAMN19405844	terrestrial	11	Yes	4	Phage_integrase;Resolvase;HTH_3	—
1798255.SAMN04315987.MGXA01000(	typeVI	223,8	high	Pseudomonadota	Betaproteobacteria	Nitrosomonadales	—	—	Gallionellales bacterium RIFCSPLOW(	SAMN04315987	terrestrial	11	Yes	4	Rhodanese;ARM_LIN;DUF4325;HT	—
1122616.SAMN02441143.AULT010000	typeVI	223,5	high	Pseudomonadota	Gammaproteobacteria	Oceanospirillales	Oceanospirillaceae	Oceanospirillum	Oceanospirillum beijerinckii	SAMN02441143	aquatic	11	Yes	4	YdiH;HTH_3;PIN;MazE_antitoxin	—
2052162.SAMN11532980.VIKH010000(	typeVI	222,3	high	Pseudomonadota	Betaproteobacteria	Nitrosomonadales	Gallionellaceae	—	Gallionellaceae bacterium	SAMN11532980	terrestrial	11	Yes	4	Bac_export_3;Bac_export_1;Chor_l	—
1913988.SAMN19298773.JAHKAM0100	typeVI	222,2	high	Pseudomonadota	Alphaproteobacteria	—	—	—	Alphaproteobacteria bacterium	SAMN19298773	anthropogenic	11	Yes	4	TonB_dep_Rec_b-barrel;UvrB_inter	—
2779351.SAMN16401875.JADCKL0100	typeVI	221,6	high	Bacillota	Clostridia	Lachnospirales	Lachnospiraceae	Claveliimonas	Claveliimonas monacensis	SAMN16401875	host-associated	11	Yes	4	BsuBI_PstI_RE;Eco57I;HTH_3	—
1236514.SAMD00010222.BAKL010001	typeVI	220,2	high	Bacteroidota	Bacteroidia	Bacteroidales	Bacteroidaceae	Bacteroides	Bacteroides stercorirosoris	SAMD00010222	host-associated	9	Yes	4	DUF5347;HTH_3;Fib_succ_major	—
1321819.SAMN02436886.KE993103_2	typeVI	219	high	Bacteroidota	Bacteroidia	Bacteroidales	Bacteroidaceae	Bacteroides	Bacteroides pyogenes	SAMN02436886	host-associated	7	Yes	3	DDE_Tnp_ISL3;HTH_3;DUF5347	—
1232436.SAMEA2272249.CAPF010000	typeVI	218,8	high	Actinomycetota	Coriobacteriia	Coriobacteriales	Coriobacteriaceae	Enorma	Enorma timonensis	SAMEA2272249	host-associated	11	Yes	4	ResIII;N6_N4_Mtase;HTH_3	—
1123279.SAMN02440570.ATUS010000	typeVI	217,9	high	Pseudomonadota	Gammaproteobacteria	Cellvibrionales	Spongiibacteraceae	Spongiibacter	Spongiibacter tropicus	SAMN02440570	temperature	11	Yes	4	AAA_13;Apo-VLDL-II;HTH_3;ARM_	—
2137880.SAMEA8805625.CAJUJH0100	typeVI	217,5	high	Candidatus Melainabacte	—	Candidatus Gastranaerop	—	—	Candidatus Gastranaerophilales bacte	SAMEA8805625	host-associated	11	Yes	4	Methylase_S;N6_Mtase;HTH_3;Mla	—
2212467.SAMN16344105.JAFTAJ0100	typeVI	216	high	Bacteroidota	Bacteroidia	Bacteroidales	Bacteroidaceae	—	Bacteroidaceae bacterium	SAMN16344105	—	8	Yes	0	—	—
370804.SAMEA6152912.CADCMC0100(	typeVI	216	high	Bacteroidota	Bacteroidia	Bacteroidales	Prevotellaceae	—	uncultured Prevotellaceae bacterium	SAMEA6152912	host-associated	8	Yes	0	—	—
876091.SAMEA8805256.CAJTZK01000	typeVI	215,6	high	Bacillota	Clostridia	Eubacteriales	Oscillospiraceae	Oscillibacter	uncultured Oscillibacter sp.	SAMEA8805256	host-associated	11	Yes	4	DegV;Phage_integrase;DUF6809	—
194843.SAMEA6150337.CACYRK0100	typeVI	215,2	high	Bacteroidota	Bacteroidia	Bacteroidales	—	—	uncultured Bacteroidales bacterium	SAMEA6150337	host-associated	9	Yes	4	HATPase_c;Response_reg;TerC;A	—
317.SAMN16237900.JACYWI01000001	typeVI	215,2	high	Pseudomonadota	Gammaproteobacteria	Pseudomonadales	Pseudomonadaceae	Pseudomonas	Pseudomonas syringae	SAMN16237900	host-associated	11	Yes	4	DUF4172;GFA	—
853.SAMN07350508.NMTY01000004_4	typeVI	215,2	high	Bacillota	Clostridia	Eubacteriales	Oscillospiraceae	Faecalibacterium	Faecalibacterium prausnitzii	SAMN07350508	host-associated	11	Yes	4	HD_5;Response_reg;DUF4300	—
718252.SAMEA3138378.FP929045_22	typeVI	215,1	high	Bacillota	Clostridia	Eubacteriales	Oscillospiraceae	Faecalibacterium	Faecalibacterium prausnitzii	SAMEA3138378	host-associated	11	Yes	4	RuvC;Relaxase	—
748224.SAMN00189147.GL538328_11	typeVI	214,9	high	Bacillota	Clostridia	Eubacteriales	Oscillospiraceae	Faecalibacterium	Faecalibacterium prausnitzii	SAMN00189147	host-associated	7	Yes	0	—	—
445971.SAMN02299418.DS560015_20	typeVI	214,7	high	Bacillota	Clostridia	Eubacteriales	Eubacteriaceae	Anaerofustis	Anaerofustis stercorihominis	SAMN02299418	host-associated	11	Yes	4	BtrH_N;Fungus-induced;Peptidase_	—
551459.SAMN06049055.MUGO010000	typeVI	214,2	high	Bacteroidota	Flavobacteriia	Flavobacteriales	Weeksellaceae	Chryseobacterium	Chryseobacterium piscicola	SAMN06049055	anthropogenic	11	Yes	4	DUF5347;HTH_3;NERD;Pdc1_Ge	—
59823.SAMN16344881.JAFUEF010000	typeVI	214,1	high	Bacteroidota	Bacteroidia	Bacteroidales	Prevotellaceae	Prevotella	Prevotella sp.	SAMN16344881	—	6	Yes	0	—	—
59620.SAMEA4892316.USGX0100008	typeVI	213,8	high	Bacillota	Clostridia	Eubacteriales	Clostridiaceae	Clostridium	uncultured Clostridium sp.	SAMEA4892316	host-associated	2	Yes	1	YnfE	—
1776379.SAMEA3932115.LT556050_3	typeVI	213,6	high	Bacteroidota	Bacteroidia	Bacteroidales	Prevotellaceae	Leyella	Leyella lascolaii	SAMEA3932115	—	11	Yes	0	—	—
537013.SAMN00008812.EQ973344_64	typeVI	213,5	high	Bacillota	Clostridia	Eubacteriales	Oscillospiraceae	—	[Clostridium] methylpentosum	SAMN00008812	—	11	Yes	0	—	—
2485925.SAMN16342071.JAFQCV0100	typeVI	212,5	high	Bacillota	Clostridia	Eubacteriales	Oscillospiraceae	—	Oscillospiraceae bacterium	SAMN16342071	host-associated	11	Yes	4	zf_PCFS4;Recombinase;Zona_CL2	—
2030927.SAMN14407242.JAAVFI0100(	typeVI	211,7	high	Bacteroidota	Bacteroidia	Bacteroidales	—	—	Bacteroidales bacterium	SAMN14407242	host-associated	11	Yes	4	ResIII;Reeler	—
2748031.SAMN16347300.JAFXXG0100	typeVI	211,6	high	Bacteroidota	Bacteroidia	Bacteroidales	Paludibacteraceae	—	Paludibacteraceae bacterium	SAMN16347300	—	5	Yes	0	—	—
59823.SAMN16343164.JAFRQB01000(	typeVI	211,6	high	Bacteroidota	Bacteroidia	Bacteroidales	Prevotellaceae	Prevotella	Prevotella sp.	SAMN16343164	host-associated	8	Yes	0	—	—
370804.SAMEA6149422.CACXIP01000	typeVI	211,2	high	Bacteroidota	Bacteroidia	Bacteroidales	Prevotellaceae	—	uncultured Prevotellaceae bacterium	SAMEA6149422	host-associated	7	Yes	0	—	—
2044939.SAMN16347922.JAFYVE0100	typeVI	211,2	high	Bacillota	Clostridia	—	—	—	Clostridia bacterium	SAMN16347922	—	11	Yes	0	—	—
2720829.SAMEA7202527.CAJFOI0100	typeVI	211,2	high	Bacillota	Clostridia	—	—	Candidatus Avispirillum	Candidatus Avispirillum faecium	SAMEA7202527	host-associated	11	Yes	4	Rhomboid;RE_MjaI	—
1161415.SAMN02597443.AZYV010000	typeVI	211,2	high	Bacillota	Bacilli	Lactobacillales	Streptococcaceae	Streptococcus	Streptococcus sp. BS29a	SAMN02597443	host-associated	11	Yes	4	DUF4435;Methylase_S;DUF6341;D	—
871327.SAMN05444001.FNVS0100002	typeVI	211	high	Bacteroidota	Bacteroidia	Bacteroidales	Tannerellaceae	Parabacteroides	Parabacteroides chinchillae	SAMN05444001	host-associated	11	Yes	4	HTH_17;FliS_cochap;YnfE;Virulenc	—
2718.SAMEA8395222.CAJPRL0100000	typeVI	210,6	high	Pseudomonadota	Gammaproteobacteria	Cardiobacteriales	Cardiobacteriaceae	Cardiobacterium	Cardiobacterium hominis	SAMEA8395222	host-associated	9	Yes	4	FUSC-like;Competence;YdiH	—
1121870.SAMN02440607.AUAA010000	typeVI	210,3	high	Bacteroidota	Flavobacteriia	Flavobacteriales	Weeksellaceae	Epilithonimonas	Epilithonimonas tenax	SAMN02440607	—	10	Yes	0	—	—
2485925.SAMEA6152165.CADBJK0100	typeVI	209,7	high	Bacillota	Clostridia	Eubacteriales	Oscillospiraceae	—	Oscillospiraceae bacterium	SAMEA6152165	host-associated	11	Yes	4	Flg_new_2;Zn-C2H2_12	—
1796613.SAMN04621613.CP015401_8	typeVI	209,3	high	Bacteroidota	Bacteroidia	Bacteroidales	Bacteroidaceae	Bacteroides	Bacteroides caecimuris	SAMN04621613	host-associated	11	Yes	0	—	—
194843.SAMEA7847407.CAJLWQ0100	typeVI	209	high	Bacteroidota	Bacteroidia	Bacteroidales	—	—	uncultured Bacteroidales bacterium	SAMEA7847407	host-associated	7	Yes	3	LMBR1;Fic	—
1122147.SAMN02369451.AZFW01000(	typeVI	207,9	high	Bacillota	Bacilli	Lactobacillales	Lactobacillaceae	Schleiferilactobacillus	Schleiferilactobacillus harbinensis	SAMN02369451	host-associated	11	Yes	4	GT2_TM_C;Peptidase_M78;HTH_3	—
186571.SAMN10864689.SLXM0100001	typeVI	207,8	high	Bacteroidota	Flavobacteriia	Flavobacteriales	Flavobacteriaceae	Tenacibaculum	Tenacibaculum skagerrakense	SAMN10864689	aquatic	11	Yes	4	DUF5347;HTH_3;Peptidase_S58;V	—
2795120.SAMN17004923.JAELUC0100	typeVI	207,5	high	Bacteroidota	Sphingobacteriia	Sphingobacteriales	Sphingobacteriaceae	Pedobacter	Pedobacter sp. ASV12	SAMN17004923	host-associated	1	No	0	—	—
2048242.SAMEA104588875.LT985749_	typeVI	206,9	high	Bacteroidota	Bacteroidia	Bacteroidales	Rikenellaceae	Alistipes	Alistipes sp. Marseille-P5061	SAMEA104588875	host-associated	11	Yes	4	ParB_N;HTH_3	—
165185.SAMEA7847541.CAJMER0100	typeVI	206,9	high	Bacillota	Clostridia	Eubacteriales	Eubacteriaceae	Eubacterium	uncultured Eubacterium sp.	SAMEA7847541	host-associated	8	Yes	4	PFam54_60;SseF;Fic;rve	—
658143.SAMEA4891723.URKB0100000	typeVI	206,3	high	Mycoplasmatota	—	—	—	—	uncultured Mycoplasmatota bacterium	SAMEA4891723	host-associated	10	Yes	4	DUF4010;Helicase_C;DUF4391	—
449673.SAMN00000004.DS499667_48	typeVI	205,9	high	Bacteroidota	Bacteroidia	Bacteroidales	Bacteroidaceae	Bacteroides	Bacteroides stercoris	SAMN00000004	—	11	Yes	0	—	—
1898104.SAMN08179015.PMXD01000	typeVI	205,6	high	Bacteroidota	—	—	—	—	Bacteroidota bacterium	SAMN08179015	temperature	11	Yes	4	DUF5347;HTH_3;WG_beta_rep;PH	—
1975676.SAMN05954931.MRCM01000	typeVI	205,5	high	Bacteroidota	Flavobacteriia	Flavobacteriales	Flavobacteriaceae	Flavobacterium	Flavobacterium sp. ACN2	SAMN05954931	host-associated	11	Yes	4	DUF5347;HTH_3;Lipase_GDSL_2	—
290398.SAMN02598275.CP000285_12	typeVI	205,1	high	Pseudomonadota	Gammaproteobacteria	Oceanospirillales	Halomonadaceae	Chromohalobacter	Chromohalobacter israelensis	SAMN02598275	aquatic	11	Yes	4	Phage_integrase;PyocinActivator	—
1298593.SAMEA2272589.HF680312_3	typeVI	205,1	high	Pseudomonadota	Gammaproteobacteria	Oceanospirillales	Oceanospirillaceae	Thalassolituus	Thalassolituus oleivorans	SAMEA2272589	aquatic	11	Yes	4	Peptidase_M19;Resolvase	—
1191309.SAMN02470478.AJZH020000	typeVI	204,1	high	Pseudomonadota	Gammaproteobacteria	Vibrionales	Vibrionaceae	Vibrio	Vibrio splendidus	SAMN02470478	aquatic	11	Yes	4	SIS;Cys_Met_Meta_PP;HTH_3	—
2320122.SAMN10024804.RAYW01000(	typeVI	203,8	high	—	—	—	—	—	bacterium J10(2018)	SAMN10024804	host-associated	5	Yes	3	ATPase_2;APC_r;DDE_Tnp_ISL3	—
1898104.SAMN19298609.JAHJUE0100	typeVI	203,4	high	Bacteroidota	—	—	—	—	Bacteroidota bacterium	SAMN19298609	terrestrial	11	Yes	4	Bul1_C;HTH_3;DUF2326;MC6	—
25.SAMN12272628.VKGK01000009_11	typeVI	202,8	high	Pseudomonadota	Gammaproteobacteria	Alteromonadales	Shewanellaceae	Shewanella	Shewanella hanedai	SAMN12272628	temperature	11	Yes	4	HTH_3;Fapy_DNA_glyco	—
2049046.SAMN09989679.QYQM01000	typeVI	202,6	high	Bacteroidota	Bacteroidia	Bacteroidales	Porphyromonadaceae	—	Porphyromonadaceae bacterium	SAMN09989679	aquatic	11	Yes	4	DUF5347;HTH_3;Trypsin_2	—
1898104.SAMN21435362.JAIUNW0100	typeVI	198,9	high	Bacteroidota	—	—	—	—	Bacteroidota bacterium	SAMN21435362	anthropogenic	10	Yes	4	YafQ_toxin;HTH_3;HTH_Tnp_1;rve	—
2049048.SAMN11294459.SUZK010000	typeVI	194,2	high	Bacteroidota	Bacteroidia	Bacteroidales	Rikenellaceae	—	Rikenellaceae bacterium	SAMN11294459	host-associated	11	Yes	4	KAP_NTPase;GloB_C	—
2026760.SAMN10744134.SCKI0100000	typeVI	192,5	high	Fidelibacterota	—	—	—	—	Fidelibacterota bacterium	SAMN10744134	aquatic	11	Yes	4	Fer4_21;DsrE;Esterase	—
880526.SAMN02441234.KE386488_19	typeVI	192,2	high	Bacteroidota	Bacteroidia	Bacteroidales	Rikenellaceae	Rikenella	Rikenella microfusus	SAMN02441234	host-associated	11	Yes	4	DUF6236	—
2052142.SAMN08179812.PMAT010000	typeVI	191,9	high	Acidobacteriota	Terriglobia	Terriglobales	Acidobacteriaceae	—	Acidobacteriaceae bacterium	SAMN08179812	terrestrial	11	Yes	4	DDE_Tnp_1_assoc;CBM_48;HTH_	—
2052142.SAMN08179208.PLGM01000	typeVI	190,6	high	Acidobacteriota	Terriglobia	Terriglobales	Acidobacteriaceae	—	Acidobacteriaceae bacterium	SAMN08179208	temperature	6	Yes	2	type_II_gspD_N0;HTH_3	—
1895771.SAMN05660602.MKVH01000(	typeVI	189,2	high	Candidatus Kapaibacteric	Candidatus Kapaibacte	Candidatus Kapaibacteria	Candidatus Kapaibacteria	Candidatus Kapaibacte	Candidatus Kapaibacterium thiocyanat	SAMN05660602	anthropogenic	11	Yes	4	rve;HTH_Tnp_1;Nuc_deoxyrib_tr	—
2282154.SAMN11533020.WSYB01000(	typeVI	188,3	high	Thermodesulfobacteriota	Desulfuromonadia	Geobacterales	Geobacteraceae	—	Geobacteraceae bacterium	SAMN11533020	terrestrial	11	Yes	4	MTES_1575;SNase;HTH_3;DUF67	—
1978231.SAMN08912159.QHZN01000	typeVI	183,8	high	Acidobacteriota	—	—	—	—	Acidobacteriota bacterium	SAMN08912159	terrestrial	11	Yes	4	HTH_37;DUF5955;Big_2;STN	—
2035772.SAMEA6955419.CAIQIN0100	typeVI	175,5	high	Candidatus Omnitrophota	—	—	—	—	Candidatus Omnitrophota bacterium	SAMEA6955419	aquatic	11	Yes	4	SPC25;PDDEXK_1;HTH_3;TPR_II	—
2026778.SAMN13893689.JAAYCR0100	typeVI	174,2	high	Planctomycetota	Phycisphaerae	—	—	—	Phycisphaerae bacterium	SAMN13893689	temperature	11	Yes	4	Resolvase;MarR_2;HTH_3;DAXX_	—
2528828.SAMN11124364.SOIB010001	typeVI	173,1	high	Candidatus Stahliibacteri	—	—	—	—	Candidatus Stahliibacteriota bacterium	SAMN11124364	aquatic	3	Yes	2	HTH_3;DUF7683	—
876.SAMN09779309.CP036295_2579	typeVI	172	high	Thermodesulfobacteriota	Desulfovibrionia	Desulfovibrionales	Desulfovibrionaceae	Desulfovibrio	Desulfovibrio desulfuricans	SAMN09779309	anthropogenic	11	Yes	4	HTH_3;Phage_integrase;Phage_co	—
152509.SAMEA6955447.CAIVKC01000	typeVI	170,3	high	Bacteroidota	—	—	—	—	uncultured Bacteroidota bacterium	SAMEA6955447	aquatic	11	Yes	4	ABC2_membrane_3;Transglut_prok	—
529385.SAMEA8805564.CAJUHZ0100	typeVI	167,7	high	Thermodesulfobacteriota	Desulfovibrionia	Desulfovibrionales	Desulfovibrionaceae	Bilophila	uncultured Bilophila sp.	SAMEA8805564	host-associated	7	Yes	3	Flagellin_C;HTH_3;Glyco_transf_9	—
58621.SAMN14908373.JACHGO01000	typeVI	163,5	high	Thermodesulfobacteriota	Desulfovibrionia	Desulfovibrionales	Desulfovibrionaceae	Desulfovibrio	Desulfovibrio intestinalis	SAMN14908373	host-associated	11	Yes	4	HTH_3;SEC-C;IncFII_repA	—
2049043.SAMN19225054.JAHZEL0100	typeVI	162,8	low	Thermodesulfobacteriota	Desulfovibrionia	Desulfovibrionales	Desulfovibrionaceae	—	Desulfovibrionaceae bacterium	SAMN19225054	host-associated	10	Yes	4	Peptidase_S58;HATPase_c	—

2049428.SAMN16425662.JADJEI01000(	**typeVI**	160,3	low	Ignavibacteriota	Ignavibacteria	Ignavibacteriales	—	—	Ignavibacteriales bacterium	SAMN16425662	anthropogenic	10	Yes	4	Y1_Tnp;HTH_3;LETM1_C	—
2026779.SAMN13893644.JAAYAY0100	**typeVI**	157,9	low	Planctomycetota	Planctomycetia	Planctomycetales	Planctomycetaceae	—	Planctomycetaceae bacterium	SAMN13893644	temperature	10	Yes	4	DUF2461;HTH_31;HTH_PafC	—
576117.SAMN04432131.LRUD0100000	**typeVII-A1**	351	high	Pseudomonadota	Alphaproteobacteria	Rhodobacterales	Roseobacteraceae	Celeribacter	Celeribacter halophilus	SAMN04432131	aquatic	11	Yes	4	MerR-DNA-bind;Hydrolase;Lsr2_DN	—
1912891.SAMN07426584.QFOA010000	**typeVII-A1**	327,6	high	Pseudomonadota	Alphaproteobacteria	Sphingomonadales	Sphingobiaceae	Sphingobium	Sphingobium sp.	SAMN07426584	anthropogenic	11	Yes	4	TPR_14	—
1977087.SAMN21435408.JAIUOU0100	**typeVII-A1**	321,3	high	Pseudomonadota	—	—	—	—	Pseudomonadota bacterium	SAMN21435408	—	7	Yes	0	—	—
1035191.SAMN02436690.KB291762_8	**typeVII-A1**	320	high	Pseudomonadota	Alphaproteobacteria	Caulobacterales	Caulobacteraceae	Brevundimonas	Brevundimonas diminuta	SAMN02436690	terrestrial	9	Yes	4	DUF2200;MPTase-PolyVal;DUF29	—
751586.SAMN02469913.GL883086_93	**typeVII-A1**	320	high	Pseudomonadota	Alphaproteobacteria	Caulobacterales	Caulobacteraceae	Brevundimonas	Brevundimonas diminuta	SAMN02469913	—	11	Yes	0	—	—
1977087.SAMN08158042.PKDC010000	**typeVII-A1**	319,1	high	Pseudomonadota	—	—	—	—	Pseudomonadota bacterium	SAMN08158042	terrestrial	11	Yes	4	Hydrolase;Thioredoxin_4;SseB	—
2282150.SAMN18059876.JAFLCT0100	**typeVII-A1**	294,3	borderline	Pseudomonadota	Alphaproteobacteria	Caulobacterales	—	—	Caulobacterales bacterium	SAMN18059876	anthropogenic	4	Yes	3	Cpn10;Cpn60_TCP1;DUF4168	—
1797342.SAMN04313819.MEOF010000	**typeVII-A2**	303,1	high	Bacteroidota	—	—	—	—	Bacteroidetes bacterium GWF2_33_3	SAMN04313819	terrestrial	11	Yes	4	DUF6602;DUF3800;PFK;PfkB	—
1948560.SAMN06451549.DIWM010000	**typeVII-A2**	291,3	borderline	—	Deltaproteobacteria	—	—	—	Deltaproteobacteria bacterium UBA61	SAMN06451549	anthropogenic	10	Yes	4	Trypsin_2;DUF3800;AlaDh_PNT_C	—
1977087.SAMN15717994.JAGDMU010	**typeVII-A2**	174,9	low	Pseudomonadota	—	—	—	—	Pseudomonadota bacterium	SAMN15717994	aquatic	10	Yes	4	Polyketide_cyc;Phage_integrase;Cp	—
1032480.SAMD00061117.AP012204_4	**typeVII-A2**	167,2	low	Actinomycetota	Actinomycetes	Propionibacteriales	Propionibacteriaceae	Microlunatus	Microlunatus phosphovorus	SAMD00061117	anthropogenic	10	Yes	4	DDE_Tnp_1_assoc;Complex1_LYR	—
1948743.SAMN06451839.DLRY010000	**typeX**	326,4	high	Verrucomicrobiota	Opitutia	—	—	—	Opitutia bacterium UBA7876	SAMN06451839	aquatic	8	Yes	4	3keto-disac_hyd;MTES_1575;Bac_	—
2026801.SAMN13495009.WTHK01000	**typeX**	323,6	high	Verrucomicrobiota	Verrucomicrobiia	Verrucomicrobiales	—	—	Verrucomicrobiales bacterium	SAMN13495009	aquatic	6	Yes	2	TRAP-delta;Abhydrolase_7	—
2026801.SAMN07620217.PAPL010000	**typeX**	310,1	high	Verrucomicrobiota	Verrucomicrobiia	Verrucomicrobiales	—	—	Verrucomicrobiales bacterium	SAMN07620217	aquatic	11	Yes	4	ATLF;Bac_DNA_binding;Abhydrola	—
2026779.SAMN07619713.PBOJ010001	**typeX**	308,1	high	Planctomycetota	Planctomycetia	Planctomycetales	Planctomycetaceae	—	Planctomycetaceae bacterium	SAMN07619713	aquatic	8	Yes	4	HTH_17;DUF1501;Sulfatase	—
2026801.SAMN10967627.DUBC010000	**typeX**	306,7	high	Verrucomicrobiota	Verrucomicrobiia	Verrucomicrobiales	—	—	Verrucomicrobiales bacterium	SAMN10967627	aquatic	11	Yes	4	Abhydrolase_7;Bac_DNA_binding;A	—
2026779.SAMN08017441.DLVN010000	**typeX**	306,6	high	Planctomycetota	Planctomycetia	Planctomycetales	Planctomycetaceae	—	Planctomycetaceae bacterium	SAMN08017441	aquatic	7	Yes	3	DRL_cat	—
2026779.SAMN07619712.PBOU010000	**typeX**	304,3	borderline	Planctomycetota	Planctomycetia	Planctomycetales	Planctomycetaceae	—	Planctomycetaceae bacterium	SAMN07619712	aquatic	7	Yes	4	GlpR;DUF1501;Sulfatase	—
2026771.SAMN14914005.JABHBR010	**typeX**	303	borderline	Verrucomicrobiota	Opitutia	—	—	—	Opitutia bacterium	SAMN14914005	aquatic	3	Yes	3	PA14;GidB;Bac_DNA_binding	—
2026779.SAMN14915429.JABJDJ0100	**typeX**	283,6	borderline	Planctomycetota	Planctomycetia	Planctomycetales	Planctomycetaceae	—	Planctomycetaceae bacterium	SAMN14915429	aquatic	10	Yes	4	DUF4231;Ferritin	—
153809.SAMEA6953877.CAINMA0100	**typeX**	237,6	low	Pseudomonadota	—	—	—	—	uncultured Pseudomonadota bacterium	SAMEA6953877	aquatic	8	Yes	4	FGE-sulfatase;Bac_DNA_binding	—
1506601.SAMEA7846325.CAJKAR010	**typeX**	224,1	low	Lentisphaerota	Lentisphaeria	—	—	—	uncultured Lentisphaeria bacterium	SAMEA7846325	host-associated	4	Yes	3	HMGL-like;CPSase_L_D2;Lipocalin	—
1506601.SAMEA7847370.CAJMAW010	**typeX**	212,7	low	Lentisphaerota	Lentisphaeria	—	—	—	uncultured Lentisphaeria bacterium	SAMEA7847370	—	10	Yes	4	NRDD;PGA_cap;N_methyl;Glycos_	—
2026780.SAMN09638790.DRQF010000	**typeX**	187,5	low	Planctomycetota	—	—	—	—	Planctomycetota bacterium	SAMN09638790	temperature	9	Yes	4	ALGX;Peptidase_M28;Lipase_GDS	—
490829.SAMN05421850.FNEB0100002	**typeX**	173	low	Pseudomonadota	Alphaproteobacteria	Rhodobacterales	Roseobacteraceae	Lutimaribacter	Lutimaribacter saemankumensis	SAMN05421850	aquatic	10	Yes	4	OPT;Hydrolase;TetR_N	—
208544.SAMEA6951753.CAIVGK0100	**typeX**	168,3	low	Pseudomonadota	Betaproteobacteria	Burkholderiales	—	—	uncultured Burkholderiales bacterium	SAMEA6951753	aquatic	2	Yes	2	rve;HTH_Tnp_1	—
1674809.SAMN03837491.LUYM010000	**typeX**	150,6	low	Actinomycetota	Actinomycetes	Actinomycetales	—	—	Actinomycetales bacterium Actino_02	SAMN03837491	host-associated	10	Yes	4	Nitro_FeMo-Co;DUF1919;AAA_15;	—
1215112.SAMD00046934.BDAI0100000	**typeXI**	317,3	high	Pseudomonadota	Gammaproteobacteria	Pseudomonadales	Pseudomonadaceae	Pseudomonas	Pseudomonas nitroreducens	SAMD00046934	host-associated	11	Yes	4	DUF3732;HEPN_RiboL-PSP;YagK	—
425022.SAMEA6956137.CAIWDD0100	**typeXI**	310,9	high	Cyanobacteriota	Cyanophyceae	Nostocales	Nodulariaceae	Nodularia	uncultured Nodularia sp.	SAMEA6956137	aquatic	11	Yes	4	Ferritin;ParB_N;T1-F;Sarcoglycan_	—
1211.SAMEA6946765.CAINGU0100000	**typeXI**	307,4	high	Cyanobacteriota	—	—	—	—	uncultured cyanobacterium	SAMEA6946765	aquatic	11	Yes	4	KIX_2;PIN_3;DUF6972	—
2024841.SAMN10967645.DUBU010000	**typeXI**	304,8	high	Bdellovibrionota	Bdellovibrionia	Bdellovibrionales	Pseudobdellovibrionace	Micavibrio	Micavibrio sp.	SAMN10967645	aquatic	7	Yes	3	Resolvase;HATPase_c_3	—
2692823.SAMN13702774.JACJPD010	**typeXI**	304,1	high	Cyanobacteriota	Cyanophyceae	Oscillatoriales	Microcoleaceae	Microcoleus	Microcoleus sp. FACHB-53	SAMN13702774	terrestrial	11	Yes	4	Acetyltransf_1;DOPA_dioxygen;RB	—
1919144.SAMN06020721.PXPI0100010	**typeXI**	303,9	high	Cyanobacteriota	—	—	—	—	Cyanobacteria bacterium SW_9_44_5	SAMN06020721	salinity	6	Yes	2	adh_short;GTP_cyclohydrol	—
32049.SAMN01081740.CP000955_26	**typeXI**	303,3	high	Cyanobacteriota	Cyanophyceae	Chroococcales	Geminocystaceae	Picosynechococcus	Picosynechococcus sp. PCC 7002	SAMN01081740	aquatic	11	Yes	4	DUF5516;Endonuclease_NS;AAA_	—
1419583.SAMN02770242.AZQQ010000	**typeXI**	302,9	high	Pseudomonadota	Gammaproteobacteria	Pseudomonadales	Pseudomonadaceae	Pseudomonas	Pseudomonas mandelii	SAMN02770242	terrestrial	11	Yes	4	Fic;DUF4238;MatE	—
1850361.SAMN05003984.LXYR010000	**typeXI**	302,1	high	Cyanobacteriota	Cyanophyceae	Leptolyngbyales	Leptolyngbyaceae	Phormidesmis	Phormidesmis priestleyi	SAMN05003984	temperature	8	Yes	4	Amidase;MazE_antitoxin;SLFN_Alb	—
1961416.SAMN06454634.DBZJ010002	**typeXI**	301,5	high	Thermodesulfobacteriota	Syntrophia	Syntrophales	Syntrophaceae	—	Syntrophaceae bacterium UBA1163	SAMN06454634	salinity	6	Yes	2	Adhesin_E;SPICE	—
2773166.SAMN13287367.JAACTD010	**typeXI**	299,6	high	Cyanobacteriota	—	—	—	—	Cyanobacteria bacterium CG_2015-16	SAMN13287367	terrestrial	11	Yes	4	YgjP-like;TUSC1;Phasin;AAA_33	—
312306.SAMN10523265.CP034337_76	**typeXI**	299,1	high	Pseudomonadota	Gammaproteobacteria	Pseudomonadales	Pseudomonadaceae	Pseudomonas	Pseudomonas entomophila	SAMN10523265	host-associated	11	Yes	4	Transposase_mut;HATPase_c_3	—
1916131.SAMN05959523.MPNC010000	**typeXI**	293,9	high	Chloroflexota	Dehalococcoidia	—	—	—	SAR202 cluster bacterium bin87	SAMN05959523	host-associated	11	Yes	3	gag-asp_proteas;CoA_transf_3;adh	—
1850361.SAMN05003984.LXYR010000	**typeXI**	292,7	high	Cyanobacteriota	Cyanophyceae	Leptolyngbyales	Leptolyngbyaceae	Phormidesmis	Phormidesmis priestleyi	SAMN05003984	temperature	11	Yes	4	AF2212-like;TPR_16;DUF4953	—
2013652.SAMN06767553.PHET010000	**typeXI**	289,5	high	Actinomycetota	—	—	—	—	Actinobacteria bacterium HGW-Actinol	SAMN06767553	terrestrial	8	Yes	4	MC8;DUF2326;MazG-like;SLFN-g3	—
2035772.SAMN19298168.JAHJHP0100	**typeXI**	287,5	high	Candidatus Omnitrophota	—	—	—	—	Candidatus Omnitrophota bacterium	SAMN19298168	terrestrial	9	Yes	4	HEPN_SAV_6107;Phage_integrase	—
1656094.SAMN05511237.MDHN01000	**typeXI**	285,9	high	Pseudomonadota	Gammaproteobacteria	Alteromonadales	Alteromonadaceae	Alteromonas	Alteromonas confluentis	SAMN05511237	aquatic	8	Yes	4	HATPase_c;dCMP_cyt_deam_1;Ca	—
1310370.SAMN15791687.JACKZK010	**typeXI**	285,4	high	Pseudomonadota	Gammaproteobacteria	Pseudomonadales	Pseudomonadaceae	Pseudomonas	Pseudomonas sp. MSSRFD41	SAMN15791687	anthropogenic	8	Yes	4	Glyco_hydro_19;Phage_integrase;H	—
195105.SAMN04279595.NIPU0100001	**typeXI**	282,5	high	Pseudomonadota	Alphaproteobacteria	Rhodobacterales	Paracoccaceae	Haematobacter	Haematobacter massiliensis	SAMN04279595	host-associated	9	Yes	4	Zn_ribbon_recom;AAA_15;UvrD-he	—
1881068.SAMN05428950.FNZB010000	**typeXI**	282,3	high	Pseudomonadota	Alphaproteobacteria	Sphingomonadales	Sphingomonadaceae	Sphingomonas	Sphingomonas sp. OV641	SAMN05428950	anthropogenic	11	Yes	4	HATPase_c;DCD;AGS_C;TniB	—
2722791.SAMN14402108.JAAVJH0100	**typeXI**	282,3	high	Pseudomonadota	Alphaproteobacteria	Sphingomonadales	Sphingomonadaceae	Sphingomonas	Sphingomonas corticis	SAMN14402108	—	11	Yes	0	—	—
481743.SAMN00191238.CP001793_26	**typeXI**	278,9	high	Bacillota	Bacilli	Bacillales	Paenibacillaceae	Paenibacillus	Paenibacillus sp. Y412MC10	SAMN00191238	host-associated	11	Yes	4	RNB;SmpB;TIR_2;nSTAND3	—
640205.SAMN05216381.FNBM010000	**typeXI**	278,6	high	Pseudomonadota	Gammaproteobacteria	Pseudomonadales	Pseudomonadaceae	Phytopseudomonas	Phytopseudomonas seleniipraecipitans	SAMN05216381	host-associated	11	Yes	4	Bflower;YobI-ATPase;Acetyltransf_	—
2583453.SAMN11812194.VCIG010000	**typeXI**	278,4	high	Pseudomonadota	Alphaproteobacteria	Hyphomicrobiales	Brucellaceae	Ochrobactrum	Ochrobactrum sp. CGA5	SAMN11812194	host-associated	11	Yes	4	Resolvase;FRG	—
367825.SAMN05904704.CP017754_18	**typeXI**	278,4	high	Pseudomonadota	Betaproteobacteria	Burkholderiales	Burkholderiaceae	Cupriavidus	Cupriavidus malaysiensis	SAMN05904704	anthropogenic	11	Yes	4	PAAR_motif;SRAP;HTH_31;T5orf1	—
1230476.SAMN01893863.KE747856_1	**typeXI**	275,8	high	Pseudomonadota	Alphaproteobacteria	Hyphomicrobiales	Nitrobacteraceae	Bradyrhizobium	Bradyrhizobium sp. DFCI-1	SAMN01893863	anthropogenic	11	Yes	4	GST_N_2;AMNp_N	—
1874826.SAMN16426229.JADJYM0100	**typeXI**	275,1	high	Pseudomonadota	Alphaproteobacteria	Sphingomonadales	Sphingomonadaceae	Novosphingobium	Novosphingobium sp.	SAMN16426229	anthropogenic	11	Yes	4	DUF7007;Trypsin_2;Trep_dent_lipo	—
1802172.SAMN04315629.MIAM010000	**typeXI**	275	high	Pseudomonadota	Alphaproteobacteria	Sphingomonadales	Sphingopyxidaceae	Sphingopyxis	Sphingopyxis sp. RIFCSPHIGHO2_12	SAMN04315629	anthropogenic	11	Yes	4	Glutaredoxin;DUF1178;KAP_NTPa	—
1913989.SAMN19297468.JAHIGR0100	**typeXI**	274,8	high	Pseudomonadota	Gammaproteobacteria	—	—	—	Gammaproteobacteria bacterium	SAMN19297468	terrestrial	10	Yes	4	Oxidored_FMN;HTH_60;NAD_bind	—
277961.SAMN11791491.VCGW010000	**typeXI**	274,6	high	Pseudomonadota	Gammaproteobacteria	Pseudomonadales	Marinobacteraceae	Marinobacter	Marinobacter maritimus	SAMN11791491	aquatic	11	Yes	4	TIR_2;PhiKZ_VTX;Thioredoxin;Tau	—
1660088.SAMN03652433.MEDN01000	**typeXI**	274,1	high	Pseudomonadota	Alphaproteobacteria	Hyphomicrobiales	Rhizobiaceae	Agrobacterium	Agrobacterium sp. SCN 61-19	SAMN03652433	anthropogenic	11	Yes	4	Pribosyltran;Dak2;APH;GCV_T	—
1903720.SAMEA5278229.CAAGCO010	**typeXI**	273,2	high	Bacillota	Bacilli	—	—	—	Bacilli bacterium	SAMEA5278229	host-associated	11	Yes	4	Thr_synth_N;Hydrolase;PLK4_bind	—
2013842.SAMN06767759.PGXO010000	**typeXI**	273,2	high	Synergistota	—	—	—	—	Synergistetes bacterium HGW-Synerg	SAMN06767759	aquatic	11	Yes	4	NOV_C;Peptidase_S24;HTH_3	—
2508294.SAMEA5537675.CABPRY010	**typeXI**	270,5	high	Pseudomonadota	Betaproteobacteria	Burkholderiales	Burkholderiaceae	Pandoraea	Pandoraea cepalis	SAMEA5537675	anthropogenic	7	Yes	3	AAA_15;GIY-YIG;AMP-binding	—
2762296.SAMN15732460.JACOGB010	**typeXI**	270	high	Pseudomonadota	Betaproteobacteria	Burkholderiales	Oxalobacteraceae	Undibacterium	Undibacterium sp. FT79W	SAMN15732460	terrestrial	11	Yes	4	Cys_rich_CPCC;TniQ	—
2775282.SAMN16237746.JACYUG010	**typeXI**	269,4	high	Pseudomonadota	Alphaproteobacteria	Sphingomonadales	Sphingomonadaceae	Sphingomonas	Sphingomonas sp. CFBP 8760	SAMN16237746	host-associated	11	Yes	4	AAA_15;DUF5131	—
1886787.SAMN11366401.SSTI0100002	**typeXI**	264,2	high	Pseudomonadota	Alphaproteobacteria	Sphingomonadales	Sphingomonadaceae	Sphingomonas	Sphingomonas olei	SAMN11366401	host-associated	8	Yes	4	MarR_2;SIR2_2;Piwi	—
409.SAMN20165186.JAIETD01000006	**typeXI**	261,2	high	Pseudomonadota	Alphaproteobacteria	Hyphomicrobiales	Methylobacteriaceae	Methylobacterium	Methylobacterium sp.	SAMN20165186	aquatic	3	Yes	2	DUF2958	—
331632.SAMEA6152175.CADBJR0100	**typeXI**	261,1	high	Actinomycetota	Coriobacteriia	Coriobacteriales	Coriobacteriaceae	—	uncultured Coriobacteriaceae bacteriu	SAMEA6152175	host-associated	6	Yes	2	FRG	—
208549.SAMEA6954897.CAIZEI010000	**typeXI**	259	high	Pseudomonadota	Alphaproteobacteria	Hyphomicrobiales	—	—	uncultured Hyphomicrobiales bacteriu	SAMEA6954897	aquatic	4	Yes	3	RNase_PH;Ham1p_like	—
458233.SAMD00060930.AP009484_36	**typeXI**	258,8	high	Bacillota	Bacilli	Bacillales	Staphylococcaceae	Macrococcoides	Macrococcoides caseolyticum	SAMD00060930	host-associated	11	Yes	4	SLFN-g3_helicase;MazG-like;Metall	—
1660160.SAMN03652429.MEDJ010000	**typeXI**	257,5	high	Acidobacteriota	—	—	—	—	Acidobacteria bacterium SCN 69-37	SAMN03652429	anthropogenic	8	Yes	4	IstB_IS21;HEPN_Swt1;Helicase_C	—
2795124.SAMN17004917.JAELUI0100	**typeXI**	253,3	high	Pseudomonadota	Betaproteobacteria	Burkholderiales	Burkholderiaceae	Ralstonia	Ralstonia sp. ASV6	SAMN17004917	host-associated	9	Yes	4	AAA;Hydrolase;HTH_Tnp_1;TnpB_	—
2282150.SAMN09639277.DSIT0100009	**typeXI**	253,2	high	Pseudomonadota	Alphaproteobacteria	Caulobacterales	—	—	Caulobacterales bacterium	SAMN09639277	temperature	11	Yes	4	TetR_C_28;UvrB_inter;TbpB_B_D	—
1961564.SAMN06450404.DING010000	**typeXI**	253,2	high	Thermodesulfobacteriota	Desulfovibrionia	Desulfovibrionales	Desulfovibrionaceae	—	Desulfovibrionaceae bacterium UBA55	SAMN06450404	terrestrial	11	Yes	4	4HBT;SIS;GATase_7;CPSase_L_D	—
1345023.SAMN02471612.ATCL010000	**typeXI**	251,3	high	Bacillota	Bacilli	Bacillales	—	Exiguobacterium	Exiguobacterium chiriqhucha	SAMN02471612	host-associated	11	Yes	4	Methyltrans_RNA;DUF4435;Peptid	—
152509.SAMEA6152703.CADCEC0100	**typeXI**	249,2	high	Bacteroidota	—	—	—	—	uncultured Bacteroidota bacterium	SAMEA6152703	host-associated	11	Yes	4	iREC;XendoU;WYL	—
1529005.SAMN04075706.LJSC010000	**typeXI**	249,2	high	Pseudomonadota	Alphaproteobacteria	Rhodobacterales	Paracoccaceae	Gemmobacter	Gemmobacter sp. LW-1	SAMN04075706	anthropogenic	11	Yes	4	Exonuc_VII_L;Arm-DNA-bind_3;Bs	—
1916129.SAMN05959503.MPMM01000	**typeXI**	247,4	high	Chloroflexota	Dehalococcoidia	—	—	—	SAR202 cluster bacterium bin22	SAMN05959503	host-associated	11	Yes	4	FRG	—
1895921.SAMN05660545.MKTC010000	**typeXI**	244	high	Bacteroidota	—	—	—	—	Bacteroidetes bacterium 37-13	SAMN05660545	anthropogenic	8	Yes	4	KilA-N;HEPN_MAE_28990;AAA_3	—

370804.SAMEA6150200.CACYME0100	typeXI	242	high	Bacteroidota	Bacteroidia	Bacteroidales	Prevotellaceae	—	uncultured Prevotellaceae bacterium	SAMEA6150200	host-associated	11	Yes	4	CGGC;HisKin-conflict;MTUS1_CCI	—
2030927.SAMN16349853.JAGBQJ0100	typeXI	240,6	high	Bacteroidota	Bacteroidia	Bacteroidales	—	—	Bacteroidales bacterium	SAMN16349853	host-associated	8	Yes	4	WYL;YhcG_C;WG_beta_rep	—
196367.SAMN06075480.MTHB010000	typeXI	240,6	high	Pseudomonadota	Betaproteobacteria	Burkholderiales	Burkholderiaceae	Caballeronia	Caballeronia sordidicola	SAMN06075480	terrestrial	7	Yes	3	DUF6933;Trypsin_2;Phage_integra	—
2030927.SAMN16347308.JAFXXO0100	typeXI	239,9	high	Bacteroidota	Bacteroidia	Bacteroidales	—	—	Bacteroidales bacterium	SAMN16347308	host-associated	11	Yes	4	WYL;HisKin-conflict;Tenui_NCP	—
286133.SAMEA5852096.CABMQD0100	typeXI	239,5	high	Pseudomonadota	Betaproteobacteria	Burkholderiales	Sutterellaceae	Sutterella	uncultured Sutterella sp.	SAMEA5852096	host-associated	11	Yes	4	MrcB_N;ATPase_2;DUF927	—
1129257.SAMEA6152766.CADCGK010	typeXI	237,8	high	Bacteroidota	Bacteroidia	—	—	—	uncultured Bacteroidia bacterium	SAMEA6152766	host-associated	11	Yes	4	HD;SACS	—
2030927.SAMN16349994.JAGBVU0100	typeXI	237,3	high	Bacteroidota	Bacteroidia	Bacteroidales	—	—	Bacteroidales bacterium	SAMN16349994	host-associated	11	Yes	4	GerT_N;DUF7002;Beta-prop_CAF1	—
1916129.SAMN05959503.MPMM01000	typeXI	236,1	high	Chloroflexota	Dehalococcoidia	—	—	—	SAR202 cluster bacterium bin22	SAMN05959503	host-associated	11	Yes	4	Phage_integrase;HNH;FRG;DUF69	—
2748031.SAMN16348336.JAFZLC0100	typeXI	235,8	high	Bacteroidota	Bacteroidia	Bacteroidales	Paludibacteraceae	—	Paludibacteraceae bacterium	SAMN16348336	host-associated	8	Yes	4	Pribosyltran;DNA_processg_A;WHI	—
152509.SAMEA6150143.CACYJU0100	typeXI	235,6	high	Bacteroidota	—	—	—	—	uncultured Bacteroidota bacterium	SAMEA6150143	host-associated	9	Yes	4	DnaB_C;T1-F;DUF2846;WYL	—
2030927.SAMN16347413.JAFYBP0100	typeXI	235,2	high	Bacteroidota	Bacteroidia	Bacteroidales	—	—	Bacteroidales bacterium	SAMN16347413	host-associated	9	Yes	4	Sulfotransfer_1;DnaB_C	—
59823.SAMN19224964.DXTA01000038	typeXI	235	high	Bacteroidota	Bacteroidia	Bacteroidales	Prevotellaceae	Prevotella	Prevotella sp.	SAMN19224964	host-associated	8	Yes	4	iREC;HisKin-conflict;SufE	—
152509.SAMEA6151326.CADADB0100	typeXI	234,7	high	Bacteroidota	—	—	—	—	uncultured Bacteroidota bacterium	SAMEA6151326	host-associated	11	Yes	4	GVIN1_C;WYL;T1-F;Cyano_EgtBD	—
2026756.SAMN11294391.SUWU01000	typeXI	234,6	high	Bacteroidota	Bacteroidia	Bacteroidales	Lentimicrobiaceae	—	Lentimicrobiaceae bacterium	SAMN11294391	host-associated	10	Yes	4	WYL;Asp_protease_2;T1-F	—
194843.SAMEA6150145.CACYJZ0100	typeXI	233,4	high	Bacteroidota	Bacteroidia	Bacteroidales	—	—	uncultured Bacteroidales bacterium	SAMEA6150145	host-associated	11	Yes	4	WYL;HisKin-conflict;WHD_eIF2D	—
754429.SAMN06133352.CP019333_12	typeXI	232,7	low	Bacteroidota	Flavobacteriia	Flavobacteriales	Flavobacteriaceae	Gilvibacter	Gilvibacter sp. SZ-19	SAMN06133352	temperature	10	Yes	4	AAA_30;Seryl_tRNA_N;MRP-S26	—
59823.SAMN11294429.SUYG01000053	typeXI	232,6	low	Bacteroidota	Bacteroidia	Bacteroidales	Prevotellaceae	Prevotella	Prevotella sp.	SAMN11294429	host-associated	10	Yes	4	Plasmid_parti_N;CGGC;T1-F;EMY	—
2030927.SAMN16341878.JAFPVK0100	typeXI	232,6	low	Bacteroidota	Bacteroidia	Bacteroidales	—	—	Bacteroidales bacterium	SAMN16341878	host-associated	10	Yes	4	iREC;WHD_eIF2D;WG_beta_rep	—
2049048.SAMN16344483.JAFTOX0100	typeXI	231,9	low	Bacteroidota	Bacteroidia	Bacteroidales	Rikenellaceae	—	Rikenellaceae bacterium	SAMN16344483	host-associated	10	Yes	4	AAA_30;WYL;Seryl_tRNA_N	—
2030927.SAMN16343841.JAFSQF0100	typeXI	231,2	low	Bacteroidota	Bacteroidia	Bacteroidales	—	—	Bacteroidales bacterium	SAMN16343841	host-associated	10	Yes	4	DnaJ;DnaB_C;Bflower_2	—
2030927.SAMN19225073.JAHZEX0100	typeXI	230,9	low	Bacteroidota	Bacteroidia	Bacteroidales	—	—	Bacteroidales bacterium	SAMN19225073	host-associated	10	Yes	4	YkzI;Seryl_tRNA_N;WYL;TM225	—
59823.SAMN16343164.JAFRQB01000	typeXI	230,8	low	Bacteroidota	Bacteroidia	Bacteroidales	Prevotellaceae	Prevotella	Prevotella sp.	SAMN16343164	host-associated	10	Yes	4	PLDc_2;CGGC;AAA_30	—
1236515.SAMD00010076.BAKM01000	typeXI	230,7	low	Bacteroidota	Bacteroidia	Bacteroidales	Bacteroidaceae	Phocaeicola	Phocaeicola sartorii	SAMD00010076	host-associated	10	Yes	4	DUF1972;YjbH;MTUS1_CCDC69_	—
1129257.SAMEA6152677.CADCCZ010	typeXI	229,7	low	Bacteroidota	Bacteroidia	—	—	—	uncultured Bacteroidia bacterium	SAMEA6152677	host-associated	10	Yes	4	iREC;MTUS1_CCDC69_CC1;Asp_	—
54062.SAMD00166260.BJWE0100000	typeXI	229,5	low	Bacillota	Bacilli	Lactobacillales	Lactobacillaceae	Pediococcus	Pediococcus parvulus	SAMD00166260	anthropogenic	10	Yes	4	rve;HTH_28;VanZ;DUF2715	—
370804.SAMEA6151525.CADAKX0100	typeXI	229,1	low	Bacteroidota	Bacteroidia	Bacteroidales	Prevotellaceae	—	uncultured Prevotellaceae bacterium	SAMEA6151525	host-associated	7	Yes	4	CGGC;PLDc_2	—
194843.SAMEA6150768.CACZHT0100	typeXI	228,9	low	Bacteroidota	Bacteroidia	Bacteroidales	—	—	uncultured Bacteroidales bacterium	SAMEA6150768	host-associated	10	Yes	4	Cytochrome_C7;WG_beta_rep;WY	—
1952463.SAMN06456358.DDQG01000	typeXI	228,9	low	Bacteroidota	Bacteroidia	Bacteroidales	Prevotellaceae	—	Prevotellaceae bacterium UBA2448	SAMN06456358	host-associated	10	Yes	4	AAA_30;DUF7002;DUF5496;YhcG	—
370804.SAMEA6150222.CACYNE0100	typeXI	228,8	low	Bacteroidota	Bacteroidia	Bacteroidales	Prevotellaceae	—	uncultured Prevotellaceae bacterium	SAMEA6150222	host-associated	10	Yes	4	SufE;HisKin-conflict;iREC	—
1704277.SAMN04002371.LIYN020000	typeXI	228,8	low	Deinococcota	Deinococci	Deinococcales	Deinococcaceae	Deinococcus	Deinococcus sp. UR1	SAMN04002371	terrestrial	10	Yes	4	AIRS_C;GATase_7;DUF2196	—
370804.SAMEA6149422.CACXIP01000	typeXI	228,6	low	Bacteroidota	Bacteroidia	Bacteroidales	Prevotellaceae	—	uncultured Prevotellaceae bacterium	SAMEA6149422	host-associated	10	Yes	4	Beta-prop_IFT122_1st;WYL;HisKin	—
2079531.SAMEA104588899.LT985324_	typeXI	228,3	low	Bacteroidota	Bacteroidia	Bacteroidales	Prevotellaceae	Prevotella	Prevotella merdae	SAMEA104588899	host-associated	10	Yes	4	PIN;iREC;Ribosomal_L27	—
370804.SAMEA6152573.CADBYY0100	typeXI	228	low	Bacteroidota	Bacteroidia	Bacteroidales	Prevotellaceae	—	uncultured Prevotellaceae bacterium	SAMEA6152573	host-associated	10	Yes	4	AAA_30;PLDc_2	—
1129257.SAMEA6151604.CADANY010	typeXI	227,8	low	Bacteroidota	Bacteroidia	—	—	—	uncultured Bacteroidia bacterium	SAMEA6151604	host-associated	9	Yes	4	MreB_Mbl;Orthopox_A43R;DUF42	—
2030927.SAMN16347328.JAFXYI0100	typeXI	227,6	low	Bacteroidota	Bacteroidia	Bacteroidales	—	—	Bacteroidales bacterium	SAMN16347328	host-associated	10	Yes	4	iREC;HisKin-conflict;WYL;HATPase	—
1929085.SAMN16350086.JAGBZI0100	typeXI	227,3	low	Bacteroidota	Bacteroidia	Bacteroidales	Rikenellaceae	Tidjanibacter	Tidjanibacter sp.	SAMN16350086	host-associated	6	Yes	3	LRR_5;FTHFS;CE2_N	—
159272.SAMEA7847924.CAJMCE0100	typeXI	227	low	Bacteroidota	Bacteroidia	Bacteroidales	Prevotellaceae	Prevotella	uncultured Prevotella sp.	SAMEA7847924	host-associated	8	Yes	4	T1-F;WYL	—
1129257.SAMEA6148842.CACWMH01	typeXI	226,5	low	Bacteroidota	Bacteroidia	—	—	—	uncultured Bacteroidia bacterium	SAMEA6148842	host-associated	10	Yes	4	AAA_11;T1-F;WG_beta_rep;WYL	—
194843.SAMEA6151427.CADAGY0100	typeXI	226,3	low	Bacteroidota	Bacteroidia	Bacteroidales	—	—	uncultured Bacteroidales bacterium	SAMEA6151427	host-associated	10	Yes	4	MTUS1_CCDC69_CC1;HisKin-cor	—
1929085.SAMN16344624.JAFTUI0100	typeXI	226,1	low	Bacteroidota	Bacteroidia	Bacteroidales	Rikenellaceae	Tidjanibacter	Tidjanibacter sp.	SAMN16344624	host-associated	10	Yes	4	DUF6261;T1-F;MIT_C;AAA_15	—
370804.SAMEA6150912.CACZNL0100	typeXI	226,1	low	Bacteroidota	Bacteroidia	Bacteroidales	Prevotellaceae	—	uncultured Prevotellaceae bacterium	SAMEA6150912	host-associated	10	Yes	0	—	—
2212467.SAMN16349935.JAGBTN0100	typeXI	226	low	Bacteroidota	Bacteroidia	Bacteroidales	Bacteroidaceae	—	Bacteroidaceae bacterium	SAMN16349935	host-associated	10	Yes	4	WG_beta_rep;T1-F	—
194843.SAMEA6149915.CACYBD0100	typeXI	225,7	low	Bacteroidota	Bacteroidia	Bacteroidales	—	—	uncultured Bacteroidales bacterium	SAMEA6149915	host-associated	10	Yes	4	PLDc_2;CGGC	—
2486470.SAMN10365287.RIBI0100000	typeXI	225,6	low	Bacteroidota	Bacteroidia	Bacteroidales	Muribaculaceae	—	Muribaculaceae bacterium Isolate-102	SAMN10365287	host-associated	10	Yes	4	WYL;T1-F;HAD_2	—
2030927.SAMN13893983.JAAYJQ0100	typeXI	225,3	low	Bacteroidota	Bacteroidia	Bacteroidales	—	—	Bacteroidales bacterium	SAMN13893983	temperature	10	Yes	4	WYL;T1-F;DUF3695	—
194843.SAMEA6150745.CACZHD0100	typeXI	224,7	low	Bacteroidota	Bacteroidia	Bacteroidales	—	—	uncultured Bacteroidales bacterium	SAMEA6150745	host-associated	10	Yes	4	WYL;HisKin-conflict;iREC	—
2212467.SAMN16346420.JAFWPN010	typeXI	224,7	low	Bacteroidota	Bacteroidia	Bacteroidales	Bacteroidaceae	—	Bacteroidaceae bacterium	SAMN16346420	host-associated	10	Yes	4	YhcG_C;Plasmid_parti_N;DUF7002	—
2301481.SAMEA8805426.CAJUAJ0100	typeXI	224,6	low	Bacteroidota	Bacteroidia	Bacteroidales	Muribaculaceae	—	uncultured Muribaculaceae bacterium	SAMEA8805426	host-associated	10	Yes	4	BAR_4;HisKin-conflict;Por_Secre_t	—
194843.SAMEA6150050.CACYGD0100	typeXI	224,3	low	Bacteroidota	Bacteroidia	Bacteroidales	—	—	uncultured Bacteroidales bacterium	SAMEA6150050	host-associated	10	Yes	4	WYL;WG_beta_rep;T1-F;LRR_5	—
194843.SAMEA6150348.CACYRT0100	typeXI	224,3	low	Bacteroidota	Bacteroidia	Bacteroidales	—	—	uncultured Bacteroidales bacterium	SAMEA6150348	host-associated	10	Yes	4	iREC;HisKin-conflict;WYL;GP57	—
2133944.SAMN08763369.CP033459_2	typeXI	224	low	Bacteroidota	Bacteroidia	Bacteroidales	Prevotellaceae	Pseudoprevotella	Pseudoprevotella muciniphila	SAMN08763369	host-associated	10	Yes	4	HisKin-conflict;iREC	—
2212467.SAMN16243600.JAEELS0100	typeXI	223,4	low	Bacteroidota	Bacteroidia	Bacteroidales	Bacteroidaceae	—	Bacteroidaceae bacterium	SAMN16243600	host-associated	10	Yes	4	DUF5496;CGGC;DUF1848	—
370804.SAMEA6150573.CACZAB0100	typeXI	223,2	low	Bacteroidota	Bacteroidia	Bacteroidales	Prevotellaceae	—	uncultured Prevotellaceae bacterium	SAMEA6150573	host-associated	10	Yes	4	UvrD-helicase;LRR_5	—
2030927.SAMN16243923.JAEEXD0100	typeXI	223,1	low	Bacteroidota	Bacteroidia	Bacteroidales	—	—	Bacteroidales bacterium	SAMN16243923	host-associated	10	Yes	4	SLFN_AlbA_2;DNA_processg_A	—
194843.SAMEA6152128.CADBHU0100	typeXI	223,1	low	Bacteroidota	Bacteroidia	Bacteroidales	—	—	uncultured Bacteroidales bacterium	SAMEA6152128	host-associated	10	Yes	4	AMP_deaminase	—
370804.SAMEA6151246.CADAAG0100	typeXI	222,2	low	Bacteroidota	Bacteroidia	Bacteroidales	Prevotellaceae	—	uncultured Prevotellaceae bacterium	SAMEA6151246	host-associated	10	Yes	4	iREC;HisKin-conflict;WYL;DUF701	—
152509.SAMEA6954497.CAINMV0100	typeXI	222,1	low	Bacteroidota	—	—	—	—	uncultured Bacteroidota bacterium	SAMEA6954497	temperature	10	Yes	4	Por_Secre_tail;DnaB_C;VWA_2	—
2498093.SAMN16342757.JAFQZK0100	typeXI	222	low	Bacteroidota	Bacteroidia	Bacteroidales	Muribaculaceae	—	Muribaculaceae bacterium	SAMN16342757	host-associated	10	Yes	4	WYL;T1-F;HAD_2	—
152509.SAMEA6954080.CAIWCG0100	typeXI	221,8	low	Bacteroidota	—	—	—	—	uncultured Bacteroidota bacterium	SAMEA6954080	aquatic	10	Yes	4	UvrD_C;VWA_2;DnaB_C;AAA	—
2033407.SAMN14407237.JAAVFD0100	typeXI	221,6	low	Bacteroidota	Bacteroidia	Bacteroidales	Barnesiellaceae	Barnesiella	Barnesiella sp.	SAMN14407237	host-associated	10	Yes	4	BAR_4;HisKin-conflict;Glyco_hydro	—
2212467.SAMN16243652.JAEENS0100	typeXI	221,5	low	Bacteroidota	Bacteroidia	Bacteroidales	Bacteroidaceae	—	Bacteroidaceae bacterium	SAMN16243652	host-associated	10	Yes	4	YhcG_C;Beta-prop_IFT122_1st;DU	—
152509.SAMEA6953637.CAIUSH0100	typeXI	221,5	low	Bacteroidota	—	—	—	—	uncultured Bacteroidota bacterium	SAMEA6953637	aquatic	3	Yes	3	iREC;HisKin-conflict;WYL	—
370804.SAMEA104667055.ONMA0100	typeXI	221,5	low	Bacteroidota	Bacteroidia	Bacteroidales	Prevotellaceae	—	uncultured Prevotellaceae bacterium	SAMEA104667055	host-associated	10	Yes	4	WYL;WG_beta_rep;HisKin-conflict;	—
1841864.SAMN14913643.JACJJG0100	typeXI	221,3	low	Bacteroidota	Bacteroidia	Bacteroidales	Prevotellaceae	Marseilla	Marseilla massiliensis	SAMN14913643	host-associated	10	Yes	4	WYL;WG_beta_rep;Seryl_tRNA_N;	—
2030927.SAMN16346686.JAFWZP010	typeXI	221,2	low	Bacteroidota	Bacteroidia	Bacteroidales	—	—	Bacteroidales bacterium	SAMN16346686	host-associated	8	Yes	4	WG_beta_rep;T1-F;TPR_7	—
194843.SAMEA6152634.CADCBN0100	typeXI	221	low	Bacteroidota	Bacteroidia	Bacteroidales	—	—	uncultured Bacteroidales bacterium	SAMEA6152634	host-associated	10	Yes	4	CarbopepD_reg_2;DUF7002;DuOB	—
194843.SAMEA6150745.CACZHD0100	typeXI	220,5	low	Bacteroidota	Bacteroidia	Bacteroidales	—	—	uncultured Bacteroidales bacterium	SAMEA6150745	host-associated	10	Yes	4	LprI;WYL	—
2030927.SAMEA5279175.CAAFXO010	typeXI	220,4	low	Bacteroidota	Bacteroidia	Bacteroidales	—	—	Bacteroidales bacterium	SAMEA5279175	host-associated	10	Yes	4	HisKin-conflict;iREC	—
159274.SAMEA5278472.CAAEQB0100	typeXI	220,3	low	Bacteroidota	Bacteroidia	Bacteroidales	Porphyromonadaceae	Porphyromonas	uncultured Porphyromonas sp.	SAMEA5278472	host-associated	7	Yes	4	PepSY_like;WYL	—
159272.SAMEA104666937.OMZM0100	typeXI	220,3	low	Bacteroidota	Bacteroidia	Bacteroidales	Prevotellaceae	Prevotella	uncultured Prevotella sp.	SAMEA104666937	host-associated	10	Yes	4	WG_beta_rep;Pox_E6;AA_kinase	—
348578.SAMEA7847109.CAJLTU0100	typeXI	220,3	low	Bacteroidota	Bacteroidia	Bacteroidales	Porphyromonadaceae	—	uncultured Porphyromonadaceae bact	SAMEA7847109	host-associated	10	Yes	4	ResIII;HTH_17;HisKin-conflict;BAR_	—
1898104.SAMN16426341.JADKCP010	typeXI	220,2	low	Bacteroidota	—	—	—	—	Bacteroidota bacterium	SAMN16426341	anthropogenic	10	Yes	4	HTH_31;DnaB_C	—
370804.SAMEA6151724.CADASP0100	typeXI	220,1	low	Bacteroidota	Bacteroidia	Bacteroidales	Prevotellaceae	—	uncultured Prevotellaceae bacterium	SAMEA6151724	host-associated	5	Yes	2	LRR_5	—
348578.SAMEA4891951.URTA010000	typeXI	219,6	low	Bacteroidota	Bacteroidia	Bacteroidales	Porphyromonadaceae	—	uncultured Porphyromonadaceae bact	SAMEA4891951	host-associated	10	Yes	4	Hydrolase_4;Seryl_tRNA_N;WG_b	—
2301481.SAMEA8805709.CAJULY0100	typeXI	219,4	low	Bacteroidota	Bacteroidia	Bacteroidales	Muribaculaceae	—	uncultured Muribaculaceae bacterium	SAMEA8805709	host-associated	10	Yes	4	BAR_4;HisKin-conflict;HTH_17;DU	—
2030927.SAMN16344869.JAFUDT010	typeXI	219,4	low	Bacteroidota	Bacteroidia	Bacteroidales	—	—	Bacteroidales bacterium	SAMN16344869	host-associated	10	Yes	4	CdsD_C;WYL;T1-F	—
1872444.SAMN16349454.JAGBAX010	typeXI	219,4	low	Bacteroidota	Bacteroidia	Bacteroidales	Rikenellaceae	Alistipes	Alistipes sp.	SAMN16349454	host-associated	10	Yes	4	WYL;T1-F	—
159272.SAMEA7848212.CAJMJX0100	typeXI	219,3	low	Bacteroidota	Bacteroidia	Bacteroidales	Prevotellaceae	Prevotella	uncultured Prevotella sp.	SAMEA7848212	host-associated	10	Yes	4	WYL;WG_beta_rep;Aconitase_C	—
194843.SAMEA6150745.CACZHD0100	typeXI	219,3	low	Bacteroidota	Bacteroidia	Bacteroidales	—	—	uncultured Bacteroidales bacterium	SAMEA6150745	host-associated	10	Yes	4	WYL;Baculo_p74	—

2498093.SAMN16349242.JAGAST0100	**typeXI**	219,1	low	Bacteroidota	Bacteroidia	Bacteroidales	Muribaculaceae	—	Muribaculaceae bacterium	SAMN16349242	host-associated	10	Yes	4	WYL	—
2840839.SAMN15816954.JADIMG0100	**typeXI**	218,6	low	Bacteroidota	Bacteroidia	Bacteroidales	—	Candidatus Gallipaludi	Candidatus Gallipaludibacter merdaviu	SAMN15816954	host-associated	10	Yes	4	LysM	—
2053307.SAMN16091041.JADQBJ010	**typeXI**	218,5	low	Thermodesulfobacteriota	Desulfobulbia	Desulfobulbales	Desulfobulbaceae	—	Desulfobulbaceae bacterium	SAMN16091041	aquatic	10	Yes	4	SmpB;Phage_int_M;Response_reg	—
2030927.SAMN18120043.JAGOLH010	**typeXI**	218,5	low	Bacteroidota	Bacteroidia	Bacteroidales	—	—	Bacteroidales bacterium	SAMN18120043	anthropogenic	8	Yes	4	Glyco_hydro_25;DnaB_C;Por_Secr	—
194843.SAMEA6150559.CACYZT0100	**typeXI**	218,3	low	Bacteroidota	Bacteroidia	Bacteroidales	—	—	uncultured Bacteroidales bacterium	SAMEA6150559	host-associated	10	Yes	4	DUF488;DuOB;T1-F;Feld-I_B	—
1898104.SAMN17573936.JAFEAA0100	**typeXI**	218,2	low	Bacteroidota	—	—	—	—	Bacteroidota bacterium	SAMN17573936	anthropogenic	10	Yes	4	WYL;DUF3127	—
361581.SAMN08779091.RCCS010000	**typeXI**	218,2	low	Bacteroidota	Flavobacteriia	Flavobacteriales	Flavobacteriaceae	Tenacibaculum	Tenacibaculum discolor	SAMN08779091	aquatic	10	Yes	4	HTH_18;NHL;Leu_Phe_trans;SCF	—
246.SAMN10316127.CP033935_66	**typeXI**	218,1	low	Bacteroidota	Flavobacteriia	Flavobacteriales	Weeksellaceae	Chryseobacterium	Chryseobacterium balustinum	SAMN10316127	aquatic	10	Yes	4	BAR_4;HisKin-conflict;ResIII;VapC	—
159272.SAMEA7846766.CAJLGE0100	**typeXI**	217,6	low	Bacteroidota	Bacteroidia	Bacteroidales	Prevotellaceae	Prevotella	uncultured Prevotella sp.	SAMEA7846766	host-associated	6	Yes	3	WYL;WG_beta_rep;Seryl_tRNA_N	—
2678657.SAMN13336490.WOFO01000	**typeXI**	216,8	low	Bacteroidota	Cytophagia	Cytophagales	Cyclobacteriaceae	Cyclobacterium	Cyclobacterium sp. SYSU L10401	SAMN13336490	salinity	10	Yes	4	iREC;HisKin-conflict;WYL;Peptidas	—
152509.SAMEA6953983.CAINDA0100	**typeXI**	216,7	low	Bacteroidota	—	—	—	—	uncultured Bacteroidota bacterium	SAMEA6953983	aquatic	10	Yes	4	YjdM;Seryl_tRNA_N;E2-CBASS	—
152509.SAMEA6955963.CAITNS01000	**typeXI**	216,6	low	Bacteroidota	—	—	—	—	uncultured Bacteroidota bacterium	SAMEA6955963	aquatic	5	Yes	2	IL15;WYL	—
194843.SAMEA6951536.CAIYES01000	**typeXI**	216,5	low	Bacteroidota	Bacteroidia	Bacteroidales	—	—	uncultured Bacteroidales bacterium	SAMEA6951536	aquatic	7	Yes	4	Response_reg;DUF3127;WYL	—
152509.SAMEA6951679.CAIKXD01000	**typeXI**	216,2	low	Bacteroidota	—	—	—	—	uncultured Bacteroidota bacterium	SAMEA6951679	aquatic	3	Yes	2	Methyltransf_23;2OG-FeII_Oxy_2	—
1284775.SAMN02850936.JRNC010001	**typeXI**	215,7	low	Bacteroidota	Bacteroidia	Bacteroidales	Prevotellaceae	Prevotella	Prevotella sp. S7-1-8	SAMN02850936	host-associated	8	Yes	4	UvrD_C_2;SufE;Seryl_tRNA_N;SL	—
1898104.SAMN20441989.JAIFNN0100	**typeXI**	215	low	Bacteroidota	—	—	—	—	Bacteroidota bacterium	SAMN20441989	aquatic	5	Yes	2	DnaB_C;Y1_Tnp	—
2030927.SAMN18118926.JAGNBV010	**typeXI**	215	low	Bacteroidota	Bacteroidia	Bacteroidales	—	—	Bacteroidales bacterium	SAMN18118926	anthropogenic	7	Yes	4	Claudin_TMEM179-179B;Peptidas	—
194843.SAMEA6151813.CADAWB010	**typeXI**	214,8	low	Bacteroidota	Bacteroidia	Bacteroidales	—	—	uncultured Bacteroidales bacterium	SAMEA6151813	host-associated	10	Yes	4	UvrD_C_2;SufE	—
2448779.SAMN18120629.JAGPDS010	**typeXI**	214,8	low	Bacteroidota	Chitinophagia	Chitinophagales	—	—	Chitinophagales bacterium	SAMN18120629	anthropogenic	6	Yes	3	DUF2325;WYL	—
2202734.SAMN16426033.JADJRO010	**typeXI**	214,8	low	Bacteroidota	Saprospiria	Saprospirales	Saprospiraceae	—	Saprospiraceae bacterium	SAMN16426033	anthropogenic	10	Yes	4	DUF7874;E2-CBASS;WYL	—
213322.SAMEA6944250.CAJAII010000	**typeXI**	214,6	low	Bacteroidota	Flavobacteriia	Flavobacteriales	—	—	uncultured Flavobacteriales bacterium	SAMEA6944250	temperature	10	Yes	4	PHM7_cyt;DnaB_C;Hydrolase_3;Si	—
212695.SAMEA6944788.CAIXMS0100	**typeXI**	214,3	low	Bacteroidota	Flavobacteriia	—	—	—	uncultured Flavobacteriia bacterium	SAMEA6944788	temperature	10	Yes	4	HTH_11;Malic_M;DnaB_C;DNA_pr	—
2748031.SAMN16347797.JAFYQJ0100	**typeXI**	214	low	Bacteroidota	Bacteroidia	Bacteroidales	Paludibacteraceae	—	Paludibacteraceae bacterium	SAMN16347797	host-associated	10	Yes	4	HAD_2;Aminotran_1_2;cREC_REC	—
2740583.SAMN15052362.JABUMY010	**typeXI**	213,8	low	Bacteroidota	Chitinophagia	Chitinophagales	Chitinophagaceae	Flavisolibacter	Flavisolibacter longurius	SAMN15052362	host-associated	6	Yes	3	Response_reg;WYL	—
152509.SAMEA6948151.CAJAEO0100	**typeXI**	213,8	low	Bacteroidota	—	—	—	—	uncultured Bacteroidota bacterium	SAMEA6948151	aquatic	10	Yes	4	iREC;HisKin-conflict;WYL;DUF671	—
2498093.SAMN16345734.JAFVPD010	**typeXI**	213,7	low	Bacteroidota	Bacteroidia	Bacteroidales	Muribaculaceae	—	Muribaculaceae bacterium	SAMN16345734	host-associated	10	Yes	4	CstA;PHP;HTS;AAA_lid_6	—
159272.SAMEA8030440.CAJOPE0100	**typeXI**	213,6	low	Bacteroidota	Bacteroidia	Bacteroidales	Prevotellaceae	Prevotella	uncultured Prevotella sp.	SAMEA8030440	host-associated	10	Yes	4	DUF7632;Orf78;DUF8000;TPK_B1	—
667015.SAMN00713589.CP002530_21	**typeXI**	213,1	low	Bacteroidota	Bacteroidia	Bacteroidales	Bacteroidaceae	Phocaeicola	Phocaeicola salanitronis	SAMN00713589	host-associated	10	Yes	4	DNA_processg_A	—
1122985.SAMN02441341.KB899220_5	**typeXI**	212,2	low	Bacteroidota	Bacteroidia	Bacteroidales	Prevotellaceae	Hoylesella	Hoylesella loescheii	SAMN02441341	host-associated	10	Yes	4	Exonuc_VII_L;Exonuc_VII_S;RuvA	—
2030927.SAMN13893767.JAAYFR0100	**typeXI**	212,1	low	Bacteroidota	Bacteroidia	Bacteroidales	—	—	Bacteroidales bacterium	SAMN13893767	anthropogenic	5	Yes	3	WYL;DUF2325;iREC	—
2044603.SAMN07768642.PEAZ010000	**typeXI**	212	low	Bacteroidota	Sphingobacteriia	Sphingobacteriales	Sphingobacteriaceae	Sphingobacterium	Sphingobacterium sp. 1.A.4	SAMN07768642	host-associated	10	Yes	4	HXXEE;WYL;Yop-YscD_ppl_3rd;A	—
1898104.SAMN17573936.JAFEAA0100	**typeXI**	211,9	low	Bacteroidota	—	—	—	—	Bacteroidota bacterium	SAMN17573936	anthropogenic	10	Yes	4	Ribosomal_L17;DnaB_C;ArlS_N;D	—
702447.SAMN00007489.ADKP0100012	**typeXI**	211,8	low	Bacteroidota	Bacteroidia	Bacteroidales	Bacteroidaceae	Bacteroides	Bacteroides xylanisolvens	SAMN00007489	host-associated	10	Yes	4	iREC;PucR;Phage_integrase;NusG	—
1852808.SAMN05004131.MWCX01000	**typeXI**	211,7	low	Bacteroidota	—	—	—	—	Bacteroidetes bacterium ADurb.Bin17	SAMN05004131	—	5	Yes	0	—	—
1898104.SAMN10966580.VGMK01000	**typeXI**	211,6	low	Bacteroidota	—	—	—	—	Bacteroidota bacterium	SAMN10966580	aquatic	6	Yes	3	DUF7874;DUF6717	—
159272.SAMEA8030157.CAJOEC0100	**typeXI**	211,3	low	Bacteroidota	Bacteroidia	Bacteroidales	Prevotellaceae	Prevotella	uncultured Prevotella sp.	SAMEA8030157	host-associated	10	Yes	4	WDR55;Seryl_tRNA_N	—
162156.SAMEA7847592.CAJLYR0100	**typeXI**	210,7	low	Bacteroidota	Bacteroidia	Bacteroidales	Bacteroidaceae	Bacteroides	uncultured Bacteroides sp.	SAMEA7847592	host-associated	10	Yes	4	Tricorn_N;DNA_processg_A	—
213322.SAMEA6950112.CAJAVU0100	**typeXI**	210,5	low	Bacteroidota	Flavobacteriia	Flavobacteriales	—	—	uncultured Flavobacteriales bacterium	SAMEA6950112	aquatic	9	Yes	4	DAGAT;DnaB_C;Parathyroid;Lamin	—
1797353.SAMN04313814.MEOQ01000	**typeXI**	210,4	low	Bacteroidota	—	—	—	—	Bacteroidetes bacterium GWF2_43_6	SAMN04313814	terrestrial	10	Yes	4	DuOB;WYL;HisKin-conflict;WHD_e	—
2030927.SAMN16056124.JAHDPK010	**typeXI**	209,9	low	Bacteroidota	Bacteroidia	Bacteroidales	—	—	Bacteroidales bacterium	SAMN16056124	anthropogenic	9	Yes	4	HisKin-conflict;WYL;Transposase_r	—
2301481.SAMEA7847604.CAJKVR010	**typeXI**	209,8	low	Bacteroidota	Bacteroidia	Bacteroidales	Muribaculaceae	—	uncultured Muribaculaceae bacterium	SAMEA7847604	host-associated	9	Yes	4	tRNA-synt_2;Seryl_tRNA_N;DNA_	—
1423816.SAMD00036561.BACQ01000	**typeXI**	209,5	low	Bacillota	Bacilli	Lactobacillales	Lactobacillaceae	Lacticaseibacillus	Lacticaseibacillus zeae	SAMD00036561	host-associated	10	Yes	4	MMR_HSR1;Trypsin_2;DUF2568;	—
152509.SAMEA6954493.CAIYNF01000	**typeXI**	209,5	low	Bacteroidota	—	—	—	—	uncultured Bacteroidota bacterium	SAMEA6954493	aquatic	6	Yes	3	Nramp;T2SS-T3SS_pil_N;DnaB_C	—
1898104.SAMN16425514.JADIYW0100	**typeXI**	209,3	low	Bacteroidota	—	—	—	—	Bacteroidota bacterium	SAMN16425514	—	6	Yes	0	—	—
2202734.SAMN16425904.JADJNH010	**typeXI**	209,2	low	Bacteroidota	Saprospiria	Saprospirales	Saprospiraceae	—	Saprospiraceae bacterium	SAMN16425904	anthropogenic	10	Yes	4	iREC;HisKin-conflict;WYL;TerC	—
2448779.SAMN18119813.JAGNZZ010	**typeXI**	209,2	low	Bacteroidota	Chitinophagia	Chitinophagales	—	—	Chitinophagales bacterium	SAMN18119813	anthropogenic	1	Yes	1	DnaB_C	—
1974047.SAMN06659620.PCUD01000	**typeXI**	208,9	low	Ignavibacteriota	Ignavibacteria	Ignavibacteriales	—	—	Ignavibacteriales bacterium CG18_big	SAMN06659620	terrestrial	7	Yes	4	DEAD;DUF2225;GIDA_C_1st	—
2053306.SAMN09639373.DSML01000	**typeXI**	208,6	low	Ignavibacteriota	Ignavibacteria	—	—	—	Ignavibacteria bacterium	SAMN09639373	temperature	10	Yes	4	GHL10;WYL;POTRA_3;iREC	—
880074.SAMN02769649.CP007034_23	**typeXI**	208,5	low	Bacteroidota	Bacteroidia	Bacteroidales	Barnesiellaceae	Barnesiella	Barnesiella viscericola	SAMN02769649	host-associated	10	Yes	4	DpnD-PcfM;HisKin-conflict;WHD_e	—
194843.SAMEA6945217.CAIOAD0100	**typeXI**	208,4	low	Bacteroidota	Bacteroidia	Bacteroidales	—	—	uncultured Bacteroidales bacterium	SAMEA6945217	aquatic	3	Yes	3	DnaB_C	—
575615.SAMN02595329.GG740078_36	**typeXI**	208,2	low	Bacteroidota	Bacteroidia	Bacteroidales	Prevotellaceae	Prevotella	Prevotella sp. oral taxon 317	SAMN02595329	host-associated	10	Yes	4	NERD;DUF5895;RuvA_N;SprA_N	—
1129257.SAMEA6950934.CAINLJ0100	**typeXI**	207,7	low	Bacteroidota	Bacteroidia	—	—	—	uncultured Bacteroidia bacterium	SAMEA6950934	aquatic	10	Yes	4	TnpB_IS66;Phage_T4_Y06J;WYL	—
213322.SAMEA6951797.CAIUSK01000	**typeXI**	207,6	low	Bacteroidota	Flavobacteriia	Flavobacteriales	—	—	uncultured Flavobacteriales bacterium	SAMEA6951797	aquatic	8	Yes	4	TatA_B_E;Thiolase_N;DnaB_C;VW	—
1972561.SAMEA5279589.CAAEQH010	**typeXI**	207,6	low	Actinomycetota	Coriobacteriia	Eggerthellales	Eggerthellaceae	—	Eggerthellaceae bacterium	SAMEA5279589	host-associated	4	Yes	3	SMC_N	—
1965650.SAMN06473744.NFHT010000	**typeXI**	206,8	low	Bacteroidota	Bacteroidia	Bacteroidales	Rikenellaceae	Alistipes	Alistipes sp. An66	SAMN06473744	host-associated	10	Yes	4	DUF6140;TPR_12;PLDc_2;Cons_	—
212695.SAMEA6951713.CAIYXD010000	**typeXI**	206,7	low	Bacteroidota	Flavobacteriia	—	—	—	uncultured Flavobacteriia bacterium	SAMEA6951713	aquatic	8	Yes	4	DnaB_C;Pol_alpha_B_N	—
1129257.SAMEA6151141.CACZWF010	**typeXI**	205,8	low	Bacteroidota	Bacteroidia	—	—	—	uncultured Bacteroidia bacterium	SAMEA6151141	host-associated	10	Yes	4	DUF6242_C;PLDc_2;DUF4172;DU	—
2838443.SAMN15816893.DWXH01000	**typeXI**	205,6	low	Bacteroidota	Bacteroidia	Bacteroidales	Rikenellaceae	Alistipes	Candidatus Alistipes merdigallinarum	SAMN15816893	host-associated	10	Yes	4	Mgm101p;TPR_12;DUF6140	—
1855373.SAMN05216518.FNBQ010000	**typeXI**	205,4	low	Bacteroidota	Bacteroidia	Bacteroidales	—	—	Bacteroidales bacterium KHT7	SAMN05216518	host-associated	10	Yes	4	DAHP_synth_1;SIS;2-Hacid_dh_C	—
2212467.SAMN16348663.JAFZWM010	**typeXI**	205,4	low	Bacteroidota	Bacteroidia	Bacteroidales	Bacteroidaceae	—	Bacteroidaceae bacterium	SAMN16348663	—	10	Yes	0	—	—
2487074.SAMN10343198.RJUG010000	**typeXI**	205,3	low	Bacteroidota	Flavobacteriia	Flavobacteriales	Weeksellaceae	Kaistella	Kaistella daneshvariae	SAMN10343198	host-associated	10	Yes	4	Peptidase_G2;T1-F;Beta-prop_IFT1	—
1129257.SAMEA6153040.CADCRA010	**typeXI**	205,1	low	Bacteroidota	Bacteroidia	—	—	—	uncultured Bacteroidia bacterium	SAMEA6153040	host-associated	5	Yes	4	DUF87;DUF3920;RyR;LRR_5	—
475299.SAMN07621195.QKTX0100000	**typeXI**	205,1	low	Bacteroidota	Cytophagia	Cytophagales	Cyclobacteriaceae	Algoriphagus	Algoriphagus aquaeductus	SAMN07621195	aquatic	10	Yes	4	YhcG_C;T1-F;WYL;DUF4349	—
2044944.SAMN16426336.JADKCK010	**typeXI**	204,6	low	Bacteroidota	Sphingobacteriia	Sphingobacteriales	—	—	Sphingobacteriales bacterium	SAMN16426336	anthropogenic	10	Yes	4	DUF4835;4HBT;HisKin-conflict	—
2498093.SAMN12406360.VSPM01000	**typeXI**	204,1	low	Bacteroidota	Bacteroidia	Bacteroidales	Muribaculaceae	—	Muribaculaceae bacterium	SAMN12406360	host-associated	7	Yes	4	TPX2;WYL;UPF0227;bpMoxR	—
2026799.SAMN17496206.JAFMKE010	**typeXI**	204	low	Verrucomicrobiota	—	—	—	—	Verrucomicrobiota bacterium	SAMN17496206	aquatic	6	Yes	3	MORN_2;DnaB_C;ABC_tran	—
2301481.SAMEA8805003.CAJTLZ0100	**typeXI**	203,5	low	Bacteroidota	Bacteroidia	Bacteroidales	Muribaculaceae	—	uncultured Muribaculaceae bacterium	SAMEA8805003	host-associated	10	Yes	4	Hexapep;WYL;UPF0227;bpMoxR	—
1898104.SAMN16426506.JADKIU0100	**typeXI**	203,1	low	Bacteroidota	—	—	—	—	Bacteroidota bacterium	SAMN16426506	anthropogenic	10	Yes	4	TEBP_beta;DnaB_C;Transglut_cor	—
1218108.SAMN02440982.KB908302_8	**typeXI**	202,5	low	Bacteroidota	Flavobacteriia	Flavobacteriales	Weeksellaceae	Empedobacter	Empedobacter brevis	SAMN02440982	anthropogenic	10	Yes	4	Phage_integrase;WYL;HisKin-confli	—
1122991.SAMN02745169.QJJX010000	**typeXI**	200,3	low	Bacteroidota	Bacteroidia	Bacteroidales	Prevotellaceae	Hoylesella	Hoylesella shahii	SAMN02745169	host-associated	10	Yes	4	SprA_N;RuvA_N;Exonuc_VII_S;Ex	—
28132.SAMD00078755.AP018049_14	**typeXI**	200,3	low	Bacteroidota	Bacteroidia	Bacteroidales	Prevotellaceae	Prevotella	Prevotella melaninogenica	SAMD00078755	host-associated	10	Yes	4	Lycopene_cyc;Sigma70_r2;Aminotr	—
213322.SAMEA6956517.CAIQQJ01000	**typeXI**	199,7	low	Bacteroidota	Flavobacteriia	Flavobacteriales	—	—	uncultured Flavobacteriales bacterium	SAMEA6956517	temperature	4	Yes	2	ACBP4_C;DnaB_C	—
2099680.SAMN19791121.JAIBBO0100	**typeXI**	199,7	low	Methanobacteriota	Methanobacteria	Methanobacteriales	Methanobacteriaceae	—	Methanobacteriaceae archaeon	SAMN19791121	anthropogenic	5	Yes	2	YxeD;BUD31	—
2030927.SAMEA5278599.CAAFAP010	**typeXI**	199,2	low	Bacteroidota	Bacteroidia	Bacteroidales	—	—	Bacteroidales bacterium	SAMEA5278599	host-associated	10	Yes	4	Methyltransf_23;WYL;UPF0227;PD	—
1869212.SAMN17497506.JAFEIJ01000	**typeXI**	199,1	low	Bacteroidota	Chitinophagia	Chitinophagales	Chitinophagaceae	—	Chitinophagaceae bacterium	SAMN17497506	aquatic	10	Yes	4	2CompART;Methyltransf_23;DnaB_	—
213322.SAMEA8498464.CAJQJX01000	**typeXI**	198,6	low	Bacteroidota	Flavobacteriia	Flavobacteriales	—	—	uncultured Flavobacteriales bacterium	SAMEA8498464	aquatic	10	Yes	4	DUF2805;HpcH_HpaI;SpoU_methy	—
370804.SAMEA6152226.CADBLU0100	**typeXI**	198,5	low	Bacteroidota	Bacteroidia	Bacteroidales	Prevotellaceae	—	uncultured Prevotellaceae bacterium	SAMEA6152226	host-associated	5	Yes	2	DUF2201	—
2486470.SAMN10365287.RIBI0100000	**typeXI**	197,8	low	Bacteroidota	Bacteroidia	Bacteroidales	Muribaculaceae	—	Muribaculaceae bacterium Isolate-102	SAMN10365287	host-associated	10	Yes	4	DEAD;WYL;UPF0227	—
1236518.SAMN03897722.CP016204_3	**typeXI**	197,7	low	Bacteroidota	Bacteroidia	Bacteroidales	Prevotellaceae	Prevotella	Prevotella scopos	SAMN03897722	host-associated	10	Yes	4	Chorismate_bind;Aminotran_4;Sigm	—

563031.SAMN02463828.JH114149_60	typeXI	197	low	Bacteroidota	Bacteroidia	Bacteroidales	Prevotellaceae	Prevotella	Prevotella sp. C561	SAMN02463828	host-associated	10	Yes	4	Lycopene_cyc;Sigma70_r2;Aminotr	—
1898104.SAMN15870297.JACTMM010	typeXI	196,5	low	Bacteroidota	—	—	—	—	Bacteroidota bacterium	SAMN15870297	anthropogenic	5	Yes	2	WYL	—
1945886.SAMN06298210.FZRE010000	typeXI	195,9	low	Bacteroidota	Bacteroidia	Bacteroidales	Prevotellaceae	—	Prevotellaceae bacterium KH2P17	SAMN06298210	host-associated	10	Yes	4	Bac_DnaA;TonB_dep_Rec_b-barre	—
1236517.SAMN03704035.CP012074_1	typeXI	195,6	low	Bacteroidota	Bacteroidia	Bacteroidales	Prevotellaceae	Prevotella	Prevotella fusca	SAMN03704035	—	10	Yes	0	—	—
152509.SAMEA6951128.CAISCU01000	typeXI	195,6	low	Bacteroidota	—	—	—	—	uncultured Bacteroidota bacterium	SAMEA6951128	aquatic	10	Yes	4	Esterase;3-dmu-9_3-mt;DnaB_C;UI	—
888743.SAMN00253307.GL872282_81	typeXI	195,3	low	Bacteroidota	Bacteroidia	Bacteroidales	Prevotellaceae	Prevotella	Prevotella multiformis	SAMN00253307	host-associated	10	Yes	4	Lipase_GDSL_2;LRR_8	—
348578.SAMEA4891951.URTA0100000	typeXI	194,9	low	Bacteroidota	Bacteroidia	Bacteroidales	Porphyromonadaceae	—	uncultured Porphyromonadaceae bact	SAMEA4891951	host-associated	8	Yes	4	dCMP_cyt_deam_1;WYL;Ribosoma	—
28132.SAMN18352174.CP072361_100	typeXI	194,8	low	Bacteroidota	Bacteroidia	Bacteroidales	Prevotellaceae	Prevotella	Prevotella melaninogenica	SAMN18352174	host-associated	10	Yes	4	Chorismate_bind;Aminotran_4;Sigm	—
2840691.SAMN15816958.DVIH010000	typeXI	194,8	low	Bacteroidota	Bacteroidia	Bacteroidales	Bacteroidaceae	Candidatus Avibacteroi	Candidatus Avibacteroides excrementi	SAMN15816958	—	5	Yes	0	—	—
553174.SAMN00001916.CP002122_18	typeXI	194,1	low	Bacteroidota	Bacteroidia	Bacteroidales	Prevotellaceae	Prevotella	Prevotella melaninogenica	SAMN00001916	—	10	Yes	0	—	—
1953166.SAMN06454853.DITN010000	typeXI	194,1	low	Bacteroidota	—	—	—	—	Bacteroidetes bacterium UBA6183	SAMN06454853	aquatic	10	Yes	4	PALP;Glucosaminidase;DnaB_C;Cl	—
767031.SAMN00031760.CP002589_44	typeXI	193,4	low	Bacteroidota	Bacteroidia	Bacteroidales	Prevotellaceae	Prevotella	Prevotella denticola	SAMN00031760	host-associated	10	Yes	4	Chorismate_bind;Aminotran_4;GT8	—
1913989.SAMN09215160.QNFE010000	typeXI	193	low	Pseudomonadota	Gammaproteobacteria	—	—	—	Gammaproteobacteria bacterium	SAMN09215160	temperature	10	Yes	4	DUF488;dnstrm_HI1420;Response	—
2301481.SAMEA8805108.CAJTOZ0100	typeXI	191,4	low	Bacteroidota	Bacteroidia	Bacteroidales	Muribaculaceae	—	uncultured Muribaculaceae bacterium	SAMEA8805108	host-associated	10	Yes	4	DUF1453;PLDc_2;PM0109;IDEAL	—
2768039.SAMEA8805111.CAJTQR0100	typeXI	191,3	low	Bacteroidota	Bacteroidia	Bacteroidales	Muribaculaceae	Duncaniella	uncultured Duncaniella sp.	SAMEA8805111	host-associated	10	Yes	4	GmrSD_N;DpnD-PcfM;PLDc_2	—
1872444.SAMN16342050.JAFQCA0100	typeXI	191,1	low	Bacteroidota	Bacteroidia	Bacteroidales	Rikenellaceae	Alistipes	Alistipes sp.	SAMN16342050	host-associated	9	Yes	4	DUF169;HTH_17	—
2045217.SAMN16425918.CP064974_5	typeXI	185,5	low	Candidatus Moraniibacter	—	—	—	—	Candidatus Moraniibacteriota bacterium	SAMN16425918	anthropogenic	10	Yes	4	CCB3_YggT;G0-G1_switch_2;MCM	—
1898104.SAMN21435363.JAIUPL01000	typeXI	179,2	low	Bacteroidota	—	—	—	—	Bacteroidota bacterium	SAMN21435363	anthropogenic	6	Yes	3	CRISPR_Cas2;Cas_Cas1	—
1262921.PRJEB697.HF992632_9	typeXI	176,1	low	Bacteroidota	Bacteroidia	Bacteroidales	Prevotellaceae	Prevotella	Prevotella sp. CAG:1185	PRJEB697	host-associated	7	Yes	4	WG_beta_rep;Fer4_7	—
2202734.SAMN14239044.JABWBR010	typeXI	170,7	low	Bacteroidota	Saprospiria	Saprospirales	Saprospiraceae	—	Saprospiraceae bacterium	SAMN14239044	anthropogenic	8	Yes	4	PIN_3;DUF1801;CRISPR_Cas2;C	—
2202734.SAMN16425902.JADJNF0100	typeXI	164,1	low	Bacteroidota	Saprospiria	Saprospirales	Saprospiraceae	—	Saprospiraceae bacterium	SAMN16425902	anthropogenic	10	Yes	4	TackOD1;CotH;Por_Secre_tail	—
194843.SAMEA6951536.CAIYES01000	typeXI	155,6	low	Bacteroidota	Bacteroidia	Bacteroidales	—	—	uncultured Bacteroidales bacterium	SAMEA6951536	aquatic	8	Yes	4	OKR_DC_1_N;Cas6b_C;Cas_Cas	—
194843.SAMEA6951804.CAIQUS01000	typeXI	148,3	low	Bacteroidota	Bacteroidia	Bacteroidales	—	—	uncultured Bacteroidales bacterium	SAMEA6951804	aquatic	8	Yes	4	CRISPR_Cas2;Cas_Cas1;Cas6b_(	—
2013697.SAMN06767598.PHDA010000	typeXI	139,5	low	Bacteroidota	—	—	—	—	Bacteroidetes bacterium HGW-Bacter	SAMN06767598	terrestrial	7	Yes	4	Phosphoprotein;Cas6b_C;Cas_Cas	—
42895.SAMN17801374.JAHABZ010000	typeXII	299	high	Pseudomonadota	Gammaproteobacteria	Enterobacterales	Enterobacteriaceae	Enterobacter	Enterobacter sp.	SAMN17801374	host-associated	11	Yes	4	DUF5375;Zn_Ribbon_Prim;RVT_1	—
685445.SAMN02440965.KB911089_44	typeXII	295,7	high	Pseudomonadota	Gammaproteobacteria	Enterobacterales	Enterobacteriaceae	Klebsiella	Klebsiella aerogenes	SAMN02440965	host-associated	11	Yes	4	HTH_3;TMEM170A_B;Resolvase;F	—
910996.SAMN10245858.RCWP010000	typeXII	294,5	high	Pseudomonadota	Gammaproteobacteria	Enterobacterales	Hafniaceae	Hafnia	Hafnia alvei	SAMN10245858	—	11	Yes	0	—	—
1608994.SAMN03328806.JYLF0100000	typeXII	292	high	Pseudomonadota	Gammaproteobacteria	Pseudomonadales	Pseudomonadaceae	Pseudomonas	Pseudomonas weihenstephanensis	SAMN03328806	anthropogenic	11	Yes	4	Arfaptin;ThiD2	—
1211707.PRJNA170904.HF570389_10	typeXII	284,5	high	Pseudomonadota	Gammaproteobacteria	Lysobacterales	Lysobacteraceae	Xanthomonas	Xanthomonas campestris	PRJNA170904	anthropogenic	11	Yes	4	Glyco_hydro_15;Trehalose_PPase;	—
190485.SAMN02603845.AE008922_36	typeXII	284,5	high	Pseudomonadota	Gammaproteobacteria	Lysobacterales	Lysobacteraceae	Xanthomonas	Xanthomonas campestris	SAMN02603845	—	11	Yes	0	—	—
406819.SAMN02141584.AJHK0200001	typeXII	284,5	high	Pseudomonadota	Betaproteobacteria	Burkholderiales	Burkholderiaceae	Burkholderia	Burkholderia sp. SJ98	SAMN02141584	terrestrial	11	Yes	4	ER_lumen_recept;MCPsignal;Dabb	—
1802253.SAMN04313803.MIBZ010000	typeXII	284,3	high	Campylobacterota	Epsilonproteobacteria	Campylobacterales	Sulfurimonadaceae	Sulfurimonas	Sulfurimonas sp. RIFCSPLOWO2_12	SAMN04313803	terrestrial	8	Yes	4	PhdYeFM_antitox;ParE_toxin;DUF6	—
1417228.SAMN04498477.CP014579_1	typeXII	281,9	high	Pseudomonadota	Betaproteobacteria	Burkholderiales	Burkholderiaceae	Paraburkholderia	Paraburkholderia phytofirmans	SAMN04498477	temperature	11	Yes	4	MacP_activator;SIR2_2;Nuc_deoxy	—
2026748.SAMN16635730.JADMJM0100	typeXII	279	high	Pseudomonadota	Alphaproteobacteria	Hyphomonadales	Hyphomonadaceae	—	Hyphomonadaceae bacterium	SAMN16635730	terrestrial	11	Yes	4	AAA_31;TPR_12	—
2282150.SAMN18059877.JAFLCU0100	typeXII	278,5	high	Pseudomonadota	Alphaproteobacteria	Caulobacterales	—	—	Caulobacterales bacterium	SAMN18059877	anthropogenic	6	Yes	2	Acyl-CoA_dh_M;AKAP2_C	—
224209.SAMEA8805150.CAJTUR0100	typeXII	273,5	high	Bacillota	Bacilli	—	—	—	uncultured Bacilli bacterium	SAMEA8805150	host-associated	11	Yes	4	HTH_3;FtsX;DnaB_C;Dockerin_1	—
1913988.SAMN16745442.JAGWZH010	typeXII	269,8	high	Pseudomonadota	Alphaproteobacteria	—	—	—	Alphaproteobacteria bacterium	SAMN16745442	aquatic	7	Yes	3	Acetyltransf_6;TPR_19	—
1913988.SAMN19298566.JAHJSN0100	typeXII	268,6	high	Pseudomonadota	Alphaproteobacteria	—	—	—	Alphaproteobacteria bacterium	SAMN19298566	terrestrial	11	Yes	4	DUF2147;Gly_transporter;DUF128	—
1479235.SAMN03785500.LJZS0100000	typeXII	268,6	high	Pseudomonadota	Gammaproteobacteria	Oceanospirillales	Halomonadaceae	Halomonas	Halomonas sp. HL-48	SAMN03785500	salinity	11	Yes	4	Sbt_1;ZinT;SmpB;Polyketide_cyc	—
1116369.SAMN02441719.KB890024_1	typeXII	260,8	low	Pseudomonadota	Alphaproteobacteria	Hyphomicrobiales	Rhizobiaceae	Hoeflea	Hoeflea sp. 108	SAMN02441719	anthropogenic	10	Yes	4	FA_desaturase;DUF5631;Phage_in	—
2862676.SAMN20394465.JAHXYK0100	typeXII	256,5	low	Pseudomonadota	Gammaproteobacteria	Lysobacterales	Lysobacteraceae	Lysobacter	Lysobacter sp. ESA13C	SAMN20394465	host-associated	10	Yes	4	Zeta_toxin;MraZ;XAC0095_dom;NA	—
194843.SAMEA104666335.ONBO0100	typeXII	229,4	low	Bacteroidota	Bacteroidia	Bacteroidales	—	—	uncultured Bacteroidales bacterium	SAMEA104666335	host-associated	10	Yes	4	AAA-ATPase_like;HAD_SAK_2	—
152509.SAMEA6946966.CAITFF01000	typeXII	226,1	low	Bacteroidota	—	—	—	—	uncultured Bacteroidota bacterium	SAMEA6946966	aquatic	8	Yes	4	MHV_Nsp3_DPUP;DUF4784;DnaE	—
1913988.SAMN12581861.WRAU01000	typeXII	213,6	low	Pseudomonadota	Alphaproteobacteria	—	—	—	Alphaproteobacteria bacterium	SAMN12581861	host-associated	10	Yes	4	NRDD;Fer4_12;NMO;RNase_PH	—
1300343.SAMN03135132.JSAQ010000	typeXII	211,4	low	Bacteroidota	Flavobacteriia	Flavobacteriales	Flavobacteriaceae	Dokdonia	Dokdonia donghaensis	SAMN03135132	host-associated	10	Yes	4	Ribosomal_S15;RNase_PH;GH85_	—
2055788.SAMN08019674.DOVO01000	typeXIII	364,3	high	Cyanobacteriota	—	—	—	—	Cyanobacteria bacterium UBA9226	SAMN08019674	anthropogenic	10	Yes	4	Response_reg;LCIB_C_CA;CBM_1	—
2055779.SAMN08018872.DNXU010000	typeXIII	363,4	high	Cyanobacteriota	—	—	—	—	Cyanobacteria bacterium UBA8803	SAMN08018872	anthropogenic	4	Yes	3	Response_reg	—
2692870.SAMN13702772.JACJPF0100	typeXIII	362,5	high	Cyanobacteriota	Cyanophyceae	Leptolyngbyales	Trichocoleusaceae	Trichocoleus	Trichocoleus sp. FACHB-40	SAMN13702772	terrestrial	9	Yes	4	SWIM;VWA	—
1211.SAMEA6944671.CAIRPA0100003	typeXIII	362,4	high	Cyanobacteriota	—	—	—	—	uncultured cyanobacterium	SAMEA6944671	aquatic	11	Yes	4	WGR;SWIM;PaO;Metallophos	—
1454205.SAMN05826284.CP017708_2	typeXIII	359,2	high	Cyanobacteriota	Cyanophyceae	Coleofasciculales	Coleofasciculaceae	Moorena	Moorena producens	SAMN05826284	aquatic	11	Yes	4	DUF2157;Pneumo_M2;XisI;XisH	—
393003.SAMN09074762.QLNL0100000	typeXIII	355,3	high	Bacteroidota	Chitinophagia	Chitinophagales	Chitinophagaceae	Chitinophaga	Chitinophaga ginsengisegetis	SAMN09074762	host-associated	11	Yes	4	WGR;SWIM;TonB_dep_Rec_b-bar	—
1157708.SAMN02440495.KB907450_1	typeXIII	353,1	high	Pseudomonadota	Betaproteobacteria	Burkholderiales	Comamonadaceae	Variovorax	Variovorax atrisoli	SAMN02440495	host-associated	11	Yes	4	HEAT_2;SWIM;Acetyltransf_1;RIO	—
1869181.SAMN17140208.JAFAIX0100	typeXIII	352,6	high	Bacteroidota	Chitinophagia	Chitinophagales	Chitinophagaceae	Chitinophaga	Chitinophaga sp.	SAMN17140208	terrestrial	11	Yes	4	WGR;SWIM;TonB_dep_Rec_b-bar	—
1095768.PRJEA70551.HE578945_39	typeXIII	350,3	high	Pseudomonadota	Gammaproteobacteria	Enterobacterales	Enterobacteriaceae	Phytobacter	Phytobacter massiliensis	PRJEA70551	host-associated	11	Yes	4	WGR;SWIM;HATPase_c;Lipoprote	—
1849603.SAMN17801224.JAGZWF010	typeXIII	349,3	high	Pseudomonadota	Gammaproteobacteria	Enterobacterales	Enterobacteriaceae	—	Enterobacteriaceae bacterium	SAMN17801224	host-associated	11	Yes	4	WGR;SWIM;HATPase_c;Lipoprote	—
1884385.SAMN05518669.FNOL010000	typeXIII	348,9	high	Pseudomonadota	Betaproteobacteria	Burkholderiales	Comamonadaceae	Variovorax	Variovorax sp. YR634	SAMN05518669	host-associated	11	Yes	4	HEAT_2;SWIM;Acetyltransf_1;RIO	—
1871043.SAMN18061513.JAFKMS0100	typeXIII	347,2	high	Pseudomonadota	Betaproteobacteria	Burkholderiales	Comamonadaceae	Variovorax	Variovorax sp.	SAMN18061513	anthropogenic	11	Yes	4	HEAT_2;SWIM;Acetyltransf_1;RIO	—
1882827.SAMN05443579.FOWG01000	typeXIII	347,2	high	Pseudomonadota	Betaproteobacteria	Burkholderiales	Comamonadaceae	Variovorax	Variovorax sp. PDC80	SAMN05443579	anthropogenic	11	Yes	4	HEAT_2;SWIM;4HBT;RIO1	—
1297865.SAMN02952940.APJD010000	typeXIII	346,9	high	Pseudomonadota	Alphaproteobacteria	Hyphomicrobiales	Nitrobacteraceae	Bradyrhizobium	Bradyrhizobium sp. OHSU_III	SAMN02952940	anthropogenic	11	Yes	4	LplA-B_cat;FliMN_C;SWIM;WGR	—
1761785.SAMN04487979.FZNL010000	typeXIII	346,3	high	Bacteroidota	Flavobacteriia	Flavobacteriales	Flavobacteriaceae	Flavobacterium	Flavobacterium sp. ov086	SAMN04487979	host-associated	11	Yes	4	SWIM;WGR;AMP-binding;DUF771	—
1128427.SAMN02256433.KB904821_2	typeXIII	346,1	high	Cyanobacteriota	Cyanophyceae	Oscillatoriales	—	—	filamentous cyanobacterium ESFC-1	SAMN02256433	salinity	11	Yes	4	Endo_dU;Uma2;Psb34	—
2789216.SAMN12641115.CP043489_3	typeXIII	344,8	high	Pseudomonadota	Alphaproteobacteria	Hyphomicrobiales	Xanthobacteraceae	Labrys	Labrys sp. KNU-23	SAMN12641115	host-associated	11	Yes	4	WGR;SWIM;Rubrerythrin;Yip1	—
2056868.SAMN08111093.QEHP01000	typeXIII	344,8	high	Bacteroidota	Flavobacteriia	Flavobacteriales	Weeksellaceae	Chryseobacterium	Chryseobacterium sp. HMWF035	SAMN08111093	anthropogenic	11	Yes	4	Arm-DNA-bind_5;WGR;SWIM	—
2578106.SAMN11620940.CP042171_9	typeXIII	344,2	high	Bacteroidota	Sphingobacteriia	Sphingobacteriales	Sphingobacteriaceae	Pedobacter	Pedobacter sp. KBS0701	SAMN11620940	host-associated	11	Yes	4	DUF4953;LRR_ComC;WGR;SWIM	—
2587064.SAMN12024188.JACHWM010	typeXIII	343	high	Pseudomonadota	Alphaproteobacteria	Hyphomicrobiales	Methylobacteriaceae	Methylobacterium	Methylobacterium sp. R2-1	SAMN12024188	terrestrial	11	Yes	4	WGR;SWIM;Acetyltransf_6	—
651561.SAMN06453202.DCFI0100002	typeXIII	343	high	Bacteroidota	Flavobacteriia	Flavobacteriales	Weeksellaceae	Chryseobacterium	Chryseobacterium arthrosphaerae	SAMN06453202	host-associated	11	Yes	4	Amidohydro_1;Urocanase_C;WGR;	—
946333.SAMN04621830.CP015118_35	typeXIII	342,7	high	Pseudomonadota	Betaproteobacteria	Burkholderiales	Sphaerotilaceae	Piscinibacter	Piscinibacter gummiphilus	SAMN04621830	host-associated	11	Yes	4	XPC-binding;SWIM;LysR_substrate	—
2026763.SAMN10607053.SKYM010000	typeXIII	341,6	high	Myxococcota	Myxococcia	Myxococcales	—	—	Myxococcales bacterium	SAMN10607053	salinity	8	Yes	4	SWIM;Beta-prop_IFT122_1st;DUF	—
1230476.SAMN01893863.KE747879_2	typeXIII	338,7	high	Pseudomonadota	Alphaproteobacteria	Hyphomicrobiales	Nitrobacteraceae	Bradyrhizobium	Bradyrhizobium sp. DFCI-1	SAMN01893863	anthropogenic	11	Yes	4	Polysacc_deac_1;DUF4148;FliMN_	—
2015576.SAMN07280669.NKIY0100000	typeXIII	338,7	high	Pseudomonadota	Alphaproteobacteria	Hyphomicrobiales	Nitrobacteraceae	—	Bradyrhizobiaceae bacterium PARB1	SAMN07280669	—	11	Yes	0	—	—
1045867.SAMN18262562.JAGGDU010	typeXIII	338,1	high	Cyanobacteriota	Cyanophyceae	Oscillatoriales	Microcoleaceae	Trichodesmium	Trichodesmium erythraeum	SAMN18262562	aquatic	11	Yes	4	GATase;Ribonucleas_3_3;Glyco_tr	—
2040292.SAMEA104414344.OEPV0100	typeXIII	338	high	Bacteroidota	Bacteroidia	Bacteroidales	Dysgonomonadaceae	Pseudodysgonomonas	Pseudodysgonomonas massiliensis	SAMEA104414344	host-associated	11	Yes	4	Bgal_small_N;Alpha_L_fucos;WGR	—
693986.SAMN03075686.CP003811_16	typeXIII	337,9	high	Pseudomonadota	Alphaproteobacteria	Hyphomicrobiales	Methylobacteriaceae	Methylobacterium	Methylobacterium oryzae	SAMN03075686	—	11	Yes	0	—	—
426355.SAMN00000277.CP001001_13	typeXIII	337,6	high	Pseudomonadota	Alphaproteobacteria	Hyphomicrobiales	Methylobacteriaceae	Methylobacterium	Methylobacterium radiotolerans	SAMN00000277	anthropogenic	11	Yes	4	Enterotoxin_HS1;SWIM;WGR	—
179408.SAMN02261335.CP003614_51	typeXIII	335,8	high	Cyanobacteriota	Cyanophyceae	Oscillatoriales	Oscillatoriaceae	Phormidium	Phormidium nigroviride	SAMN02261335	anthropogenic	11	Yes	4	HicA_toxin;HicB_lk_antitox;DUF567	—
2587122.SAMN12024204.JACHYS0100	typeXIII	335,6	high	Pseudomonadota	Betaproteobacteria	Burkholderiales	Sphaerotilaceae	Roseateles	Mitsuaria sp. BK037	SAMN12024204	host-associated	11	Yes	4	XPC-binding;SWIM;YitT_membrane	—
2777973.SAMN16351716.JADEWD010	typeXIII	334,5	high	Cyanobacteriota	Cyanophyceae	Nostocales	Aphanizomenonaceae	Cuspidothrix	Cuspidothrix issatschenkoi	SAMN16351716	aquatic	6	Yes	2	HSP70;NAGPA	—
398578.SAMN00623041.CP000884_16	typeXIII	332,6	high	Pseudomonadota	Betaproteobacteria	Burkholderiales	Comamonadaceae	Delftia	Delftia acidovorans	SAMN00623041	host-associated	11	Yes	4	TPR_NOC4;HTH_18;SWIM;XPC-b	—
2448779.SAMN18059842.JAFLBL0100	typeXIII	331,4	high	Bacteroidota	Chitinophagia	Chitinophagales	—	—	Chitinophagales bacterium	SAMN18059842	anthropogenic	11	Yes	4	WGR;SWIM;Amidohydro_1;LacI	—

1032.SAMN18076803.JAGVHW010000	typeXIII	330,9	high	Pseudomonadota	Gammaproteobacteria	Thiotrichales	Thiotrichaceae	Thiothrix	Thiothrix sp.	SAMN18076803	anthropogenic	11	Yes	4	Aminotran_5;WGR;SWIM	—
2052484.SAMN07200918.NIOE0100000	typeXIII	330,8	high	Pseudomonadota	Betaproteobacteria	Burkholderiales	Sphaerotilaceae	Roseateles	Roseateles noduli	SAMN07200918	host-associated	11	Yes	4	Amidohydro_2;YitT_membrane;SWI	—
513160.SAMN12129767.VIDT0100084	typeXIII	330,6	high	Pseudomonadota	Betaproteobacteria	Burkholderiales	Sphaerotilaceae	Ideonella	Ideonella azotifigens	SAMN12129767	host-associated	2	Yes	1	SWIM	—
2692887.SAMN13702731.JACJQT0100	typeXIII	330,3	high	Cyanobacteriota	Cyanophyceae	Nostocales	Aphanizomenonaceae	Aphanizomenon	Aphanizomenon flos-aquae	SAMN13702731	aquatic	11	Yes	4	HicA_toxin;Peptidase_C13;AdoHcy	—
2745558.SAMN15428588.CP058988_2	typeXIII	328,1	high	Bacteroidota	Flavobacteriia	Flavobacteriales	Flavobacteriaceae	Cellulophaga	Cellulophaga sp. HaHa_2_95	SAMN15428588	host-associated	11	Yes	4	WGR;SWIM;LRR_14	—
100233.SAMEA6952382.CAIXQV01000	typeXIII	327,7	high	Planctomycetota	Planctomycetia	Planctomycetales	Planctomycetaceae	—	uncultured Planctomycetaceae bacteri	SAMEA6952382	aquatic	4	Yes	3	Sulfatase;RlaP	—
179408.SAMN02261335.CP003614_38	typeXIII	326,5	high	Cyanobacteriota	Cyanophyceae	Oscillatoriales	Oscillatoriaceae	Phormidium	Phormidium nigroviride	SAMN02261335	anthropogenic	11	Yes	4	DEAD;Band_7;FrhB_FdhB_C	—
2026763.SAMN16426551.JADKKK010	typeXIII	326,4	high	Myxococcota	Myxococcia	Myxococcales	—	—	Myxococcales bacterium	SAMN16426551	anthropogenic	11	Yes	4	WGR;SWIM;Sel1;ABC_tran	—
2651161.SAMN12878373.WBUJ010000	typeXIII	325,9	high	Pseudomonadota	Gammaproteobacteria	—	Candidatus Competibacte	Candidatus Contendiba	Candidatus Contendobacter sp.	SAMN12878373	—	9	Yes	0	—	—
2052164.SAMN08179277.PLDW01000	typeXIII	325,5	high	Planctomycetota	Planctomycetia	Gemmatales	Gemmataceae	—	Gemmataceae bacterium	SAMN08179277	temperature	11	Yes	4	Trypsin_2;PDZ_2;SWIM;HEAT_2	—
2053538.SAMN10587497.RXIW010000	typeXIII	324,9	high	Pseudomonadota	Gammaproteobacteria	—	Candidatus Competibacte	—	Candidatus Competibacteraceae bacte	SAMN10587497	anthropogenic	9	Yes	4	WGR;SWIM;Lactate_perm	—
2026763.SAMN16426013.JADJQX010	typeXIII	324,1	high	Myxococcota	Myxococcia	Myxococcales	—	—	Myxococcales bacterium	SAMN16426013	anthropogenic	11	Yes	4	WGR;SWIM;DUF937	—
2562705.SAMEA6956407.CAIXXB0100	typeXIII	324,1	high	Verrucomicrobiota	Verrucomicrobiia	Verrucomicrobiales	Akkermansiaceae	—	Akkermansiaceae bacterium	SAMEA6956407	aquatic	6	Yes	2	NHL;PIN	—
2024858.SAMN07620073.PABA010001	typeXIII	323,8	high	Myxococcota	—	Polyangiales	Sandaracinaceae	Sandaracinus	Sandaracinus sp.	SAMN07620073	aquatic	8	Yes	4	HEAT_2;SWIM;PEGA	—
92487.SAMN02745130.FUYB01000016	typeXIII	321,8	high	Pseudomonadota	Gammaproteobacteria	Thiotrichales	Thiotrichaceae	Thiothrix	Thiothrix eikelboomii	SAMN02745130	anthropogenic	11	Yes	4	DCC1-like;dCache_2;SWIM;HEAT_	—
2053538.SAMN16426005.JADJQQ0100	typeXIII	321,7	high	Pseudomonadota	Gammaproteobacteria	—	Candidatus Competibacte	—	Candidatus Competibacteraceae bacte	SAMN16426005	anthropogenic	11	Yes	4	WGR;Chalcone_3;SWIM	—
502025.SAMN00002596.CP001804_15	typeXIII	321,5	high	Myxococcota	—	Haliangiales	Kofleriaceae	Haliangium	Haliangium ochraceum	SAMN00002596	salinity	11	Yes	4	HEAT_2;SWIM;WGR	—
1131567.SAMD00245618.BMYU01000	typeXIII	321,3	high	Pseudomonadota	Betaproteobacteria	Burkholderiales	Oxalobacteraceae	Undibacterium	Undibacterium squillarum	SAMD00245618	anthropogenic	11	Yes	4	Phosphoesterase;CC_CEP250;SW	—
2052181.SAMN11369556.SYPF010001	typeXIII	321,3	high	Planctomycetota	Planctomycetia	—	—	—	Planctomycetia bacterium	SAMN11369556	aquatic	8	Yes	4	Pan_kinase;MMR_HSR1;SWIM	—
2053538.SAMN16426005.JADJQQ0100	typeXIII	320,5	high	Pseudomonadota	Gammaproteobacteria	—	Candidatus Competibacte	—	Candidatus Competibacteraceae bacte	SAMN16426005	anthropogenic	11	Yes	0	—	—
1951615.SAMN06451712.DCNP01000	typeXIII	320	high	Pseudomonadota	Gammaproteobacteria	—	Candidatus Competibacte	—	Competibacteraceae bacterium UBA1	SAMN06451712	anthropogenic	8	Yes	4	AAA_22;SWIM;WGR	—
1965545.SAMN06473586.NFLT010000	typeXIII	318,9	high	Bacillota	Clostridia	Lachnospirales	Lachnospiraceae	Tyzzerella	Tyzzerella sp. An114	SAMN06473586	host-associated	11	Yes	4	DUF6849;SWIM;PTS_EIIA_2;PTS	—
2769423.SAMD00245656.BNCN01000	typeXIII	318,3	high	Pseudomonadota	Betaproteobacteria	Burkholderiales	Comamonadaceae	Comamonas	Comamonas sp. KCTC 72670	SAMD00245656	host-associated	11	Yes	4	GMC_oxred_C;Hydrolase_4;SWIM	—
156588.SAMEA6945809.CAJCBL0100	typeXIII	318,2	high	Verrucomicrobiota	—	—	—	—	uncultured Verrucomicrobiota bacteriu	SAMEA6945809	aquatic	10	Yes	4	OTCace;DUF3239;SWIM;HEAT_2	—
261164.SAMN04549283.CP014945_13	typeXIII	315,6	high	Pseudomonadota	Gammaproteobacteria	Moraxellales	Moraxellaceae	Psychrobacter	Psychrobacter alimentarius	SAMN04549283	anthropogenic	11	Yes	4	HEAT_2;SWIM;Lipase_GDSL_2;C	—
2024858.SAMN07620073.PABA010001	typeXIII	315,5	high	Myxococcota	—	Polyangiales	Sandaracinaceae	Sandaracinus	Sandaracinus sp.	SAMN07620073	aquatic	7	Yes	3	23S_rRNA_IVP;NTP_transf_9;DUF	—
244328.SAMEA8805375.CAJTZO0100	typeXIII	315,5	high	Bacillota	Clostridia	—	—	—	uncultured Clostridia bacterium	SAMEA8805375	host-associated	11	Yes	4	CoaE;DNA_pol_A;SWIM;DUF6849	—
2053632.SAMN19225299.DXZA010000	typeXIII	315,4	high	Bacillota	Clostridia	Lachnospirales	Lachnospiraceae	Tyzzerella	Tyzzerella sp.	SAMN19225299	host-associated	11	Yes	4	DUF6849;SWIM;ProRS-C_1;Lar_r	—
244328.SAMEA7202299.CAJFIR0100	typeXIII	314,8	high	Bacillota	Clostridia	—	—	—	uncultured Clostridia bacterium	SAMEA7202299	host-associated	10	Yes	4	DUF6849;SWIM;Pantoate_transf;A	—
2053538.SAMN16425960.JADJPA0100	typeXIII	314,1	high	Pseudomonadota	Gammaproteobacteria	—	Candidatus Competibacte	—	Candidatus Competibacteraceae bacte	SAMN16425960	—	11	Yes	0	—	—
244328.SAMEA8805706.CAJUKL0100	typeXIII	313,9	high	Bacillota	Clostridia	—	—	—	uncultured Clostridia bacterium	SAMEA8805706	—	11	Yes	0	—	—
297314.SAMEA8805501.CAJUDQ0100	typeXIII	312,8	high	Bacillota	Clostridia	Lachnospirales	Lachnospiraceae	—	uncultured Lachnospiraceae bacterium	SAMEA8805501	host-associated	11	Yes	4	ABC_membrane;Response_reg;SW	—
297314.SAMEA8805536.CAJUIB0100	typeXIII	312,8	high	Bacillota	Clostridia	Lachnospirales	Lachnospiraceae	—	uncultured Lachnospiraceae bacterium	SAMEA8805536	—	11	Yes	0	—	—
997898.SAMN02596761.KB851130_27	typeXIII	312,7	high	Bacillota	Clostridia	Eubacteriales	Clostridiaceae	Clostridium	Clostridium butyricum	SAMN02596761	host-associated	11	Yes	4	MarR;A_deaminase;SWIM;DUF684	—
1946346.SAMN06454910.DCDL01000	typeXIII	312,2	high	Bacillota	Clostridia	Eubacteriales	Clostridiaceae	Clostridium	Clostridium sp. UBA1056	SAMN06454910	anthropogenic	11	Yes	4	DUF6849;SWIM;Resolvase	—
244328.SAMEA8805806.CAJUOZ0100	typeXIII	311,5	high	Bacillota	Clostridia	—	—	—	uncultured Clostridia bacterium	SAMEA8805806	host-associated	11	Yes	4	DUF6849;SWIM;AA_permease_2;	—
2052164.SAMN08179277.PLDW01000	typeXIII	311,4	high	Planctomycetota	Planctomycetia	Gemmatales	Gemmataceae	—	Gemmataceae bacterium	SAMN08179277	temperature	11	Yes	4	HEAT_2;SWIM;Pkinase;Sigma70_	—
297314.SAMEA8805527.CAJUDE0100	typeXIII	311,2	high	Bacillota	Clostridia	Lachnospirales	Lachnospiraceae	—	uncultured Lachnospiraceae bacterium	SAMEA8805527	host-associated	11	Yes	4	DUF6849;SWIM;DUF6056;DUF60	—
2024858.SAMN07620073.PABA010001	typeXIII	311	high	Myxococcota	—	Polyangiales	Sandaracinaceae	Sandaracinus	Sandaracinus sp.	SAMN07620073	aquatic	11	Yes	4	DUF779;Abhydrolase_1;SWIM;HE	—
446043.SAMEA8805476.CAJUBU0100	typeXIII	310,9	high	Bacillota	Clostridia	Lachnospirales	Lachnospiraceae	Lachnospira	uncultured Lachnospira sp.	SAMEA8805476	host-associated	11	Yes	4	DUF6849;SWIM;SBP_bac_1;HATF	—
100233.SAMEA6949581.CAIYBH01000	typeXIII	310,7	high	Planctomycetota	Planctomycetia	Planctomycetales	Planctomycetaceae	—	uncultured Planctomycetaceae bacteri	SAMEA6949581	aquatic	11	Yes	4	LRR_RI_capping;Methyltransf_11;	—
1898207.SAMN13894064.JAAYMT010	typeXIII	310,6	high	Bacillota	Clostridia	Eubacteriales	—	—	Clostridiales bacterium	SAMN13894064	temperature	7	Yes	3	SWIM;DUF4179	—
297314.SAMEA8805036.CAJTOU0100	typeXIII	309,9	high	Bacillota	Clostridia	Lachnospirales	Lachnospiraceae	—	uncultured Lachnospiraceae bacterium	SAMEA8805036	—	6	Yes	0	—	—
707003.SAMEA8805243.CAJTTB01000	typeXIII	309,5	high	Bacillota	Clostridia	Eubacteriales	Oscillospiraceae	—	uncultured Oscillospiraceae bacterium	SAMEA8805243	host-associated	11	Yes	4	MIF;NUDIX;SWIM;DUF6849	—
935198.SAMN02603538.CP001056_18	typeXIII	309,3	high	Bacillota	Clostridia	Eubacteriales	Clostridiaceae	Clostridium	Clostridium botulinum	SAMN02603538	host-associated	11	Yes	4	Exo_endo_phos;DUF4004;SWIM;D	—
1946690.SAMN06453118.DESG01000	typeXIII	309,3	high	Bacillota	Clostridia	Lachnospirales	Lachnospiraceae	Lachnoclostridium	Lachnoclostridium sp. UBA3320	SAMN06453118	host-associated	10	Yes	4	DUF6849;SWIM;DUF848	—
244328.SAMEA8805759.CAJUNT0100	typeXIII	309,1	high	Bacillota	Clostridia	—	—	—	uncultured Clostridia bacterium	SAMEA8805759	host-associated	8	Yes	3	DUF6548;SWIM;DUF6849	—
100233.SAMEA6949573.CAIYBK01000	typeXIII	308,6	high	Planctomycetota	Planctomycetia	Planctomycetales	Planctomycetaceae	—	uncultured Planctomycetaceae bacteri	SAMEA6949573	aquatic	10	Yes	4	GFO_IDH_MocA_C2;ACP_syn_III_	—
297314.SAMEA8805577.CAJUGZ0100	typeXIII	308,3	high	Bacillota	Clostridia	Lachnospirales	Lachnospiraceae	—	uncultured Lachnospiraceae bacterium	SAMEA8805577	host-associated	9	Yes	4	Response_reg;HATPase_c_5;SWI	—
251229.SAMN02261359.CP003597_35	typeXIII	307,8	high	Cyanobacteriota	Cyanophyceae	Chroococcidiopsidales	Chroococcidiopsidaceae	Chroococcidiopsis	Chroococcidiopsis thermalis	SAMN02261359	anthropogenic	11	Yes	4	DUF7755;ParE_toxin;Band_7	—
2026763.SAMN17180964.JAEUJY0100	typeXIII	307,7	high	Myxococcota	Myxococcia	Myxococcales	—	—	Myxococcales bacterium	SAMN17180964	anthropogenic	7	Yes	4	HEAT_2;SWIM;WGR;DNA_ligase_	—
297314.SAMEA8805519.CAJUCS0100	typeXIII	307,7	high	Bacillota	Clostridia	Lachnospirales	Lachnospiraceae	—	uncultured Lachnospiraceae bacterium	SAMEA8805519	host-associated	11	Yes	4	DUF6849;SWIM;ROK;Response_r	—
297314.SAMEA8804990.CAJTJY0100	typeXIII	307,6	high	Bacillota	Clostridia	Lachnospirales	Lachnospiraceae	—	uncultured Lachnospiraceae bacterium	SAMEA8804990	host-associated	11	Yes	4	DUF6849;SWIM;NMO;ACCA	—
297314.SAMEA8804971.CAJTJZ0100	typeXIII	307,2	high	Bacillota	Clostridia	Lachnospirales	Lachnospiraceae	—	uncultured Lachnospiraceae bacterium	SAMEA8804971	host-associated	11	Yes	4	GAIN_ADGRA3;SWIM;MCPsignal;	—
297314.SAMEA8805211.CAJTRU0100	typeXIII	306,8	high	Bacillota	Clostridia	Lachnospirales	Lachnospiraceae	—	uncultured Lachnospiraceae bacterium	SAMEA8805211	—	9	Yes	0	—	—
297314.SAMEA8805591.CAJUGJ0100	typeXIII	306,8	high	Bacillota	Clostridia	Lachnospirales	Lachnospiraceae	—	uncultured Lachnospiraceae bacterium	SAMEA8805591	host-associated	11	Yes	4	HTH_28;Fic;SWIM;OrfB_IS605	—
297314.SAMEA8805451.CAJUEI0100	typeXIII	306,4	high	Bacillota	Clostridia	Lachnospirales	Lachnospiraceae	—	uncultured Lachnospiraceae bacterium	SAMEA8805451	—	6	Yes	0	—	—
297314.SAMEA8805268.CAJTXA0100	typeXIII	306,3	high	Bacillota	Clostridia	Lachnospirales	Lachnospiraceae	—	uncultured Lachnospiraceae bacterium	SAMEA8805268	—	11	Yes	0	—	—
297314.SAMEA8805166.CAJTUU0100	typeXIII	305,8	high	Bacillota	Clostridia	Lachnospirales	Lachnospiraceae	—	uncultured Lachnospiraceae bacterium	SAMEA8805166	host-associated	11	Yes	4	WYL;SWIM;DUF6849	—
297314.SAMEA8805034.CAJTNT0100	typeXIII	305,3	high	Bacillota	Clostridia	Lachnospirales	Lachnospiraceae	—	uncultured Lachnospiraceae bacterium	SAMEA8805034	host-associated	11	Yes	4	ATP_bind_3;LapA_dom;SWIM;DU	—
446043.SAMEA8805821.CAJUTV0100	typeXIII	305,3	high	Bacillota	Clostridia	Lachnospirales	Lachnospiraceae	Lachnospira	uncultured Lachnospira sp.	SAMEA8805821	—	11	Yes	0	—	—
297314.SAMEA8805562.CAJUHN0100	typeXIII	305,2	high	Bacillota	Clostridia	Lachnospirales	Lachnospiraceae	—	uncultured Lachnospiraceae bacterium	SAMEA8805562	host-associated	6	Yes	2	SWIM;DUF6849	—
297314.SAMEA8805329.CAJTXH0100	typeXIII	304,7	high	Bacillota	Clostridia	Lachnospirales	Lachnospiraceae	—	uncultured Lachnospiraceae bacterium	SAMEA8805329	host-associated	8	Yes	4	UvrD-helicase;DUF2157;SWIM;DU	—
297314.SAMEA8805824.CAJUSO0100	typeXIII	304,4	high	Bacillota	Clostridia	Lachnospirales	Lachnospiraceae	—	uncultured Lachnospiraceae bacterium	SAMEA8805824	host-associated	11	Yes	4	DUF4179;ATP_bind_3;SWIM;DUF	—
34034.SAMEA5364575.CAADGB01000	typeXIII	304,1	high	—	Deltaproteobacteria	—	—	—	uncultured delta proteobacterium	SAMEA5364575	temperature	6	Yes	4	DNA_ligase_OB_2;WGR;SWIM;HE	—
297314.SAMEA8805835.CAJUPJ0100	typeXIII	303,2	high	Bacillota	Clostridia	Lachnospirales	Lachnospiraceae	—	uncultured Lachnospiraceae bacterium	SAMEA8805835	host-associated	11	Yes	4	UvrD-helicase;DUF2157;SWIM;DU	—
100371.SAMEA6947806.CAIJGL01000	typeXIII	299,5	high	Planctomycetota	Planctomycetia	Planctomycetales	—	—	uncultured Planctomycetales bacterium	SAMEA6947806	aquatic	11	Yes	4	SpoIIE;MR_MLE_C;HEAT_2;PTP-I	—
2800791.SAMN17140641.JAFAZH0100	typeXIII	298,8	high	Abditibacteriota	Abditibacteriia	Abditibacteriales	Abditibacteriaceae	—	Abditibacteriaceae bacterium	SAMN17140641	host-associated	11	Yes	4	SWIM;PSD4;HXXSHH	—
642492.SAMN00013959.CP002582_83	typeXIII	298,5	high	Bacillota	Clostridia	Lachnospirales	Cellulosilyticaceae	Cellulosilyticum	Cellulosilyticum lentocellum	SAMN00013959	host-associated	11	Yes	4	Flagellin_N;tRNA-synt_2b;SWIM;D	—
360056.SAMN09639612.DSVQ010000	typeXIII	298,3	high	Planctomycetota	Planctomycetia	Planctomycetales	Planctomycetaceae	Schlesneria	Schlesneria paludicola	SAMN09639612	temperature	11	Yes	4	GHMP_kinases_N;SGSH_C;DUF1	—
2052181.SAMD00166091.BJHJ010000	typeXIII	298,2	high	Planctomycetota	Planctomycetia	—	—	—	Planctomycetia bacterium	SAMD00166091	aquatic	9	Yes	4	CbiN;BD-FAE	—
2485925.SAMN12581690.WQUF01000	typeXIII	297,3	high	Bacillota	Clostridia	Eubacteriales	Oscillospiraceae	—	Oscillospiraceae bacterium	SAMN12581690	host-associated	6	Yes	2	PIN	—
48.SAMN07426514.QFQP01000022_1	typeXIII	291,9	high	Myxococcota	Myxococcia	Myxococcales	Archangiaceae	Archangium	Archangium gephyra	SAMN07426514	anthropogenic	11	Yes	4	Peptidase_M54;YrhK;SWIM	—
100233.SAMEA6952302.CAITYU01000	typeXIII	291,4	high	Planctomycetota	Planctomycetia	Planctomycetales	Planctomycetaceae	—	uncultured Planctomycetaceae bacteri	SAMEA6952302	aquatic	6	Yes	3	DUF7474	—
2049045.SAMN12582053.WRIE010000	typeXIII	290,6	high	Bacillota	Clostridia	Eubacteriales	Eubacteriaceae	—	Eubacteriaceae bacterium	SAMN12582053	host-associated	11	Yes	4	HATPase_c;SAICAR_synt;SWIM;D	—
2073117.SAMN15013936.JABTTZ0100	typeXIII	290,3	high	Chloroflexota	Anaerolineae	Anaerolineales	—	—	Anaerolineales bacterium	SAMN15013936	temperature	11	Yes	4	Peptidase_S51;Peripla_BP_2;DUF	—
2026780.SAMN17496266.JAFMMM010	typeXIII	289,1	high	Planctomycetota	—	—	—	—	Planctomycetota bacterium	SAMN17496266	aquatic	6	Yes	2	ACP_syn_III_C;GFO_IDH_MocA_(	—
1952931.SAMN06452880.DIXK010000	typeXIII	287,8	high	Verrucomicrobiota	Verrucomicrobiia	—	—	—	Verrucomicrobia subdivision 3 bacteriu	SAMN06452880	anthropogenic	8	Yes	4	SWIM;GFO_IDH_MocA	—
257465.SAMEA6953639.CAINLR01000	typeXIII	287,5	high	Verrucomicrobiota	Verrucomicrobiia	Limisphaerales	—	—	uncultured Verrucomicrobia subdivisio	SAMEA6953639	aquatic	11	Yes	4	SWIM;NAPRTase_N;NAD_synthas	—

2052179.SAMN12582057.WRII0100002	**typeXIII**	285,2	high	Bacillota	Clostridia	Eubacteriales	Peptococcaceae	—	Peptococcaceae bacterium	SAMN12582057	host-associated	11	Yes	4	DUF6849;SWIM;DA_C	—
2026780.SAMN09639629.DSWH01000	**typeXIII**	285	high	Planctomycetota	—	—	—	—	Planctomycetota bacterium	SAMN09639629	temperature	11	Yes	4	N6_N4_Mtase;GFO_IDH_MocA;PII	—
2026780.SAMN09639583.DSUN010000	**typeXIII**	285	high	Planctomycetota	—	—	—	—	Planctomycetota bacterium	SAMN09639583	temperature	11	Yes	4	Glyco_hydro_10;NPCBM;GFO_IDH	—
156588.SAMEA6944372.CAIMXY01000	**typeXIII**	281,9	high	Verrucomicrobiota	—	—	—	—	uncultured Verrucomicrobiota bacteriu	SAMEA6944372	aquatic	10	Yes	4	SWIM;TIR_2	—
2052186.SAMN11532986.VKHA010000	**typeXIII**	280,5	high	Verrucomicrobiota	Verrucomicrobiia	Limisphaerales	—	—	Limisphaerales bacterium	SAMN11532986	terrestrial	7	Yes	3	SWIM	—
2026763.SAMN10607053.SKYM010002	**typeXIII**	279,1	high	Myxococcota	Myxococcia	Myxococcales	—	—	Myxococcales bacterium	SAMN10607053	salinity	8	Yes	4	Radical_SAM;IPPT	—
2026735.SAMN10966762.VGTK010002	**typeXIII**	274,5	high	Myxococcota	Myxococcia	—	—	—	Deltaproteobacteria bacterium	SAMN10966762	aquatic	5	Yes	4	SWIM;DUF2779;FGE-sulfatase	—
100233.SAMEA6956128.CAIXVO01000	**typeXIII**	272,2	high	Planctomycetota	Planctomycetia	Planctomycetales	Planctomycetaceae	—	uncultured Planctomycetaceae bacteri	SAMEA6956128	aquatic	3	Yes	2	N6_N4_Mtase	—
1913988.SAMN17181029.JAEUML0100	**typeXIII**	269,7	high	Pseudomonadota	Alphaproteobacteria	—	—	—	Alphaproteobacteria bacterium	SAMN17181029	anthropogenic	5	Yes	3	SWIM;tRNA-synt_1g	—
1951185.SAMN06451746.DDIR010000	**typeXIII**	257,9	high	Planctomycetota	Phycisphaerae	Phycisphaerales	—	—	Phycisphaerales bacterium UBA1845	SAMN06451746	anthropogenic	11	Yes	4	NUDIX;Aminotran_5;CoA_trans;ME	—
297314.SAMEA8804971.CAJTJZ01000	**typeXIII**	255,2	high	Bacillota	Clostridia	Lachnospirales	Lachnospiraceae	—	uncultured Lachnospiraceae bacterium	SAMEA8804971	host-associated	8	Yes	0	—	—
2026763.SAMN17180967.JAEUKB0100	**typeXIII**	254,1	high	Myxococcota	Myxococcia	Myxococcales	—	—	Myxococcales bacterium	SAMN17180967	anthropogenic	11	Yes	4	DUF4139;Polysacc_synt_3;Aldo_ke	—
1632864.SAMN03417854.CP011270_1	**typeXIII**	250,7	high	Planctomycetota	Planctomycetia	Planctomycetales	Planctomycetaceae	Planctomyces	Planctomyces sp. SH-PL14	SAMN03417854	anthropogenic	11	Yes	4	DUF899;DUF7569;PSD1;HEAT_2	—
2052164.SAMN08179277.PLDW01000	**typeXIII**	248,1	high	Planctomycetota	Planctomycetia	Gemmatales	Gemmataceae	—	Gemmataceae bacterium	SAMN08179277	temperature	11	Yes	4	Pkinase;DUF3024;FGE-sulfatase	—
100233.SAMEA6946666.CAIVBC01000	**typeXIII**	240,6	high	Planctomycetota	Planctomycetia	Planctomycetales	Planctomycetaceae	—	uncultured Planctomycetaceae bacteri	SAMEA6946666	aquatic	3	Yes	2	SWIM	—
2026780.SAMN11533026.WSZJ010001	**typeXIII**	227,5	high	Planctomycetota	—	—	—	—	Planctomycetota bacterium	SAMN11533026	terrestrial	9	Yes	4	HEAT_2;DNA_pol3_alpha;Beta-pro	—
2094028.SAMN13893733.JAAYEJ0100	**typeXIII**	219,1	low	Planctomycetota	Candidatus Brocadiia	Candidatus Brocadiales	Candidatus Brocadiaceae	—	Candidatus Brocadiaceae bacterium	SAMN13893733	temperature	7	Yes	4	Lipase_GDSL_2;DUF4011;Creatini	—
1129257.SAMEA6153040.CADCRA010	**typeXIII**	218,6	low	Bacteroidota	Bacteroidia	—	—	—	uncultured Bacteroidia bacterium	SAMEA6153040	host-associated	10	Yes	4	FtsK_SpoIIIE;TIR_2	—
2026780.SAMN09639622.DSWA01000	**typeXIII**	217,7	low	Planctomycetota	—	—	—	—	Planctomycetota bacterium	SAMN09639622	temperature	10	Yes	4	GH123_N;SGL;Alpha_L_fucos;GH	—
2026779.SAMN13893644.JAAYAY0100	**typeXIII**	215,8	low	Planctomycetota	Planctomycetia	Planctomycetales	Planctomycetaceae	—	Planctomycetaceae bacterium	SAMN13893644	temperature	6	Yes	3	TGT;RNA_pol_A_CTD	—
2026779.SAMN14914873.JABIHZ01000	**typeXIII**	213,7	low	Planctomycetota	Planctomycetia	Planctomycetales	Planctomycetaceae	—	Planctomycetaceae bacterium	SAMN14914873	aquatic	9	Yes	4	Ldh_2;SGL;Lum_binding;DUF6009	—
156588.SAMEA9695016.CAJXYV01000	**typeXIII**	211,3	low	Verrucomicrobiota	—	—	—	—	uncultured Verrucomicrobiota bacteriu	SAMEA9695016	anthropogenic	10	Yes	4	Metallophos;AAA_23;DUF3293;Ser	—
2840714.SAMN15817152.DVNY010001	**typeXIII**	209,5	low	Bacteroidota	Bacteroidia	Bacteroidales	Prevotellaceae	Candidatus Caccomon	Candidatus Caccomonas pullistercoris	SAMN15817152	host-associated	6	Yes	3	DUF7017;AAA_30	—
1262935.PRJEB997.HF989231_22	**typeXIII**	207,6	low	Bacteroidota	Bacteroidia	Bacteroidales	Prevotellaceae	Prevotella	Prevotella sp. CAG:755	PRJEB997	—	10	Yes	0	—	—
2026778.SAMN13893681.JAAYCJ0100	**typeXIII**	201,5	low	Planctomycetota	Phycisphaerae	—	—	—	Phycisphaerae bacterium	SAMN13893681	temperature	1	Yes	1	URO-D	—
2723666.SAMN14513815.CP050848_3	**typeXIII**	193,7	low	Planctomycetota	Planctomycetia	Planctomycetales	—	—	Planctomycetales bacterium ZRK34	SAMN14513815	aquatic	10	Yes	4	Secretin;CPSase_sm_chain;MgtE;A	—
2026780.SAMN09724347.QWPT01000	**typeXIII**	183,5	low	Planctomycetota	—	—	—	—	Planctomycetota bacterium	SAMN09724347	temperature	10	Yes	4	AAA_5;DUF2201;LTI65_LTI78_NY	—
286133.SAMEA104667185.OMXR0100	**typeXIII**	160,8	low	Pseudomonadota	Betaproteobacteria	Burkholderiales	Sutterellaceae	Sutterella	uncultured Sutterella sp.	SAMEA104667185	host-associated	10	Yes	4	AAA_5;DUF2201_N;N6_N4_Mtase	—
2021391.SAMN07620311.PBEC010000	**typeXIII**	158	low	Bacteroidota	Flavobacteriia	Flavobacteriales	—	—	Flavobacteriales bacterium	SAMN07620311	aquatic	5	Yes	2	iREC;HisKin-conflict	—
40545.SAMN17800730.JAGZDF010000	**typeXIII**	149,6	low	Pseudomonadota	Betaproteobacteria	Burkholderiales	Sutterellaceae	Sutterella	Sutterella wadsworthensis	SAMN17800730	host-associated	10	Yes	4	PSF2_N;SNF2-rel_dom;Radical_SA	—
**TOTAL: 1,286 candidates | 934 high | 25 borderline | 327 low**																
