# Supplementary material for: Landscape of retron diversity across the SPIRE microbial metagenome resource reveals candidate novel type XI-like lineages
Source: mSystems. 2026 Sep 11;11(10):e00732-26. doi: 10.1128/msystems.00732-26 (PMC13712802; doi:10.1128/msystems.00732-26)
Supplement: Supplementary file 4 [file msystems.00732-26-s0006.pdf]

## Supplementary Table S3. Retron group enrichment by taxonomic order (standardized Pearson residuals ≥ 2)

Chi-square test of independence, group × order contingency table (order n≥5, group n≥10; 898 of 1,286 candidates, 21 groups × 35 orders): $\chi 2$ = 3438.3, d.f. = 680, $p < 1\times10^{-300}$. Rows are group–order pairs with a standardized Pearson residual [(observed − expected) / √expected] ≥ 2, sorted by group then descending residual.

| Retron group | Taxonomic order | Observed (n) | Expected (n) | Standardized Pearson residual |
|---|---|---|---|---|
| clade11-A | Micrococcales | 9 | 0,55 | 11,38 |
| clade11-A | Bryobacterales | 7 | 0,35 | 11,23 |
| clade11-A | Burkholderiales | 16 | 3,11 | 7,31 |
| clade11-A | Planctomycetales | 6 | 1,90 | 2,97 |
| clade11-B | Mycobacteriales | 18 | 0,63 | 21,80 |
| clade11-B | Planctomycetales | 11 | 1,27 | 8,64 |
| clade2_Ec107like | Eubacteriales | 83 | 27,07 | 10,75 |
| clade2_Ec107like | Lachnospirales | 63 | 22,41 | 8,57 |
| clade2_Ec107like | Erysipelotrichales | 8 | 1,61 | 5,03 |
| typeI-A | Vibrionales | 3 | 0,08 | 10,09 |
| typeI-A | Moraxellales | 2 | 0,15 | 4,77 |
| typeI-A | Oceanospirillales | 2 | 0,15 | 4,77 |
| typeI-A | Alteromonadales | 2 | 0,18 | 4,24 |
| typeI-A | Chromatiales | 1 | 0,10 | 2,84 |
| typeI-A | Nitrosomonadales | 1 | 0,10 | 2,84 |
| typeI-B2 | Lachnospirales | 9 | 2,23 | 4,54 |
| typeI-B2 | Bacillales | 1 | 0,16 | 2,10 |
| typeI-B2 | Eubacteriales | 6 | 2,69 | 2,02 |
| typeI-C1 | Enterobacterales | 6 | 0,74 | 6,10 |
| typeI-C1 | Aeromonadales | 2 | 0,13 | 5,21 |
| typeI-C1 | Hyphomicrobiales | 5 | 0,94 | 4,20 |
| typeI-C1 | Moraxellales | 2 | 0,29 | 3,17 |
| typeI-C1 | Burkholderiales | 6 | 2,00 | 2,83 |
| typeI-C1 | Caulobacterales | 1 | 0,16 | 2,09 |
| typeI-C2 | Lactobacillales | 8 | 0,39 | 12,19 |
| typeI-C2 | Eubacteriales | 13 | 4,20 | 4,29 |
| typeI-C2 | Campylobacterales | 1 | 0,17 | 2,04 |
| typeI-C3 | Campylobacterales | 3 | 0,06 | 11,99 |
| typeI-C3 | Bacteroidales | 5 | 1,93 | 2,20 |
| typeII-A1 | Sphingomonadales | 6 | 0,66 | 6,56 |
| typeII-A1 | Lysobacterales | 4 | 0,33 | 6,38 |
| typeII-A1 | Terriglobales | 2 | 0,18 | 4,24 |
| typeII-A1 | Hyphomicrobiales | 4 | 1,07 | 2,84 |
| typeII-A1 | Rhodospirillales | 1 | 0,15 | 2,22 |
| typeII-A3 | Chromatiales | 1 | 0,10 | 2,84 |
| typeII-A3 | Enterobacterales | 2 | 0,38 | 2,61 |
| typeII-A3 | Lachnospirales | 5 | 2,09 | 2,02 |
| typeIII-A1 | Flavobacteriales | 8 | 1,40 | 5,58 |
| typeIII-A1 | Eubacteriales | 12 | 5,72 | 2,63 |
| typeIII-A2 | Bacteroidales | 28 | 7,74 | 7,28 |

## Supplementary Table S3. Retron group enrichment by taxonomic order (standardized Pearson residuals ≥ 2)

Chi-square test of independence, group × order contingency table (order n≥5, group n≥10; 898 of 1,286 candidates, 21 groups × 35 orders): $\chi2 = 3438.3$, d.f. = 680, $p < 1\times10^{-300}$. Rows are group–order pairs with a standardized Pearson residual [(observed − expected) / √expected] ≥ 2, sorted by group then descending residual.

| Retron group | Taxonomic order | Observed (n) | Expected (n) | Standardized Pearson residual |
|---|---|---|---|---|
| typeIII-A2 | Moraxellales | 3 | 0,36 | 4,39 |
| typeIII-A2 | Aeromonadales | 1 | 0,16 | 2,10 |
| typeIII-A3 | Hyphomicrobiales | 6 | 1,10 | 4,68 |
| typeIII-A3 | Desulfovibrionales | 3 | 0,42 | 4,00 |
| typeIII-A3 | Alteromonadales | 3 | 0,42 | 4,00 |
| typeIII-A3 | Chromatiales | 2 | 0,23 | 3,72 |
| typeIII-A3 | Lysobacterales | 2 | 0,34 | 2,84 |
| typeIII-A3 | Rhodobacterales | 2 | 0,42 | 2,45 |
| typeIII-A3 | Micrococcales | 2 | 0,42 | 2,45 |
| typeIII-A4 | Flavobacteriales | 6 | 0,87 | 5,52 |
| typeIII-A4 | Bacteroidales | 15 | 4,51 | 4,94 |
| typeIII-A5 | Bacillales | 3 | 0,37 | 4,32 |
| typeIII-A5 | Lachnospirales | 11 | 5,15 | 2,58 |
| typeIII-A5 | Rhodospirillales | 1 | 0,16 | 2,06 |
| typeV | Aeromonadales | 1 | 0,04 | 4,53 |
| typeV | Pseudomonadales | 2 | 0,19 | 4,16 |
| typeV | Vibrionales | 1 | 0,06 | 4,00 |
| typeV | Burkholderiales | 4 | 0,69 | 3,98 |
| typeV | Alteromonadales | 1 | 0,12 | 2,51 |
| typeVI | Nitrosomonadales | 3 | 0,46 | 3,74 |
| typeVI | Desulfovibrionales | 4 | 0,85 | 3,43 |
| typeVI | Oceanospirillales | 3 | 0,69 | 2,78 |
| typeVI | Terriglobales | 2 | 0,38 | 2,61 |
| typeX | Verrucomicrobiales | 3 | 0,07 | 11,06 |
| typeX | Planctomycetales | 4 | 0,38 | 5,86 |
| typeX | Rhodobacterales | 1 | 0,11 | 2,68 |
| typeXI | Bacteroidales | 113 | 35,03 | 13,17 |
| typeXI | Chitinophagales | 4 | 1,45 | 2,11 |
| typeXII | Lysobacterales | 3 | 0,15 | 7,35 |
| typeXII | Enterobacterales | 3 | 0,38 | 4,22 |
| typeXII | Caulobacterales | 1 | 0,08 | 3,17 |
| typeXII | Campylobacterales | 1 | 0,10 | 2,84 |
| typeXII | Oceanospirillales | 1 | 0,15 | 2,19 |
| typeXIII | Myxococcales | 7 | 0,92 | 6,33 |
| typeXIII | Oscillatoriales | 4 | 0,61 | 4,32 |
| typeXIII | Planctomycetales | 12 | 3,89 | 4,11 |
| typeXIII | Lachnospirales | 25 | 12,81 | 3,41 |
| typeXIII | Burkholderiales | 13 | 6,35 | 2,64 |
| typeXIII | Chitinophagales | 3 | 0,82 | 2,41 |
| typeXIII | Hyphomicrobiales | 7 | 2,97 | 2,34 |
