# Supplementary material for: Landscape of retron diversity across the SPIRE microbial metagenome resource reveals candidate novel type XI-like lineages
Source: mSystems. 2026 Sep 11;11(10):e00732-26. doi: 10.1128/msystems.00732-26 (PMC13712802; doi:10.1128/msystems.00732-26)
Supplement: Supplementary file 5 [file msystems.00732-26-s0007.pdf]

Supplementary Table S4. Detailed host-organism and biome associations of host-associated SPIRE retron candidates

Host-associated candidates (n=660 of 1,286) were assigned to finer-resolution host/biome categories using the SPIRE biome ontology field associated with each candidate. Panel A summarizes the overall category breakdown; Panel B shows the retron-group composition of the explicit human-host category (n=220); Panel C shows its phylum composition; Panel D details the taxonomic order and genus composition of Bacillota retron-containing genomes (n=361 total; 318 host-associated).

A. Host-associated candidates by biome category (n = 660)

Host / biome category	n candidates	% of host-associated (n=660)
animal host: digestive tract (non-human/unspecified)	299	45.3%
animal host: human	220	33.3%
animal host: digestive tract (rumen)	66	10.0%
plant host: rhizosphere	37	5.6%
animal/plant host: unspecified	13	2.0%
plant host: phyllosphere	8	1.2%
animal host: insect	8	1.2%
plant host: unspecified	5	0.8%
plant host: litter	3	0.5%
animal host: coral	1	0.2%

B. Human-host candidates by retron group (n = 220)

Retron group	n candidates	% of human-host (n=220)
clade2_Ec107like	65	29.5%
typeXI	33	15.0%
typeVI	20	9.1%
typeI-C2	18	8.2%
typeIII-A2	17	7.7%
typeIII-A1	14	6.4%
typeIII-A5	10	4.5%
typeI-C1	7	3.2%
typeXIII	6	2.7%
typeII-A3	6	2.7%
typeI-B2	5	2.3%
typeII-A1	4	1.8%
clade11-B	3	1.4%
typeIII-A3	3	1.4%
typeI-A	3	1.4%
typeIII-A4	2	0.9%
typeXII	2	0.9%
typeII-A2	1	0.5%
typeV	1	0.5%

C. Human-host candidates by phylum (n = 220)

Phylum	n candidates	% of human-host (n=220)
Bacillota	124	56.4%

Bacteroidota	53	24.1%
Pseudomonadota	25	11.4%
Actinomycetota	6	2.7%
Mycoplasmatota	4	1.8%
Lentisphaerota	2	0.9%
Spirochaetota	2	0.9%
Campylobacterota	1	0.5%
Fusobacteriota	1	0.5%
Thermodesulfobacteriota	1	0.5%

D1. Bacillota retron-containing genomes by taxonomic order (n = 361)

Taxonomic order	n candidates	% of Bacillota (n=361)
Eubacteriales	151	41.8%
Lachnospirales	125	34.6%
Lactobacillales	14	3.9%
Bacillales	9	2.5%
Erysipelotrichales	9	2.5%
Peptostreptococcales	3	0.8%
Acetivibrionales	2	0.6%
Veillonellales	2	0.6%
Halanaerobiales	2	0.6%
Selenomonadales	1	0.3%

D2. Bacillota retron-containing genomes by genus (n = 361)

Genus	n candidates	% of Bacillota (n=361)
Ruminococcus	21	5.8%
Clostridium	19	5.3%
Oscillibacter	9	2.5%
Eubacterium	8	2.2%
Blautia	8	2.2%
Lacrimispora	7	1.9%
Hungatella	6	1.7%
Faecalibacterium	6	1.7%
Lachnoclostridium	5	1.4%
Eisenbergiella	5	1.4%
Streptococcus	5	1.4%
Enterocloster	3	0.8%
Intestinimonas	3	0.8%
Anaerotruncus	3	0.8%
Enterococcus	3	0.8%
Anaeromassilibacillus	3	0.8%
Robinsoniella	3	0.8%
Lysinibacillus	2	0.6%
Cellulosilyticum	2	0.6%
Tyzzerella	2	0.6%
Lachnospira	2	0.6%

Dorea	2	0.6%
Acetivibrio	2	0.6%
Butyrivibrio	2	0.6%
Veillonella	2	0.6%
Faecalicatena	2	0.6%
Butyricicoccus	2	0.6%
Pediococcus	2	0.6%
Flavonifractor	2	0.6%
Pseudoflavonifractor	2	0.6%
Subdoligranulum	2	0.6%
Anaerofustis	2	0.6%
Peptoanaerobacter	1	0.3%
Staphylococcus	1	0.3%
Coprococcus	1	0.3%
Caproicibacterium	1	0.3%
Schaedlerella	1	0.3%
Candidatus Avimonas	1	0.3%
Bariatricus	1	0.3%
Carnobacterium	1	0.3%
Jingyaoa	1	0.3%
Wansuia	1	0.3%
Flintibacter	1	0.3%
Yanshouia	1	0.3%
Candidatus Fimenecus	1	0.3%
Lactonifactor	1	0.3%
Cuneatibacter	1	0.3%
Otoolea	1	0.3%
Mediterraneibacter	1	0.3%
Faecalimonas	1	0.3%
Solobacterium	1	0.3%
Aerococcus	1	0.3%
Candidatus Metaruminococcus	1	0.3%
Paenibacillus	1	0.3%
Candidatus Aphodocola	1	0.3%
Macrococcoides	1	0.3%
Lacticaseibacillus	1	0.3%
Candidatus Avoscillospira	1	0.3%
Exiguobacterium	1	0.3%
Candidatus Stoquefichus	1	0.3%
Rummeliibacillus	1	0.3%
Halonatronomonas	1	0.3%
Pseudocoprococcus	1	0.3%
Halanaerobium	1	0.3%
Pseudobutyrivibrio	1	0.3%
Candidatus Howiella	1	0.3%
Candidatus Avispirillum	1	0.3%
Ruminiclostridium	1	0.3%
Schleiferilactobacillus	1	0.3%

Claveliimonas	1	0.3%

D3. Bacillota retron-containing genomes by host/biome category (n = 318 host-associated)

Host / biome category	n candidates	% of Bacillota host-associated (n=318)
animal host: digestive tract (non-human/unspecified)	165	51.9%
animal host: human	124	39.0%
animal host: digestive tract (rumen)	19	6.0%
animal/plant host: unspecified	8	2.5%
plant host: rhizosphere	2	0.6%
