# Supplementary material for: Landscape of retron diversity across the SPIRE microbial metagenome resource reveals candidate novel type XI-like lineages
Source: mSystems. 2026 Sep 11;11(10):e00732-26. doi: 10.1128/msystems.00732-26 (PMC13712802; doi:10.1128/msystems.00732-26)
Supplement: Supplementary file 6 [file msystems.00732-26-s0008.pdf]

Supplementary Table S5. Systematic evidence review of all 28 SPIRE retron classification groups. For each group, five evaluation criteria are scored: absent (-), weak (+), moderate (++), strong (+++). Scores were assigned using uniform quantitative thresholds applied across all groups. The composite novelty divergence score (none/low/medium/high) integrates all five criteria. Groups marked as 'known-type expansion' were excluded from the curated evidence matrix (Figure 3A) as they displayed fully canonical characteristics providing no discriminatory information.

Retron group	N total	N high (%)	N border (%)	N low (%)	Top phylum	Top biome	RT phylogenetic coherence	Accessory architecture	Confidence support	Taxonomic coherence	Ecological coherence	Novelty divergence	Review classification
**typeXI**	232	65 (28%)	0 (0%)	167 (72%)	Bacteroidota	host-associated	+++	+++	++	+++	++	high	**candidate Type XI-like lineage**
**clade2_Ec107like**	192	188 (98%)	0 (0%)	4 (2%)	Bacillota	host-associated	++	−	+++	++	++	low	known-like/minimal expansion
**typeXIII**	143	129 (90%)	0 (0%)	14 (10%)	Bacillota	host-associated	++	++	+++	+++	++	medium	known-type expansion
**typeVI**	93	90 (97%)	0 (0%)	3 (3%)	Bacteroidota	host-associated	—	—	—	—	—	—	known-type expansion
**clade11-A**	71	40 (56%)	0 (0%)	31 (44%)	Pseudomonadota	host-associated	−	−	+	−	−	none	heterogeneous/low priority
**typeIII-A5**	70	32 (46%)	0 (0%)	38 (54%)	Bacillota	host-associated	+	++	+	+	+	low	known-type expansion
**typeIII-A1**	50	43 (86%)	0 (0%)	7 (14%)	Bacillota	host-associated	—	—	—	—	—	—	known-type expansion
**typeII-A1**	47	46 (98%)	0 (0%)	1 (2%)	Pseudomonadota	host-associated	—	—	—	—	—	—	known-type expansion
**typeIII-A2**	42	30 (71%)	0 (0%)	12 (29%)	Bacteroidota	host-associated	—	—	—	—	—	—	known-type expansion
**typeI-C1**	44	44 (100%)	0 (0%)	0 (0%)	Pseudomonadota	host-associated	—	—	—	—	—	—	known-type expansion
**typeIII-A3**	40	40 (100%)	0 (0%)	0 (0%)	Pseudomonadota	aquatic	—	—	—	—	—	—	known-type expansion
**clade11-B**	39	18 (46%)	0 (0%)	21 (54%)	Actinomycetota	aquatic	−	−	+	+	+	none	heterogeneous/low priority
**typeI-C2**	31	31 (100%)	0 (0%)	0 (0%)	Bacillota	host-associated	—	—	—	—	—	—	known-type expansion
**typeII-A3**	28	27 (96%)	0 (0%)	1 (4%)	Bacillota	host-associated	—	—	—	—	—	—	known-type expansion
**typeI-B2**	21	9 (43%)	12 (57%)	0 (0%)	Bacillota	host-associated	++	+++	+	++	+	medium	known-type expansion
**typeIII-A4**	21	19 (90%)	2 (10%)	0 (0%)	Bacteroidota	host-associated	—	—	—	—	—	—	known-type expansion
**typeI-A**	21	18 (86%)	0 (0%)	3 (14%)	Pseudomonadota	host-associated	—	—	—	—	—	—	known-type expansion
**typeXII**	21	15 (71%)	0 (0%)	6 (29%)	Pseudomonadota	host-associated	—	—	—	—	—	—	known-type expansion
**typeX**	16	6 (38%)	3 (19%)	7 (44%)	Planctomycetota	aquatic	+	+	+	++	+++	none	low-count known group
**typeI-C3**	14	3 (21%)	2 (14%)	9 (64%)	Bacteroidota	aquatic	+	++	+	++	+	none	low-count known group
**typeV**	11	10 (91%)	1 (9%)	0 (0%)	Pseudomonadota	anthropogenic	+	+++	+++	+++	+	none	low-count known group
**clade11**	9	9 (100%)	0 (0%)	0 (0%)	Actinomycetota	aquatic	—	—	—	—	—	—	known-type expansion
**typeVII-A1**	7	6 (86%)	1 (14%)	0 (0%)	Pseudomonadota	anthropogenic	—	—	—	—	—	—	known-type expansion
**typeII-A2**	7	3 (43%)	3 (43%)	1 (14%)	Pseudomonadota	aquatic	—	—	—	—	—	—	known-type expansion
**typeIX**	5	5 (100%)	0 (0%)	0 (0%)	Pseudomonadota	aquatic	—	—	—	—	—	—	known-type expansion
**typeIV**	5	5 (100%)	0 (0%)	0 (0%)	Pseudomonadota	aquatic	—	—	—	—	—	—	known-type expansion
**typeVII-A2**	4	1 (25%)	1 (25%)	2 (50%)	Pseudomonadota	anthropogenic	—	—	—	—	—	—	known-type expansion
**typeI-B1**	2	2 (100%)	0 (0%)	0 (0%)	Pseudomonadota	aquatic	—	—	—	—	—	—	known-type expansion

Score legend: − (absent) = no evidence; + (weak) = marginal evidence; ++ (moderate) = consistent evidence; +++ (strong) = robust evidence across multiple independent indicators

Note: Groups classified as 'known-type expansion' were not included in the curated evidence matrix (Figure 3A) because they displayed fully canonical characteristics across all criteria, providing no discriminatory information for novelty assessment. Scores marked '—' indicate groups for which detailed criterion-level scoring was not performed as part of the curated review.
